# 1-Zirconacyclobuta-2,3-dienes: synthesis of organometallic analogs of elusive 1,2-cyclobutadiene, unprecedented intramolecular C–H activation, and reactivity studies†

**DOI:** 10.1039/d1sc06052j

**Published:** 2021-11-23

**Authors:** Xinzhe Shi, Sihan Li, Melanie Reiß, Anke Spannenberg, Thorsten Holtrichter-Rößmann, Fabian Reiß, Torsten Beweries

**Affiliations:** Leibniz-Institut für Katalyse e.V. Albert-Einstein-Str. 29a 18059 Rostock Germany fabian.reiss@catalysis.de torsten.beweries@catalysis.de; LANXESS Organometallics GmbH Ernst-Schering-Str. 14 59192 Bergkamen Germany

## Abstract

The structure, bonding, and reactivity of small, highly unsaturated ring systems is of fundamental interest for inorganic and organic chemistry. Four-membered metallacyclobuta-2,3-dienes, also referred to as metallacycloallenes, are among the most exotic examples for ring systems as these represent organometallic analogs of 1,2-cyclobutadiene, the smallest cyclic allene. Herein, the synthesis of the first examples of 1-zirconacyclobuta-2,3-dienes of the type [Cp′_2_Zr(Me_3_SiC_3_SiMe_3_)] (Cp′_2_ = *rac*-(ebthi), (ebthi = 1,2-ethylene-1,1′-bis(η^5^-tetrahydroindenyl)) (2a); *rac*-Me_2_Si(thi)_2_, thi = (η^5^-tetrahydroindenyl), (2b)) is presented. Both complexes undergo selective thermal C–H activation at the 7-position of the *ansa*-cyclopentadienyl ligand to produce a new type of “tucked-in” zirconocene system, 3a and 3b, that possesses a η^3^-propargyl/allenyl ligand. Both types of complexes react with carbonyl compounds, producing enynes in the case of 2a and 2b, as well as η^1^-allenyl complexes for 3a and 3b. Computational analysis of the structure and bonding of 2a and 3a reveals significant differences to a previously described related Ti complex. All complexes were fully characterised, including X-ray crystallography and experimental results were supported by DFT analysis.

## Abstract 2

A detailed study of structure, bonding and reactivity of new 1-zirconacyclobuta-2,3-dienes is presented in comparison to a lighter Ti analog. We found a unique C–H activation at the widely used *rac*-(ebthi) ligand for that only occurs for Zr.

## Graphical abstract

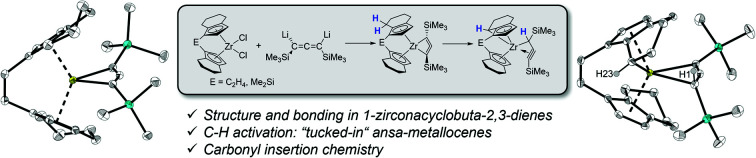

## Introduction

Organometallic complexes of early transition metals show great potential for a variety of unusual bond activation reactions and for the stabilisation of exotic bond situations.^1^ In this context the study of formation and reactivity of unsaturated, unusual five-membered metallacycles such as 1-metallacyclopent-3-ynes,^2^ 1-metallacyclopenta-2,3,4-trienes^3^ or 1-metallacyclopenta-2,3-dienes^4^ has attracted great attention in the past. In these molecules, the metal centre plays an important role for the stability of the seemingly abnormal cyclic geometries, interacting with the central double or triple bond of the metallacyle.^5^ In general, the chemistry of highly strained metallacycles is of particular interest to realise unusual bonding situations that can pave the way to new types of ligand architectures or new chemical transformations. A recent example was reported by Tonks, Goodpaster, Copéret and co-workers, who showed that carbodiimide coordination at Cp_2_Ti(ii) (Cp = η^5^-cyclopentadienyl)^6^ results in the formation of a strained 4-membered nitrogen-containing metallacycle bearing a free carbene.^7^

**Fig. 1:**
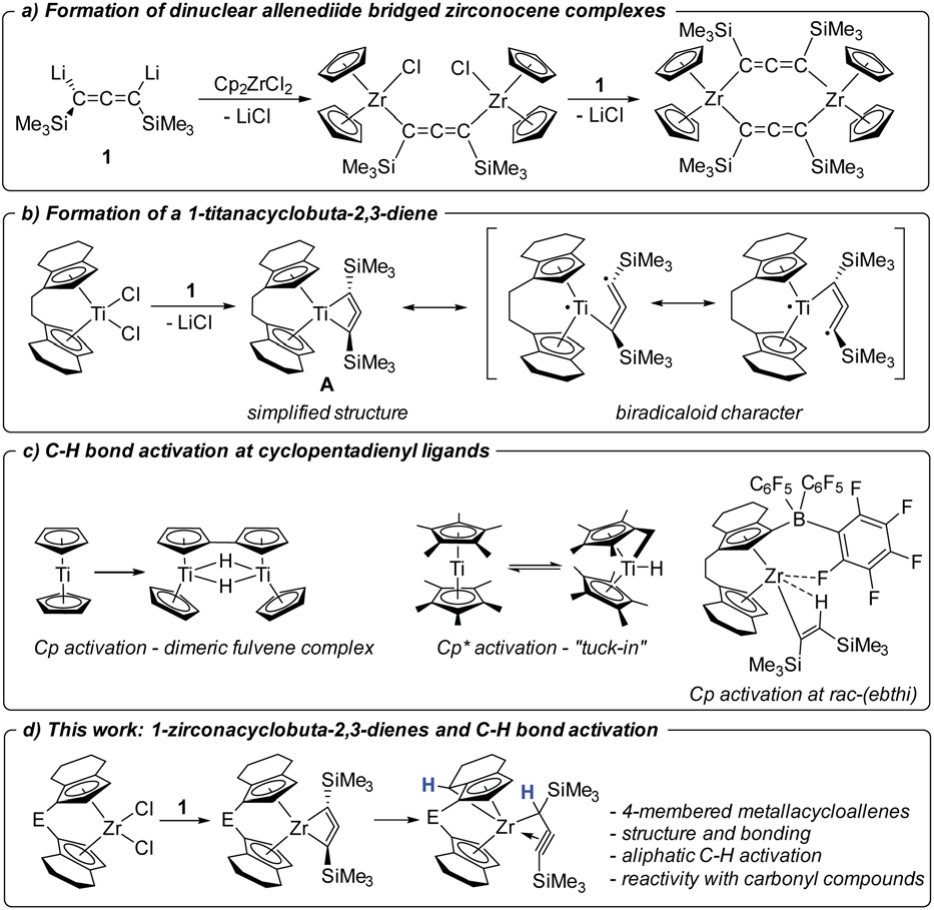
Contextualisation of the present work.

In recent years, we became interested in the synthesis of even smaller highly unsaturated four-membered all-carbon metallacycles and computationally evaluated the possibilities of accessing such structures.^8^ In the past, group 5 metallacyclobutadiene complexes have been reported as intermediates and deactivation products in alkyne metathesis.^9–11^ Following up on several unsuccessful approaches, such as attempted coupling of alkynyl and isocyanide ligands at Ti(iii),^12^ or deprotonation of a promising propyne precursor,^13^ we have presented the synthesis and isolation of a dilithiated allene synthon [Li_2_(Me_3_SiC_3_SiMe_3_)] (1) that could furnish the desired 1-metallacyclobuta-2,3-diene complexes in a simple salt metathesis reaction with metallocene dihalide.^14*a*^ However, in reactions with [Cp_2_ZrCl_2_] and [Cp_2_HfCl_2_] only dinuclear, allenediide bridged metallocene complexes could be obtained (Fig. 1a).^14^

Recently reactions of the *ansa*-titanocene [*rac*-(ebthi)TiCl_2_] (ebthi = 1,2-ethylene-1,1′-bis(η^5^-tetrahydroindenyl)) with 1 resulted in the formation of the unusual metallacycle A (Fig. 1b).^15^ This compound is best described as an unusual biradicaloid system, possessing a formal Ti(iii) centre that is antiferromagnetically coupled with a monoanionic radical ligand. First studies of the reactivity showed that A selectively reacts with ketones and aldehydes to yield enynes by oxygen transfer to titanium.

Bridged *ansa*-metallocenes such as *rac*-(ebthi)M (M = Ti, Zr, Hf) were first developed by Brintzinger^16^ and were found to show excellent performance in the stereospecific synthesis of polyolefins.^17^ Additionally these and related systems were used as catalysts for a variety of stereoselective synthetic applications.^18^ Activation reactions of the metallocene framework that result in deactivation of the catalyst or could open pathways for undesired side-reactions are typically not considered. In the organometallic chemistry of group 4 metallocenes, intramolecular aliphatic C–H activations at non-Cp containing alkyl groups have been reported before.^19^ C–H activation reactions at the metallocene fragment include the well-studied case of Cp* (Cp* = η^5^-C_5_Me_5_) ‘‘tuck(ed)-in’’^20^, for example, forming a hydride complex [(Cp*)(C_5_Me_4_CH_2_)TiH] from [Cp*_2_Ti] (Fig. 1c). The aromatic C–H activation at Cp ligands is rather uncommon, although a classical example has been described for titanium, where free “titanocene” is in fact the doubly C–H activated dimeric species [((Cp)(C_5_H_4_)TiH)_2_].^21^ In 2003 Rosenthal reported an unusual aromatic C–H activation of the *rac*-(ebthi) ligand at Zr in the presence of the Lewis acid [B(C_6_F_5_)_3_] (Fig. 1c).^22^ In addition, intermolecular C–H activation reactions are involved as key steps in the activation and coupling of small molecules at Ti and Zr complexes.^23^

In this contribution, we present the synthesis and characterisation of two Zr analogs of the Ti complex A as well as their transformation into unprecedented aliphatic C–H activation products (Fig. 1d). Furthermore, the reactivity of these complexes with carbonyl compounds is discussed in comparison with the Ti system. Finally, we attempt to rationalise the selective formation of metallacycles for the herein described examples and discuss this in the context of previous work on related zirconocenes.

## Results and discussion

### Synthesis and characterisation of 1-zirconacyclobuta-2,3-dienes

**Scheme 1:**
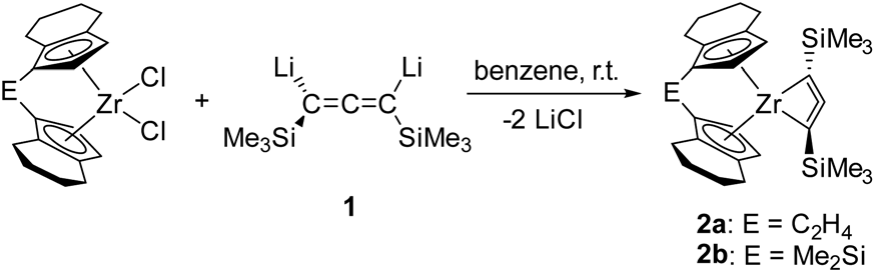
Synthesis of 1-zirconacyclobuta-2,3-diene complexes 2a and 2b.

Reaction of [*rac*-(ebthi)ZrCl_2_] and the dilithiated allene 1 at room temperature in non-polar solvents such as benzene or toluene furnishes complex 2a, the zirconocene analog of the previously described Ti complex A (Scheme 1). Similarly, the reaction of the dimethylsilyl bridged complex [Me_2_Si(thi)_2_ZrCl_2_] (thi = η^5^-tetrahydroindenyl) with 1 furnishes the corresponding complex 2b. Both complexes were characterised by NMR spectroscopy and their ^1^H NMR spectra show informative doublet resonances which correspond to the Cp protons (2a: *d* 7.20, 5.35, 2b: *d* 7.43, 5.40 ppm). In ^13^C NMR spectra, the signals of the metal bound C atoms of the formal allene unit are observed at 164.7 (2a) and 173.2 ppm (2b), whereas the internal C atoms resonate at higher field (2a: 151.4, 2b: 147.0 ppm). Compared to the previously described Ti complex A, the metal bound C atoms of the two Zr complexes 2a and 2b resonate at much higher field (Ti–*C* 213.8 ppm), while the signals of internal C atoms were found at lower field than for A (C=*C*=C 134.2 ppm), indicating significant differences in the electronic structures.

**Fig. 2:**
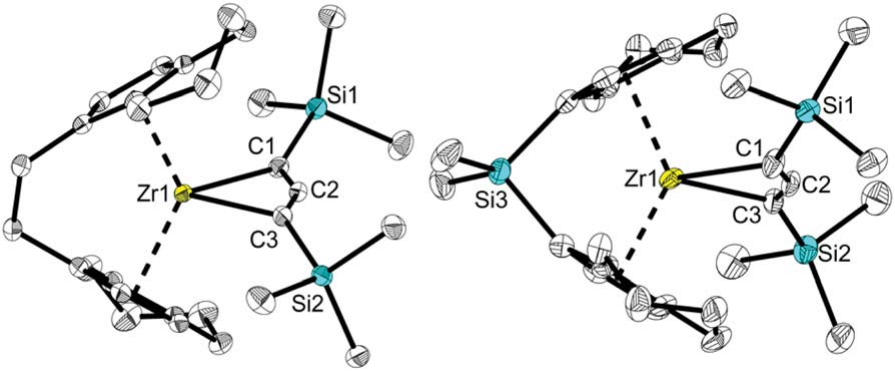
Molecular structure of complexes 2a (left) and 2b (right). Thermal ellipsoids correspond to 30% probability. Hydrogen atoms and the second position of the disordered tetrahydroindenyl group are omitted for clarity.

X-ray analysis of single crystals of complexes 2a and 2b that were obtained by storing the concentrated pentane solution at −30 °C (Fig. 2) shows the corresponding Zr centre in distorted tetrahedral coordination geometry with the bridged cyclopentadienyl ligand and the allenediide ligand. Based on the experimental bond parameters, these complexes are best described as a Zr(iv) species with a covalently bound dianionic allenediide ligand (2a: Zr1–C1 2.3099(12), Zr1–C3 2.3074(12), C1–C2 1.3100(18), C2–C3 1.3076(18) Å, C1–C2–C3 149.32(12)°; 2b: Zr1–C1 2.342(4), Zr1–C3 2.319(4), C1–C2 1.302(6), C2–C3 1.290(5) Å, C1–C2–C3 150.5(4)°; Σ*r*_cov,Zr–C_ = 2.29, Σ*r*_cov,C=C_ = 1.34 Å ^24^). Notably, while the latter values are identical in the Ti system, metal-carbon bonds are well in line with Zr–C single bonds in this case, whereas for A, much longer Ti–C distances were observed. This could be explained by the well-known greater bond strength of metal–ligand bonds for 4d compared to 3d metal systems and could point to pronounced differences in stability and reactivity (*vide infra*).

### Intramolecular aliphatic C–H bond activation

**Scheme 2:**
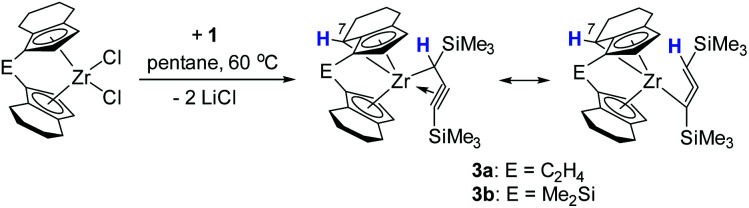
Synthesis of C–H bond activation products 3a and 3b.

Interestingly, in solution, complex 2a undergoes a selective intramolecular C–H bond activation of the CH_2_ group in the 7-position of the 4,5,6,7-tetrahydroindenyl moiety to furnish a propargyl complex 3a (Scheme 2). The nature of the thus formed metallocene fragment is strongly reminiscent of so-called “tucked-in” complexes that are commonly observed for Cp* ligands.^25^ Notably, this mode of ligand activation has not been observed to date for this type of *ansa*-cyclopentadienyl ligands. This process occurs slowly at room temperature. To facilitate this transformation, we increased the temperature to 60 °C, and found that the reaction of [*rac*-(ebthi)ZrCl_2_] with the dilithiated allene 1 in pentane or benzene generates this Zr propargyl complex 3a with full conversion after four days. The colour of the reaction solution turned from greenish to brown at last. A similar dimethylsilyl bridged C–H bond activation product 3b can be obtained from 2b, albeit in much less reaction time of only one day.

The ^1^H NMR spectra show five doublet resonances at *δ* 5.75, 5.72, 5.48, 5.43 and 4.23 ppm for 3a, and *δ* 5.86, 5.74, 5.62, 5.35 and 3.79 ppm for 3b, corresponding to the CH protons of cyclopentadienyl and fused cyclohexyl groups of the metallocene moiety. The ^13^C NMR spectra show three characteristic signals which are assigned to the terminal (C≡*C*–SiMe_3_, 3a: 141.9, 3b: 139.3 ppm), internal (*C*≡C–SiMe_3_, 3a: 96.2, 3b: 96.3 ppm) and metal bound carbon atoms (Zr–*C*, 3a: 51.5 ppm, 3b: 51.4 ppm) of the propargyl unit.

**Fig. 3:**
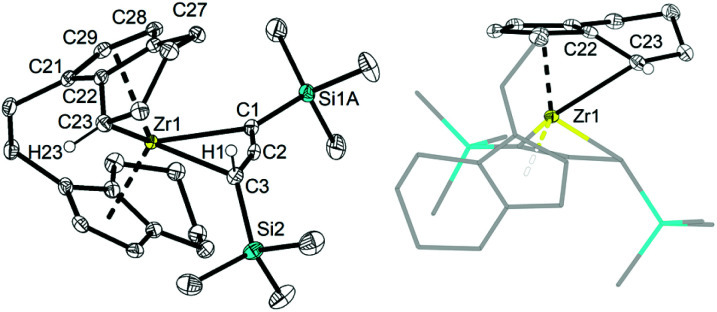
Left: molecular structure of complex 3a. Right: alternative view of complex 3a, illustrating the presence of a covalent bond between Zr1 and C–H activated C23. Thermal ellipsoids correspond to 30% probability. Hydrogen atoms (except H1 and H23), solvent molecule and the second position of the disordered tetrahydroindenyl and SiMe_3_ group are omitted for clarity. Selected bond lengths and angles for 3a: C1–C2 1.262(3), C2–C3 1.364(3), C21–C29 1.419(3), C21–C22 1.439(3), C22–C27 1.439(2), C27–C28 1.403(3), C28–C29 1.409(3), C22–C23 1.420(3), C1–Zr1 2.4407(18), C2–Zr1 2.3924(17), C3–Zr1 2.5747(18) Å; Zr1–C1–C2 72.76(11), C1–C2–C3 158.56(18), C2–C3–Zr1 66.81(10), C1–Zr1–C3 61.86(6)°, Σ(∠C22) = 350°.

**Fig. 4:**
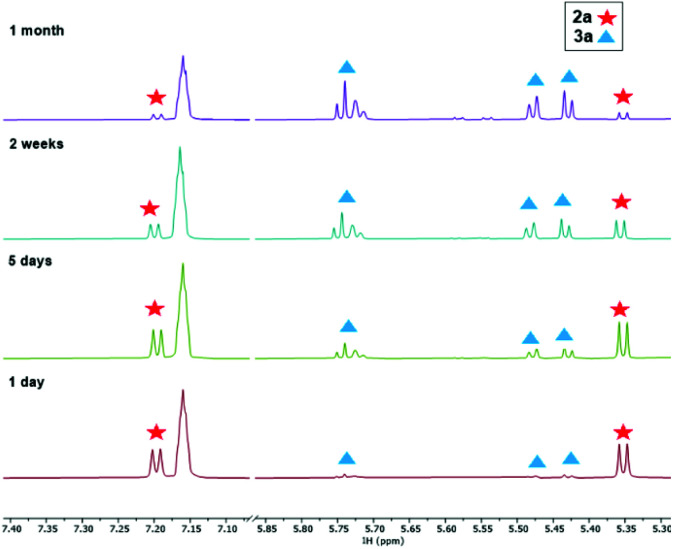
^1^H NMR spectroscopic monitoring of the transformation of 2a to 3a (25 °C, benzene-*d*_6_, 300.2 MHz, low-field region, *δ* 7.07–5.87 ppm are omitted for clarity).

Single crystals of these unusual species 3a and 3b could be obtained from concentrated benzene solution at room temperature. The molecular structure of complex 3a^26^ (Fig. 3, left) reveals the presence of a Zr propargyl complex as a four-membered ring system. Early transition metal complexes with CH_2_C≡CR units are known as the combination of η^3^-propargyl and η^3^-allenyl resonance structures.^27^ In the herein reported CH(SiMe_3_)C≡CR structure, C1–C2 and C2–C3 bond lengths correspond to a triple and double bond, respectively, and the C_3_ ligand unit is thus best described as a resonance form between η^3^-propargyl and allenyl structures. The Zr–C1–C2–C3 unit is planar (−1.5(5)°) and this is also in agreement with the planarity of such η^3^-propargyl/allenyl complexes. Contacts to the activated fragment of the former *rac*-(ebthi) ligand are 2.141 (Zr-Cp′_centroid_) and 2.5703(18) Å (Zr1–C23). Although the latter value is considerably larger than in Bouwkamp's [Cp*(η^6^-C_5_Me_4_CH_2_)Zr(thf)]^+^ (B) (2.366(4) Å)^28^ and Marks' [Cp*(η^6^-C_5_Me_4_CH_2_)ZrPh] (C) (2.388(7) Å),^29^ the deviation from planarity at C22 (Fig. 3, right; Σ∠(C22; 3a) = 350°; Σ∠(C115; B) = 346°; Σ∠(C1; C) 346°) clearly indicates the presence of a η^5^,η^1^ (or η^6^)-bound fragment. Taken together, one C23–H bond is intramolecularly activated and the proton is transferred to the C_3_ ligand, resulting in an unusual formally trianionic, bridged tucked-in metallocene structure (Fig. 3, right) that possesses a η^3^-propargyl/allenyl unit coordinated to the Zr centre. As mentioned above, slow transformation of 1-metallacyclobuta-2,3-dienes 2a and 2b in solution yielded C–H bond activation products 3a and 3b with high conversion (3a: 88%, 3b: 98%) after weeks at room temperature (Fig. 4). However, 10% of residual 2a was obtained from the solution of 3a after one month, which is not the case for 3b (Fig. S19†). In addition, the mutual interconversion between these two species 2 and 3 at room temperature can explain why pure NMR spectra of compounds 2a, 2b, and 3a are generally not possible to obtain. A similar, fast and selective C–H activation reaction was not observed using Ti complex A, however, slow conversion into a hitherto unidentified species takes place at 60 °C (Fig. S21†).

**Fig. 5:**
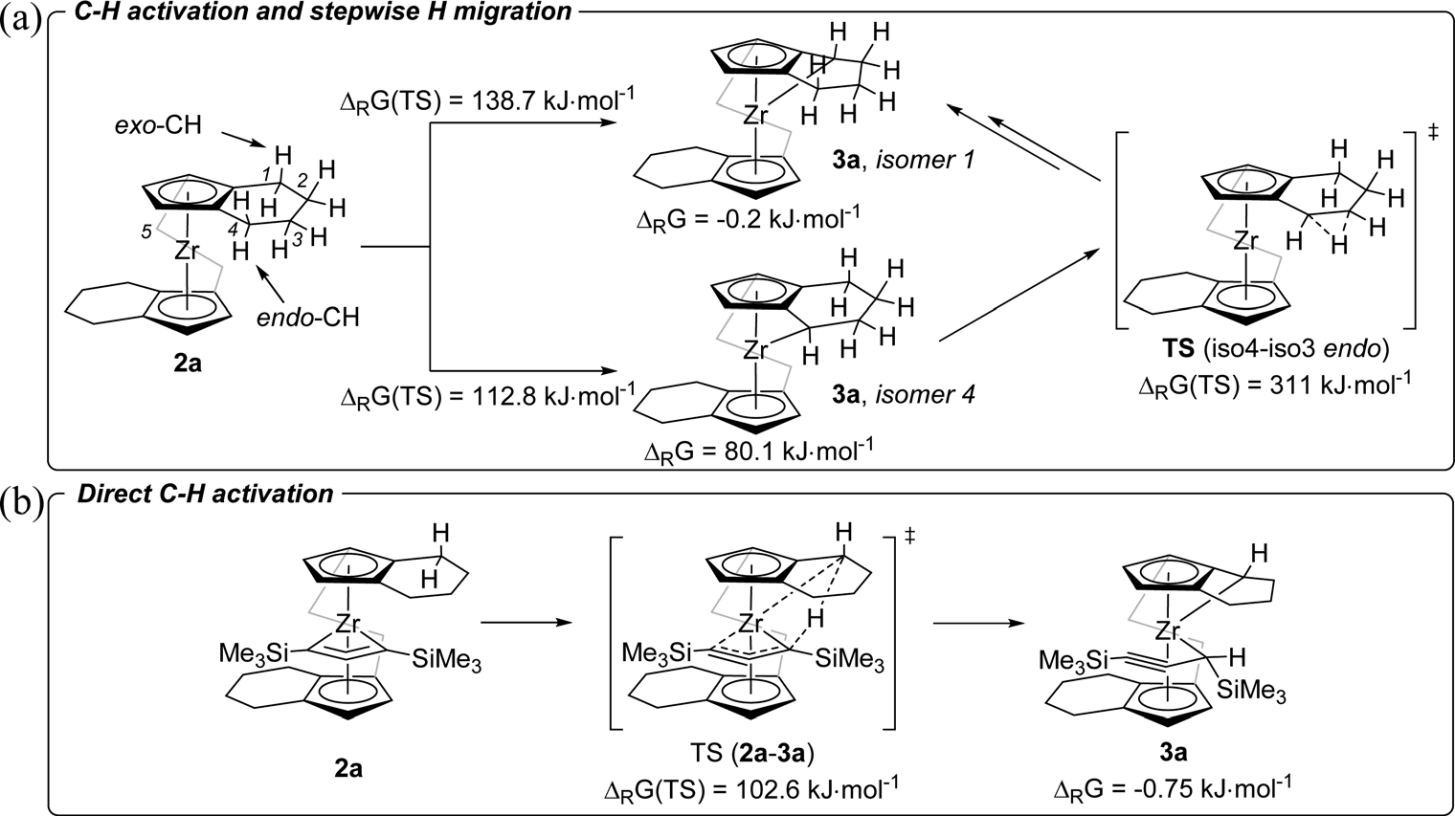
(a) Hypothetical formation of 3a from 2a*via* C–H activation and stepwise H migration. For clarity, complexes 2a and 3a are shown without the C_3_ ligand (B3LYP/GD3BJ/def2svpp). (b) Direct C–H activation to produce 3a (B3LYP/GD3BJ/def2tzvp).^30^

To obtain further insights into this unusual C–H activation sequence, we have analysed this process for the system 2a/3a computationally using a stepwise approach where we first identified an appropriate reaction path using a smaller double zeta basis set, followed by using a more sophisticated triple zeta basis set. All geometries were optimised and were confirmed to be local minima or first order saddle points (for transition states, TS) on the potential energy surface by harmonic vibration frequency calculation on the same level of theory (B3LYP^31^/GD3BJ^32^/(def2svpp)def2tzvp^33^). We were intrigued by the selective formation of complexes 3a and 3b where only one CH_2_ group of the tetrahydroindenyl fragment is activated and a proton is shifted to the metal coordinated C_3_ unit. Therefore, we first calculated the Gibbs free energies for the activation of all five CH_2_ groups present in the *rac*-(ebthi) ligand as well as their TS (Fig. 5). These calculations nicely show that only the formation of the experimentally found isomer 1 is exergonic (Δ_R_*G* = −0.2 kJ mol^−1^). However, the TS to produce isomer 4 (Δ_R_*G* = 112.8 kJ mol^−1^) is significantly lower in energy than that for isomer 1 (Δ_R_*G* = 138.7 kJ mol^−1^). We have thus next analysed two possible paths of subsequent H migration from isomer 4 to isomer 1 and found that neither the migration of the outer (*exo*) CH protons nor that of the protons facing the metal (*endo*) show TS which would support this concept (all TS >250 kJ mol^−1^, Fig. 5a and Table S6†). As consequence, direct C–H activation was evaluated using the larger basis set def2tzvp (Fig. 5b and Table S7†). These calculations confirm isomer 1 as the thermodynamically preferred product of the reaction. Interestingly, the TS for its formation (Δ_R_*G* = 102.6 kJ mol^−1^) now also is lowest in energy and even allows a C–H activation reaction at room temperature. This nicely confirms the experimentally observed formation of complex 3a from 2a within days (Fig. 4). The minor calculated energy difference between 2a and 3a of only −0.75 kJ mol^−1^ suggests the feasibility of the inverse reaction in which 2a is formed from 3a. The equilibrium composition at room temperature estimated using the Bolzmann distribution theorem is 42/58% (2a/3a). In line with this, ^1^H NMR monitoring of solutions of complex 3a over one month shows slow, but constant conversion to produce the 1-metallacyclobuta-2,3-diene 2a (Fig. S16†).

### Computational analysis of structure and bonding in complexes 2 and 3

**Fig. 6:**
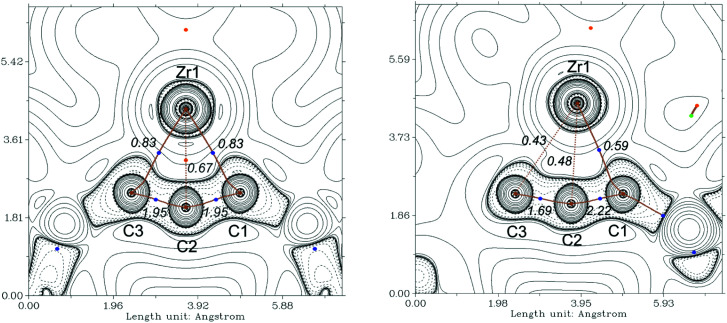
Contour plots of the Laplacians of the electron density ∇^2^*r* of complexes 2a (left) and 3a (right) in the C–Zr–C plane. Dashed lines indicate negative (local charge concentration), solid lines indicate positive values (local charge depletion). The Laplacian plot is overlaid with the molecular graph from QT-AIM analysis and Wiberg bond indices (italic small numbers). Brown lines indicate bond paths, brown dashed lines are hyphothetical bonds, blue dots correspond to bond critical points, light brown dots indicate ring critical points. Density from B3LYP/GD3BJ/def2tzvp.

As mentioned above, the Ti compound A can be described as an unusual antiferromagnetically coupled biradicaloid system, possessing a formal Ti(iii) centre coordinated with a monoanionic alleneylide ligand. In that case, Complete Active Space (CAS(8,9)) SCF calculations, determined a biradical character of *β* = 28%. To compare the bonding situation of the Zr analog 2a we first evaluated the stability of the Kohn–Sham wavefunction from B3LYP calculation and found that this is stable. However, the Hartree–Fock wavefunction shows an RHF/UHF instability for 2a, the same was observed in A, but not for complex 3a. Therefore, we investigated the electronic situation of 2a as an open-shell singlet considering similar CAS molecular orbitals (MOs) as for A (Fig. S121†). This calculation reveals a negligible occupation number in the formal LUMO (*ϕ*_5_) orbital of only 0.08 electrons. Even though we also found a lower biradicaloid character in a previous study of zirconocene phosphinidenes compared to its titanocene analogs, this finding was surprising.^34^ Based on this result we neglect the biradicaloid character of 2a and 2b. We next evaluated the contour plots of the Laplacians of the electron density ∇^2^*r* of the investigated complexes and overlaid these with the results from the quantum theory of atom in molecules (QT-AIM) analysis^35^ and their Wiberg bond indices (WBIs, given in italics) (Fig. 6 and S112–S114†). The QT-AIM analysis of related group 4 1-metallacyclobuta-2,3-diene complexes 2a, 2b, and A revealed two M–C “bond” paths in the metallacycles in between the metal centre and the α-carbon atoms, respectively (2a: Fig. 6 left; 2b: Fig. S113;†A: Fig. S114†). Ring critical points were located between the metal centre and the β-carbon atom, thus indicating the absence of a bonding interaction between the central carbon atom and the metal centre. The lower WBI between the central carbon atom and the metal centre compared to the α-C–M bonds nicely supports the findings of the QT-AIM analysis. Furthermore, the value of 1.95 for the C–C bonds in the allene units in 2a and 2b clearly reveals these as double bonds based on this theory (*cf.* 1.93 in A). For η^3^-propargyl/allenyl complexes 3a and 3b (3a: Fig. 6 right; 3b: Fig. S116†) the QT-AIM analysis shows only one “bond” path between the Me_3_SiCCCHSiMe_3_ unit and the Zr centre. The WBI of these bonds are lower than 0.6 but these values are larger than those of the other M–C interactions, which supports the QT-AIM analysis. In line with the description as η^3^-propargyl/allenyl complexes two different WBI could be determined along the C_3_ unit (1.7 and 2.2) in both complexes. Furthermore, the additional analysis of the natural bond orbitals (NBO)^36^ and the investigation of the natural localised molecular orbitals (NLMO) of complexes 2a, 2b and 3a confirm the previous results (see ESI,† section 8.3.1). The analysis of the NLMOs reveals small contributions (7.4–2.3%) of a d orbital at Zr for the allene CC π-type orbitals. These are absent for the corresponding CC σ-type orbitals. This additional interaction could contribute to the stabilisation of the four-membered ring systems studied here. Since the Laplacian plots indicate a polarised α-C–M bond, we finally summed the natural charges (NBO) of all atoms in the Me_3_SiCCCSiMe_3_ fragments which shows significantly larger values for the Zr complexes 2a and 2b compared to its lighter congener A (2a: −0.98; 2b: −0.95; A: −0.74). This points to a higher polarity of the M–C interaction in the zirconacycles 2a and 2b and is well in line with the greater biradicaloid character of the Ti complex A. This difference should affect the reactivity of the here investigated Zr complexes compared to that of A.

### Reactivity of 1-metallacyclobuta-2,3-dienes 2a/2b and propargyl/allenyl complexes 3a/3b

Before investigating the reactivity of complexes of types 2 and 3, we first evaluated their stability upon exposure to air or water. Unsurprisingly, in both cases, formation of the well-known propyne Me_3_SiC≡CCH_2_SiMe_3_ was observed^14,37^ which is in line with observations made for Ti complex A (Fig. S14, S15, S17 and S18†).

**Scheme 3:**
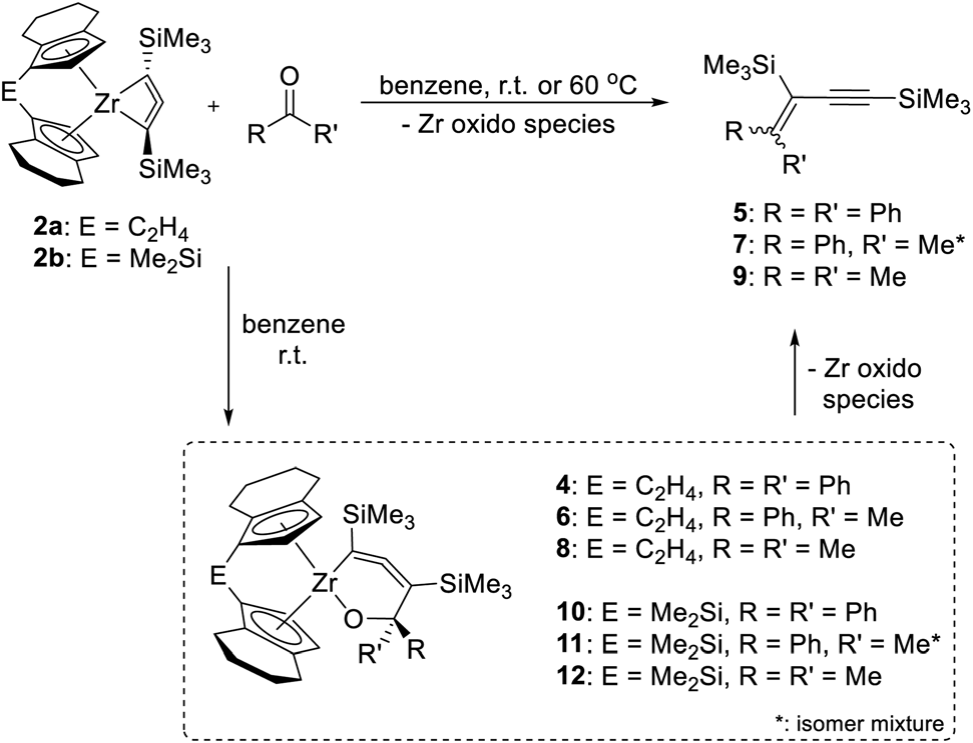
Reaction of complexes 2a and 2b with ketones to yield enynes 5, 7 and 9. Note: compound 4 contains compound 13 (*vide infra*) as an impurity as 2a, used for its synthesis always contains traces of 3a (Fig. S43†).

As mentioned above, the Ti complex A shows well-defined reactivity with carbonyl compounds, producing enynes^38^ by coupling of the allenediide fragment with the methylene unit of the substrate and oxygen transfer to the Ti centre. In general, reactions of unsaturated substrates with C = X (X = heteroatom) moieties are well-studied for a variety of group 4 metallacycles and 1,2- or 2,1-insertions are commonly observed.^39^ Reactions of complexes 2a and 2b with benzophenone, acetophenone and acetone showed similar reactivities as A to furnish corresponding enynes 5, 7, and 9 as the final product (Scheme 3).

**Fig. 7:**
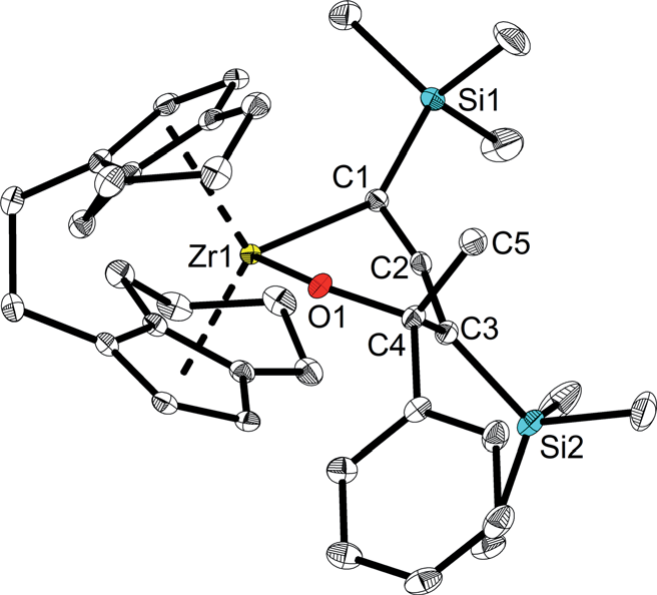
Molecular structure of complex 6. Thermal ellipsoids correspond to 30% probability. Hydrogen atoms are omitted for clarity.

However, unlike A which shows full conversion within 16 hours at room temperature, Zr complexes of type 2 required longer time at room temperature or harsher reaction conditions to produce enynes (see ESI† for details). It should be noted that after adding ketones into the solution of complex 2a at room temperature, the colour of the solution changed from green to orange immediately. To our delight, single crystals of complexes 4 and 6 could be obtained from *n*-hexane and confirmed the assignment as a six-membered ring system, formed by insertion of ketone into the Zr–C bond of the 1-metallacyclobutadi-2,3-ene.^40^ In compound 6 (Fig. 7), C1–C2 (1.2951(19) Å) is shorter than C2–C3 (1.3398(19) Å), however, both distances correspond to double bonds. The Zr1–C1 distance of 2.3172(13) Å is slightly longer than typical single bonds, while the Zr1–C2 and Zr1–C3 distances are 2.5083(13) Å and 3.0433(14) Å, respectively, as the result of the ring enlargement. Release of ring strain, compared to 2a, thus leads to substantial linearisation of the allene unit (C1–C2–C3 166.5(2)°). The Zr1–O1 distance of 2.0362(9) Å indicates the presence of a shortened Zr–O single bond (Σ*r*_cov,Zr–O_ = 2.17 Å ^24^) that is slightly longer than found in related complexes formed by insertion of carbonyl compounds.^41^

Without workup, the orange residue of 8 was analysed by NMR spectroscopy. The ^1^H NMR spectrum showed four doublet resonances at *δ* 6.68, 6.43, 5.43 and 5.18 ppm, corresponding to the Cp protons of a new metallacyclic species, formed by a similar insertion of acetone.

The reactions of related 2b with ketones were performed in benzene-*d*_6_ in Young-NMR tubes. Corresponding intermediates (10, 11 or 12) and the same final enynes (5, 7 or 9) could be clearly identified by ^1^H NMR spectroscopy without further workup. These observations are well in line with the calculated Gibbs free energies of this reaction sequence, which indicate that formation of the six-membered ring systems is exergonic in all cases (range: Δ_R_*G* = −101.32 (6); −114.52 (10) kJ mol^−1^). The subsequent formation of the enynes 5, 7, and 9 as the final products is endergonic with respect to these insertion products, but still overall exergonic (Tables S8 and S9†). Similar intermediates of reactions of A were calculated to be endergonic, which explains why we could isolate these insertion products only for the herein described Zr systems.

Reactions of ketones with five-membered all-C-metallacycloallenes were investigated before, however, isolation of the organometallic species, formed by 1,2-insertion was not reported.^4*e*^ Formation of heterometallacycles, either by insertion into the M–C bond or through cycloaddition, followed by redox-neutral^42^ or reductive cleavage^43^ of the newly formed metallacycles, is common for group 4 complexes and its utility for organic synthesis was demonstrated on various occasions.

**Scheme 4:**
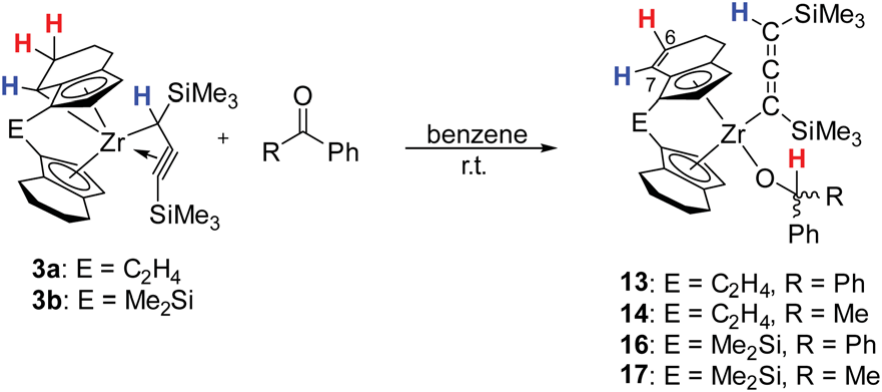
Reaction of 3 with benzophenone/acetophenone to yield 13, 14, 16 and 17.

Tucked-in complexes show a rich organometallic chemistry that is dominated by insertion reactions into the metal–carbon bond.^44^ The reactivity of complexes 3a and 3b was investigated with benzophenone or acetophenone at room temperature. After one day a new Zr(iv) complex was obtained which contains an alcoholate group covalently bound to Zr and possesses a η^5^-4,5-dihydroindenyl fragment, *i.e.* a doubly C–H activated six-membered ring of a former *rac*-(ebthi) ligand (Scheme 4). Notably, no organic products were detected after four days at 80 °C. The ^1^H NMR spectrum of complex 14 as an example shows four doublet resonances (*δ* 6.47, 5.53, 5.44 and 5.08 ppm), one quartet resonance (*δ* 5.28 ppm) and one singlet resonance (*δ* 3.30 ppm), which are consistent with the presence of Cp, alcoholate and allene groups. Besides, the two protons at 6- and 7-position of the former indenyl ring were found at 6.41 and 5.69 ppm, which was confirmed by ^1^H,^1^H COSY and NOESY experiments. In ^13^C NMR spectra, three characteristic signals are assigned to the internal C atom (195.5 ppm), metal-bound (101.1 ppm) and terminal C atom (51.9 ppm) of the C_3_ unit, whereas the signal for the O bound C atom is observed at 81.8 ppm.

**Fig. 8:**
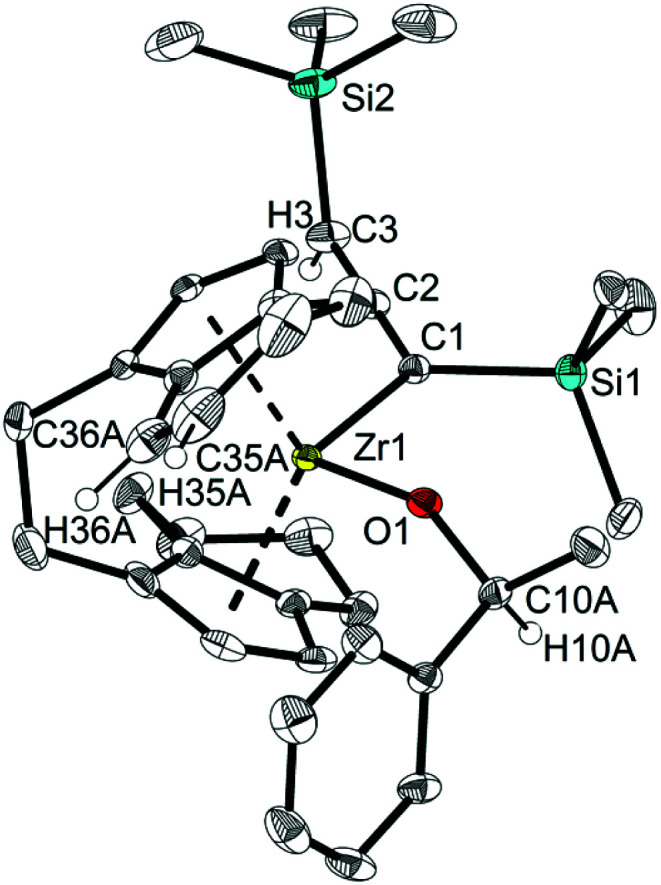
Molecular structure of complex 14. Thermal ellipsoids correspond to 30% probability. Hydrogen atoms (except H3, H10A, H35A and H36A) and the second position of the disordered group are omitted for clarity.

Single crystals of 13 and 14 obtained from *n*-hexane unequivocally clarified the above-made structural assignment.^40^ The molecular structure of complex 14 (Fig. 8) shows the bent metallocene coordinated with a covalently bound alcoholate ligand and monoanionic allenyl ligand. In line with an η^1^-allenyl/propargyl resonance C1–C2 (1.2924(17) Å) is slightly shorter than C2–C3(1.3257(18) Å) and both bond lengths are in the range of shortened double bonds. The angle C1–C2–C3 is 179.29(14)°, which shows the linear arrangement of allene. Although the molecular structure of complex 14 shows disorder, the C35A-C36A distance (1.336(4) Å) in the six-membered ring of the former *rac*-(ebthi) ligand is consistent with typical C=C bond.^45^

While *rac*-(ebthi), *rac*-(ebi) (ebi = 1,2-ethylene-1,1′-bis(η^5^-indenyl)) and related η^5^-indenyl complexes of group 4 metals are frequently used,^46^ especially in polyolefin chemistry,^47^ examples for well-defined complexes possessing 4,5-dihydroindenyl moieties as part of the metallocene fragment are elusive and to the best of our knowledge were not isolated and characterised before. Such species can be regarded as intermediates for industrially relevant hydrogenation of [*rac*-(ebi)ZrCl_2_] to produce [*rac*-(ebthi)ZrCl_2_].^48^ Furthermore, complexes 13, 14, 16, and 17 represent rare examples for stable η^1^-allenyl complexes as such species tend to be in equilibrium with η^1^-propargyl complexes.^27*g*^ We would further like to mention that these complexes result from a formal hydride transfer from the formally trianionic tucked-in ligands to the ketone substrate.

**Scheme 5:**
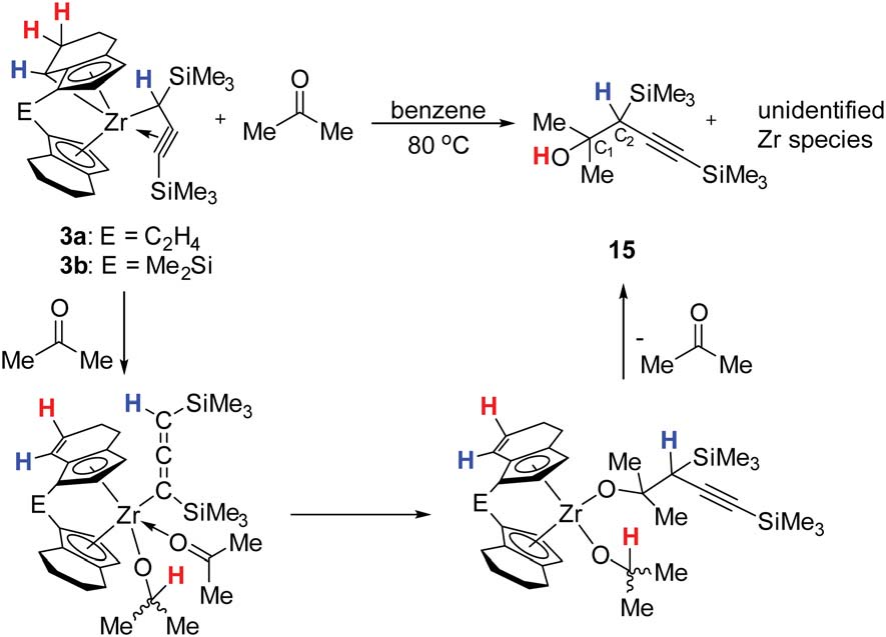
Reaction of 3 with acetone to yield 15 and postulated mechanism for the insertion of acetone and product formation.

Interestingly, complexes 3a and 3b showed a different reactivity in the reaction with acetone. When performing the reaction at room temperature, no desired organometallic product was identified by NMR spectroscopy. While monitoring the reaction with two equivalents of acetone at 80 °C, we observed the formation of a major product (Scheme 5) resulting from the insertion of the C=O bond of acetone into the Zr–C bond of the activated C_3_ ligand.

The ^1^H NMR spectrum of the organic product that was obtained after purification by column chromatography shows two singlet resonances at *δ* 1.52 (broad) and 1.97 ppm, corresponding to protons of a hydroxyl group and a methine group. The ^13^C NMR spectrum showed two resonances due to the alkynyl group at 108.4 and 88.2 ppm, while the signals at 72.5, 0.4 and −0.5 ppm suggested the presence of proton-free C atom C_1_ and SiMe_3_ groups. The IR spectrum shows a band at *ν* = 3463 cm^−1^ for the OH group (Fig. S107†). MS analysis shows fragments at *m*/*z* 186 [M–CMe_2_OH^+^], 152 [M–OH–TMS^+^], 147 [M–C=CTMS^+^] and 137 [M–OH–Me–TMS^+^] that supports the assignment as an alcohol containing an alkynyl group (15). Based on literature precedent, we postulate that compound 15 forms *via* a η^1^-allenyl complex that is similar to those shown in Scheme 4. Interaction of ketone with the metal centre of the allenyl complex, followed by insertion of the ketone into the Zr–C bond and rearrangement could produce a bis(alkoxide) species. Intramolecular protonation would result in the formation of product 15 (Scheme 5). Related reactivity was described for a titanocene system.^49^

### Consideration of selectivity determining factors: formation of 1-zirconacyclobuta-2,3-diene *vs.* dinuclear complex

**Scheme 6:**
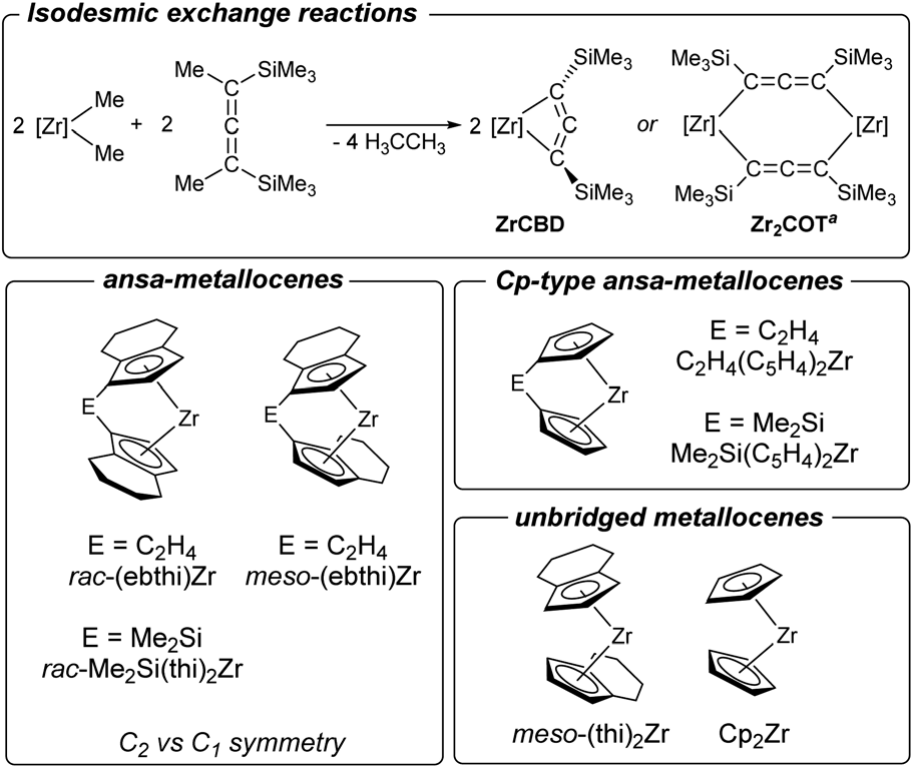
Top: computed isodesmic exchange reactions to evaluate the thermodynamics of formation of dinuclear complexes *vs.* that of 1-zirconacyclobuta-2,3-diene. Bottom: metallocene fragments used for the above calculations. ^*a*^Formation of Zr_2_COT is known to occur *via* the dinuclear allenediide bridged chloride complex [Zr](Cl)(Me_3_SiC_3_SiMe_3_)[Zr](Cl) (Zr_2_Cl_2_) (*cf.*Fig. 1a).

The herein described formation of complexes 2a and 2b contrasts with previous observations for unsubstituted and unbridged zirconocenes where formation of dinuclear dizirconacyclooctatetraene complexes took place exclusively.^14^ To rationalise these differences, we have therefore computed the thermodynamic data of putative four-membered metallacyclic (ZrCBD) and dinuclear complexes (Zr_2_COT) using a set of related cyclopentadienyl ligands (Scheme 6 and Table 1).

**Table 1:** Computed Gibbs free energies and reaction enthalpies (in kJ mol^−1^) for hypothetical 1-zirconacyclobuta-2,3-dienes (ZrCBD) and dinuclear complexes (Zr_2_COT). B3LYP/GD3BJ/def2svpp

Entry	[Zr]	2 × Δ*H*(ZrCBD)	2 × Δ*G*(ZrCBD)	Δ*H*(Zr_2_COT)	Δ*G*(Zr_2_COT)	ΔΔ*H*^a^	ΔΔ*G*^a^
1	*rac*-(ebthi)Zr	−53.22	−106.96	−137.42	−90.92	−84.2	16.04
2	*rac*-Me_2_Si(thi)_2_Zr	−50.01	−99.17	−158.48	−117.81	−108.47	−18.64
3	*meso*-(ebthi)Zr	25.77	−25.95	−148.08	−115.35	−173.85	−89.40
4	C_2_H_4_(C_5_H_4_)_2_Zr	−0.27	−48.95	−295.43	−260.22	−295.16	−211.27
5	Me_2_Si(C_5_H_4_)_2_Zr	7.59	−45.86	−291.19	−253.85	−298.78	−208.00
6	Cp_2_Zr	13.03	−30.30	−277.63	−239.16	−290.66	−208.86
7	*meso*-(thi)_2_Zr	34.06	−19.62	−188.37	−149.15	−222.43	−129.52

a ΔΔ*G* = Δ*G*(Zr_2_COT) − (2 × Δ*G*(ZrCBD)). ΔΔ*H* = Δ*H*(Zr_2_COT) − (2 × Δ*H*(ZrCBD)).

For all zirconocenes considered, both reaction channels are highly exergonic. However, the difference in Gibbs free energies ΔΔ*G* shows that formation of the corresponding four-membered ZrCBD complex (2a) is only thermodynamically preferred (ΔΔ*G* = 16.04 kJ mol^−1^) for the *rac*-(ebthi) ligand. For the *rac*-Me_2_Si(thi)_2_Zr system, which was also investigated experimentally, this value is slightly exergonic, indicating kinetic stabilisation of complex 2b (ΔΔ*G* = −18.64 kJ mol^−1^). Only for these two species clearly exothermic reaction enthalpies were calculated (Δ*H* = −26.61 (2a); −25.00 (2b) kJ mol^−1^). In consequence, formation of binuclear ZrCOT complexes should be strongly preferred for all other cases. Furthermore, Cp-based systems, whether bridged or not, should form ZrCOT complexes much more preferentially (Table 1). The presence of an indenyl unit, however, appears to impede the formation of dinuclear complexes. Reactions of the parent unsubstituted cyclopentadienyl systems were reported by us before for M = Zr, Hf and selectively gave dinuclear allenediide bridged zirconocene and hafnocene complexes,^14^ in line with the strong thermodynamic preference of these species (Table 1, entry 6).

To further support these assumptions, we have next performed NMR experiments using further zirconocene complexes shown in Scheme 6. In the reaction of non-bridged [(thi)_2_ZrCl_2_] (thi = η^5^-tetrahydroindenyl) (Table 1, entry 7) with an equimolar amount of 1 formation of mixtures of Zr_2_COT and its dinuclear chloride precursor Zr_2_Cl_2_ is evident, as indicated by the presence of two sets of ^1^H NMR signals for the cyclopentadienyl and SiMe_3_ protons (Fig. S79–S84†). Similarly, NMR analysis of the reaction of C_1_ symmetric *meso*-(ebthi)ZrCl_2_ (Table 1, entry 3) with 1 shows resonances that indicate the formation of singly and double allenediide bridged dinuclear complexes Zr_2_Cl_2_ and Zr_2_COT (Fig. S85–S87†). From this mixture, single crystals could be obtained, and an X-ray analysis confirms these as the respective dinuclear Zr chloride complex (Fig. S25†). Finally, the reaction of the Cp type *ansa*-metallocene [Me_2_Si(C_5_H_4_)_2_ZrCl_2_] (Table 1, entry 5) with 1 resulted in the formation of the Zr_2_Cl_2_ complex, which could be confirmed by ^1^H NMR spectroscopy and an X-ray analysis (Fig. S26†). Based on these model studies and the consideration of the thermodynamics of these salt metathesis reactions, we thus conclude that both, the presence of a bridging unit and C_2_ symmetry of the metallocene halide are essential for the formation of 1-zirconacyclobuta-2,3-dienes. While the former prevents the rotation of the cyclopentadienyl ligands, the latter factor, by minimizing steric strain, forces the Me_3_Si groups into *trans* position of the desired four-membered metallacycle.

## Conclusions

We have presented the synthesis of two 1-zirconacyclobuta-2,3-dienes, organometallic analogs of the elusive 1,2-cyclobutadiene. Both complexes can be prepared by salt metathesis using an *ansa*-zirconocene dichloride and a 1,3-dilithiated allene precursor. Computational analysis of the structure and bonding in these complexes shows that in contrast to the previously reported Ti analog the biradical character is neglectable. Instead, the Zr complexes are best described as Zr(iv) species that possess a dianionic allenediide ligand. Both, C_2_H_4_ and Me_2_Si bridged metallacycles undergo selective thermal C–H activation at the 7-position of the tetrahydroindenyl fragment to produce a new type of “tucked-in” metallocene complex. This activation mode is known in metallocene chemistry but was previously not reported for well-established *ansa*-cyclopentadienyl ligands. Based on DFT analysis we propose a direct C–H activation *via* deprotonation as the most likely mechanism for this process.

Reactions of 1-zirconacyclobuta-2,3-dienes with ketones occur *via* the formation of six-membered oxa-zirconacycles. Other than reported before for the Ti system, these insertion products can be isolated and only produce the enyne coupling products after longer reaction times or upon heating. Reactions of the tucked-in η^3^-propargyl/allenyl complexes with ketones furnish η^1^-allenyl complexes in which further C–H activation at the metallocene results in the formation of a hitherto unknown 4,5-dihydroindenyl ligand.

The formation of 1-zirconacyclobuta-2,3-dienes described herein contrasts with previous observations made for the parent Cp_2_Zr system where open, dinuclear, allenediide bridged complexes are formed selectively. Computational analysis of model reactions indicates a thermodynamic preference for the formation of four-membered metallacycles for the herein experimentally studied C_2_ symmetric tetrahydroindenyl *ansa*-cyclopentadienyl systems. This assumption was confirmed experimentally using selected model systems. In summary, these data help to rationalise the differences in selectivity and will guide further studies directed at the synthesis and reactivity of these and related unusual metallacycles.

## Author contributions

X. S., F. R. and T. B. conceived and conceptualised the project. X. S., S. L., M. R. and A. S. performed the experiments and analysed the data. F. R. performed DFT calculations and analysed the data. T. H.-R. provided resources used in this study. T. B. supervised the project and acquired funding. X. S., F. R. and T. B. prepared and revised the manuscript.

## Conflicts of interest

There are no conflicts to declare.

## Supplementary Material

SC-012-D1SC06052J-s001

SC-012-D1SC06052J-s002

SC-012-D1SC06052J-s003
