# Supplementary material for: 1-Zirconacyclobuta-2,3-dienes: synthesis of organometallic analogs of elusive 1,2-cyclobutadiene, unprecedented intramolecular C–H activation, and reactivity studies
Source: Chem Sci. 2021 Nov 23;12(48):16074–84. doi: 10.1039/d1sc06052j (PMC8672727; doi:10.1039/d1sc06052j)
Supplement: SC-012-D1SC06052J-s002 [file SC-012-D1SC06052J-s002.pdf]

Electronic Supplementary Material (ESI) for Chemical Science.
This journal is © The Royal Society of Chemistry 2021

SUPPORTING INFORMATION

1-Zirconacyclobuta-2,3-dienes: Synthesis of organometallic analogs of elusive 1,2-cyclobutadiene, unprecedented intramolecular C-H activation, and reactivity studies

Xinzhe Shi,[a] Sihan Li,[a] Melanie Reiß,[a] Anke Spannenberg,[a] Thorsten Holtrichter-Rößmann,[b] Fabian Reiß*[a] and Torsten Beweries*[a]

[a] Leibniz-Institut für Katalyse e.V. an der Universität Rostock, Albert-Einstein-Str. 29a, 18059 Rostock, Germany, Email: fabian.reiss@catalysis.de, torsten.beweries@catalysis.de.

[b] LANXESS Organometallics GmbH, Ernst-Schering-Str. 14, 59192 Bergkamen, Germany.

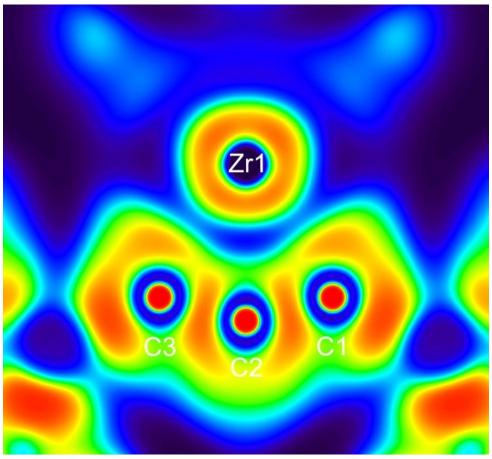

Table of Contents

1. Experimental Details 2

2. Synthesis of Compounds 4

3. Reactivity of Complexes 11

4. Proof of Stability of Compounds 24

5. Crystallographic Details 30

6. Details of NMR Spectroscopy 35

7. Vibrational Spectra 68

8. Computational Details 76

9. References 112

1. Experimental Details

1.1 General Information

All manipulations were carried out in an oxygen- and moisture-free argon atmosphere using standard Schlenk and drybox techniques. The solvents were purified with the Grubbs-type column system "Pure Solv MD-5" and dispensed into thick-walled glass Schlenk bombs equipped with Young-type Teflon valve stopcocks. *rac*-ethylenebis(4,5,6,7-tetrahydro-1-indenyl)zirconium(IV) dichloride ([*rac*-(ebthi)$ZrCl_2$], MCAT), *rac*-dimethylsilylenebis(4,5,6,7-tetrahydro-1-indenyl)zirconium(IV) dichloride ([*rac*-$Me_2Si(thi)_2ZrCl_2$], LANXESS), bis(tetrahydroindenyl)zirconium(IV) dichloride ([$(thi)_2ZrCl_2$], MCAT), *meso*-ethylenebis(4,5,6,7-tetrahydro-1-indenyl)zirconium(IV) dichloride ([*meso*-(ebthi)$ZrCl_2$], LANXESS) and dimethylsilylenebis(cyclopentadienyl)zirconium(IV) dichloride ([$Me_2Si(C_5H_4)_2ZrCl_2$], MCAT) were stored under argon and used as received. Benzophenone (99%, Fluka) was dried in *vacuo* prior to use and stored under argon. Acetophenone (99%, Sigma-Aldrich) was condensed prior to use in a Schlenk flask and stored under argon. Acetone (≥99.8%, Fisher Chemical) was degassed by three freeze-pump-thaw cycles, and stored over 3 Å molecular sieves. [$Li_2(Me_3SiC_3SiMe_3)$] was prepared according to literature procedure and isolated as white solid.[1] The synthesis of [$Ir(\mu\text{-}Cl)(CO)_2]_2$ was done according to literature.[2] Preparative chromatography was performed by elution from columns of slurry-packed Silica Gel 60 (0.04-0.063 mm, Macherey-Nagel GmbH).

NMR spectra were determined on Bruker AV300 and AV400. ^1H and ^{13}C chemical shifts were referenced to the solvent signal: benzene-*d*$_6$ (δ_H 7.16 ppm, δ_C 128.06 ppm).[3]

Raman spectra were recorded on a LabRAM HR 800 Raman Horiba spectrometer equipped with an Olympus BX41 microscope with variable lenses. The samples were excited by different laser sources: 633 nm (17 mW, air cooled), 784 nm Laser diode (100 mW, air-cooled) or 473 nm Ar+ Laser (20 mW, air-cooled). All measurements were carried out at ambient temperature.

IR spectra were recorded on a Bruker Alpha FT-IR, ATR Spectrometer, spectra are not corrected.

MS analysis was done using a Finnigan MAT 95-XP instrument (Thermo-Electron) in CI^+/CI^- mode (isobutene) and for the air stable compounds in EI mode.

Melting points are uncorrected and were determined in sealed capillaries under Ar atmosphere using a Mettler-Toledo MP 70.

X-Ray diffraction data were collected on a STOE IPDS II (**2a**) and a Bruker Kappa APEX II Duo diffractometer (**2b**, **3a**, **3b**, **4**, **6**, **13**, **14**, **19** and **20**), respectively. The structures were solved by direct methods (SHELXS-97)[4] and refined by full-matrix least-squares procedures on F^2 (SHELXL-2014).[5] Diamond[6] was used for graphical representations.

For complex **3b** the highest peak (4.58 e· Å^{-3}) in the final difference Fourier map is located 0.76 Å and the deepest hole (-1.82 e· Å^{-3}) 0.60 Å from Zr1, which is due to the overlay of two orientations of the complex in the solid. The lower occupied position of the molecule is about

10% and therefore could not be satisfactorily resolved. Nevertheless, the highest residual electron densities (4.58, 0.81, 0.77 and 0.75 e· $Å^{-3}$) coincide with the minor position of the Zr and the propargyl/allenyl unit, indicating that in the second position the whole molecule is rotated by approximately 180° around the Zr1-Si1 axis.

All DFT calculations were carried out with the Gaussian 16 package of molecular orbital programs.

2. Synthesis of Compounds

2.1 Synthesis of bis-1,3-trimethylsilylpropyne

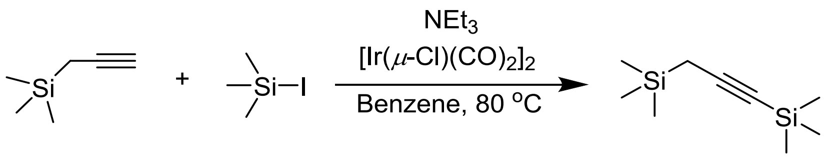

$[Ir(\mu\text{-}Cl)(CO)_2]_2$ (25.4 mg, 0.045 mmol, 0.0025 equiv.) was placed in a prepared Schlenk flask, then benzene (25 mL) and NEt_3 (2.73g, 27 mmol, 1.5 equiv.) were added. The mixture obtained was stirred for 30 minutes. The trimethylpropargylsilane (2.02 g, 18 mmol, 1 equiv.) and iodotrimethylsilane (4.7 g, 23.4 mmol, 1.3 equiv.) were added into the Schlenk flask. The deep brown solution was cooled to -78 °C, degassed and heated to 80 °C for three days. Then the reacting solution was cooled to room temperature, $[Ir(\mu\text{-}Cl)(CO)_2]_2$ (25.4 mg, 0.045 mmol, 0.0025 equiv.) was added into the reacting solution under argon atmosphere, then the solution was cooled to -78 °C, degassed and heated to 80 °C for another three days. After checking the progress of the reaction by NMR spectroscopy the characteristic peak of trimethylpropargylsilane had disappeared. Water (5 mL) was added to quench the reaction. The mixture was washed using brine (3 x 10 mL) and extracted with diethyl ether (3 x 15 mL). The combined brown organic phase was then dried over Na_2SO_4, concentrated on the rotary evaporator and condensed using reduced pressure and liquid nitrogen cooling. A colorless oil was obtained (1.69 g, 51 %). The yield was calculated by NMR with heptane as the internal standard (Figure S1).

^{1}H NMR (25 °C, benzene-d_6, 400.1 MHz): δ 1.42 (s, 2H, CH_2), 0.23 (s, $^2J_{H, Si}$ = 7.0 Hz, 9H, 3 x $SiMe_3$), 0.03 (s, $^2J_{H, Si}$ = 6.7 Hz, 9H, 3 x $SiMe_3$).

It should be noted that in order to increase the yield, the evaporation process should be carried out gently to avoid loss of the product. This will then lead to residual benzene in the product. Since the lithiation of bis-1,3-trimethylsilylpropyne in the next step is done using benzene as solvent, the residual benzene will have no effect on following reactions.

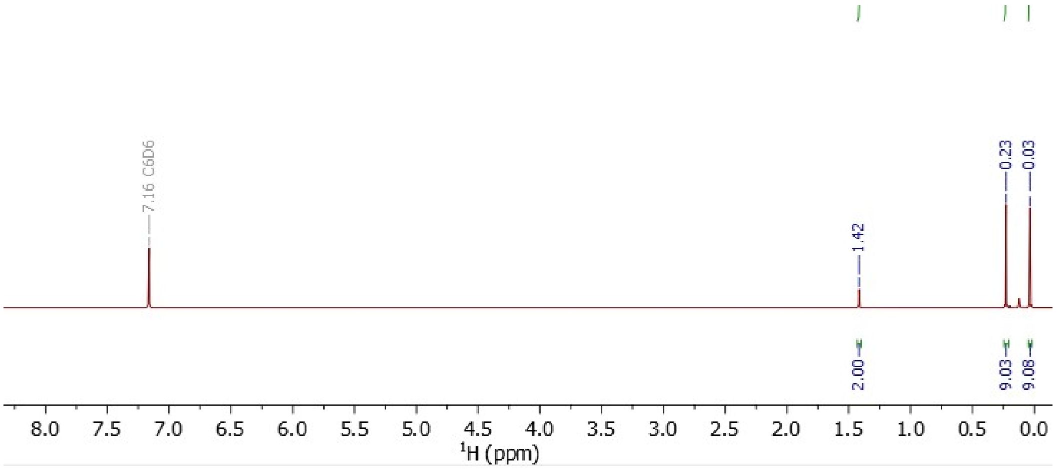

Figure S1. ^{1}H NMR spectrum of bis-1,3-trimethylsilylpropyne (25 °C, benzene-d_6, 400.1 MHz).

2.2 Synthesis of 2a

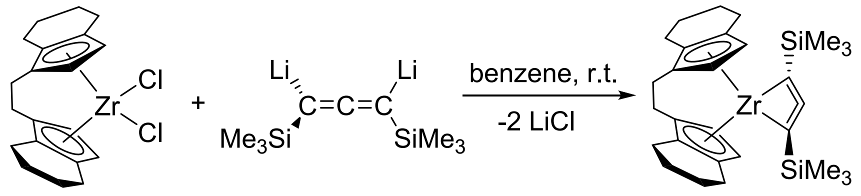

2a

[*rac*-(ebthi)$ZrCl_2$] (0.23 mmol, 100 mg) and [$Li_2(Me_3SiC_3SiMe_3)$] (0.23 mmol, 45 mg) were dissolved in benzene (15 mL) at room temperature. Then the resulting pale-yellow solution was stirred for 2 days and turned bright green. After filtration, the solvent was removed in *vacuo* and then the residue was dissolved in pentane. The solution was concentrated and stored at -30 °C for crystallization to obtain complex **2a** (Isolated yield 82%, 101 mg; NMR yield 90%, heptane as internal standard). Single crystals suitable for single crystal diffraction were grown from pentane.

m.p. 90-93 °C (Ar.). **^{1}H NMR** (25 °C, benzene-d_6, 400.1 MHz): δ 7.20 (d, $^3J_{H,H}$ = 3.3 Hz, 2H, C*H* ebthi), 5.35 (d, $^3J_{H,H}$ = 3.3 Hz, 2H, C*H* ebthi), 2.65 (s, 4H, 2 x CH_2 ebthi), 2.45-2.33 (m, 4H, 2 x CH_2 ebthi), 2.14-2.07 (m, 2H, CH_2 ebthi), 1.65-1.23 (m, 10H, 5 x CH_2 ebthi), 0.36 (s, $^2J_{H,Si}$ = 6.5 Hz, 18H, 2 x $SiMe_3$). **^{13}C NMR** (25 °C, benzene-d_6, 100.6 MHz): δ 164.7 (*C*=C=*C*), 151.4 (C=*C*=C), 125.2, 123.0, 119.1 (6 x *C* ebthi), 114.2, 101.9 (4 x CH ebthi), 27.2, 24.0, 23.8, 23.4, 22.9 (10 x CH_2 ebthi), 2.7 (2 x $SiMe_3$). **^{29}Si-inept NMR** (25 °C, benzene-d_6, 79.5 MHz): δ -12.71 (dec, $^2J_{H,Si}$ = 6.5 Hz, *Si*Me_3). (Minor impurity of **3a** was generally observed due to the slow conversion of **2a** to **3a** at room temperature.) **IR** (ATR, 32 scans, cm^{-1}): 2927 (w), 2897 (w), 2853 (w), 1890 (w), 1855 (w), 1731 (m), 1435 (w), 1338 (w), 1284 (w), 1241 (m), 1136 (w), 1035 (w), 1000 (w), 938 (w), 831 (s), 773 (s), 750 (m), 684 (m), 629 (w), 604 (w), 532 (w), 501 (w), 404 (w). **Raman** (784 nm, 20 sec, 15 acc, cm^{-1}): 535 (m), 579 (s), 601 (s), 626 (vs), 679 (s), 745 (s), 805 (vs), 851 (m), 952 (w), 975 (w), 1064 (w), 1143 (w), 1258 (m),1290 (m),1339 (m),1438 (m),1569 (w), 2099 (vw), 2158 (vw), 2903 (vw), 2944 (vw). **MS-CI$^+$** (*isobutane*): [M^+] 536 (100), 443 (73)*, [ebthi$^+$] 267 (24), [$(Me_3SiCHCCHSiMe_3+H)^+$] 185 (82), [$SiMe_3^+$] 73 (22).

2.3 Synthesis of 2b

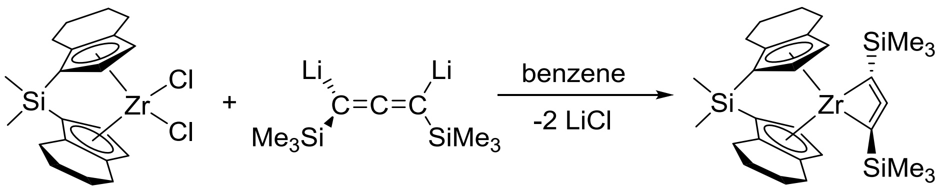

2b

[*rac*-Me_2Si(thi)$ZrCl_2$] (0.22 mmol, 100 mg) and [$Li_2(Me_3SiC_3SiMe_3)$] (0.22 mmol, 43 mg) were dissolved in benzene (3 mL). After 2 days of stirring at room temperature or 1 hour of stirring at 60 °C, the green solution was filtered and then the solvent was removed in *vacuo*. After

* This signal could not be identified. Based on its isotopic pattern the species should contain Zr.

dissolving in hexane, the residue was concentrated and stored at -30 °C for crystallization to obtain complex **2b** (Isolated yield 84%, 105 mg; NMR yield 87%, hexane as internal standard). Single crystals suitable for single crystal diffraction were grown from pentane.

m.p. 91-94 °C (Ar.). **^{1}H NMR** (25 °C, benzene-d_6, 400.1 MHz): δ 7.43 (d, $^3J_{H, H}$ = 3.2 Hz, 2H, C*H* indenyl), 5.40 (d, $^3J_{H, H}$ = 3.2 Hz, 2H, C*H* indenyl), 2.45-2.38 (m, 2H, CH_2 indenyl), 2.25-2.11 (m, 4H, 2 x CH_2 indenyl), 1.69-1.62 (m, 2H, CH_2 indenyl), 1.46-1.19 (m, 8H, 4 x CH_2 indenyl), 0.51 (s, $^2J_{H, Si}$ = 7.0 Hz, 6H, $SiMe_2$), 0.36 (s, $^2J_{H, Si}$ = 6.5 Hz, 18H, 2 x $SiMe_3$). **^{13}C NMR** (25 °C, benzene-d_6, 100.6 MHz): δ 173.2 (*C*=C=*C*), 147.0 (C=*C*=C), 129.8, 123.0 (4 x *C* indenyl), 120.1, 105.5 (4 x *C*H indenyl), 98.5 (2 x *C* indenyl), 26.8, 24.4, 23.4, 23.2 (8 x *C*H_2 indenyl), 2.5 (2 x $SiMe_3$), -2.1 (2 x $SiMe_2$). **^{29}Si-inept NMR** (25 °C, benzene-d_6, 79.5 MHz): δ -11.73 (dec, $^2J_{H, Si}$ = 6.5 Hz, *Si*Me_3), -14.76 (sep, $^2J_{H, Si}$ = 7.0 Hz, *Si*Me_2). (Minor impurity of **3b** was generally observed due to the slow conversion of **2b** to **3b** at room temperature.) **IR** (ATR, 32 scans, cm^{-1}): 2929 (w), 2895 (w), 2853 (w), 1900 (w), 1857 (w), 1834 (w), 1731 (m), 1441 (w), 1418 (w), 1340 (w), 1241 (m), 1125 (w), 1037 (w), 965 (w), 940 (w), 827 (s), 777 (s), 750 (m), 680 (s), 627 (w), 604 (w), 532 (w), 480 (w), 425 (w), 402 (w). **MS-CI$^+$** (*isobutane*): [($Me_3SiCHCCHSiMe_3$+H)$^+$] 185 (6), [M$^+$] 566 (100).

2.4 Synthesis of 3a

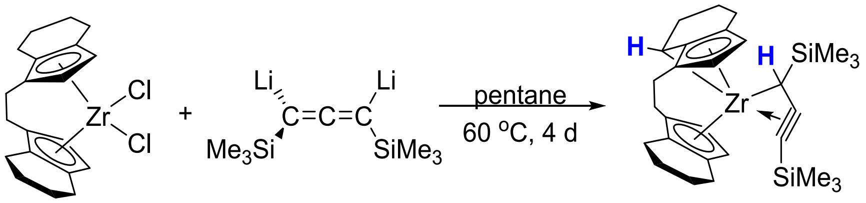

3a

[*rac*-(ebthi)$ZrCl_2$] (0.23 mmol, 100 mg) and [$Li_2(Me_3SiC_3SiMe_3)$] (0.23 mmol, 45 mg) were dissolved in pentane (6 mL). After 4 days of stirring at 60 °C, the brown solution was filtered and then the solvent was removed in *vacuo*. After dissolving in hexane, the residue was concentrated in vacuum and stored at -30 °C for crystallization to obtain complex **3a** (109 mg, 88%). Single crystals suitable for single crystal diffraction were grown from benzene at room temperature.

m.p. 127-130 °C (dec. Ar.). **^{1}H NMR** (25 °C, benzene-d_6, 400.1 MHz): δ 5.75 (d, $^3J_{H, H}$ = 3.3 Hz, 1H, C*H* ebthi), 5.72 (d, $^3J_{H, H}$ = 3.3 Hz, 1H, C*H* ebthi), 5.48 (d, $^3J_{H, H}$ = 3.2 Hz, 1H, C*H* ebthi), 5.43 (d, $^3J_{H, H}$ = 3.2 Hz, 1H, C*H* ebthi), 4.23 (d, $^3J_{H, H}$ = 8.1 Hz, 1H, C*H* ebthi), 3.08-2.97 (m, 1H), 2.84-2.78(m, 1H), 2.52-2.06 (m, 9H), 1.95-1.40 (m, 8H, CH_2 ebthi), 0.34 (s, $^2J_{H, Si}$ = 6.5 Hz, 9H, C≡C-$SiMe_3$), 0.28 (s, $^2J_{H, Si}$ = 6.7 Hz, 9H, C≡C-CH-$SiMe_3$). **^{13}C NMR** (25 °C, benzene-d_6, 100.6 MHz): δ 141.9 (C≡*C*-$SiMe_3$), 124.0, 124.0, 121.6, 119.0, 114.2 (5 x *C* ebthi), 113.8 (*C*H ebthi), 113.7 (*C* ebthi), 102.6, 99.6, 97.1 (3 x *C*H ebthi), 96.2 (*C*≡C-$SiMe_3$), 68.4 (*C*H ebthi), 51.5 (C≡C-*C*H-$SiMe_3$), 29.4, 28.1, 25.5, 24.9, 24.3, 23.8, 23.7, 22.3, 21.6 (9 x *C*H_2 ebthi), 2.4 ($SiMe_3$), 2.1 ($SiMe_3$). **^{29}Si-inept NMR** (25 °C, benzene-d_6, 79.5 MHz): δ -1.66 (dec, $^2J_{H, Si}$ = 6.5 Hz, C≡C-*Si*Me_3), -7.55(m, C≡C-CH-*Si*Me_3). **IR** (ATR, 32 scans, cm^{-1}): 3086 (vw), 2993 (vw), 2929 (w), 2906 (w), 2855 (w), 1892 (m), 1443 (w), 1371 (w), 1315 (w), 1243 (m), 1134 (w), 1037 (w), 998 (w), 835 (s), 777 (s),

759 (m), 690 (m), 629 (w), 606 (w), 532 (w), 505 (w), 462 (w), 451 (w), 408 (w). **MS-CI$^+$** (*isobutane*): [M$^+$] 536 (100), [M-SiMe$_3^+$] 463 (5), [M-Me$_3$SiCHC$_2$SiMe$_3^+$] 351 (7).

2.5 Synthesis of 3b

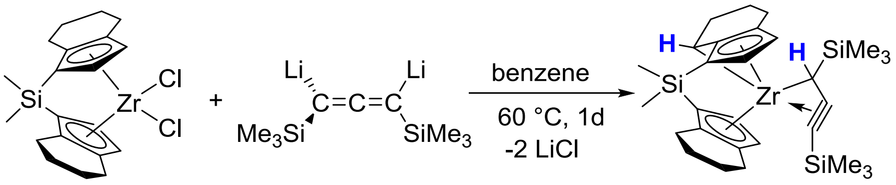

3b

[*rac*-Me$_2$Si(thi)ZrCl$_2$] (0.22 mmol, 100 mg) and [Li$_2$(Me$_3$SiC$_3$SiMe$_3$)] (0.22 mmol, 43 mg) were dissolved in benzene (3 mL). After one night of stirring at 60 °C, the brown solution was filtered and then the solvent was removed in *vacuo*. After dissolving in hexane, the residue was concentrated in vacuum and stored at -30 °C for crystallization to obtain complex **3b** (114 mg, 91%). Single crystals suitable for single crystal diffraction were grown from hexane.

m.p. 107-111°C (Ar.). **^{1}H NMR** (25 °C, benzene-*d*$_6$, 400.1 MHz): δ 5.86 (d, $^3J_{H,H}$ = 3.1 Hz, 1H, C*H* indenyl), 5.74 (d, $^3J_{H,H}$ = 2.9 Hz, 1H, C*H* indenyl), 5.62 (d, $^3J_{H,H}$ = 2.9 Hz, 1H, C*H* indenyl), 5.35 (d, $^3J_{H,H}$ = 3.1 Hz, 1H, C*H* indenyl), 3.79 (d, $^3J_{H,H}$ = 8.2 Hz, 1H, C*H* indenyl), 2.96-2.87 (m, 1H, C*H*$_2$ indenyl), 2.78-2.71 (m, 1H, C*H*$_2$ indenyl), 2.55-2.34 (m, 4H, C*H*$_2$ indenyl), 2.02-1.94 (m, 1H, C*H*$_2$ indenyl), 1.91-1.69 (m, 4H, C*H*$_2$ indenyl), 1.60-1.44 (m, 3H, C*H*$_2$ indenyl), 0.73-0.64 (m, 1H, C≡C-C*H*), 0.41 (s, $^2J_{H,Si}$ = 7.0 Hz, 3H, SiMe$_2$), 0.33 (s, $^2J_{H,Si}$ = 6.5 Hz, 9H, C≡C-SiMe$_3$), 0.26 (s, $^2J_{H,Si}$ = 6.7 Hz, 9H, C≡C-CH-SiMe$_3$), 0.22 (s, $^2J_{H,Si}$ = 7.0 Hz, 3H, SiMe$_2$). **^{13}C NMR** (25 °C, benzene-*d*$_6$, 100.6 MHz): δ 139.3 (C≡*C*-SiMe$_3$), 129.7, 129.1 (2 x *C* indenyl), 123.7 (*C*H indenyl), 116.0, 115.0 (2 x *C* indenyl), 104.4, 104.3, 103.3(3 x *C*H indenyl), 96.3 (*C*≡C-SiMe$_3$), 87.4, 86.3 (2 x *C* indenyl), 69.9 (*C*H indenyl), 51.4 (C≡C-*C*H), 28.4, 28.2, 24.7, 24.2, 23.7, 22.3, 21.9 (7 x *C*H$_2$ indenyl), 2.4, 1.8 (2 x SiMe$_3$), -1.2, -3.9 (SiMe$_2$). **^{29}Si-inept NMR** (25 °C, benzene-*d*$_6$, 79.5 MHz): δ -1.24 (dec, $^2J_{H,Si}$ = 6.5 Hz, C≡C-*Si*Me$_3$), -7.91 (m, C≡C-CH-*Si*Me$_3$), -15.42 (sep, $^2J_{H,Si}$ = 7.0 Hz, *Si*Me$_2$). **IR** (ATR, 32 scans, cm^{-1}): 3083 (vw), 3010 (vw), 2927 (m), 2897 (w), 2853 (w), 1898 (m), 1857 (w), 1443 (w), 1420 (w), 1354 (w), 1315 (w), 1245 (s), 1177 (w), 1125 (w), 1097 (w), 1041 (w), 967 (w), 829 (s), 794 (s), 779 (s), 756 (s), 678 (s), 664 (m), 625 (m), 550 (w), 530 (w), 466 (m), 447 (m). **MS-CI$^+$** (*isobutane*): [M+H$^+$] 567 (100), [M$^+$] 566 (64), [M-Me$^+$] 551 (6), [M-SiMe$_3^+$] 493 (3), [M-Me$_3$SiCHC$_2$SiMe$_3^+$] 381 (6).

2.6 Synthesis of 18a

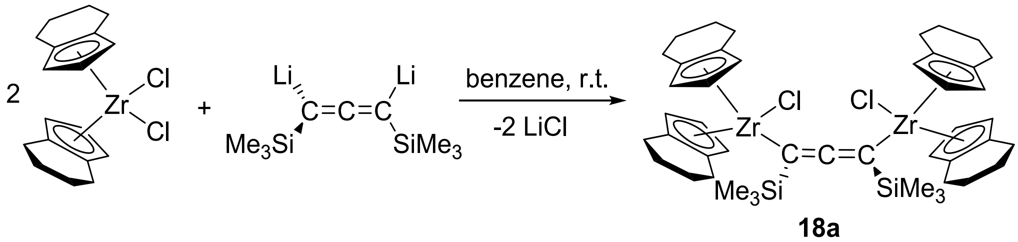

18a

[(thi)$_2$ZrCl$_2$] (2 eq, 0.3 mmol, 120.2 mg) and [Li$_2$(Me$_3$SiC$_3$SiMe$_3$)] (1 eq, 0.15 mmol, 29.4 mg) were added into a dried Schlenk flask and then dissolved in benzene (10 mL). After 2 hours of stirring at room temperature, the orange solution was filtered and then the solvent was removed in *vacuo*. After dissolving in toluene, the residue was concentrated and stored at -30 °C for crystallization to obtain complex **18a** (122 mg, 89%).

m.p. 146-149 °C (Ar.). **^{1}H NMR** (25 °C, benzene-*d*$_6$, 300.2 MHz): δ 6.73 (dd, $^3J_{H, H}$ = 3.1, 2.3 Hz, 2H, C*H* thi), 6.58 (dd, $^3J_{H, H}$ = 3.0, 2.3 Hz, 2H, C*H* thi), 6.18 (t, $^3J_{H, H}$ = 3.1 Hz, 2H, C*H* thi), 5.94 (t, $^3J_{H, H}$ = 3.1 Hz, 2H, C*H* thi), 5.49 (dd, $^3J_{H, H}$ = 3.1, 2.3 Hz, 2H, C*H* thi), 5.43 (dd, $^3J_{H, H}$ = 3.0, 2.2 Hz, 2H, C*H* thi), 3.28-3.17 (m, 4H, C*H*$_2$ thi), 2.69-2.56 (m, 4H, C*H*$_2$ thi), 2.35-2.27 (m, 8H, C*H*$_2$ thi), 1.94-1.64 (m, 8H, C*H*$_2$ thi), 1.54-1.34 (m, 8H, C*H*$_2$ thi), 0.54 (s, $^2J_{H, Si}$ = 6.2 Hz, 18H, SiMe$_3$). **^{13}C NMR** (25 °C, benzene-*d*$_6$, 100.6 MHz): δ 183.5 (C=*C*=C), 132.2, 132.0, 125.5, 123.6 (4 x *C* thi), 118.1, 114.5, 109.7, 108.6, 108.5, 107.6 (6 x *C*H thi), 101.7 (*C*=C=*C*), 25.4, 25.1, 24.71, 24.67, 23.1, 22.99, 22.97, 22.8 (8 x CH$_2$ thi), 4.1 (SiMe$_3$). **^{29}Si-inept NMR** (25 °C, benzene-*d*$_6$, 79.5 MHz): δ -7.36 (dec, $^2J_{H, Si}$ = 6.2 Hz, *Si*Me$_3$). **MS-CI$^+$** (*isobutane*): [(thi)$_2$ZrCl$^+$] 363 (100), [(Me$_3$SiCHCCHSiMe$_3$+H)$^+$] 185 (81), [SiMe$_3$$^+$] 73 (9).

2.7 Synthesis of 18b

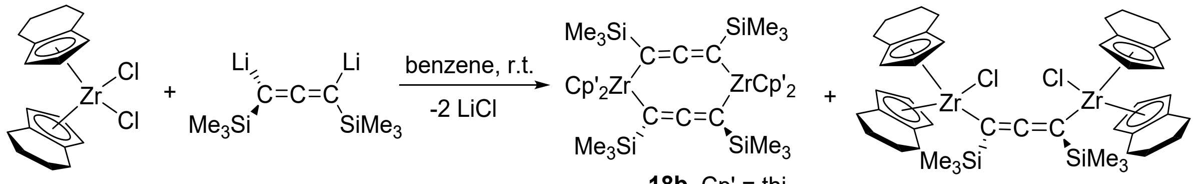

18b, Cp' = thi   **18a**

[(thi)$_2$ZrCl$_2$] (1 eq, 0.15 mmol, 60.1 mg) and [Li$_2$(Me$_3$SiC$_3$SiMe$_3$)] (1 eq, 0.15 mmol, 29.4 mg) were added into a dried Schlenk flask and then dissolved in benzene (10 mL). After 2 hours of stirring at room temperature, the brown solution was filtered and then the solvent was removed in *vacuo*. The residue was the mixture of complex **18b** and **18a** (60% and 40%).

^{1}H NMR (25 °C, benzene-*d*$_6$, 400.1 MHz): δ 6.27 (d, $^3J_{H, H}$ = 3.2 Hz, 8H, C*H* thi), 5.55 (t, $^3J_{H, H}$ = 3.2 Hz, 4H, C*H* thi), 2.37-2.28(m, 12H, C*H*$_2$ thi), 2.05-1.98 (m, 8H, C*H*$_2$ thi), 1.52-1.18 (m, 12H, C*H*$_2$ thi), 0.32 (s, $^2J_{H, Si}$ = 6.6 Hz, 36H, SiMe$_3$). **^{13}C NMR** (25 °C, benzene-*d*$_6$, 100.6 MHz): δ 167.8 (*C*=C=*C*), 148.4 (C=*C*=C), 123.3 (*C* thi), 109.3, 101.9 (2 x *C*H thi), 24.8, 23.2 (2 x *C*H$_2$ thi), 2.8 (SiMe$_3$). **^{29}Si-inept NMR** (25 °C, benzene-*d*$_6$, 79.5 MHz): δ -12.80 (dec, $^2J_{H, Si}$ = 6.6 Hz, *Si*Me$_3$). **MS-CI$^+$** (*isobutane*): [M$^+$] 1024 (5), [(M-(thi)$_2$Zr-Me$_3$SiC$_3$SiMe$_3$+H)$^+$] 511 (100), [(M-(thi)$_2$Zr-Me$_3$SiC$_3$SiMe$_3$-SiMe$_3$+2H)$^+$] 439 (42), [(Me$_3$SiCHCCHSiMe$_3$+H)$^+$] 185 (69), 117 (30), [SiMe$_3$$^+$] 73 (26).

2.8 Synthesis of 19

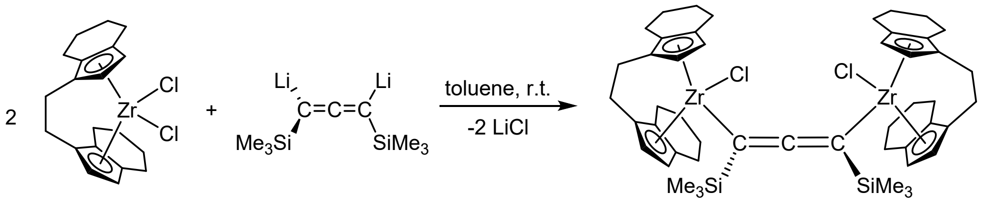

[*meso*-(ebthi)$ZrCl_2$] (2 eq, 0.3 mmol, 128.0 mg) and [$Li_2(Me_3SiC_3SiMe_3)$] (1 eq, 0.15 mmol, 29.4 mg) were added into a dried Schlenk flask and then dissolved in toluene (20 mL) at room temperature. Then the resulting red solution was stirred for 2 hours and turn to orange. After filtration, the solution was concentrated in vacuum and stored at -30 °C for crystallization to obtain orange crystal **19** (119 mg, 82%).

m.p. 165.8 °C (dec. Ar.). **^{1}H NMR** (25 °C, benzene-d_6, 400.1 MHz): δ 7.02 (d, $^3J_{H,H}$ = 2.9 Hz, 2H, C*H* ebthi), 6.73 (d, $^3J_{H,H}$ = 2.9 Hz, 2H, C*H* ebthi), 5.97 (d, $^3J_{H,H}$ = 2.9 Hz, 2H, C*H* ebthi), 5.81 (d, $^3J_{H,H}$ = 2.8 Hz, 2H, C*H* ebthi), 3.25-3.36 (m, 4H), 2.56-2.80 (m, 10H), 2.20-2.55 (m, 10H), 1.18-2.13 (m, 10H), 1.34-2.52 (m, 8H), 0.54 (s, $^2J_{H,Si}$ = 6.2 Hz, 18H, 2 x $SiMe_3$). **^{13}C NMR** (25 °C, benzene-d_6, 100.6 MHz): δ 183.5 (C=*C*=C), 132.1, 132.3, 128.2, 127.3, 125.4, 123.9 (6 x *C* ebthi), 116.9, 116.4, 108.1, 106.4 (4 x *C*H ebthi), 98.9 (*C*=C=*C*), 28.5, 27.7, 25.2, 25.2, 23.6, 23.4, 22.9, 22.7, 22.6, 21.4 (10 x *C*H_2 ebthi), 3.9 ($SiMe_3$). **^{29}Si-inept NMR** (25 °C, benzene-d_6, 79.5 MHz): δ -7.05 (dec, $^2J_{H,Si}$ = 6.2 Hz, *Si*Me_3). **MS-CI$^+$** (*isobutane*): [M-Cl$^+$] 927 (4), [M-(ebthi)$ZrCl_2^+$] 536 (100), [(ebthi)$ZrCl^+$] 389 (82), [($Me_3SiCHCCHSiMe_3$+H)$^+$] 185 (13), [$SiMe_3^+$] 73 (2). Single crystals suitable for single crystal diffraction were grown from hexane under -40 °C.

2.9 Synthesis of 20

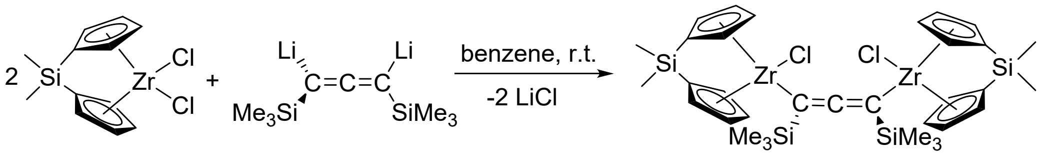

[$Me_2Si(C_5H_4)_2ZrCl_2$] (2 eq, 0.3 mmol, 104.5 mg) and [$Li_2(Me_3SiC_3SiMe_3)$] (1 eq, 0.15 mmol, 29.4 mg) were added into a dried Schlenk flask and then dissolved in benzene (10 mL). After 2 hours of stirring at room temperature, the orange solution was filtered and then the solvent was removed in *vacuo*. The complex **20** was isolated in 117 mg, 95% yield.

m.p. 200-203 °C (Ar.). ^{1}H NMR (25 °C, benzene-d_6, 400.1 MHz): δ 7.80 (td, $^3J_{H,H}$ = 3.1, 1.8 Hz, 2H, C*H* Cp), 7.34 (td, $^3J_{H,H}$ = 3.0, 1.8 Hz, 2H, C*H* Cp), 7.06 (td, $^3J_{H,H}$ = 3.0, 1.9 Hz, 2H, C*H* Cp), 6.85 (td, $^3J_{H,H}$ = 3.0, 2.0 Hz, 2H, C*H* Cp), 5.96-5.93 (m, 4H, C*H* Cp), 5.70-5.65 (m, 4H, C*H* Cp), 0.41 (s, $^2J_{H,Si}$ = 6.4 Hz, 18H, $SiMe_3$), 0.28 (s, $^2J_{H,Si}$ = 7.2 Hz, 6H, $SiMe_2$), 0.18 (s, $^2J_{H,Si}$ = 7.2 Hz, 6H, $SiMe_2$). ^{13}C NMR (25 °C, benzene-d_6, 100.6 MHz): δ 173.4 (C=*C*=C), 126.0, 125.4, 123.9, 123.2, 117.0, 116.9, 110.6 (7×*C*H Cp), 110.5 (*C*=C=*C*), 109.9 (*C*H Cp), 107.2 (*C* Cp), 106.5 (*C* Cp), 3.1 ($SiMe_3$), -4.8 ($SiMe_2$), -6.2 ($SiMe_2$). **^{29}Si-inept NMR** (25 °C, benzene-d_6, 79.5 MHz): δ -3.80 (dec, $^2J_{H,Si}$ = 6.4 Hz, *Si*Me_3), -14.33 (sep, $^2J_{H,Si}$ = 7.2 Hz, *Si*Me_2). **MS-CI$^+$** (*isobutane*): [M-HCl$^+$] 771 (28), [M-

2xHCl$^+$] 735 (16), [M-$Me_2Si(C_5H_4)_2ZrCl_2^+$] 459 (100). Single crystals suitable for single crystal diffraction were grown from toluene under -30 ^oC.

3. Reactivity of Complexes

3.1 Reactivity of 2a with ketones

3.1.1 Reaction of 2a with benzophenone to (4,4-diphenylbut-3-en-1-yne-1,3-diyl)bis(trimethylsilane) (5)

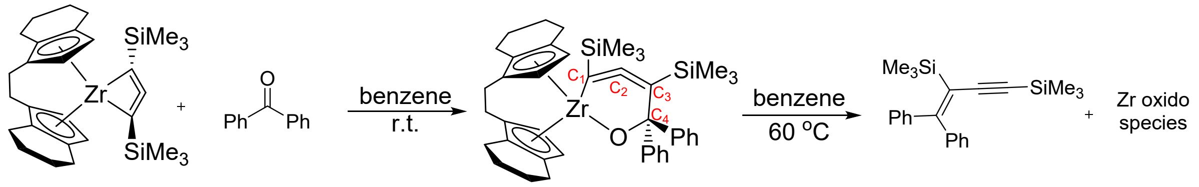

Compound **2a** (0.20 mmol, 0.108 g) and benzophenone (0.20 mmol, 37 mg) were dissolved in benzene (2 mL). After one day of stirring at room temperature the solvent was removed in *vacuo*. An NMR sample was taken and the crude NMR spectrum showed that intermediate **4** was the major species (conversion of **2a**: 100%). Then the orange residue was dissolved in hexane and stored at -30 °C for crystallization to obtain the intermediate **4**. Single crystals suitable for single crystal diffraction were grown from hexane.

m.p. 128-131 °C (Ar.). **^{1}H NMR** (25 °C, benzene-d_6, 300.2 MHz): δ 7.57-7.51 (m, 4H, Ph), 6.64 (d, $^3J_{H,H}$ = 3.0 Hz, 1H, C*H* ebthi), 6.24 (d, $^3J_{H,H}$ = 2.7 Hz, 1H, C*H* ebthi), 5.85 (d, $^3J_{H,H}$ = 2.9 Hz, 1H, C*H* ebthi), 5.13 (d, $^3J_{H,H}$ = 2.8 Hz, 1H, C*H* ebthi), 0.43 (s, $^2J_{H,Si}$ = 6.5 Hz, 9H, $SiMe_3$), 0.10 (s, $^2J_{H,Si}$ = 6.5 Hz, 9H, $SiMe_3$). **^{13}C NMR** (25 °C, benzene-d_6, 100.6 MHz): δ 180.6 (C2), 152.1, 150.8, 135.5, 135.1, 130.4, 129.1, 128.9, 128.6, 127.3, 127.2, 126.8, 121.4, 120.6 (2 x *C* ebthi), 107.43, 107.38, 104.7, 104.6 (4 x *C*H ebthi), 98.6 (C3), 92.4 (C4), 28.6, 28.4, 25.3, 24.8, 24.4, 23.54, 23.50, 23.4, 23.1, 22.5 (10 x CH_2 ebthi), 2.7, 2.1 (2 x $SiMe_3$) (Pure **4** was not obtained after crystallization, compound **13** was obtained as an impurity due to **3a** contained in the starting material **2a**. A more precise assignment was not made, since the signals in the mixture cannot be assigned without any doubt). **^{29}Si-inept NMR** (25 °C, benzene-d_6, 79.5 MHz): δ -6.59 (dec, $^2J_{H,Si}$ = 6.5 Hz, *Si*Me$_3$), -9.78 (dec, $^2J_{H,Si}$ = 6.5 Hz, *Si*Me$_3$). **IR** (ATR, 32 scans, cm^{-1}): 3053 (vw), 3028 (vw), 3018 (vw), 2923 (w), 2902 (w), 2855 (w), 1814 (m), 1597 (w), 1488 (w), 1441 (w), 1245 (m), 1187 (w), 1160 (w), 1033 (w), 993 (m), 928 (w), 905 (w), 831 (s), 781 (s), 754 (s), 701 (s), 682 (s), 620 (s), 542 (m), 501 (m), 472 (m), 447 (w), 406 (s). **Raman** (784 nm, 20 sec, 15 acc, cm^{-1}): 543 (m), 553 (m), 572 (w), 619 (m), 635 (s), 683 (s), 727 (m), 759 (w), 817 (w), 903 (w), 955 (w), 1004 (s), 1032 (m), 1161 (w), 1176 (w), 1256 (w), 1290 (w), 1340 (w), 1447 (w), 1478 (w), 1581 (vw), 1597 (w), 1818 (w), 2894 (vw), 2939 (vw), 3057 (vw). **MS-CI$^+$** (*isobutane*): [M$^+$] 718 (8), 443 (100)[†], [$SiMe_3^+$] 73 (3).

After heating the above-mentioned reaction mixture at 60 °C for 2 days (or at r.t. for 5 days), an NMR sample was taken and the crude NMR showed that compound **5** was the resulting product, which was in agreement with the previously reported literature.[7]

^{1}H NMR (25 °C, benzene-d_6, 300.2 MHz): δ 7.65 (m, 2H, Ph), 7.05 (m, 8H, Ph), 0.17 (s, $^2J_{H,Si}$ =7.0 Hz, 9H, $SiMe_3$), 0.12 (s, $^2J_{H,Si}$ = 6.7 Hz, 9H, $SiMe_3$).

[†] This signal could not be identified. Based on its isotopic pattern the species should contain Zr.

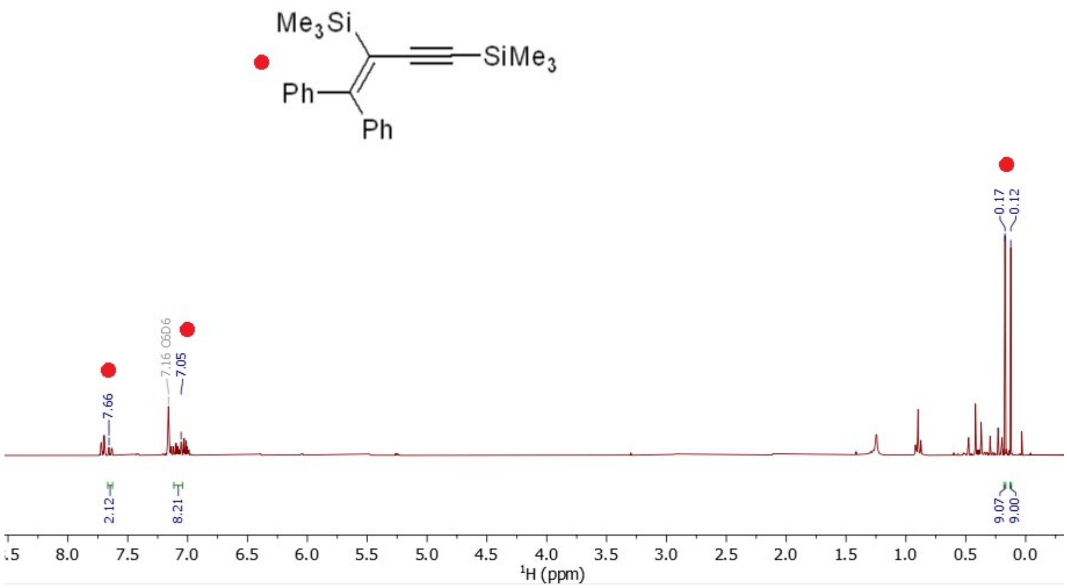

Figure S2. ^{1}H NMR spectrum of the reaction mixture for the formation of **5** (25 °C, benzene-d_6, 300.2 MHz).

3.1.2 Reaction of 2a with acetophenone to *E/Z* isomer mixture of (4-phenylpent-3-en-1-yne-1,3-diyl)bis(trimethylsilane) (7*E* and 7*Z*)

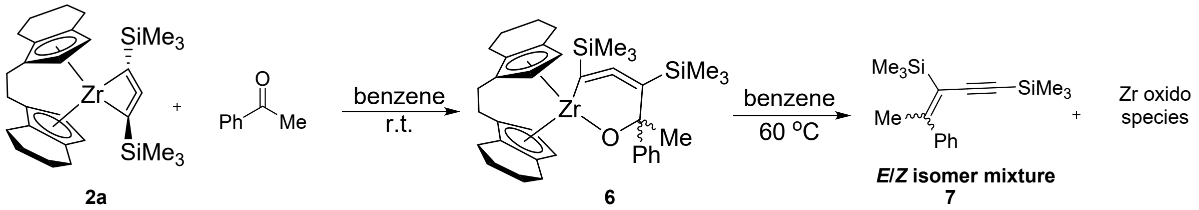

Compound **2a** (0.20 mmol, 0.108 g) was dissolved in benzene (2 mL) and acetophenone (0.20 mmol, 24 mg) was added. A color change to orange was observed immediately after addition of acetophenone. After one day of stirring at room temperature the solvent was removed in *vacuo*. An NMR sample was taken and the crude NMR spectrum showed that intermediate **4** was the major species (conversion of **2a**: 100%). Then the orange residue was dissolved in hexane and stored at -30 °C for crystallization to obtain the intermediate **6** as isomer mixture. Single crystals suitable for single crystal diffraction were grown from hexane.

m.p. 127-130 °C (Ar.). **^{1}H NMR** (25 °C, benzene-d_6, 400.1 MHz): δ 7.73-7.67 (m, 4H, Ph), 7.32-7.25 (m, 4H, Ph), 7.14-7.09 (m, 2H, Ph), 6.79 (d, $^3J_{H,H}$ = 3.1 Hz, 1H, C*H* ebthi), 6.64 (d, $^3J_{H,H}$ = 3.0 Hz, 1H, C*H* ebthi), 6.44 (d, $^3J_{H,H}$ = 2.8 Hz, 1H, C*H* ebthi), 6.30 (d, $^3J_{H,H}$ = 2.8 Hz, 1H, C*H* ebthi), 5.77 (d, $^3J_{H,H}$ = 3.0 Hz, 1H, C*H* ebthi), 5.50 (d, $^3J_{H,H}$ = 3.1 Hz, 1H, C*H* ebthi), 5.26 (d, $^3J_{H,H}$ = 2.8 Hz, 1H, C*H* ebthi), 5.17 (d, $^3J_{H,H}$ = 2.8 Hz, 1H, C*H* ebthi), 3.02-1.18 (m, 40H, 20 x CH_2 ebthi), 1.91 (s, 3H, CH_3), 1.86 (s, 3H, CH_3), 0.42-0.41 (m, 18H, 2 x $SiMe_3$), 0.27 (s, $^2J_{H,Si}$ = 6.5 Hz, 9H, $SiMe_3$), 0.07 (s, $^2J_{H,Si}$ = 6.7 Hz, 9H, $SiMe_3$). **^{13}C NMR** (25 °C, benzene-d_6, 100.6 MHz): δ 107.0, 106.7, 106.4, 105.8, 104.7, 104.4, 104.3, 103.2 (8 x CH ebthi), 2.5, 2.3, 1.9, 1.7 (4 x $SiMe_3$) (A more precise assignment was not made, since the small amount of isomer mixture was difficult to give a good spectrum). **^{29}Si-inept NMR** (25 °C, benzene-d_6, 79.5 MHz): δ -6.86 (m, 2 x *Si*Me_3), -9.28

(dec, $^{2}J_{H, Si}$ = 6.5 Hz, *Si*Me_3), -10.55 (dec, $^{2}J_{H, Si}$ = 6.5 Hz, *Si*Me_3). **IR** (ATR, 32 scans, cm^{-1}): 3092 (vw), 3055 (vw), 2945 (w), 2923 (w), 2855 (w), 2836 (w), 1855 (w), 1808 (m), 1597 (w), 1490 (w), 1441 (w), 1356 (w), 1259 (w), 1243 (m), 1214 (w), 1097 (w), 1053 (w), 1029 (w), 938 (w), 915 (w), 833 (s), 767 (s), 752 (s), 697 (s), 670 (s), 622 (m), 598 (m), 577 (w), 517 (w), 489 (w), 466 (w), 447 (w), 408 (s). **Raman** (784 nm, 15 sec, 10 acc, cm^{-1}): 518 (m), 624 (m), 632 (s), 652 (w), 679 (m), 712 (w), 732 (m), 756 (m), 766 (m), 934 (w), 1002 (s), 1027 (m), 1178 (w), 1215 (w), 1279 (w), 1458 (w), 1597 (w), 1809 (w), 2897 (vw), 2943 (vw). **MS-CI⁺** (*isobutane*): [M^+] 656 (3), [$M-Me^+$] 641 (7), [$M-Me_3SiC_3SiMe_3^+$] 473 (17), 443 (100)[‡], [$Me_3SiC_3SiMe_3CPhMe^+$] 287 (25), [$ebthi^+$] 267 (29), [$(Me_3SiCHCCHSiMe_3+H)^+$] 185 (9), [$SiMe_3^+$] 73 (6).

After heating the above-mentioned reaction mixture at 60 °C for 2 days, an NMR sample was taken and the crude NMR showed that *E* and *Z* isomers mixture of compound **7** (*E*/*Z* ratio 1:1) were the resulting products, which is in agreement with the previously reported literature.[7]

***7Z*: ¹H NMR** (25 °C, benzene-d_6, 300.2 MHz): δ 6.99 (m, 5H, Ph), 2.40 (s, 3H, CH_3), 0.29 (s, $^{2}J_{H, Si}$ =7.0 Hz, 9H, $SiMe_3$), 0.05 (s, $^{2}J_{H, Si}$ = 6.7 Hz, 9H, $SiMe_3$). ***7E*: ¹H NMR** (25 °C, benzene-d_6, 300.2 MHz): δ 7.52 (m, 2H, Ph), 7.20(m, 2H, Ph), 7.10(m, 1H, Ph), 2.03 (s, 3H, CH_3), 0.34 (s, $^{2}J_{H, Si}$ =6.6 Hz, 9H, $SiMe_3$), 0.11 (s, $^{2}J_{H, Si}$ = 7.0 Hz, 9H, $SiMe_3$).

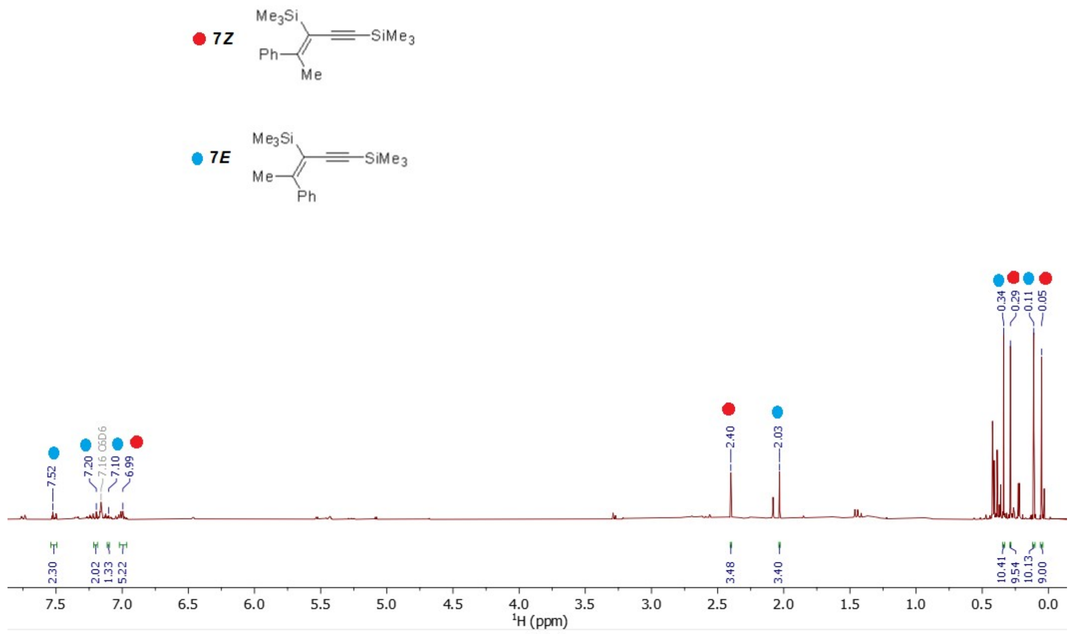

Figure S3. ¹H NMR spectrum of the reaction mixture for the formation of ***7E*/*7Z*** (25 °C, benzene-d_6, 300.2 MHz).

‡ This signal could not be identified. Based on its isotopic pattern the species should contain Zr.

3.1.3 Reaction of 2a with acetone to (4-methylpent-3-en-1-yne-1,3- diyl)bis(trimethylsilane) (9)

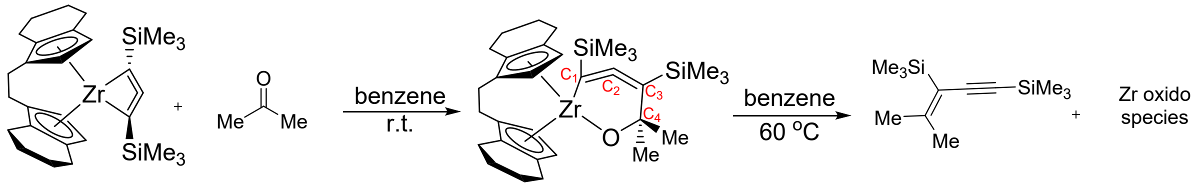

Compound **2a** (0.20 mmol, 0.108 g) was dissolved in benzene (2 mL) and acetone (0.20 mmol, 12 mg) was added. A color change to orange was observed immediately after addition of acetone. After one day of stirring at room temperature, an NMR sample was taken, and the crude NMR result proved enough to characterize the proposed intermediate **8** (conversion of **2a**: 100%).

m.p. 128-131 °C (Ar.). **^{1}H NMR** (25 °C, benzene-d_6, 300.2 MHz): δ 6.68 (d, $^{3}J_{H, H}$ = 3.1 Hz, 1H, C*H* ebthi), 6.43 (d, $^{3}J_{H, H}$ = 2.8 Hz, 1H, C*H* ebthi), 5.43 (d, $^{3}J_{H, H}$ = 3.1 Hz, 1H, C*H* ebthi), 5.18 (d, $^{3}J_{H, H}$ = 2.8 Hz, 1H, C*H* ebthi), 1.43 (s, 6H, 2 x C*H₃*), 0.38 (s, $^{2}J_{H, Si}$ = 6.5 Hz, 9H, $SiMe_3$), 0.33 (s, $^{2}J_{H, Si}$ = 6.5 Hz, 9H, $SiMe_3$) (A more precise assignment was not made, since the signals in the reaction mixture cannot be assigned without any doubt). **^{13}C NMR** (25 °C, benzene-d_6, 75.5 MHz): δ 180.6 (C2), 135.3 (C1), 133.8, 129.3, 127.3, 126.8, 121.2, 120.4 (6 x *C* ebthi), 106.9, 105.8, 104.1, 103.3 (4 x *C*H ebthi), 97.9 (C3), 82.9 (C4), 37.1, 31.7 (2 x CH_3), 28.7, 28.2, 25.0, 24.8, 24.7, 24.3, 23.6, 23.5, 23.0, 22.9 (10 x CH_2 ebthi), 2.4, 2.0 (2 x $SiMe_3$). **^{29}Si-inept NMR** (25 °C, benzene-d_6, 59.6 MHz): δ -7.17(dec, $^{2}J_{H, Si}$ = 6.5 Hz, *Si*Me₃), -10.48(dec, $^{2}J_{H, Si}$ = 6.5 Hz, *Si*Me₃). **IR** (ATR, 32 scans, cm^{-1}): 2949 (w), 2927 (w), 2853 (w), 2156 (vw), 1857 (w), 1810 (m), 1437 (w), 1367 (w), 1350 (w), 1243 (m), 1171 (w), 1136 (w), 1094 (w), 1008 (w), 936 (m), 829 (s), 775 (s), 752 (s), 684 (s), 653 (m), 618 (m), 565 (m), 491 (m), 464 (m), 406 (s). **MS-CI^{+}** (*isobutane*): [M+$OCMe_2^{+}$] 653 (24), [M+H^{+}] 595 (19), [M^{+}] 594 (13), [M-Me^{+}] 579 (10), 443 (100)[§], [M-$Me_3SiC_3SiMe_3^{+}$] 411 (51), [$ebthi^{+}$] 267 (52), [$Me_3SiC_3SiMe_3CMeO^{+}$] 225 (52), [($Me_3SiCHCCHSiMe_3$+H)$^{+}$] 185 (38), [$SiMe_3^{+}$] 73 (19).

After heating the above-mentioned reaction mixture at 60 °C for 2 days, an NMR sample was taken and the crude NMR showed that compound **9** was the resulting product, which is in agreement with the previously reported literature.[7]

^{1}H NMR (25 °C, benzene-d_6, 300.2 MHz): δ 2.03 (q, $^{4}J_{H, H}$ = 0.4 Hz, 3H, C*H₃*), 1.63 (q, $^{4}J_{H, H}$ = 0.4 Hz, 3H, C*H₃*), 0.27 (s, $^{2}J_{H, Si}$ =6.4 Hz, 9H, $SiMe_3$), 0.26 (s, $^{2}J_{H, Si}$ = 7.0 Hz, 9H, $SiMe_3$).

[§] This signal could not be identified. Based on its isotopic pattern the species should contain Zr.

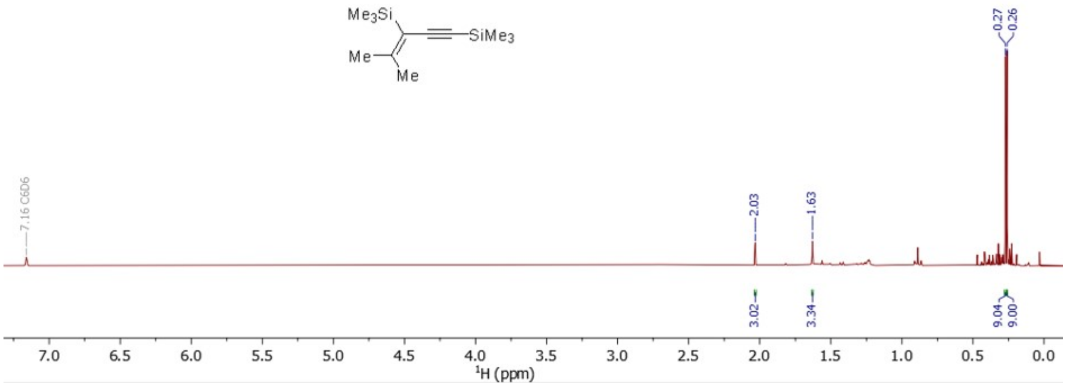

Figure S4. ^{1}H NMR spectrum of the reaction mixture for the formation of **9** (25 °C, benzene-d_6, 300.2 MHz).

3.2 Reaction of 2b with ketones

3.2.1 Reaction of 2b with benzophenone to (4,4-diphenylbut-3-en-1-yne-1,3-diyl)bis(trimethylsilane) (5)

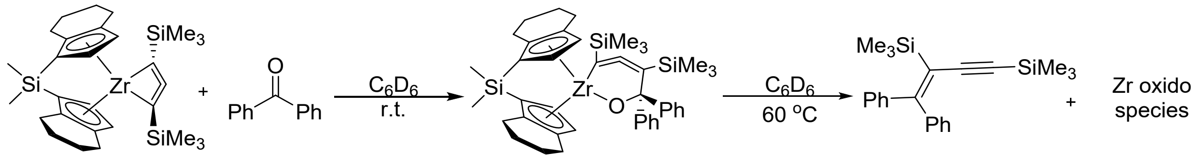

2b **10** **5**

Compound **2b** (0.05 mmol, 0.028 g) and benzophenone (0.05 mmol, 9 mg) were dissolved in benzene-d_6 (0.6 mL) in a Young-NMR tube. After 15 hours at room temperature the resulting orange solution was identified as intermediate **10** (major species) by ^{1}H NMR (conversion of **2b**: 100%). **^{1}H NMR** (25 °C, benzene-d_6, 300.2 MHz): δ 6.91 (d, $^3J_{H,H}$ = 2.9 Hz, 1H, C*H* ebthi), 6.81 (d, $^3J_{H,H}$ = 2.8 Hz, 1H, C*H* ebthi), 6.13 (d, $^3J_{H,H}$ = 2.9 Hz, 1H, C*H* ebthi), 5.04 (d, $^3J_{H,H}$ = 2.8 Hz, 1H, C*H* ebthi), 0.62 (s, $^2J_{H,Si}$ = 7.0 Hz, 3H, C*H$_3$*), 0.50 (s, $^2J_{H,Si}$ = 7.0 Hz, 3H, C*H$_3$*), 0.42 (s, $^2J_{H,Si}$ = 6.5 Hz, 9H, SiMe$_3$), 0.13 (s, $^2J_{H,Si}$ = 6.5 Hz, 9H, SiMe$_3$). (A more precise assignment was not made, since the signals in the reaction mixture cannot be assigned without any doubt.)

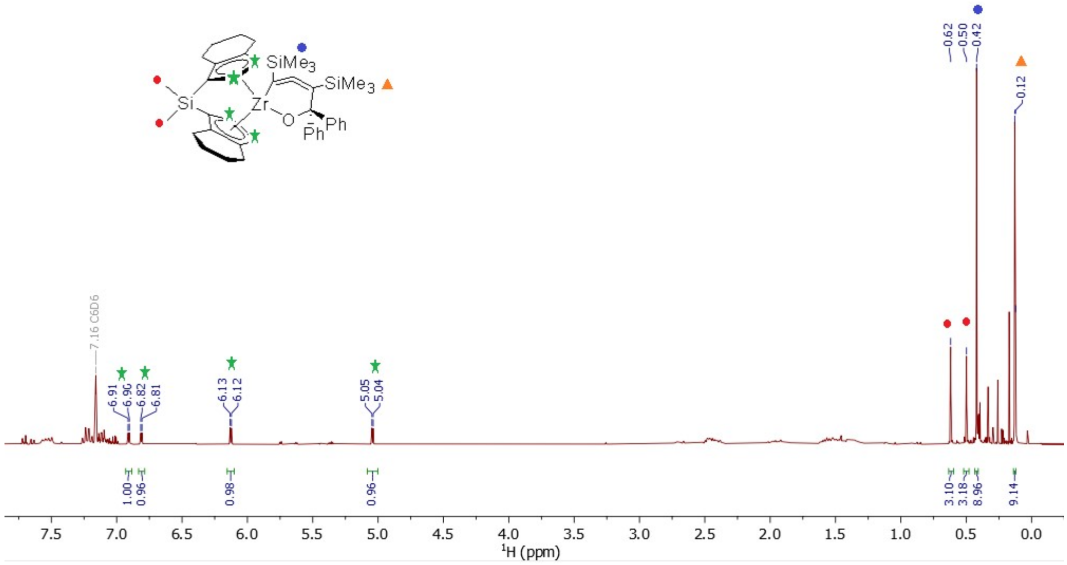

Figure S5. ^{1}H NMR spectrum of the reaction mixture for the formation of **10** (25 °C, benzene-d_6, 300.2 MHz).

After heating the above-mentioned reaction mixture at 60 °C for 1 day (or at r.t. for 3 days), the crude NMR showed that compound **5** was the resulting product, which is in agreement with the previously reported literature.[7] **^{1}H NMR** (25 °C, benzene-d_6, 300.2 MHz): δ 7.66 (m, 2H, Ph), 7.06 (m, 8H, Ph), 0.17 (s, $^2J_{H, Si}$ =7.0 Hz, 9H, SiMe$_3$), 0.12 (s, $^2J_{H, Si}$ = 6.7 Hz, 9H, SiMe$_3$).

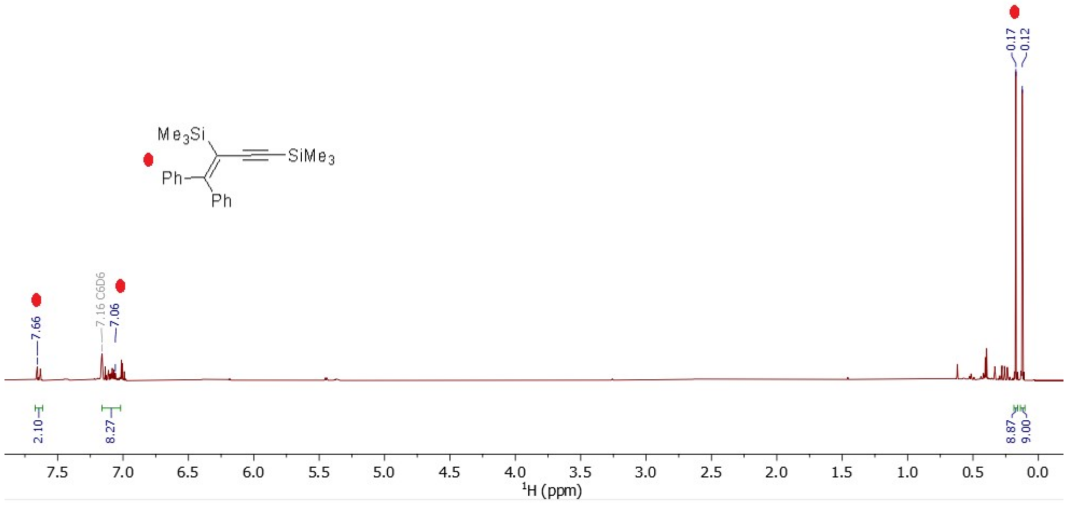

Figure S6. ^{1}H NMR spectrum of the reaction mixture for the formation of **5** (25 °C, benzene-d_6, 300.2 MHz).

3.2.2 Reaction of 2b with acetophenone to *E/Z* isomer mixture of (4-phenylpent-3-en-1-yne-1,3-diyl)bis(trimethylsilane) (7*E* and 7*Z*)

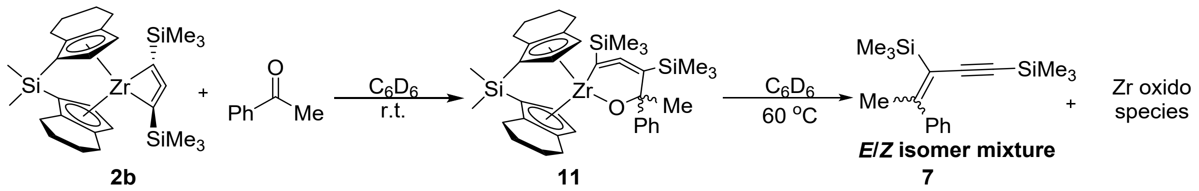

Compound **2b** (0.05 mmol, 0.028 g) was dissolved in benzene-d_6 (0.6 mL) in a Young-NMR tube and acetophenone (0.05 mmol, 6 mg) was added. A color change to orange was observed immediately after addition of acetophenone. After 15 hours at room temperature, the resulting orange solution was identified as intermediate **11** (major species) by ^{1}H NMR (conversion of **2b**: 100%). After heating the above-mentioned reaction mixture at 60 °C for 1 day, the crude NMR showed that *E* and *Z* isomers mixture of compound **7** (*E/Z* ratio 1:0.9) were the resulting products, which was in agreement with the previously reported literature.[7] ***7Z*: ^{1}H NMR** (25 °C, benzene-d_6, 300.2 MHz): δ 7.00 (m, 5H, Ph), 2.40 (s, 3H, C*H*$_3$), 0.29 (s, $^2J_{H, Si}$ =7.0 Hz, 9H, SiMe$_3$), 0.05 (s, $^2J_{H, Si}$ = 6.7 Hz, 9H, SiMe$_3$). ***7E*: ^{1}H NMR** (25 °C, benzene-d_6, 300.2 MHz): δ 7.53 (m, 2H, Ph), 7.20(m, 2H, Ph), 7.10(m, 1H, Ph), 2.03 (s, 3H, C*H*$_3$), 0.34 (s, $^2J_{H, Si}$ =6.6 Hz, 9H, SiMe$_3$), 0.11 (s, $^2J_{H, Si}$ = 7.0 Hz, 9H, SiMe$_3$).

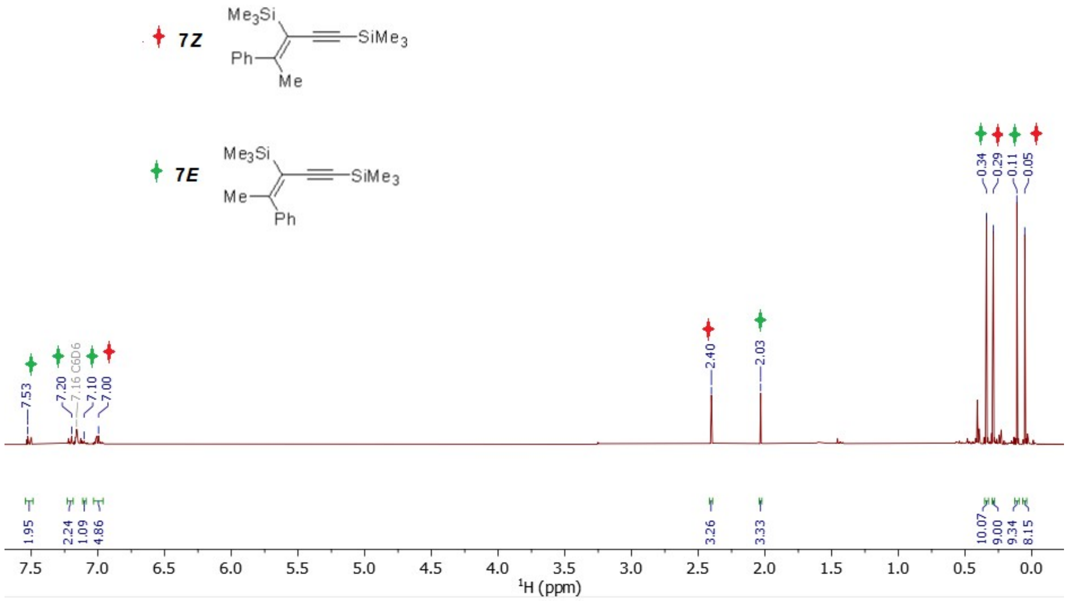

Figure S7. ^{1}H NMR spectrum of the reaction mixture for the formation of ***7E/7Z*** (25 °C, benzene-d_6, 300.2 MHz).

3.2.3 Reaction of 2b with acetone to (4-methylpent-3-en-1-yne-1,3-diyl)bis(trimethylsilane) (9)

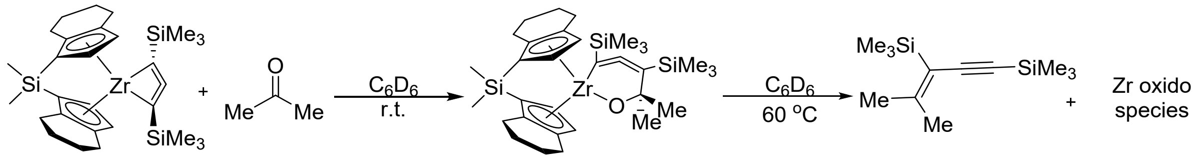

Compound **2b** (0.05 mmol, 0.028 g) was dissolved in benzene-d_6 (0.6 mL) in a Young-NMR tube and acetone (0.05 mmol, 3 mg) was added. A color change to orange was observed immediately after addition of acetone. After 15 hours at room temperature, the resulting orange solution was identified as intermediate **12** (major species) by ^{1}H NMR (conversion of **2b**: 100%). **^{1}H NMR** (25 °C, benzene-d_6, 300.2 MHz): δ 6.96 (d, $^3J_{H,H}$ = 2.8 Hz, 1H, C*H* ebthi), 6.90 (d, $^3J_{H,H}$ = 3.1 Hz, 1H, C*H* ebthi), 5.61 (d, $^3J_{H,H}$ = 3.1 Hz, 1H, C*H* ebthi), 5.07 (d, $^3J_{H,H}$ = 2.8 Hz, 1H, C*H* ebthi), 1.45 (s, 3H, C-CH_3), 1.40 (s, 3H, C-CH_3), 0.56 (s, $^2J_{H,Si}$ = 6.9 Hz, 3H, Si-CH_3), 0.49 (s, $^2J_{H,Si}$ = 6.9 Hz, 3H, Si-CH_3), 0.38 (s, $^2J_{H,Si}$ = 6.5 Hz, 9H, SiMe$_3$), 0.35 (s, $^2J_{H,Si}$ = 6.5 Hz, 9H, SiMe$_3$). (A more precise assignment was not made, since the signals in the reaction mixture cannot be assigned without any doubt.)

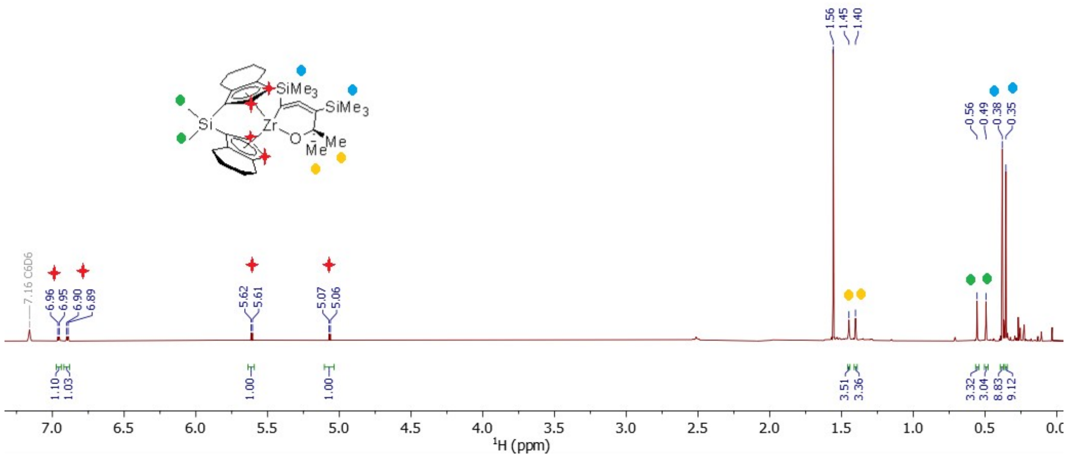

Figure S8. ^{1}H NMR spectrum of the reaction mixture for the formation of **12** (25 °C, benzene-d_6, 300.2 MHz).

After heating the above-mentioned reaction mixture at 60 °C for 2 days, the crude NMR showed that compound **9** was the resulting product, which was in agreement with the previously reported literature.[7]

^{1}H NMR (25 °C, benzene-d_6, 300.2 MHz): δ 2.03 (q, $^4J_{H,H}$ = 0.4 Hz, 3H, CH_3), 1.63 (q, $^4J_{H,H}$ = 0.4 Hz, 3H, CH_3), 0.27 (s, $^2J_{H,Si}$ =6.4 Hz, 9H, SiMe$_3$), 0.26 (s, $^2J_{H,Si}$ = 7.0 Hz, 9H, SiMe$_3$).

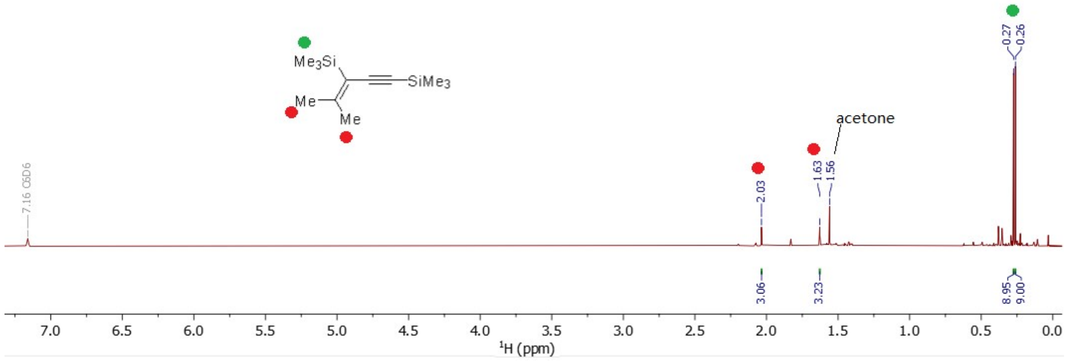

Figure S9. ^{1}H NMR spectrum of the reaction mixture for the formation of **9** (25 °C, benzene-d_6, 300.2 MHz).

3.3 Reaction of 3a with ketones

3.3.1 Reaction of 3a with benzophenone

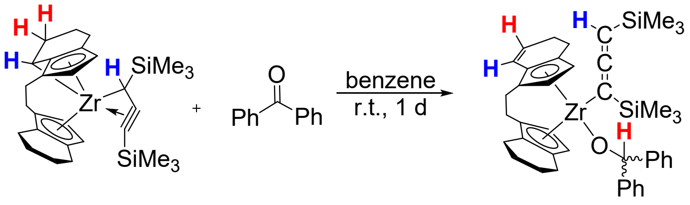

3a **13**

Compound **3a** (0.20 mmol, 0.108 g) and benzophenone (0.20 mmol, 37 mg) were dissolved in benzene (2 mL). After one day of stirring at room temperature the solvent was removed in *vacuo*. An NMR sample was taken and the crude NMR spectrum showed that **13** was the resulting products. Then the yellow residue was dissolved in hexane and stored at -30 °C for crystallization to obtain the product **13** (118 mg, 82%). Single crystals suitable for single crystal diffraction were grown from hexane.

m.p. 168-171 °C (dec. Ar.). **^{1}H NMR** (25 °C, benzene-d_6, 400.1 MHz): δ 7.43-7.38 (m, 4H, Ph), 7.22-7.18 (m, 4H, Ph), 7.11-7.03 (m, 2H, Ph), 6.49 (d, $^{3}J_{H,H}$ = 2.8 Hz, 1H, C*H* ebthi), 6.39 (s, 1H, O-C*H*), 6.38 (dd, $^{3}J_{H,H}$ = 9.8, 3.0 Hz, 1H, C*H* ebthi), 5.65-5.60 (m, 1H, C*H* ebthi), 5.49 (d, $^{3}J_{H,H}$ = 2.8 Hz, 1H, C*H* ebthi), 5.42 (d, $^{3}J_{H,H}$ = 3.0 Hz, 1H, C*H* ebthi), 5.24 (d, $^{3}J_{H,H}$ = 3.0 Hz, 1H, C*H* ebthi), 3.30 (s, $^{2}J_{H,Si}$ =4.7 Hz, 1H, C=C=C-*H*), 2.98-2.82 (m, 2H, CH_2 ebthi), 2.69-2.59 (m, 2H, CH_2 ebthi), 2.53-2.38 (m, 4H, 2 x CH_2 ebthi), 2.28-2.16 (m, 2H, CH_2 ebthi), 2.14-1.76 (m, 4H, 2 x CH_2 ebthi), 1.54-1.45 (m, 1H, CH_2 ebthi), 1.40-1.28 (m, 1H, CH_2 ebthi), 0.42 (s, $^{2}J_{H,Si}$ = 6.5 Hz, 9H, SiMe$_3$), 0.37 (s, $^{2}J_{H,Si}$ = 6.5 Hz, 9H, SiMe$_3$). **^{13}C NMR** (25 °C, benzene-d_6, 100.6 MHz): δ 194.5 (C=*C*=C), 147.0, 146.2 (2 x *C* Ph), 131.0, 129.4 (2 x *C* ebthi), 128.32, 128.26, 128.2,127.9 (CH Ph), 127.40 (*C* ebthi), 127.36 (CH Ph), 127.23 (CH ebthi), 127.16 (CH Ph), 127.1, 126.8, 122.5 (3 x *C* ebthi), 120.9, 114.6, 110.6, 109.7, 104.7 (5 x CH ebthi), 101.8 (*C*=C=C-H), 87.1 (O-CH), 52.3 (C=C=*C*-H), 28.7, 28.5, 25.6, 23.9, 23.4, 22.7, 22.3, 20.8 (8 x CH_2 ebthi), 2.2, 1.7 (2 x SiMe$_3$). **^{29}Si-inept NMR** (25 °C, benzene-d_6, 79.5 MHz): δ -5.87, -6.26. **IR** (ATR, 32 scans, cm^{-1}): 3061 (vw), 3024 (vw), 2943 (w), 2919 (w), 2892 (w), 2861 (w), 2844 (w), 1853 (s), 1492 (w), 1451 (w), 1241 (m), 1183 (w), 1088 (w), 1053 (m), 1024 (w), 919 (w), 829 (s), 791 (m), 771 (s), 742 (m), 699 (s), 684 (s), 618 (w), 602

(m), 517 (w), 482 (s), 441 (w), 404 (w). **Raman** (784 nm, 15 sec, 10 acc, cm^{-1}): 486 (s), 520 (m), 609 (m), 620 (s), 689 (m), 743 (m), 774 (m), 832 (m), 888 (w), 1002 (s), 1004 (s), 1027 (m), 1182 (w), 1284 (w), 1449 (w), 1478 (w), 1599 (w), 1615 (w), 1857 (vw), 2894 (vw), 2946 (vw). **MS-CI$^+$** (*isobutane*): [M^+] 718 (7), [$M-H^+$] 717 (9), [$M-Me_3SiCHC_2SiMe_3^+$] 533 (100), [$M-Me_3SiCHC_2SiMe_3-OCHPh_2^+$] 347 (5), [$(Me_3SiCHCCHSiMe_3+H)^+$] 185 (4), [$SiMe_3^+$] 73 (3).

3.3.2 Reaction of 3a with acetophenone

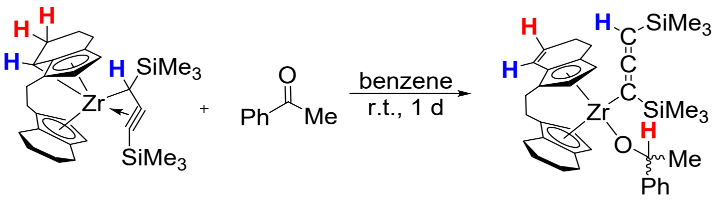

3a **14**

Compound **3a** (0.20 mmol, 0.108 g) was dissolved in benzene (2 mL) and acetophenone (0.20 mmol, 24 mg) was added. After one day of stirring at room temperature the solvent was removed in *vacuo*. An NMR sample was taken and the crude NMR spectrum showed that isomer mixture of compound **14** was the resulting products. Then the yellow residue was dissolved in hexane and stored at -30 °C for crystallization to obtain the product **14** (112 mg, 85%). Single crystals suitable for single crystal diffraction were grown from hexane.

m.p. 162-165 °C (Ar.). **^{1}H NMR** (25 °C, benzene-d_6, 400.1 MHz): δ 7.36-7.33 (m, 2H, Ph), 7.27-7.22 (m, 2H, Ph), 7.18-7.13 (m, 1H, Ph), 6.47 (d, $^3J_{H,H}$ = 2.8 Hz, 1H, C*H* ebthi), 6.41 (dd, $^3J_{H,H}$ = 9.8, 3.0 Hz, 1H, C*H* ebthi), 5.69 (m, 1H, C*H* ebthi), 5.53 (d, $^3J_{H,H}$ = 3.0 Hz, 1H, C*H* ebthi), 5.44 (d, $^3J_{H,H}$ = 2.8 Hz, 1H, C*H* ebthi), 5.28 (q, $^3J_{H,H}$ = 6.4 Hz, 1H, O-C*H*), 5.08 (d, $^3J_{H,H}$ = 2.9 Hz, 1H, C*H* ebthi), 3.30 (s, $^2J_{H,Si}$ =4.7 Hz, 1H, C=C=C-*H*), 3.02-2.93 (m, 1H, CH_2 ebthi), 2.89-2.82 (m, 1H, CH_2 ebthi), 2.71-2.40 (m, 6H, CH_2 ebthi), 2.37-2.30 (m, 1H, CH_2 ebthi), 2.25-1.94 (m, 4H, CH_2 ebthi), 1.85-1.78 (m, 1H, CH_2 ebthi), 1.52-1.46 (m, 1H, CH_2 ebthi), 1.46 (d, $^3J_{H,H}$ = 6.4 Hz, 3H, CH_3), 1.42-1.32 (m, 1H, CH_2 ebthi), 0.43 (s, $^2J_{H,Si}$ = 6.5 Hz, 9H, $SiMe_3$), 0.39 (s, $^2J_{H,Si}$ = 6.5 Hz, 9H, $SiMe_3$). **^{13}C NMR** (25 °C, benzene-d_6, 100.6 MHz): δ 195.5 (C=*C*=C), 148.2 (*C* Ph), 130.4, 129.1 (2 x *C* ebthi), 128.43, 128.40, 127.3 (3 x CH Ph), 126.6 (CH ebthi), 126.3, 125.9, 122.5 (3 x *C* ebthi), 121.5 (CH ebthi), 116.6 (*C* ebthi), 115.4, 109.9, 109.8, 104.7 (4 x *C*H ebthi), 101.1 (*C*=C=C-H), 81.8 (O-*C*H), 51.9 (C=C=*C*-H), 28.6, 28.5, 28.2, 25.5, 24.1, 23.3, 22.9, 22.3, 20.9 (CH_3 + 8 x CH_2 ebthi), 1.9, 1.7 (2 x $SiMe_3$). **^{29}Si-inept NMR** (25 °C, benzene-d_6, 59.6 MHz): δ -5.99, -6.64. **IR** (ATR, 32 scans, cm^{-1}): 3024 (vw), 2923 (m), 2871 (w), 2851 (w), 1857 (m), 1814 (w), 1492 (w), 1455 (w), 1437 (w), 1364 (w), 1317 (w), 1259 (w), 1239 (m), 1210 (w), 1101 (w), 1078 (s), 1031 (w), 1016 (m), 932 (m), 824 (s), 794 (s), 761 (s), 699 (s), 680 (s), 633 (m), 616 (m), 573 (m), 538 (m), 515 (m), 484 (s), 472 (m), 437 (w), 402 (m). **Raman** (784 nm, 30 sec, 15 acc, cm^{-1}): 427 (m), 484 (s), 518 (m), 620 (s), 677 (m), 774 (m), 862 (m), 934 (w), 1000 (s), 1030 (m), 1080 (w), 1155 (w), 1210 (w), 1277 (w), 1331 (w), 1455 (m), 1470 (m), 1615 (m), 1859 (vw), 2897 (vw). **MS-CI$^+$** (*isobutane*): [M+*isobutane*$^+$] 711 (2), [M^+] 656 (3), [$M-Me_3SiCHC_2SiMe_3^+$] 471 (100), [$M-Me_3SiCHC_2SiMe_3-OCHPhMe^+$] 347 (4).

3.3.3 Reaction of 3a with acetone

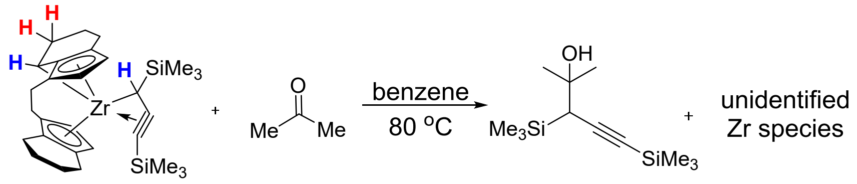

3a **15**

Compound **3a** (0.20 mmol, 0.108 g) was dissolved in benzene (2 mL) and acetone (0.40 mmol, 24 mg) was added. After 2 days of stirring at 80 °C, Silica Gel was added. After removing the solvent, the product **15** was separated by column chromatography (hexane/ethyl acetate 20:1) as colorless liquid (30 mg, 62%).

^{1}H NMR (25 °C, benzene-d_6, 300.2 MHz): δ 1.97 (s, $^2J_{H, Si}$ = 9.3 Hz, 1H, C*H*), 1.52 (s, 1H, O*H*), 1.31 (s, 3H, C*H*$_3$), 1.28 (s, 3H, C*H*$_3$), 0.22 (s, $^2J_{H, Si}$ = 6.6 Hz, 9H, SiMe$_3$), 0.18 (s, $^2J_{H, Si}$ = 7.0 Hz, 9H, SiMe$_3$). **^{13}C NMR** (25 °C, benzene-d_6, 75.4 MHz): δ 108.4, 88.1, 72.5 (*C*-Me$_2$OH), 37.8 (C≡C-*C*), 30.9, 29.6 (2 x CH$_3$), 0.4, -0.5 (2 x SiMe$_3$). **^{13}C DEPT-135 NMR** (25 °C, benzene-d_6, 75.4 MHz): δ 37.8, 30.9, 29.6, 0.4, -0.5. **^{29}Si-inept NMR** (25 °C, benzene-d_6, 79.5 MHz): δ 2.00, -19.73. **IR** (ATR, 32 scans, cm^{-1}): 3463 (w), 2956 (w), 2925 (w), 2855 (w), 2156 (w), 1674 (vw), 1459 (w), 1371 (w), 1340 (w), 1249 (m), 1140 (w), 1084 (w), 1051 (w), 952 (w), 839 (s), 759 (m), 695 (w), 672 (w), 653 (w), 620 (w), 532 (w), 410 (w). **Raman** (784 nm, 15 sec, 10 acc, cm^{-1}): 467 (m), 487 (m), 536 (m), 620 (s), 652 (m), 695 (m), 847 (m), 1170 (m), 1253 (m), 1385 (m), 1411 (m), 1431 (m), 1591 (m), 1652 (m), 2160 (w), 2901 (w), 2966 (w). **MS-EI$^+$**: [M-Me$^+$] 227 (2), [M-CMe$_2$OH$^+$] 186 (18), [M-TMS$^+$] 169 (25), [M-OH-TMS$^+$] 152 (35), [M-C$_2$TMS$^+$] 147 (50), [M-OH-Me-TMS$^+$] 137 (100), [SiMe$_3^+$] 73 (71), [CMe$_2$OH$^+$] 59 (17).

3.4 Reaction of 3b with ketones

3.4.1 Reaction of 3b with benzophenone

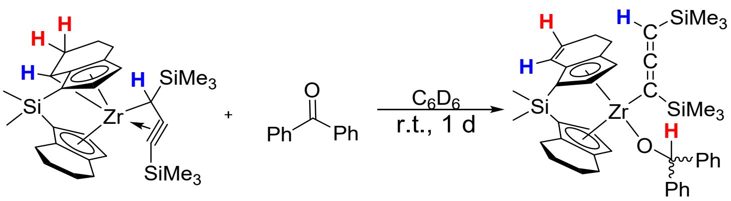

3b **16**

Compound **3b** (0.05 mmol, 0.028 g) and benzophenone (0.05 mmol, 9 mg) were dissolved in benzene-d_6 (0.6 mL) in a Young-NMR tube. After 24 hours at room temperature the resulting yellow solution was identified as product **16** (major species) by ^{1}H NMR and 2D ^{1}H-^{1}H COSY NMR spectrum (conversion of **3b**: 100%). **^{1}H NMR** (25 °C, benzene-d_6, 300.2 MHz): δ 7.45-7.41 (m, 4H, Ph), 6.84 (d, $^3J_{H, H}$ = 2.7 Hz, 1H, C*H* ebthi), 6.48 (dd, $^3J_{H, H}$ = 9.8, 3.0 Hz, 1H, C*H* ebthi), 6.39 (s, 1H, O-C*H*), 5.70-5.64 (m, 1H, C*H* ebthi), 5.63 (d, $^3J_{H, H}$ = 3.0 Hz, 1H, C*H* ebthi), 5.37-5.35

(m, 2H, 2 x C*H* ebthi), 3.25 (s, $^{2}J_{H, Si}$ =4.7 Hz, 1H, C=C=C-*H*), 2.89-2.74 (m, 2H, C*H*$_2$ ebthi), 2.66-2.56 (m, 1H, C*H*$_2$ ebthi), 2.50-2.27 (m, 3H, C*H*$_2$ ebthi), 2.15-1.70 (m, 4H, 2 x C*H*$_2$ ebthi), 1.49-1.33 (m, 2H, C*H*$_2$ ebthi), 0.51 (s, $^{2}J_{H, Si}$ = 6.9 Hz, 3H, Si-C*H*$_3$), 0.41 (s, $^{2}J_{H, Si}$ = 6.9 Hz, 3H, Si-C*H*$_3$), 0.40 (s, $^{2}J_{H, Si}$ = 6.5 Hz, 9H, SiMe$_3$), 0.39 (s, $^{2}J_{H, Si}$ = 6.5 Hz, 9H, SiMe$_3$) (A more precise assignment was not made, since the signals in the reaction mixture cannot be assigned without any doubt).

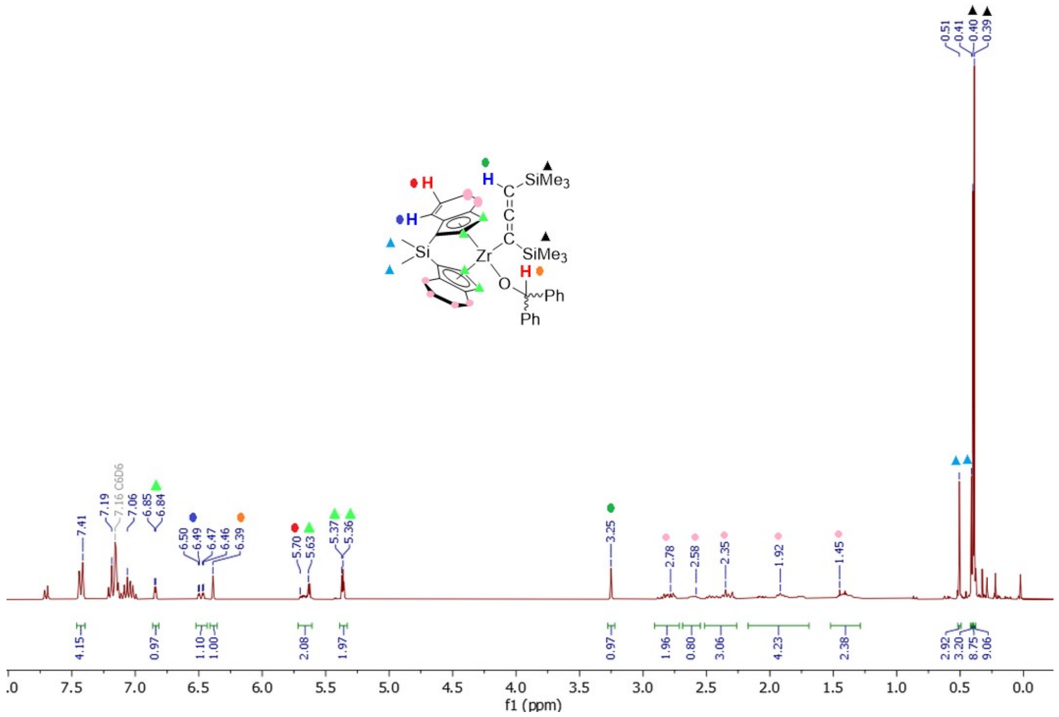

Figure S10. ^{1}H NMR spectrum of the reaction mixture for the formation of **16** (25 °C, benzene-*d*$_6$, 300.2 MHz).

3.4.2 Reaction of 3b with acetophenone

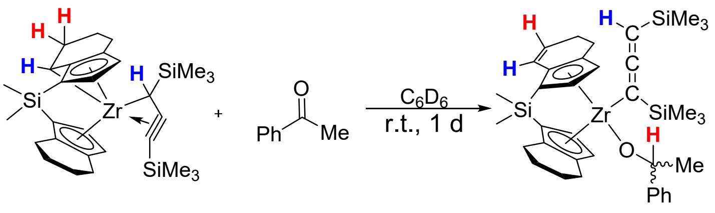

3b **17**

Compound **3b** (0.05 mmol, 0.028 g) was dissolved in benzene-*d*$_6$ (0.6 mL) in a Young-NMR tube and acetophenone (0.05 mmol, 6 mg) was added. After 24 hours at room temperature the resulting yellow solution was identified as product **17** (major species) by ^{1}H NMR and 2D ^{1}H-^{1}H COSY NMR spectrum (conversion of **3b**: 100%). **^{1}H NMR** (25 °C, benzene-*d*$_6$, 300.2 MHz): δ 7.39-7.29 (m, 3H, Ph), 7.26-7.19 (m, 2H, Ph), 6.84 (d, $^{3}J_{H, H}$ = 2.7 Hz, 1H, C*H* ebthi), 6.48 (dd, $^{3}J_{H, H}$ = 9.8, 2.9 Hz, 1H, C*H* ebthi), 5.88 (d, $^{3}J_{H, H}$ = 3.0 Hz, 1H, C*H* ebthi), 5.69-5.63 (m, 1H, C*H* ebthi), 5.36 (d, $^{3}J_{H, H}$ = 2.7 Hz, 1H, C*H* ebthi), 5.25 (q, $^{3}J_{H, H}$ = 6.4 Hz, 1H, O-C*H*), 5.21 (d, $^{3}J_{H, H}$ = 3.0 Hz, 1H, C*H* ebthi), 3.25 (s, $^{2}J_{H, Si}$ =4.7 Hz, 1H, C=C=C-*H*), 1.45 (d, $^{3}J_{H, H}$ = 6.3 Hz, 3H, C*H*$_3$), 0.48 (s, $^{2}J_{H, Si}$ = 6.9 Hz, 3H, Si-C*H*$_3$), 0.42 (s, $^{2}J_{H, Si}$ = 6.9 Hz, 3H, Si-C*H*$_3$), 0.41 (s, $^{2}J_{H, Si}$ = 6.5 Hz, 18H, 2 x SiMe$_3$)

(A more precise assignment was not made, since the signals in the reaction mixture cannot be assigned without any doubt).

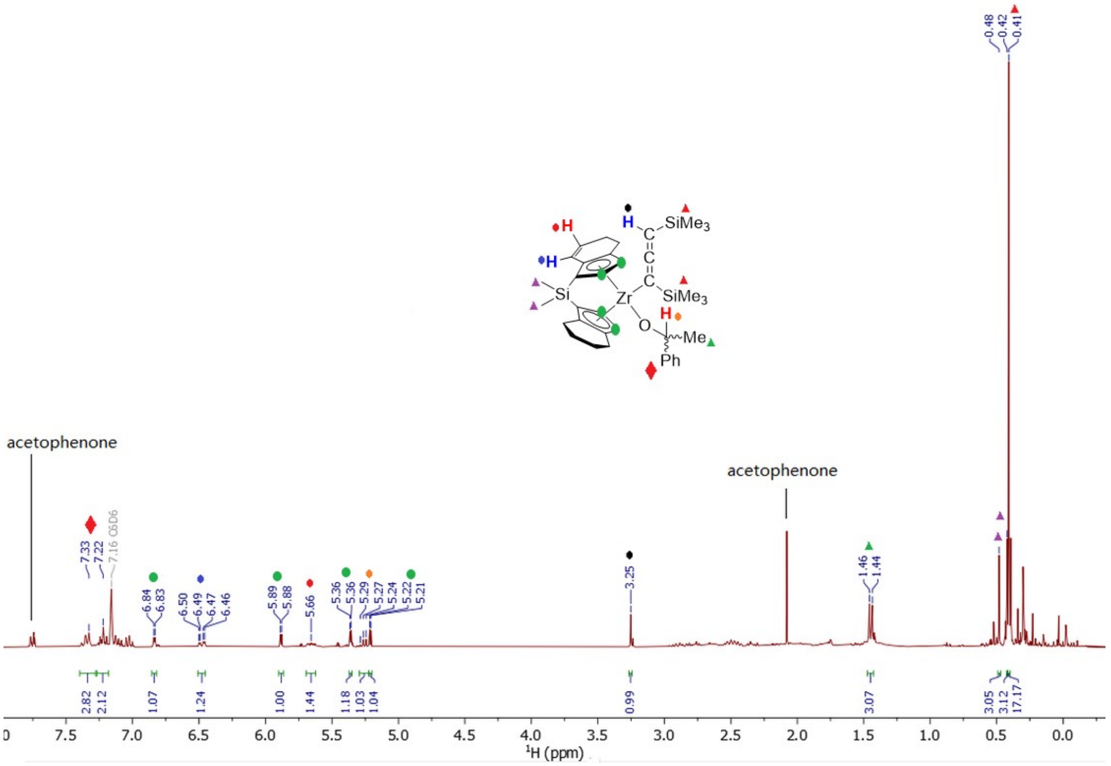

Figure S11. ^1H NMR spectrum of the reaction mixture for the formation of **17** (25 °C, benzene-d_6, 300.2 MHz).

3.4.3 Reaction of 3b with acetone

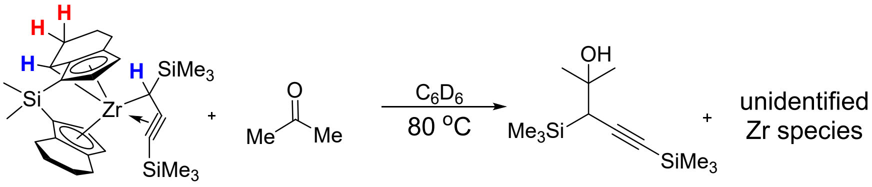

Compound **3b** (0.05 mmol, 0.028 g) was dissolved in benzene-d_6 (0.6 mL) in a Young-NMR tube and acetone (0.10 mmol, 6 mg) was added. After heating the reaction mixture at 80 °C for 2 days, the crude NMR showed that compound **15** was the resulting product (conversion of **3b**: 100%). **^{1}H NMR** (25 °C, benzene-d_6, 400.1 MHz): δ 1.97 (s, $^2J_{H,\,Si}$ = 9.3 Hz, 1H, C*H*), 1.32 (s, 3H, C*H*$_3$), 1.29 (s, 3H, C*H*$_3$), 0.22 (s, $^2J_{H,\,Si}$ = 6.6 Hz, 9H, SiMe$_3$), 0.18 (s, $^2J_{H,\,Si}$ = 7.0 Hz, 9H, SiMe$_3$). (The H atom on the OH group has no peak in this crude solution.)

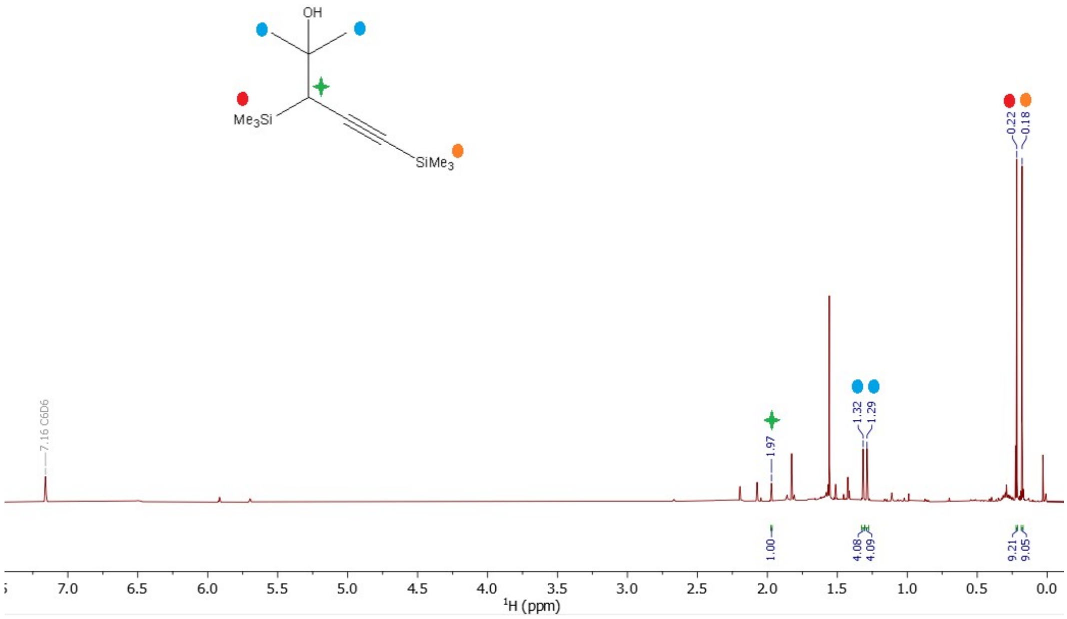

Figure S12. ^{1}H NMR spectrum of the reaction mixture for the formation of **15** (25 °C, benzene-d_6, 400.1 MHz).

4. Proof of Stability of Compounds

4.1 Stability of 2a

4.1.1 Stability of 2a in benzene at room temperature

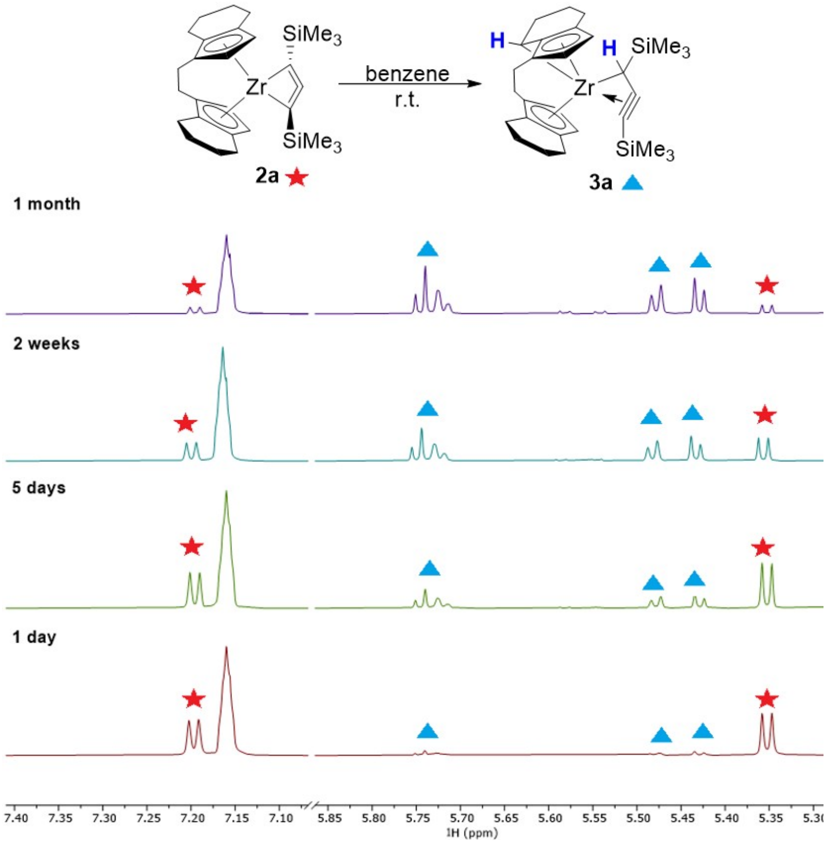

Figure S13. Comparison of ^{1}H NMR spectra of **2a** in benzene-d_6 at r.t. (25 °C, benzene-d_6, 300.2 MHz).

4.1.2 Decomposition of 2a in air and water

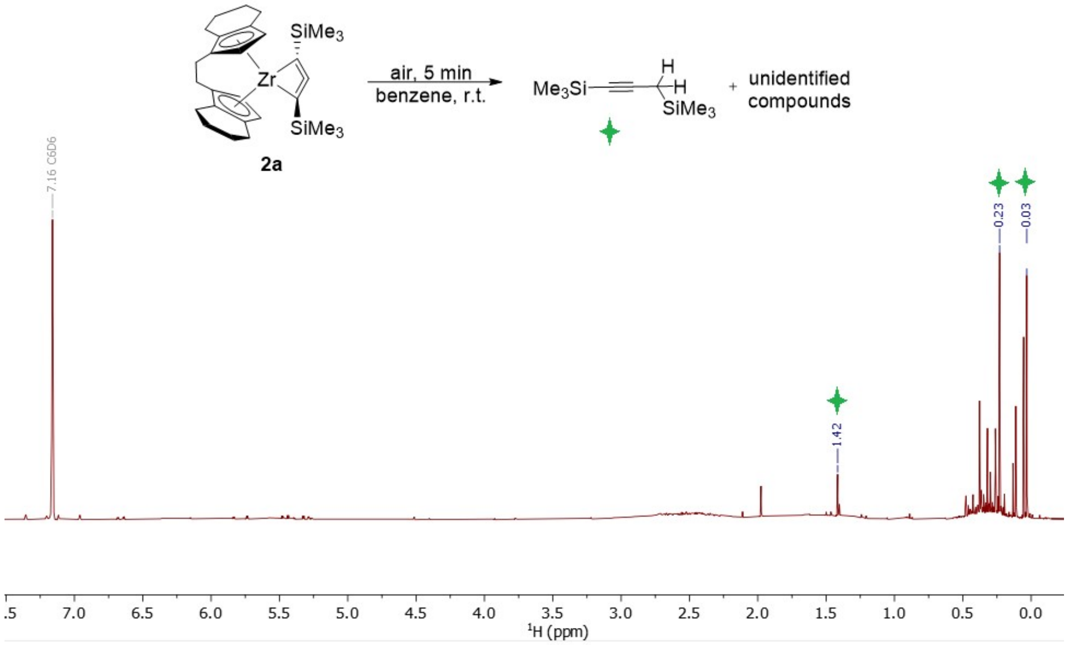

Figure S14. ^{1}H NMR spectrum of **2a** in benzene-d_6 after exposure to air for 5 min (25 °C, benzene-d_6, 400.1 MHz).

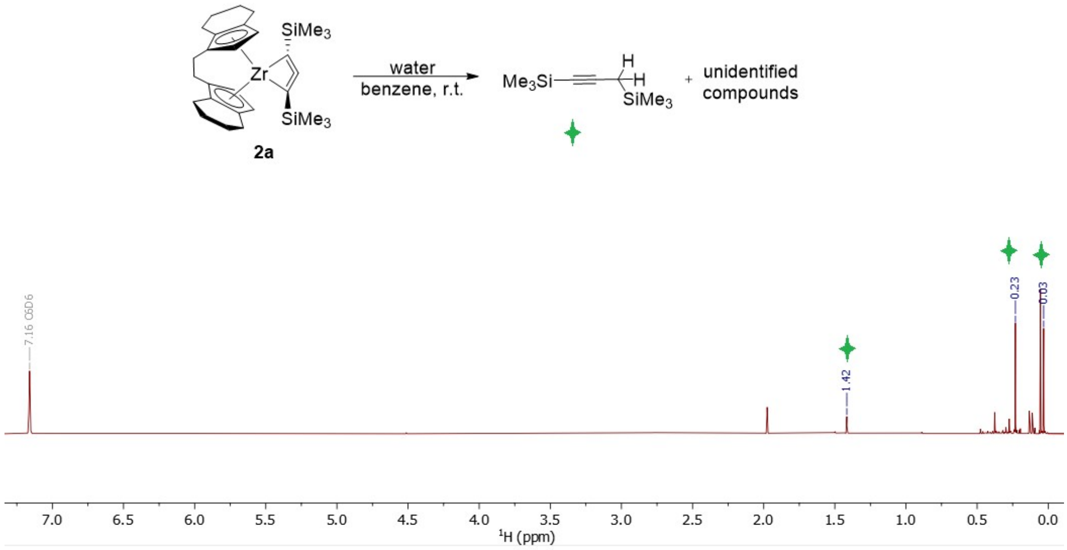

Figure S15. ^{1}H NMR spectrum of **2a** in benzene-d_6 after exposure to water (25 °C, benzene-d_6, 400.1 MHz).

4.2 Stability of 3a

4.2.1 Stability of 3a in benzene at room temperature

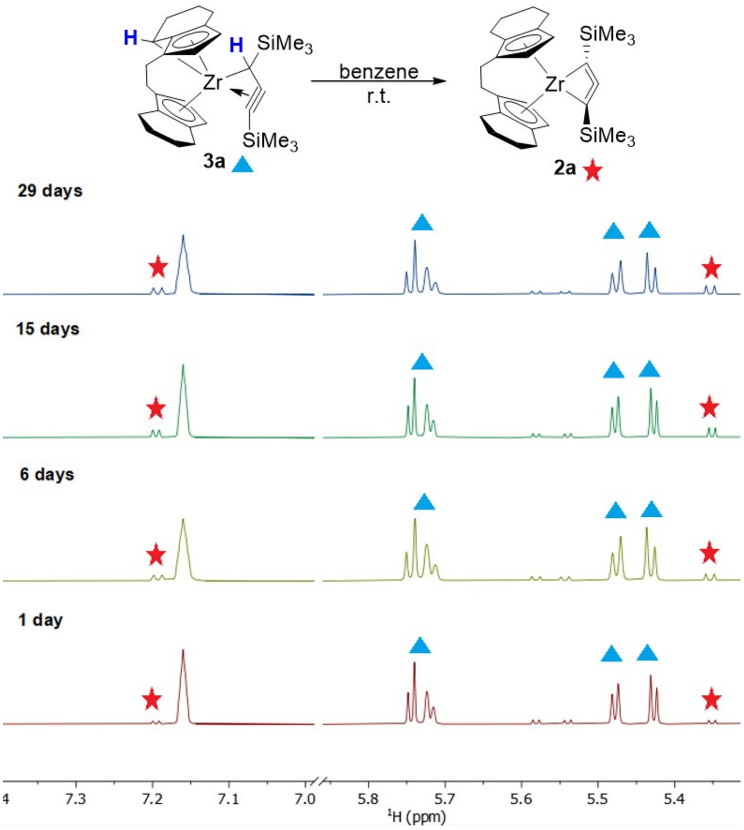

Figure S16. ^{1}H NMR spectra of **3a** in benzene-d_6 at r.t. (25 °C, benzene-d_6, 300.2 MHz).

	G_{tot}	$\Delta_R G$ [kJ/mol]	q_i	% Pop
rac-(ebthi)Zr($Me_3SiCCCSiMe_3$)	-1756.669563	0.000	1.0000	58
rac-(ebthi)*Zr($Me_3SiCHCCSiMe_3$)iso1	-1756.669849	-0.751	0.7185	42
		Summ	1.7185	

4.2.2 Decomposition of 3a in air and water

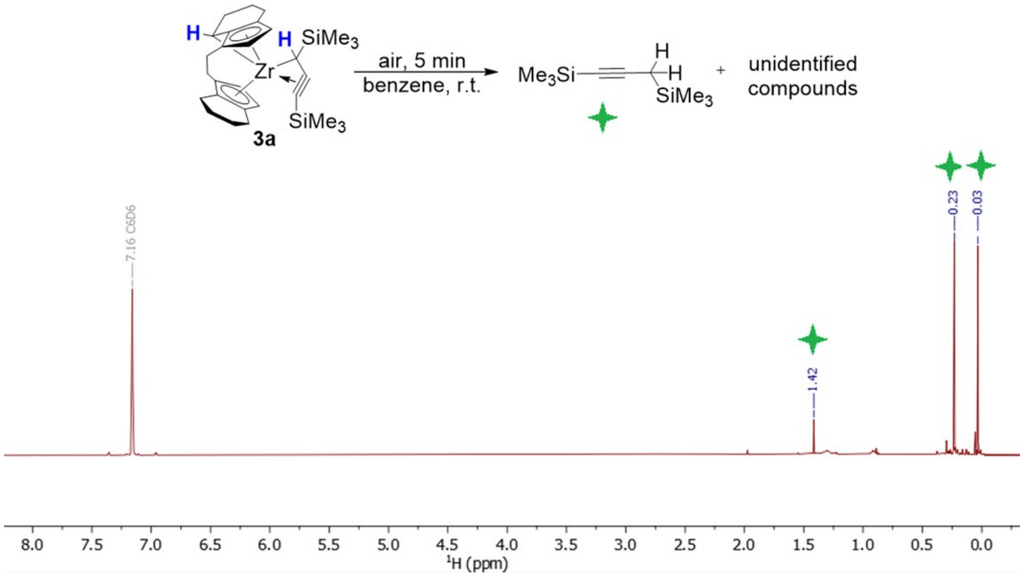

Figure S17. ^{1}H NMR spectrum of **3a** in benzene-d_6 after exposure to air for 5 min (25 °C, benzene-d_6, 400.1 MHz).

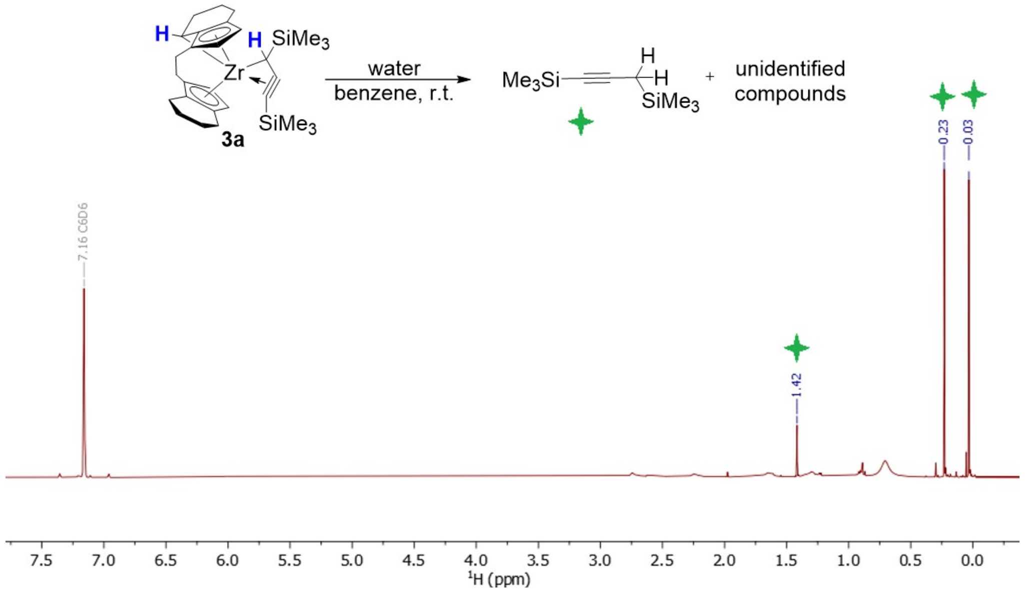

Figure S18. ^{1}H NMR spectrum of **3a** in benzene-d_6 after exposure to water (25 °C, benzene-d_6, 400.1 MHz).

4.3 Stability of 2b in benzene at room temperature

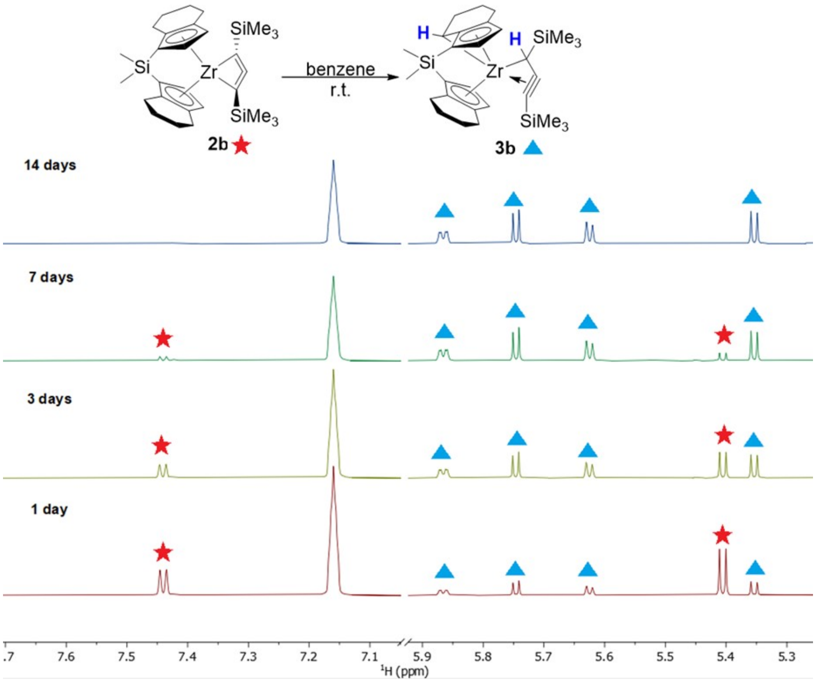

Figure S19. Comparison of ^{1}H NMR spectra of **2b** in benzene-d_6 at r.t. (25 °C, benzene-d_6, 300.2 MHz).

4.4 Stability of 3b in benzene at room temperature

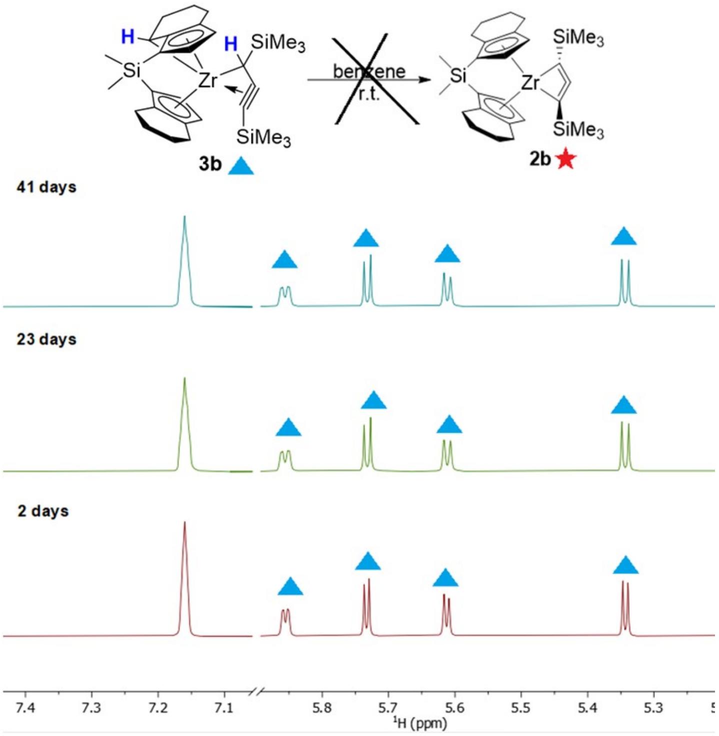

Figure S20. Comparison of ^{1}H NMR spectra of **3b** in benzene-d_6 at r.t. (25 °C, benzene-d_6, 300.2 MHz).

4.5 Stability of **A** in benzene at 60 °C

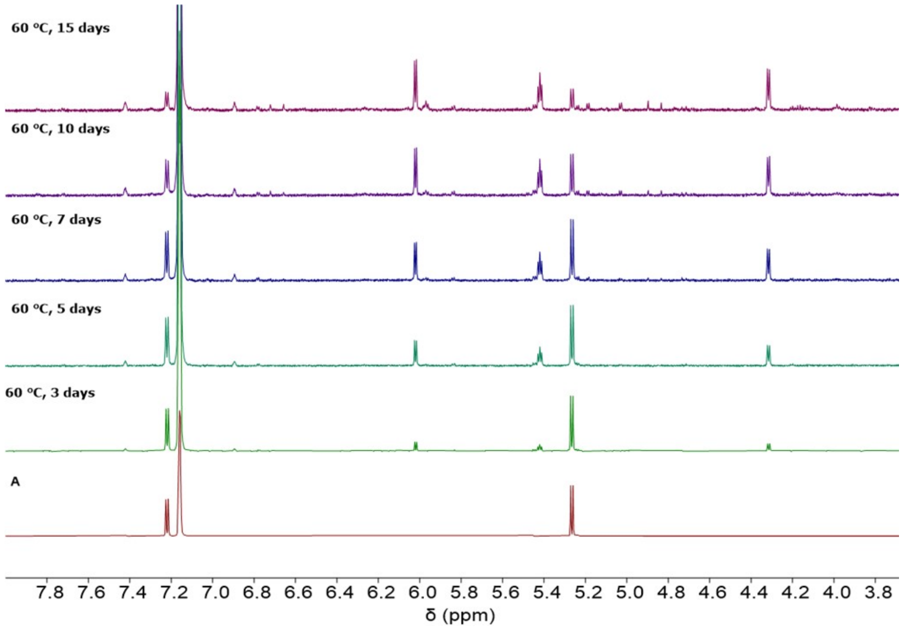

Figure S21. Comparison of ^{1}H NMR spectra of 1-titanacyclobuta-2,3-diene **A** in benzene-d_6 at 60 °C (decay of NMR intensity and degradation of **A** only after two weeks; 25 °C, benzene-d_6, 300.2 MHz).

5. Crystallographic Details

Table S1. Crystallographic details of **2a**, **2b**, **3a**, **3b**, and **19**.

Compound	**2a**	**2b**	**3a**	**3b**	**19**
Chem. Formula	$C_{29}H_{42}Si_2Zr$	$C_{29}H_{44}Si_3Zr \cdot {}^{1}/_{4}\, C_5H_{12}$	$C_{29}H_{42}Si_2Zr \cdot {}^{3}/_{4}\, C_6H_6$	$C_{29}H_{44}Si_3Zr \cdot {}^{1}/_{2}\, C_6H_{14}$	$C_{49}H_{66}Cl_2Si_2Zr_2 \cdot {}^{1}/_{2}\, C_6H_{14}$
Formula weight [g/mol]	538.02	586.17	547.79	611.21	1007.62
Color	green	yellow	yellow	yellow	yellow
Crystal system	monoclinic	monoclinic	orthorhombic	monoclinic	triclinic
Space group	$P2_1/n$	$P2_1/c$	*Pccn*	$P2_1/c$	$P\bar{1}$
a [Å]	14.3036(3)	9.6581(2)	17.3118(4)	9.4349(15)	9.6289(2)
b [Å]	10.9418(3)	11.2067(2)	34.8306(7)	11.3631(18)	13.5337(3)
c [Å]	18.2871(4)	30.3700(6)	9.5723(2)	30.084(5)	19.3253(5)
α [°]	90	90	90	90	81.3716(9)
β [°]	97.510(2)	96.3408(10)	90	95.027(3)	83.9610(9)
γ [°]	90	90	90	90	74.9222(9)
V [Å^3]	2837.51(12)	3267.00(11)	5771.9(2)	3212.9(9)	2398.37(10)
Z	4	4	8	4	2
$\rho_{calcd.}$ [g/cm^3]	1.259	1.192	1.261	1.264	1.395
μ [mm^{-1}]	0.486	3.913	0.479	0.473	5.315
T [K]	140(2)	150(2)	150(2)	150(2)	150(2)
Measured reflections	44563	34248	57300	85017	34323
Independent reflections	7197	5772	6969	7760	8469
Reflections with $I > 2\sigma(I)$	6178	5341	6046	7085	7952
R_{int}	0.0178	0.0360	0.0318	0.0325	0.0296
$F(000)$	1136	1242	2314	1300	1054
R_1 [$I > 2\sigma(I)$]	0.0226	0.0467	0.0322	0.0476	0.0255
wR_2 (all data)	0.0616	0.1263	0.0836	0.1152	0.0665
GooF	1.001	1.106	1.043	1.029	1.026
No. of Parameters	314	352	348	360	605
CCDC	2113148	2113149	2113150	2113151	2113156

Table S2. Crystallographic details of **4**, **6**, **13**, **14**, and **20**.

Compound	**4**	**6**	**13**	**14**	**20**
Chem. Formula	$C_{42}H_{52}OSi_2Zr$	$C_{37}H_{50}OSi_2Zr$	$C_{42}H_{52}OSi_2Zr$	$C_{37}H_{50}OSi_2Zr$	$C_{33}H_{46}Cl_2Si_4Zr_2 \cdot C_7H_8$
Formula weight [g/mol]	720.23	658.17	720.23	658.17	900.53
Color	yellow	yellow	yellow	yellow	orange
Crystal system	monoclinic	monoclinic	monoclinic	triclinic	monoclinic
Space group	$P2_1/c$	$P2_1/n$	$P2_1/n$	$P\bar{1}$	$C2/c$
a [Å]	17.1284(12)	10.3283(4)	10.9612(2)	8.9199(7)	41.3372(10)
b [Å]	18.2898(12)	30.8309(13)	20.0342(4)	13.4195(10)	10.2403(3)
c [Å]	11.8291(8)	11.1841(5)	17.3010(4)	15.0202(11)	22.8711(6)
α [°]	90	90	90	100.6924(17)	90
β [°]	93.8968(15)	109.3362(13)	98.6571(9)	99.1577(16)	117.4411(8)
γ [°]	90	90	90	97.8646(16)	90
V [Å^3]	3697.2(4)	3360.5(2)	3755.99(13)	1718.4(2)	8592.1(4)
Z	4	4	4	2	8
$\rho_{calcd.}$ [g/cm^3]	1.294	1.301	1.274	1.272	1.392
μ [mm^{-1}]	0.394	0.426	3.233	0.417	0.748
T [K]	150(2)	150(2)	150(2)	150(2)	150(2)
Measured reflections	76139	66738	39733	76625	54901
Independent reflections	10002	9180	6632	9228	11192
Reflections with $I > 2\sigma(I)$	7809	8457	6159	8663	9916
R_{int}	0.0438	0.0257	0.0380	0.0205	0.0204
F(000)	1520	1392	1520	696	3712
R_1 [$I > 2\sigma(I)$]	0.0387	0.0286	0.0285	0.0248	0.0257
wR_2 (all data)	0.0999	0.0717	0.0732	0.0640	0.0633
GooF	1.014	1.074	1.034	1.063	1.054
No. of Parameters	519	377	574	520	444
CCDC	2113152	2113153	2113154	2113155	2113157

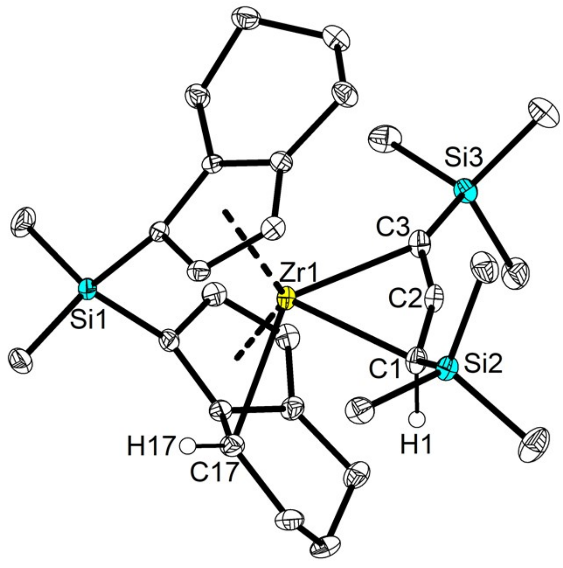

Figure S22. Molecular structure of complex **3b**. Thermal ellipsoids correspond to 30% probability. Hydrogen atoms (except H1 and H17) are omitted for clarity.

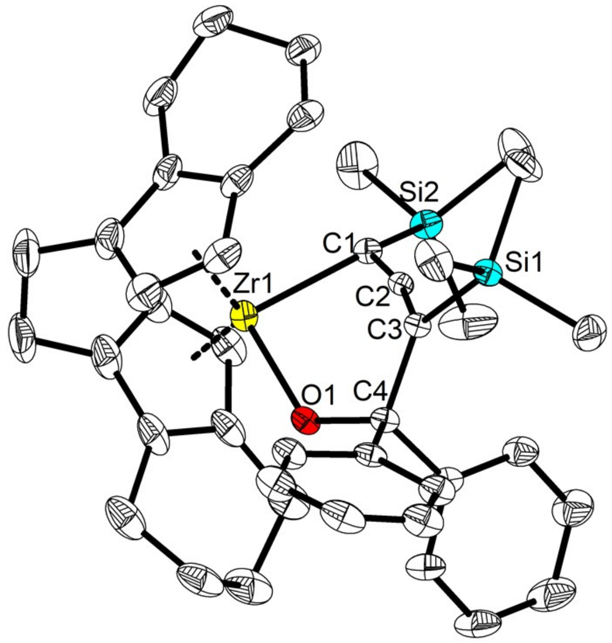

Figure S23. Molecular structure of complex **4**. Thermal ellipsoids correspond to 30% probability. Hydrogen atoms and the second position of the disordered group are omitted for clarity.

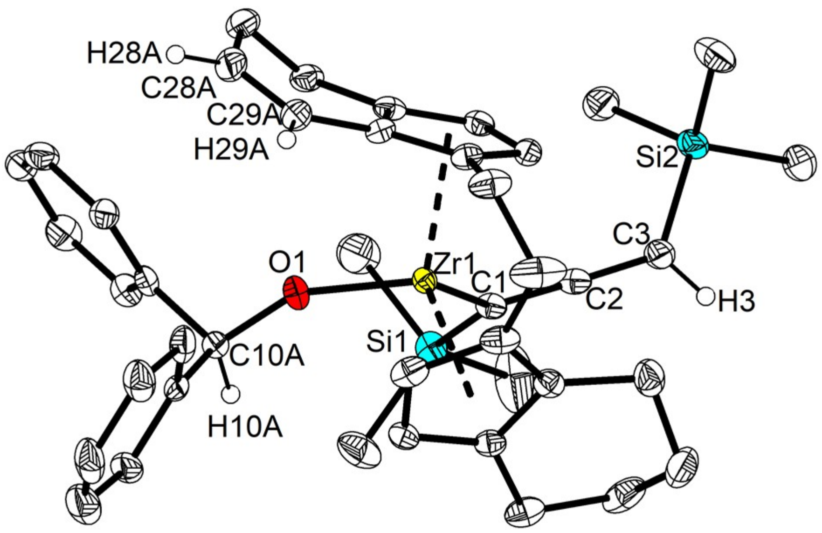

Figure S24. Molecular structure of complex **13**. Thermal ellipsoids correspond to 30% probability. Hydrogen atoms (except H3, H10A, H28A and H29A) and the second position of the disordered group are omitted for clarity.

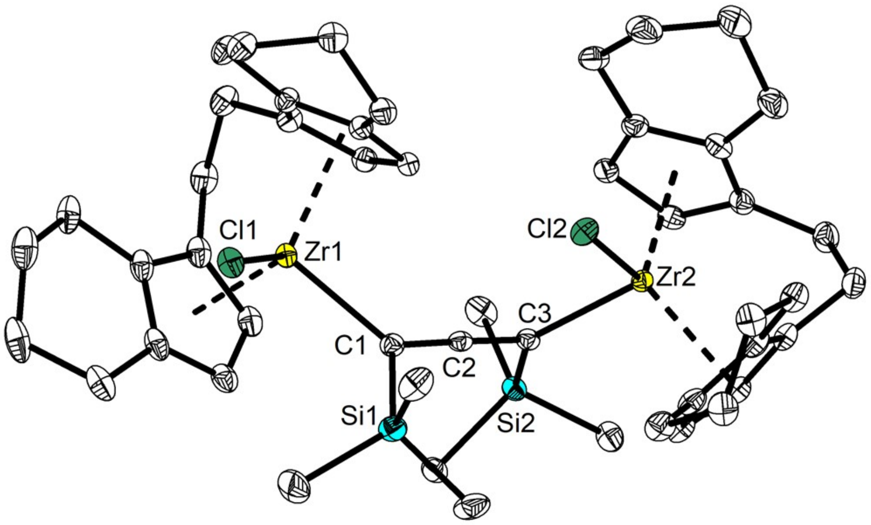

Figure S25. Molecular structure of complex **19**. Thermal ellipsoids correspond to 30% probability. Hydrogen atoms and the second position of the disordered indenyl group are omitted for clarity.

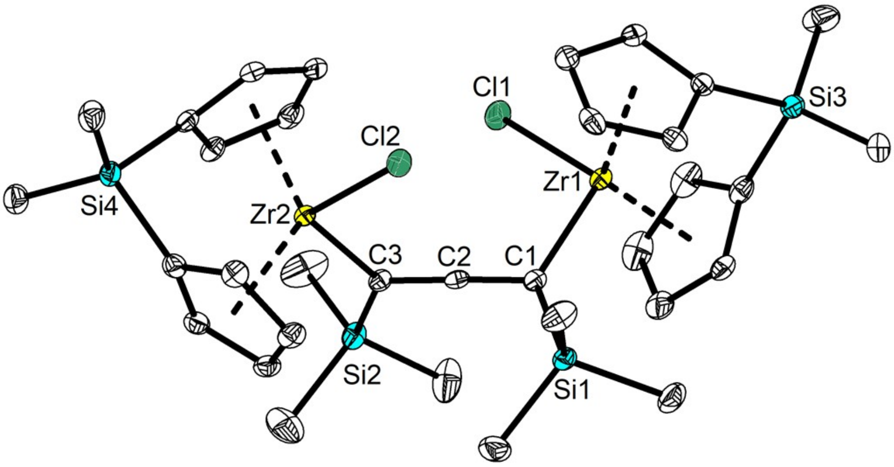

Figure S26. Molecular structure of complex **20**. Thermal ellipsoids correspond to 30% probability. Hydrogen atoms are omitted for clarity.

6. Details of NMR Spectroscopy

6.1 NMR spectra of 2a

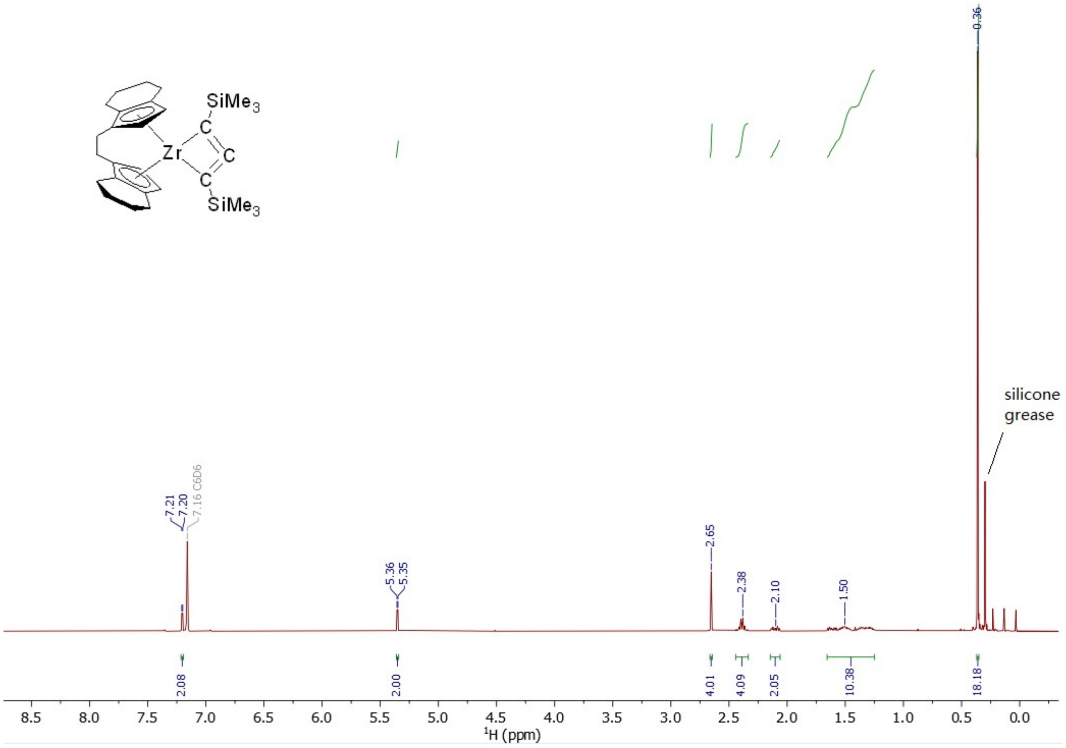

Figure S27. ^{1}H NMR spectrum of **2a** (25 °C, benzene-d_6, 400.1 MHz).

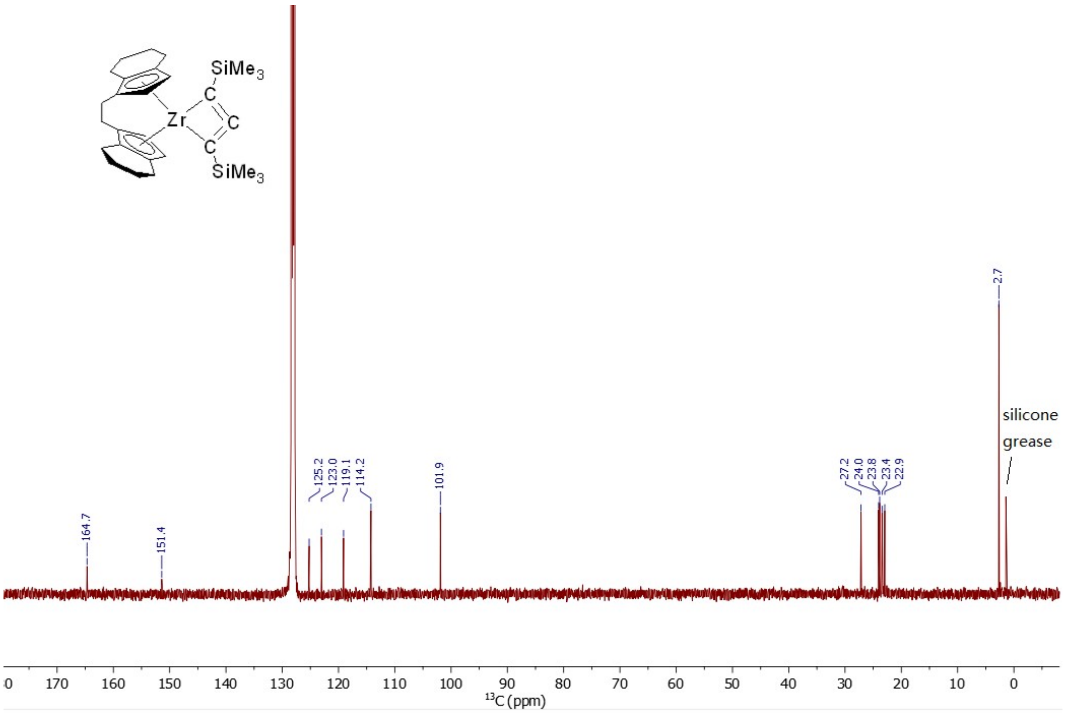

Figure S28. ^{13}C NMR spectrum of **2a** (25 °C, benzene-d_6, 100.6 MHz).

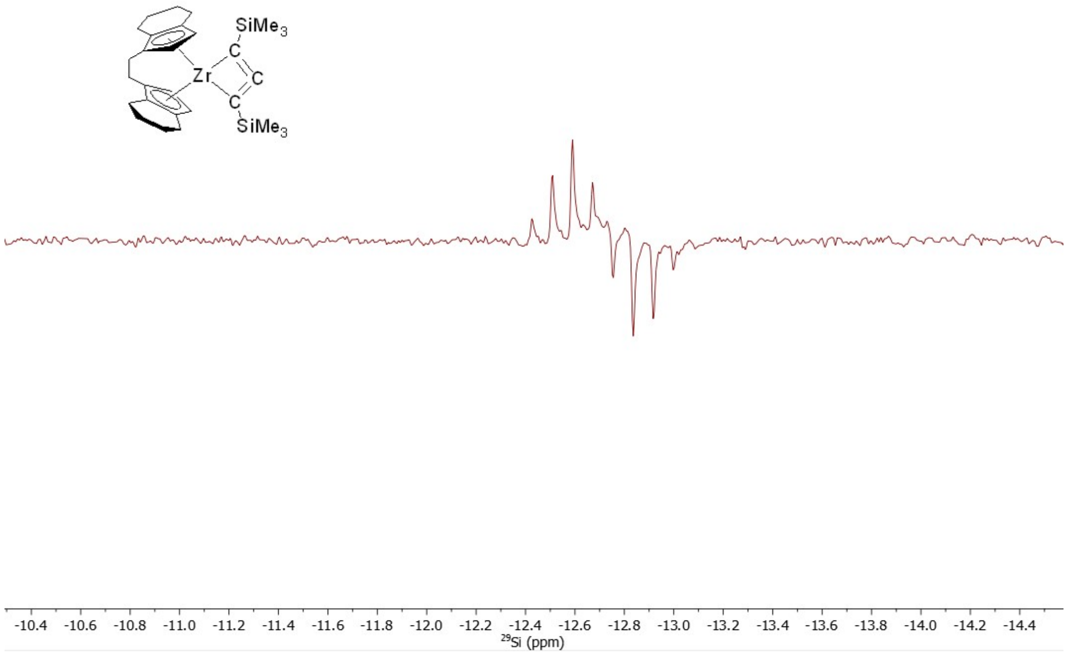

Figure S29. ^{29}Si INEPT NMR spectrum of **2a** (25 °C, benzene-d_6, 79.5 MHz).

6.2 NMR spectra of 2b

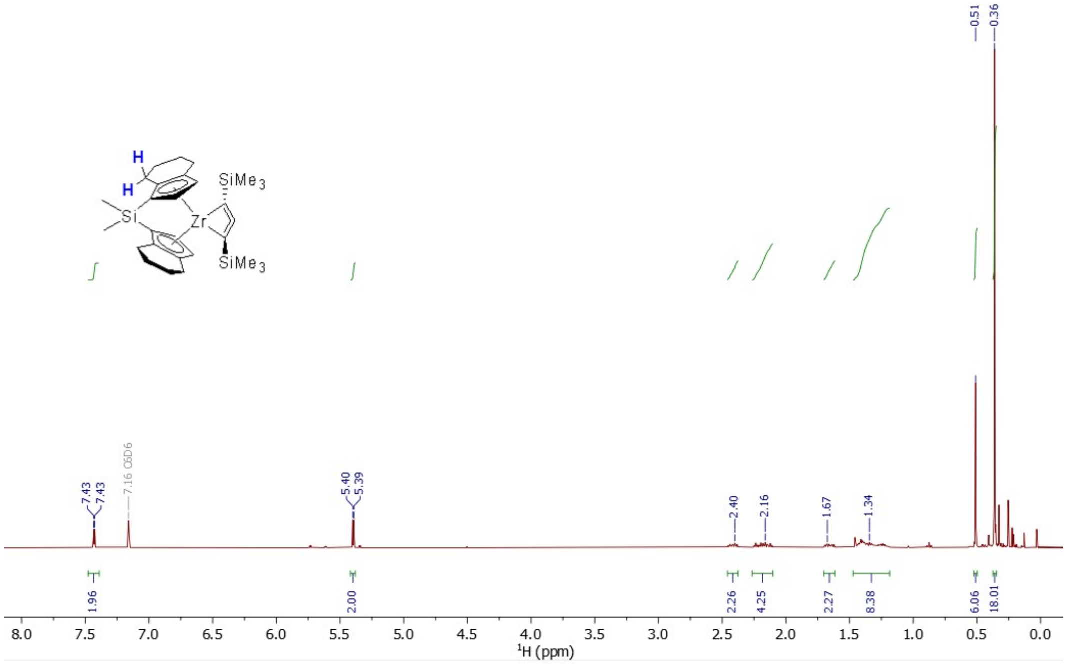

Figure S30. ^{1}H NMR spectrum of **2b** (25 °C, benzene-d_6, 400.1 MHz).

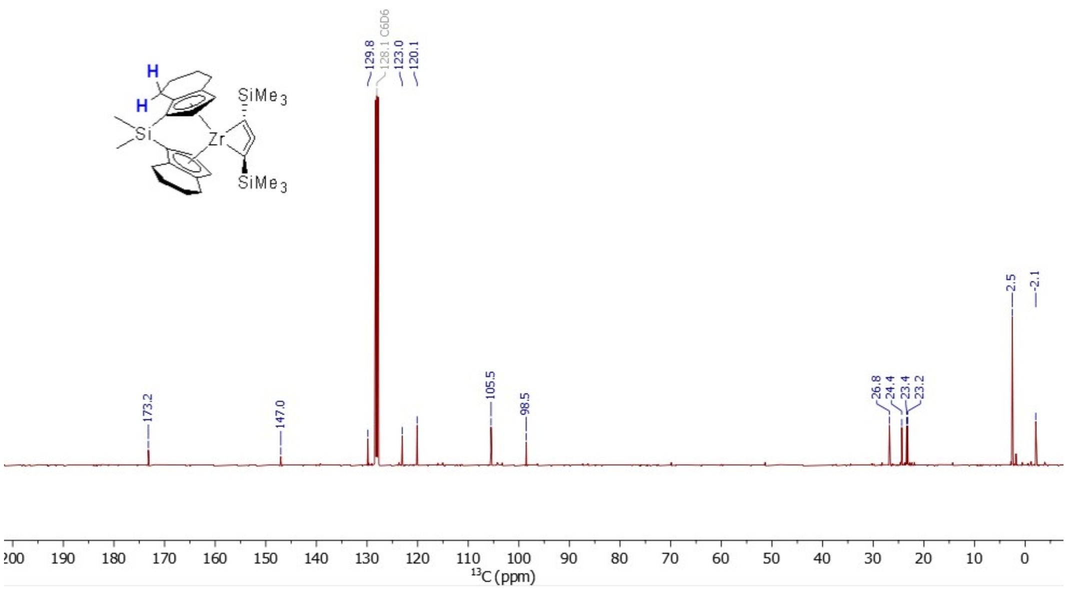

Figure S31. ^{13}C NMR spectrum of **2b** (25 °C, benzene-d_6, 100.6 MHz).

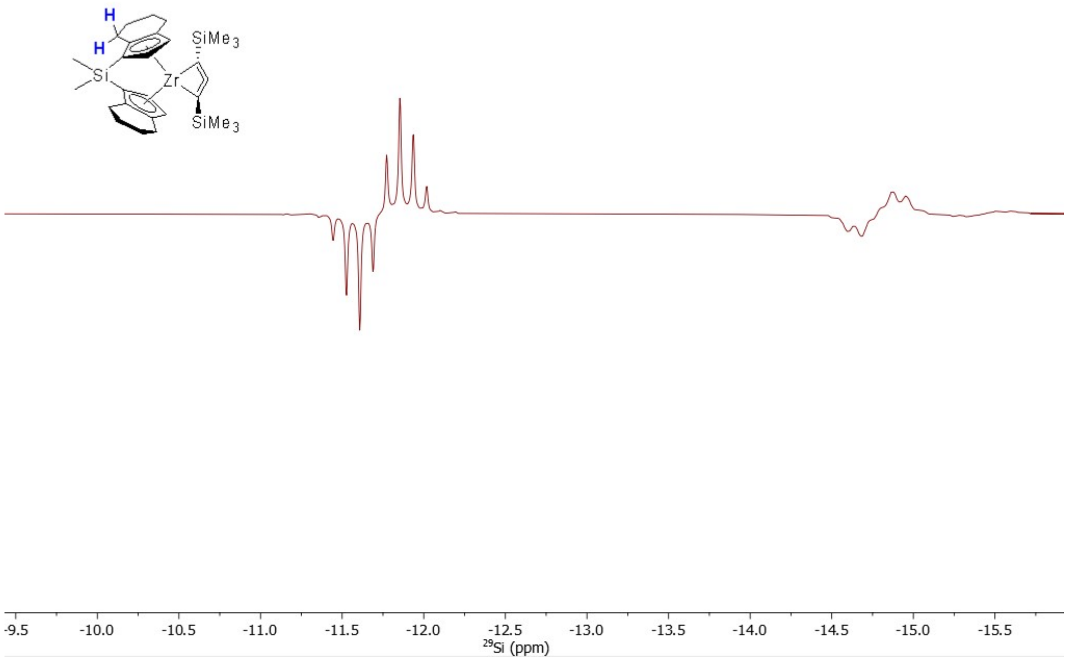

Figure S32. ^{29}Si INEPT NMR spectrum of **2b** (25 °C, benzene-d_6, 79.5 MHz).

6.3 NMR spectra of 3a

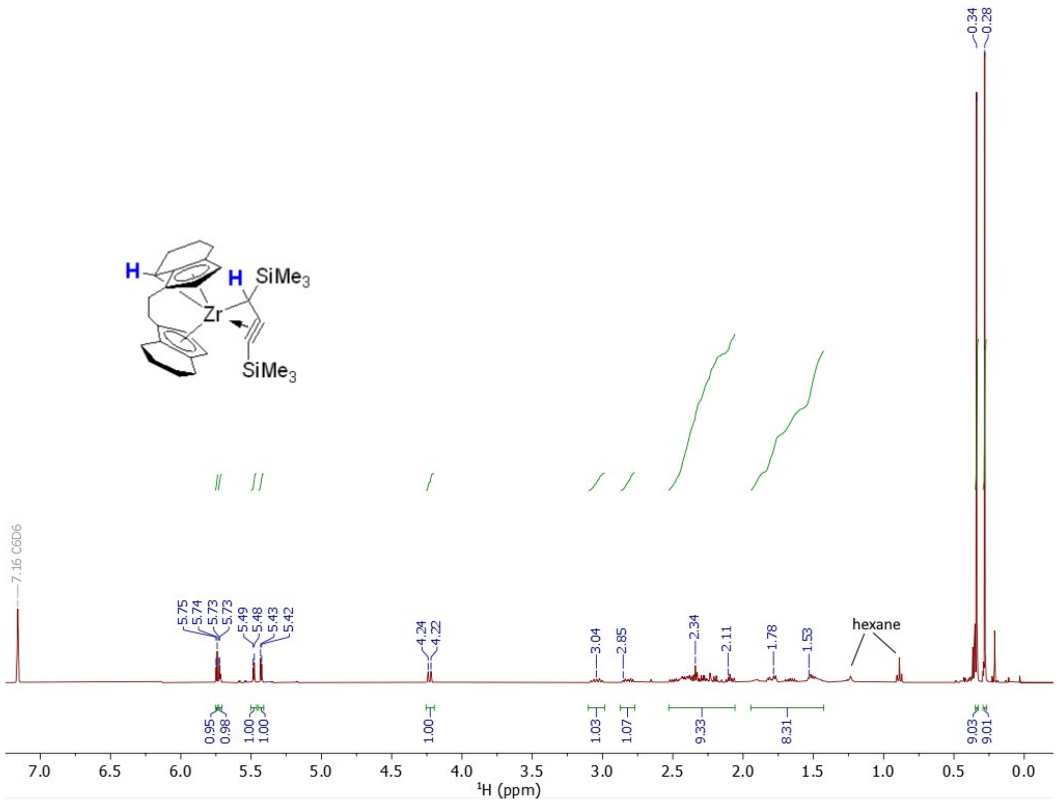

Figure S33. ^{1}H NMR spectrum of **3a** (25 °C, benzene-d_6, 400.1 MHz).

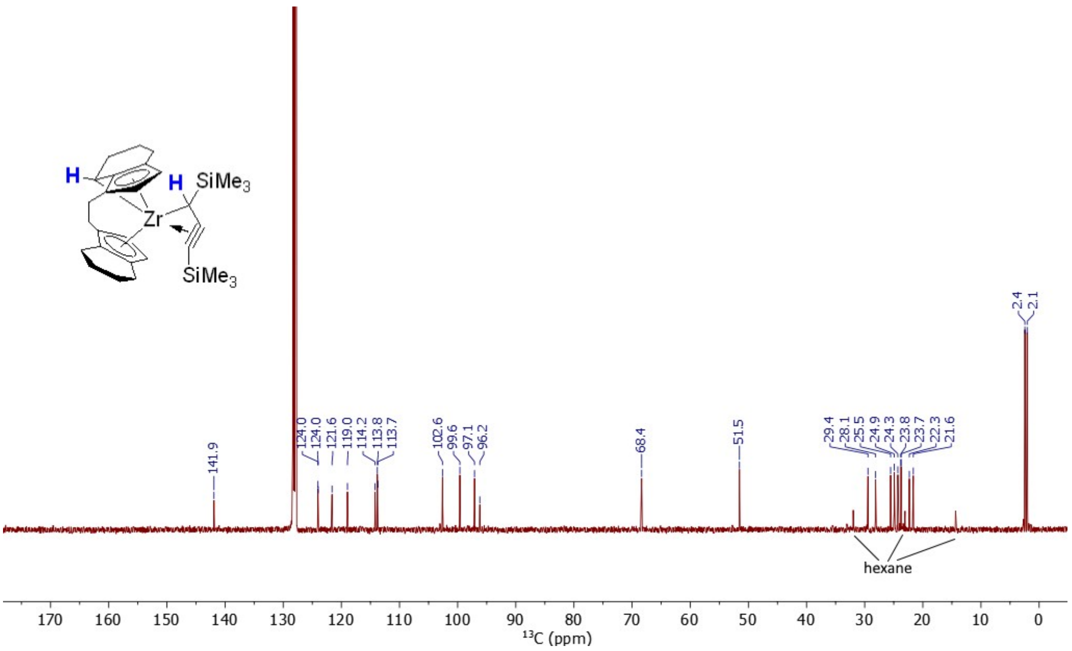

Figure S34. ^{13}C NMR spectrum of **3a** (25 °C, benzene-d_6, 100.6 MHz).

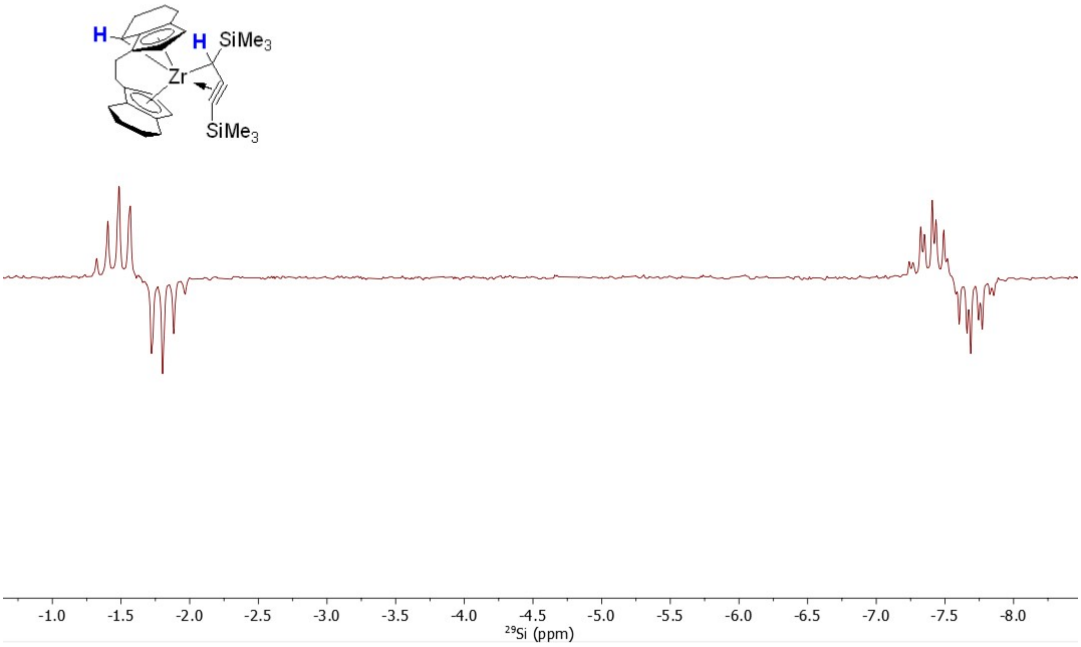

Figure S35. ^{29}Si INEPT NMR spectrum of **3a** (25 °C, benzene-d_6, 79.5 MHz).

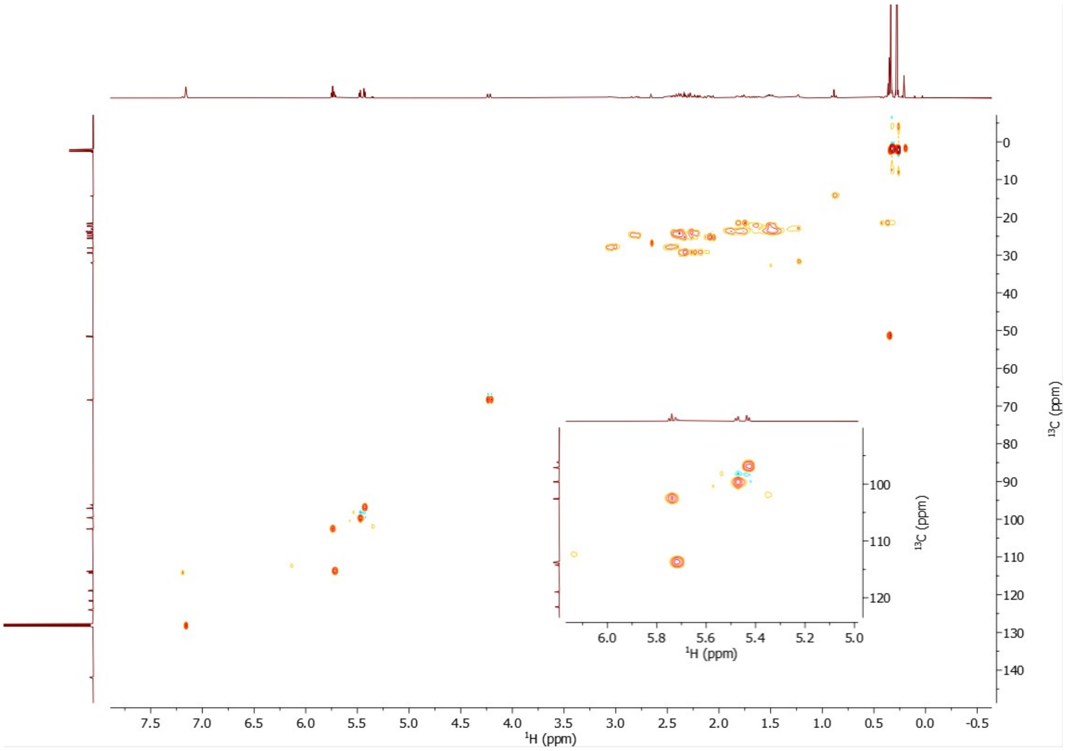

Figure S36. ^{1}H-^{13}C HSQC NMR spectrum of **3a** (25 °C, benzene-d_6, 300.2 MHz).

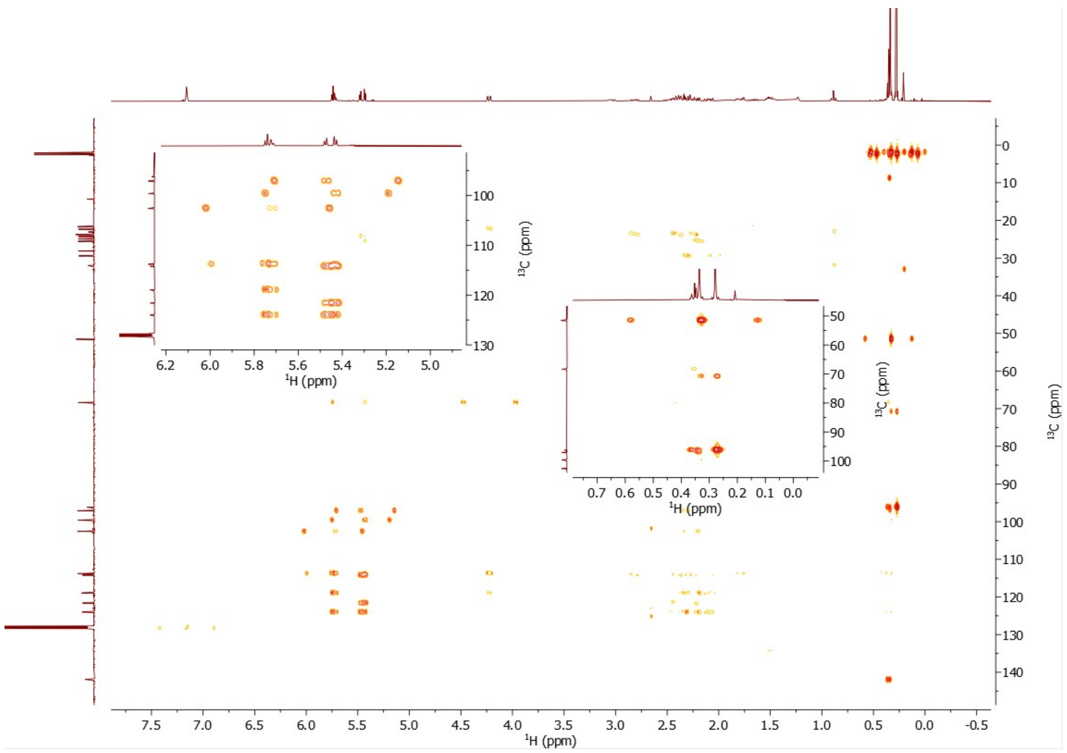

Figure S37. ^{1}H-^{13}C HMBC NMR spectrum of **3a** (25 °C, benzene-d_6, 300.2 MHz).

6.4 NMR spectra of 3b

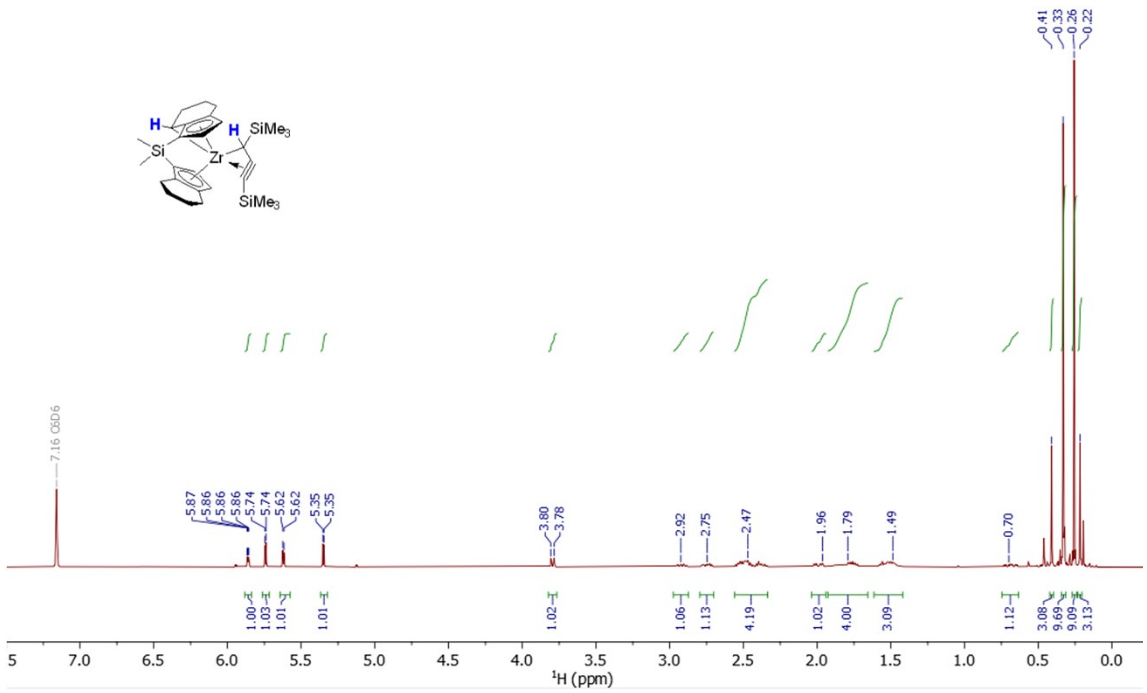

Figure S38. ^{1}H NMR spectrum of **3b** (25 °C, benzene-d_6, 400.1 MHz).

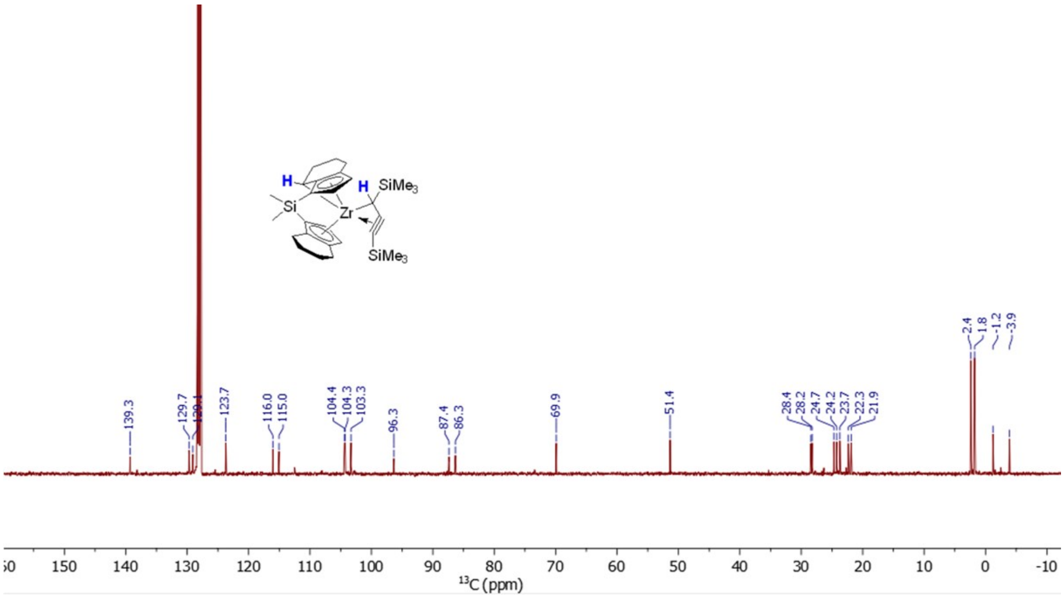

Figure S39. ^{13}C NMR spectrum of **3b** (25 °C, benzene-d_6, 100.6 MHz).

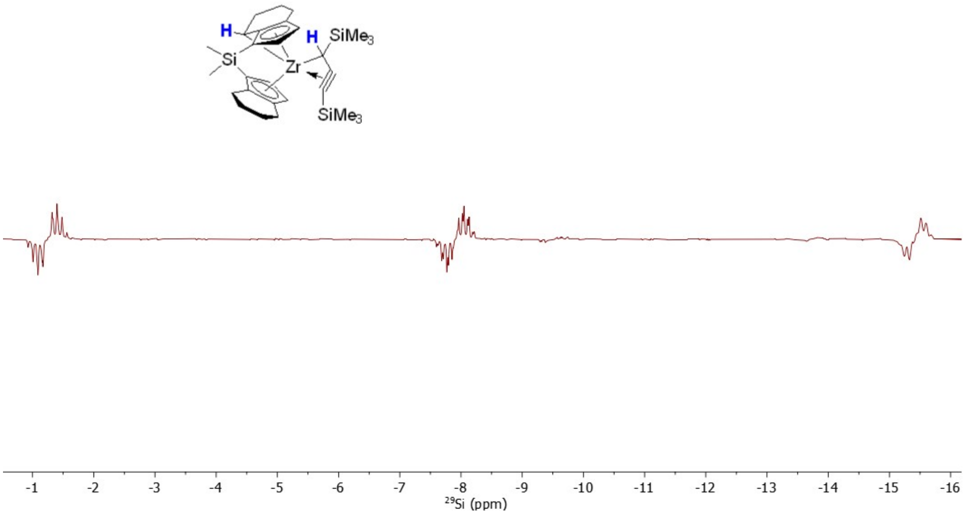

Figure S40. ^{29}Si INEPT NMR spectrum of **3b** (25 °C, benzene-d_6, 79.5 MHz).

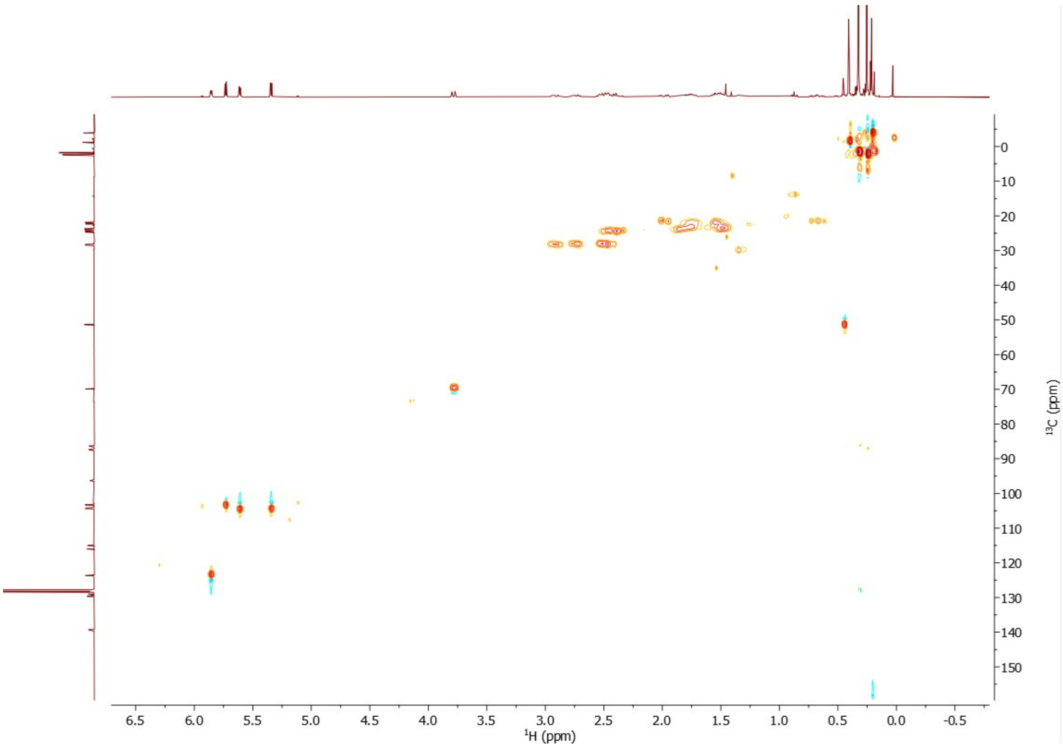

Figure S41. ^{1}H-^{13}C HSQC NMR spectrum of **3b** (25 °C, benzene-d_6, 300.2 MHz).

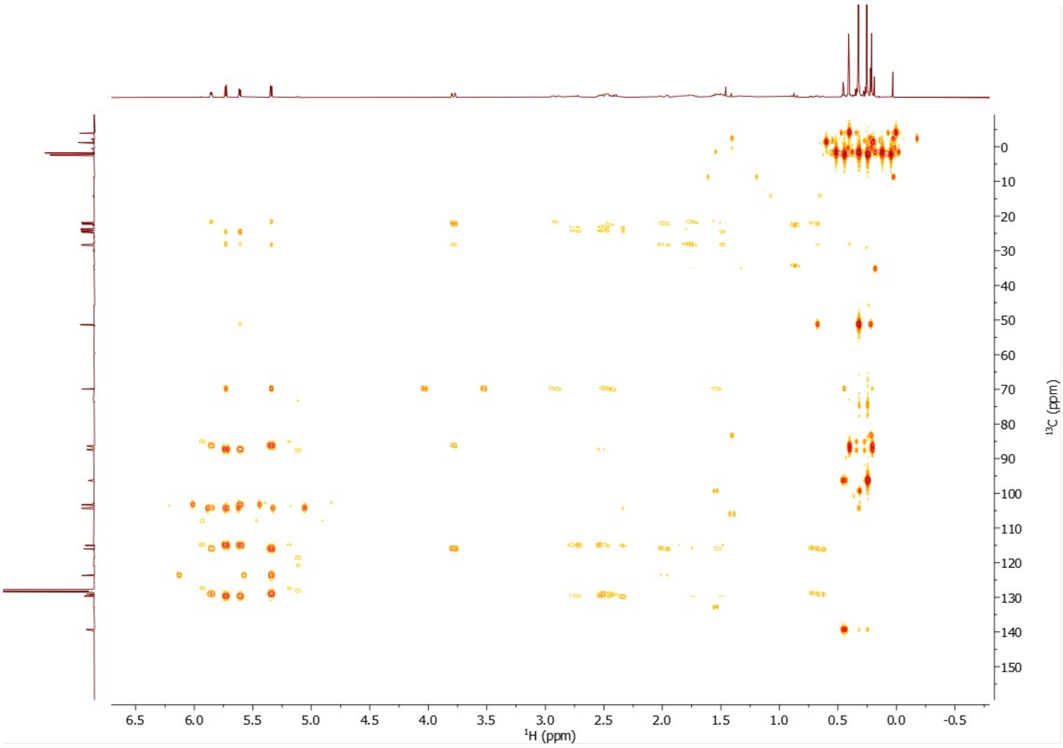

Figure S42. ^{1}H-^{13}C HMBC NMR spectrum of **3b** (25 °C, benzene-d_6, 300.2 MHz).

6.5 NMR spectra of 4

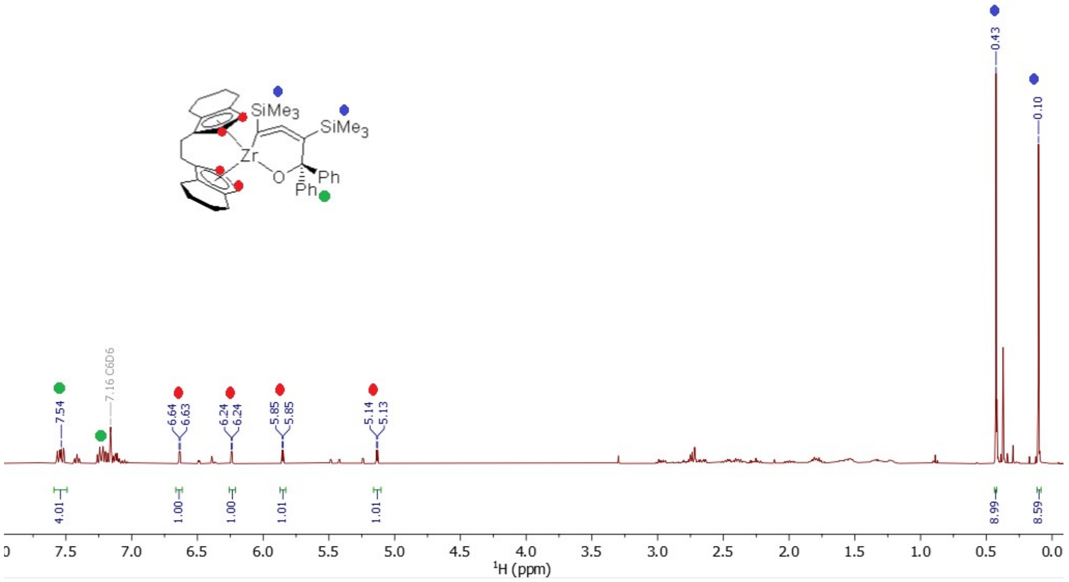

Figure S43. ^{1}H NMR spectrum of **4** (Pure **4** was not obtained after crystallization, compound **13** was obtained as an impurity due to **3a** contained in the starting material **2a**; 25 °C, benzene-d_6, 400.1 MHz).

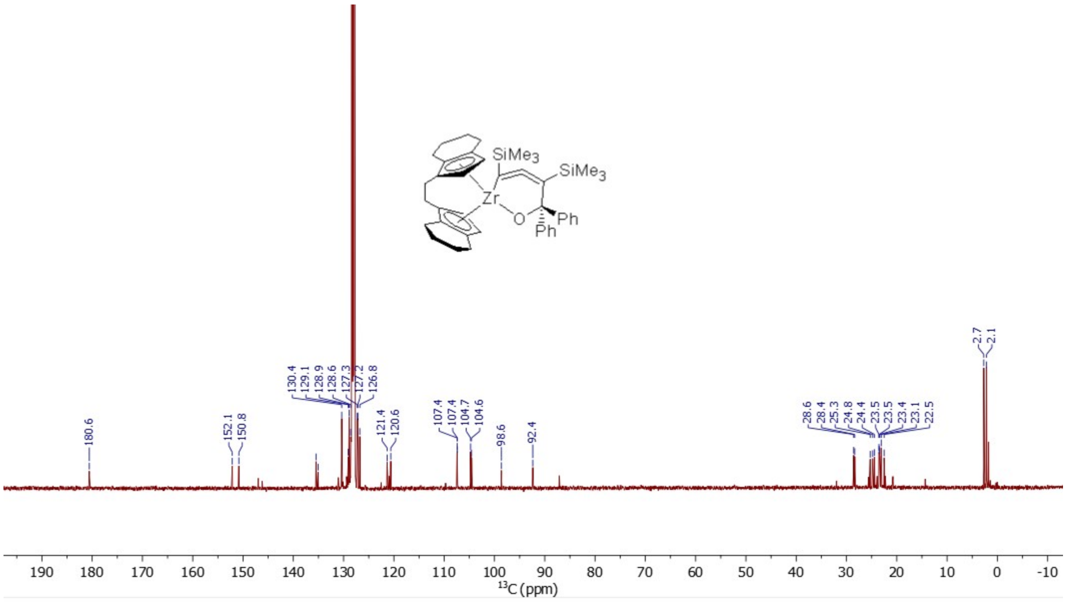

Figure S44. ^{13}C NMR spectrum of **4** (25 °C, benzene-d_6, 100.6 MHz).

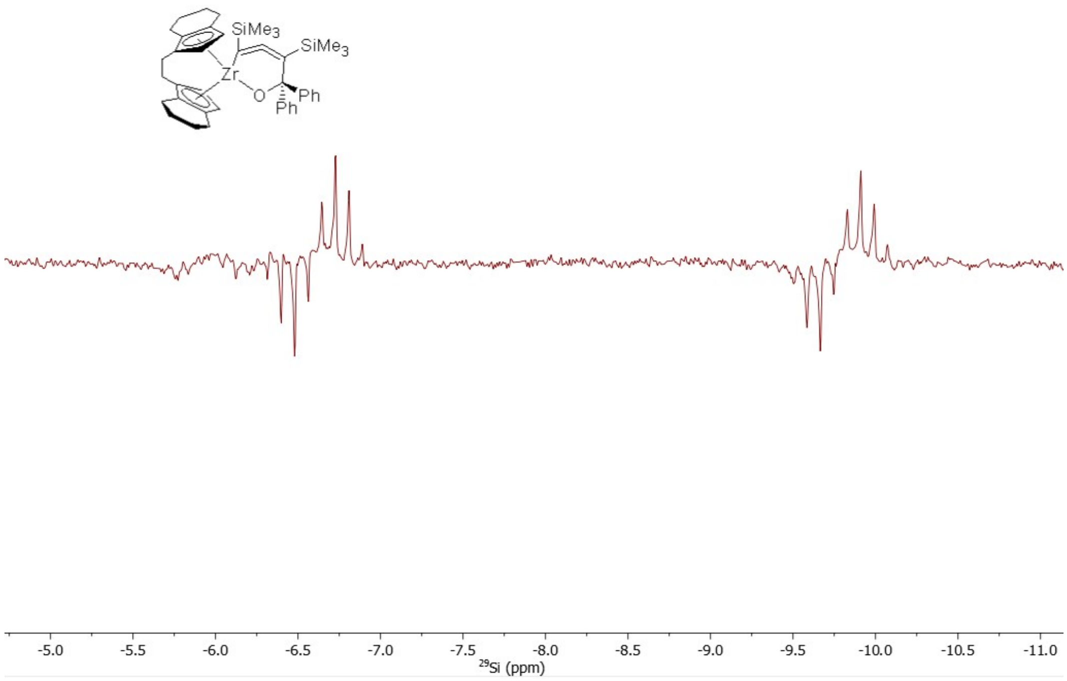

Figure S45. ^{29}Si INEPT NMR spectrum of **4** (25 °C, benzene-d_6, 79.5 MHz).

6.6 NMR spectra of 6 isomer mixture

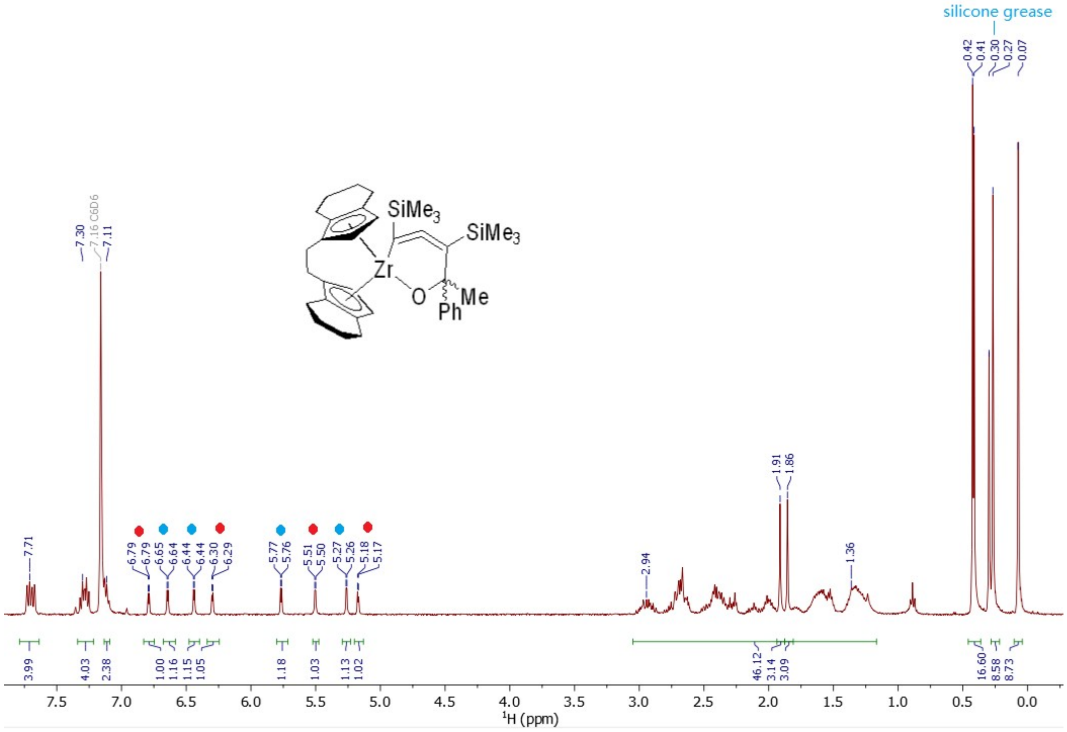

Figure S46. ^{1}H NMR spectrum of **6** (isomer mixture, 25 °C, benzene-d_6, 400.1 MHz).

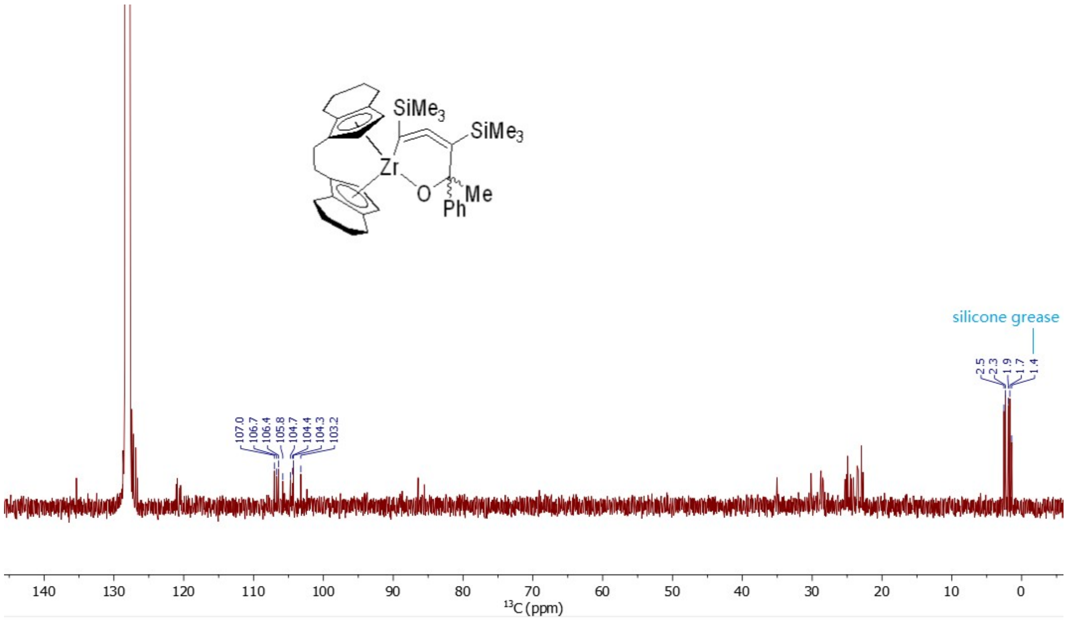

Figure S47. ^{13}C NMR spectrum of **6** (isomer mixture, 25 °C, benzene-d_6, 100.6 MHz).

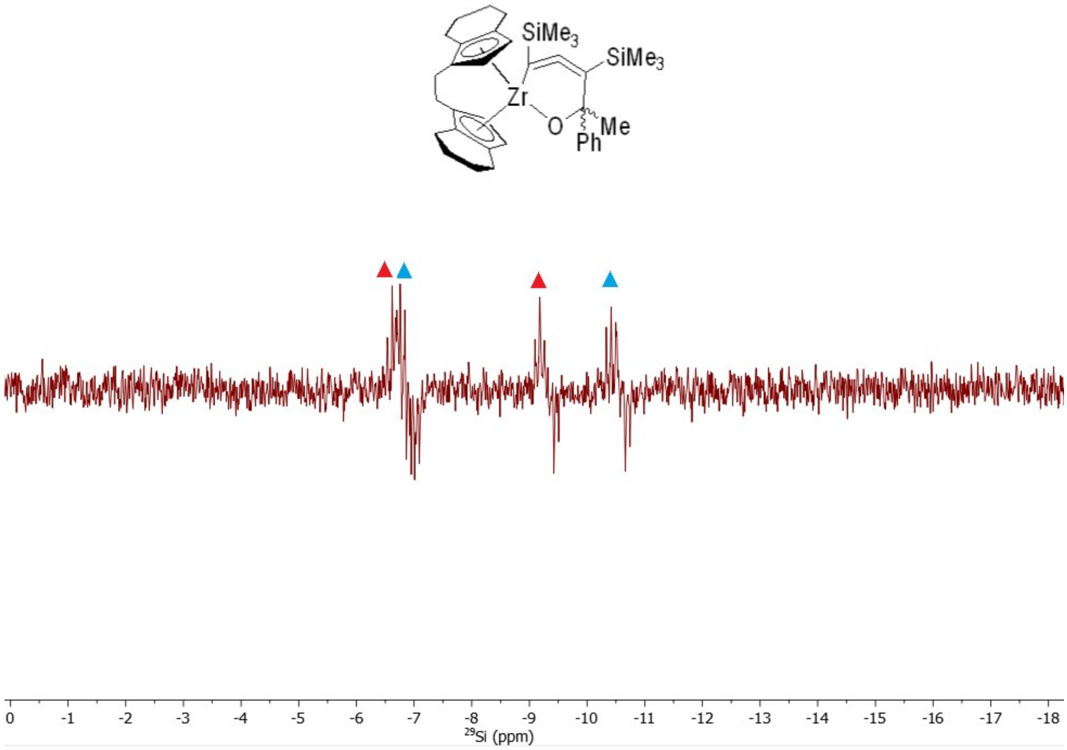

Figure S48. ^{29}Si INEPT NMR spectrum of **6** (isomer mixture, 25 °C, benzene-d_6, 79.5 MHz).

6.7 NMR spectra of 8

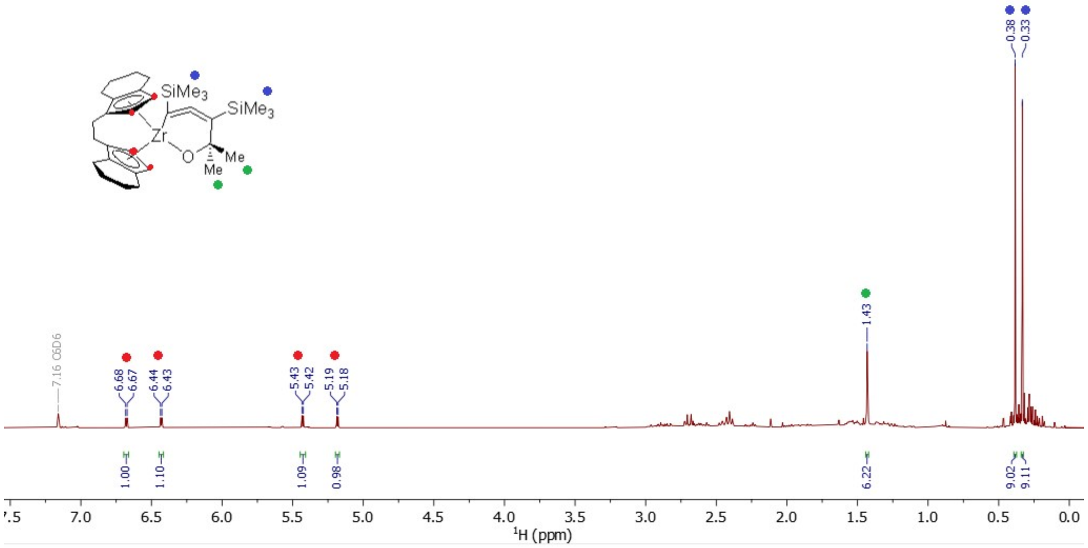

Figure S49. ^{1}H NMR spectrum of the reaction mixture for the formation of **8** (25 °C, benzene-d_6, 300.2 MHz).

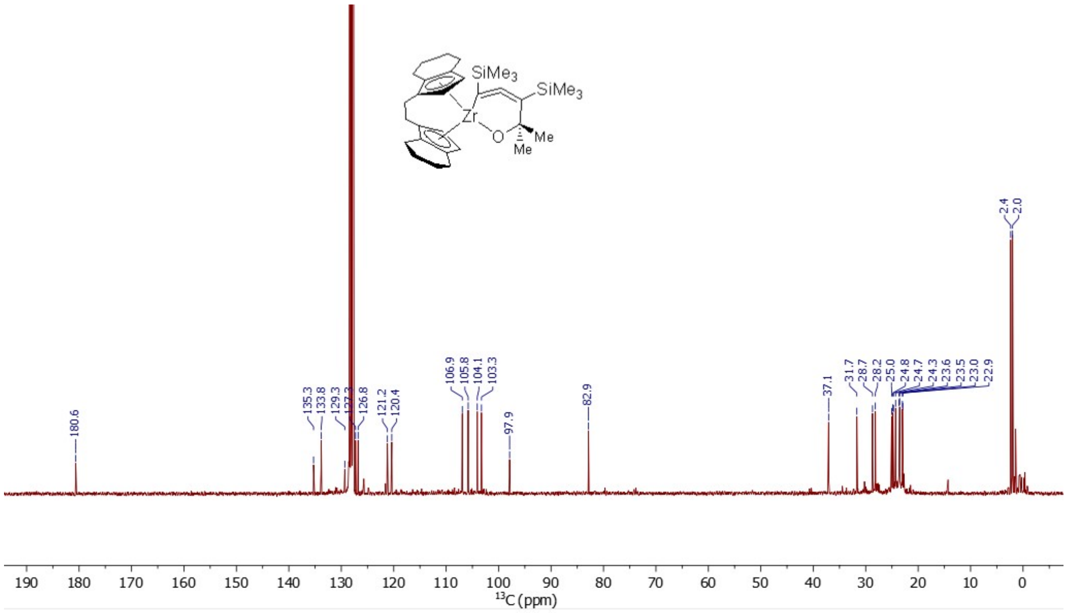

Figure S50. ^{13}C NMR spectrum of **8** (25 °C, benzene-d_6, 75.5 MHz).

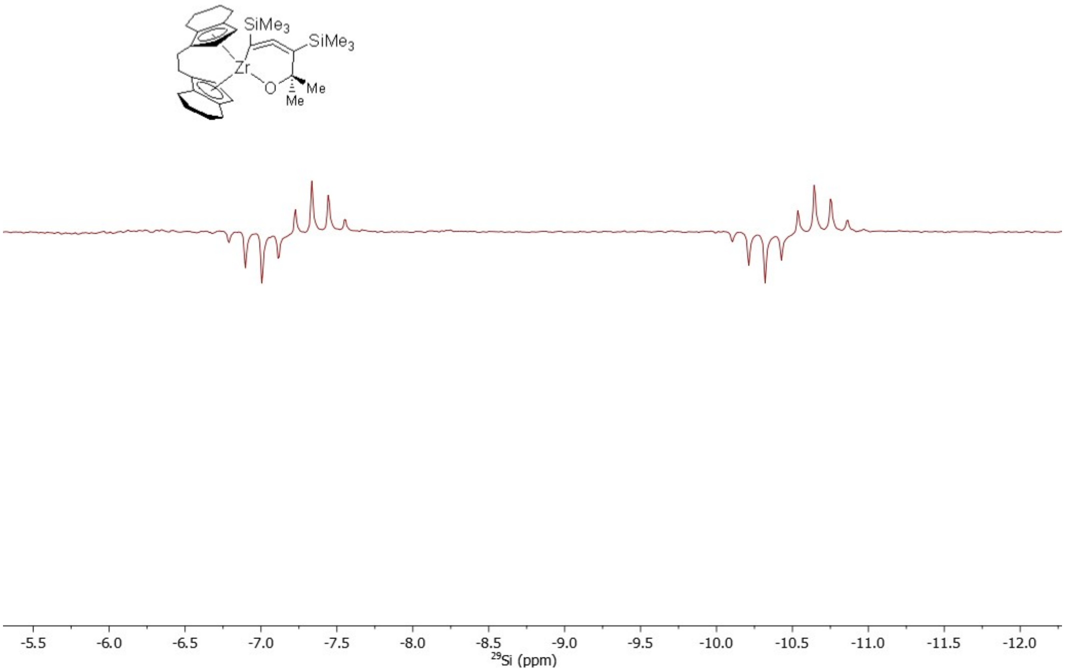

Figure S51. ^{29}Si INEPT NMR spectrum of **8** (25 °C, benzene-d_6, 59.6 MHz).

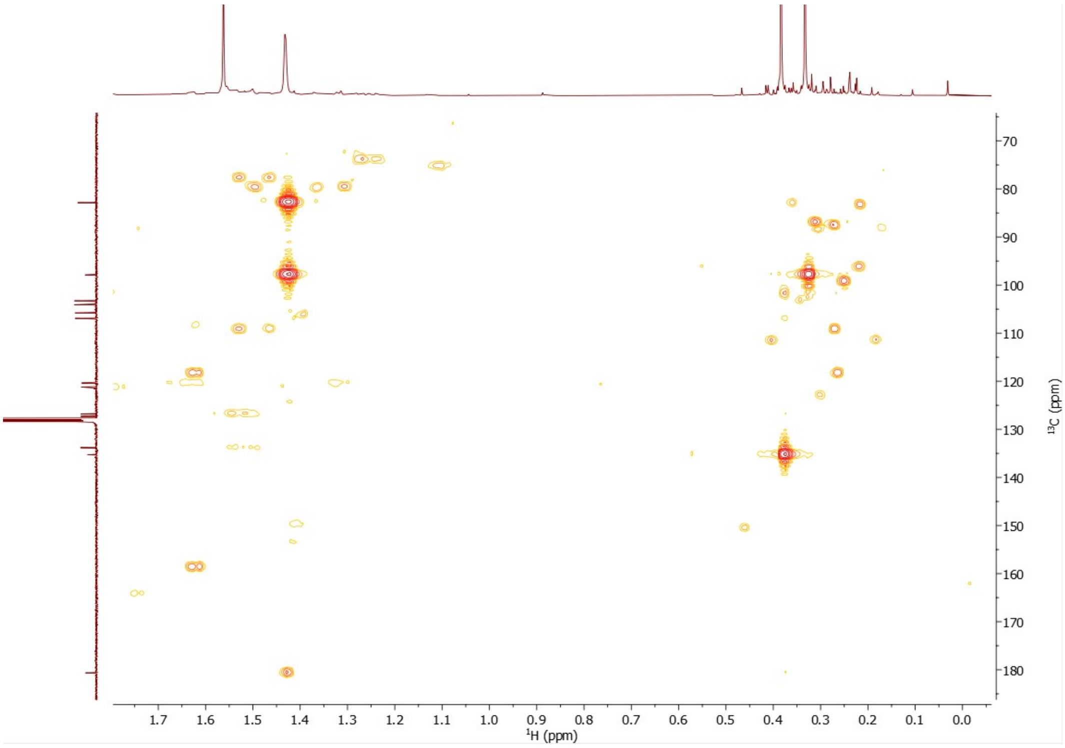

Figure S52. ^{1}H-^{13}C HMBC NMR spectrum of **8** (25 °C, benzene-d_6, 300.2 MHz, high-field region).

6.8 NMR spectra of 13

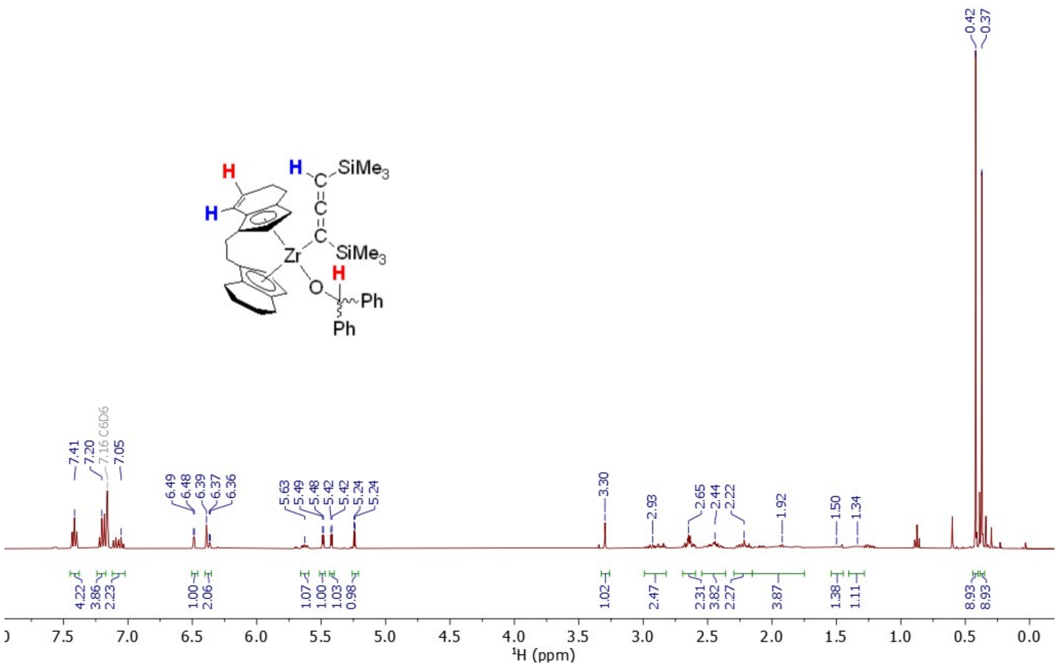

Figure S53. ^{1}H NMR spectrum of **13** (25 °C, benzene-d_6, 400.1 MHz).

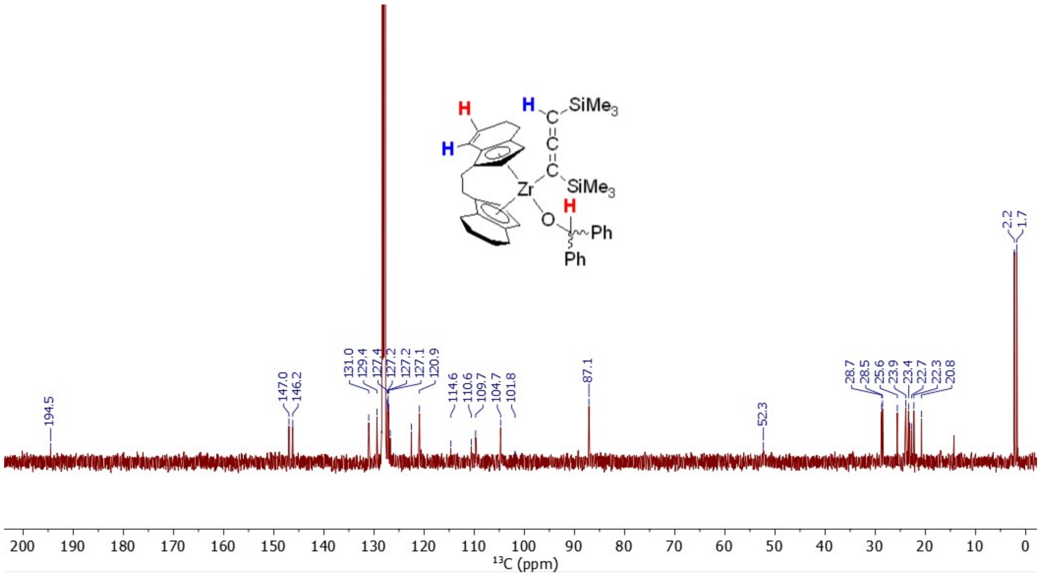

Figure S54. ^{13}C NMR spectrum of **13** (25 °C, benzene-d_6, 100.6 MHz).

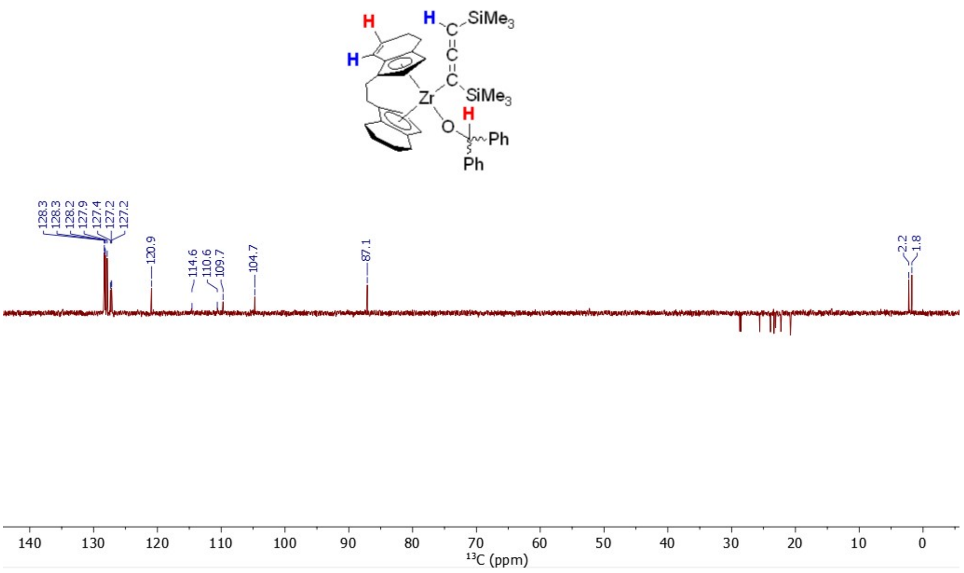

Figure S55. ^{13}C DEPT-135 NMR spectrum of **13** (25 °C, benzene-d_6, 100.6 MHz).

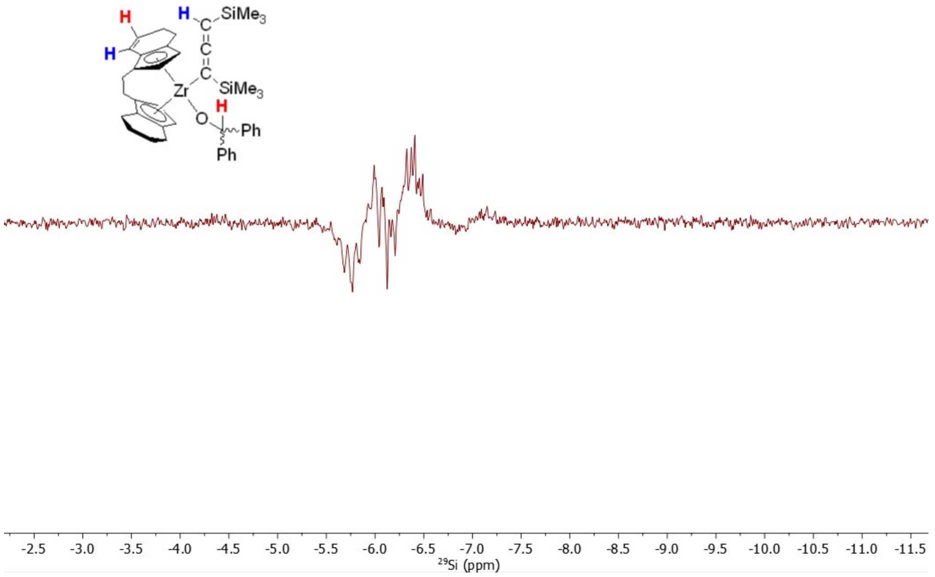

Figure S56. ^{29}Si INEPT NMR spectrum of **13** (25 °C, benzene-d_6, 79.5 MHz).

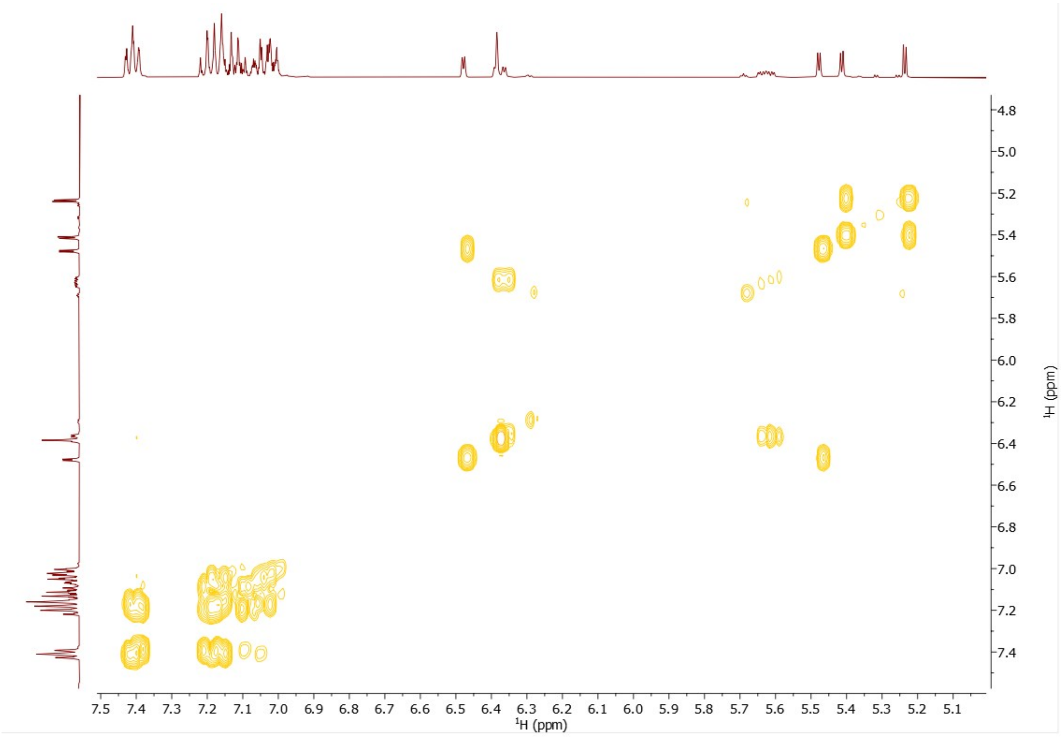

Figure S57. ^{1}H-^{1}H COSY NMR spectrum of **13** (25 °C, benzene-d_6, 400.1 MHz, low-field region).

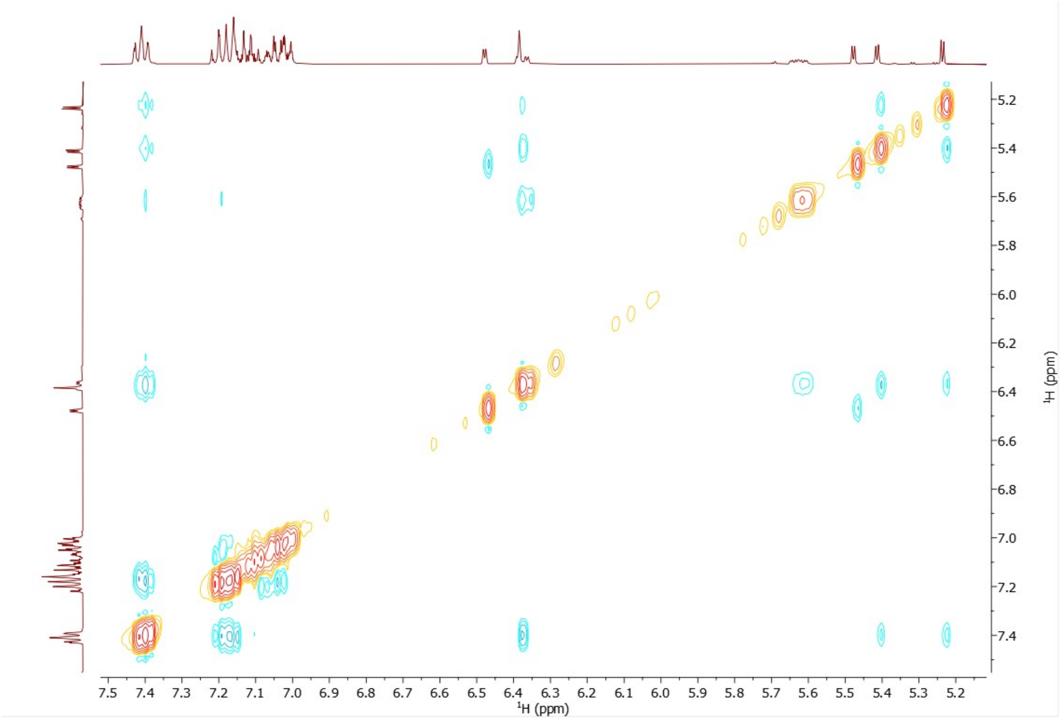

Figure S58. ^{1}H-^{1}H NOESY NMR spectrum of **13** (25 °C, benzene-d_6, 400.1 MHz, low-field region).

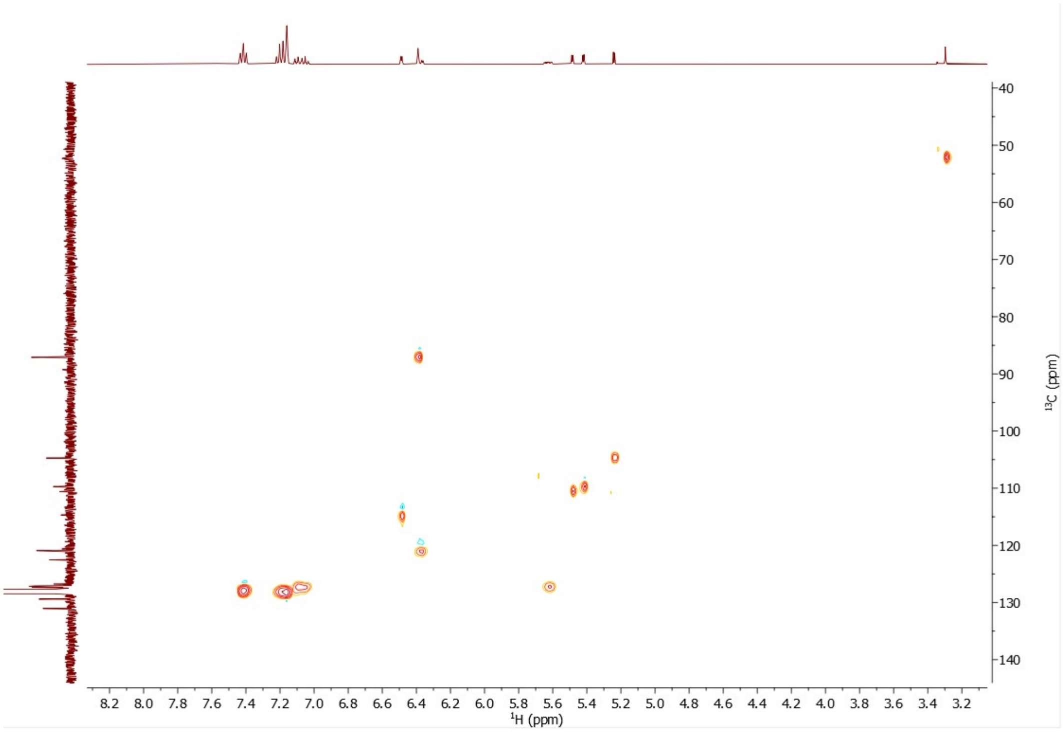

Figure S59. ${}^{1}H$-${}^{13}C$ HSQC NMR spectrum of **13** (25 °C, benzene-d_6, 400.1 MHz, low-field region).

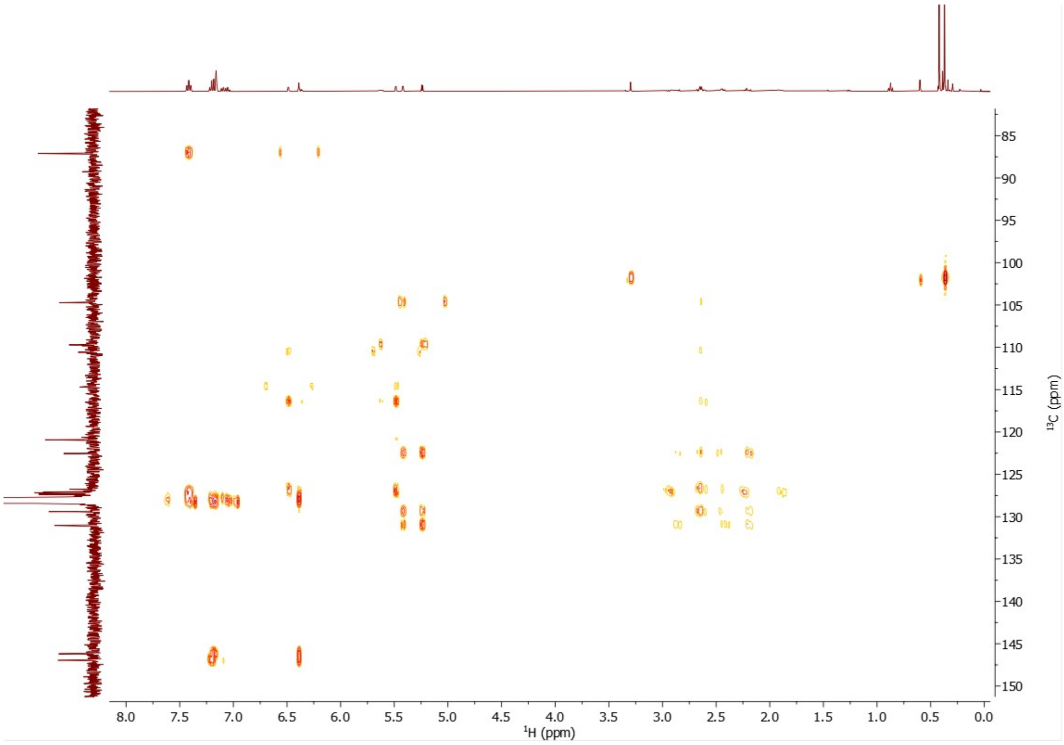

Figure S60. ${}^{1}H$-${}^{13}C$ HMBC NMR spectrum of **13** (25 °C, benzene-d_6, 400.1 MHz, low-field region).

6.9 NMR spectra of **14**

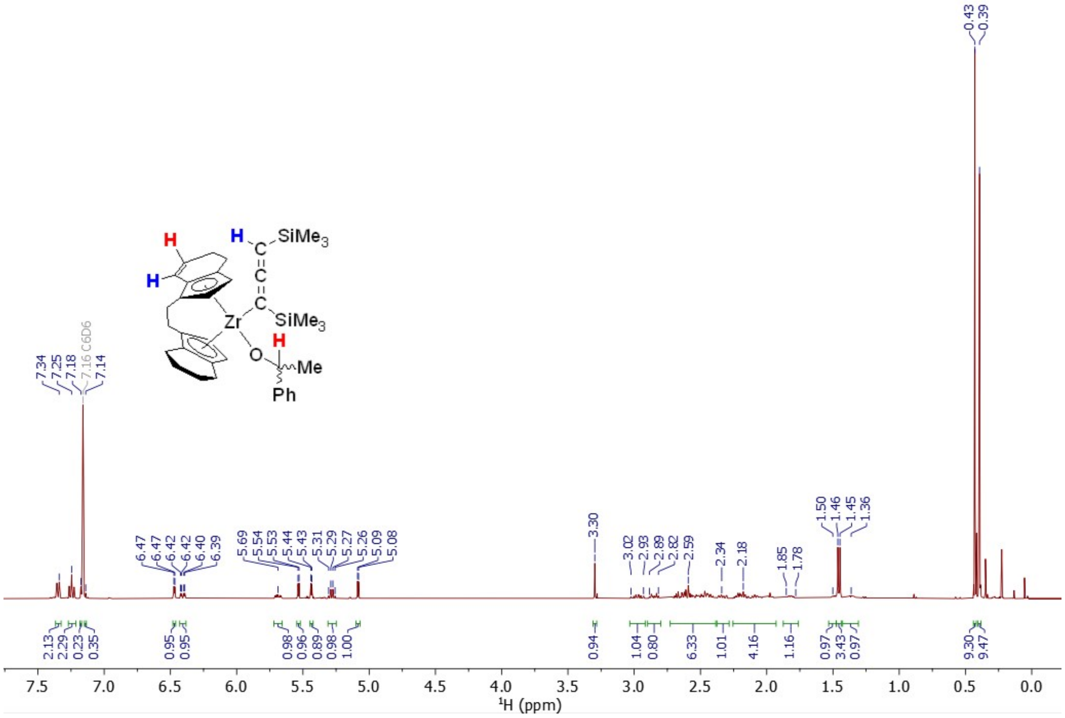

Figure S61. ^{1}H NMR spectrum of **14** (25 °C, benzene-d_6, 400.1 MHz).

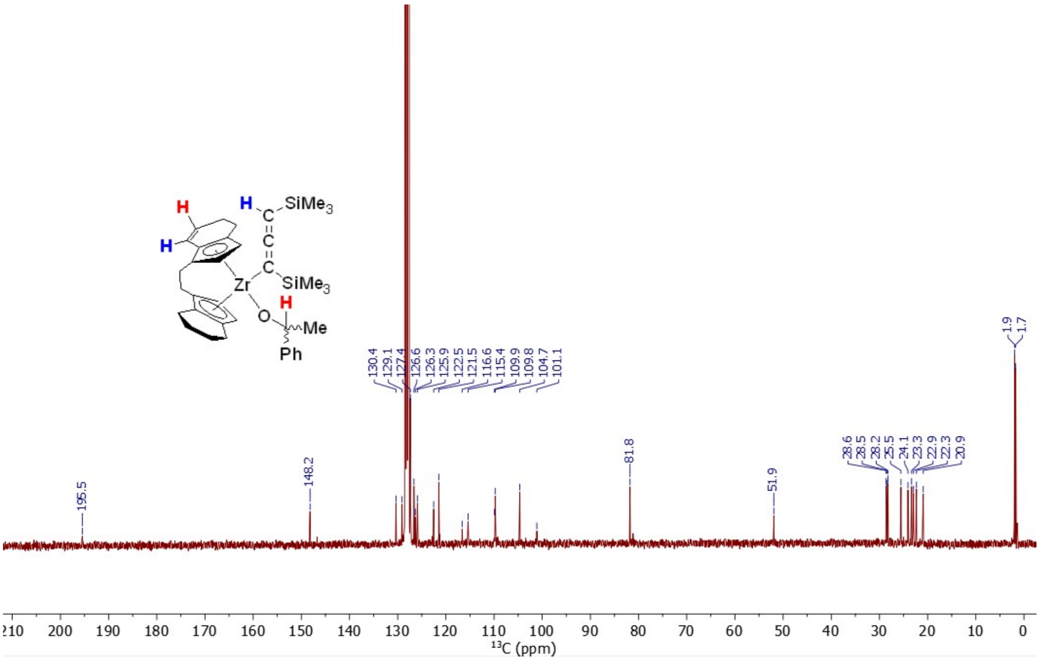

Figure S62. ^{13}C NMR spectrum of **14** (25 °C, benzene-d_6, 100.6 MHz).

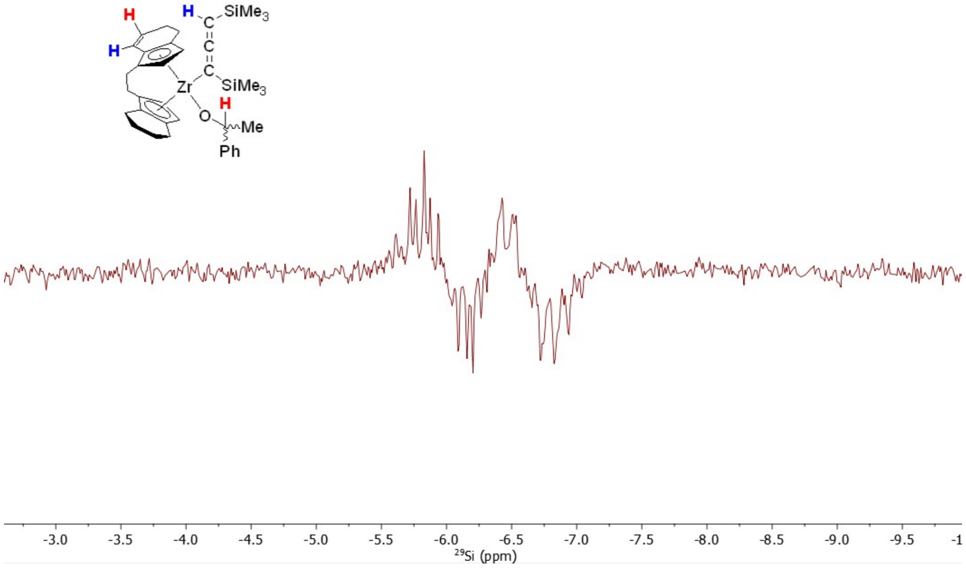

Figure S63. ^{29}Si INEPT NMR spectrum of **14** (25 °C, benzene-d_6, 59.6 MHz).

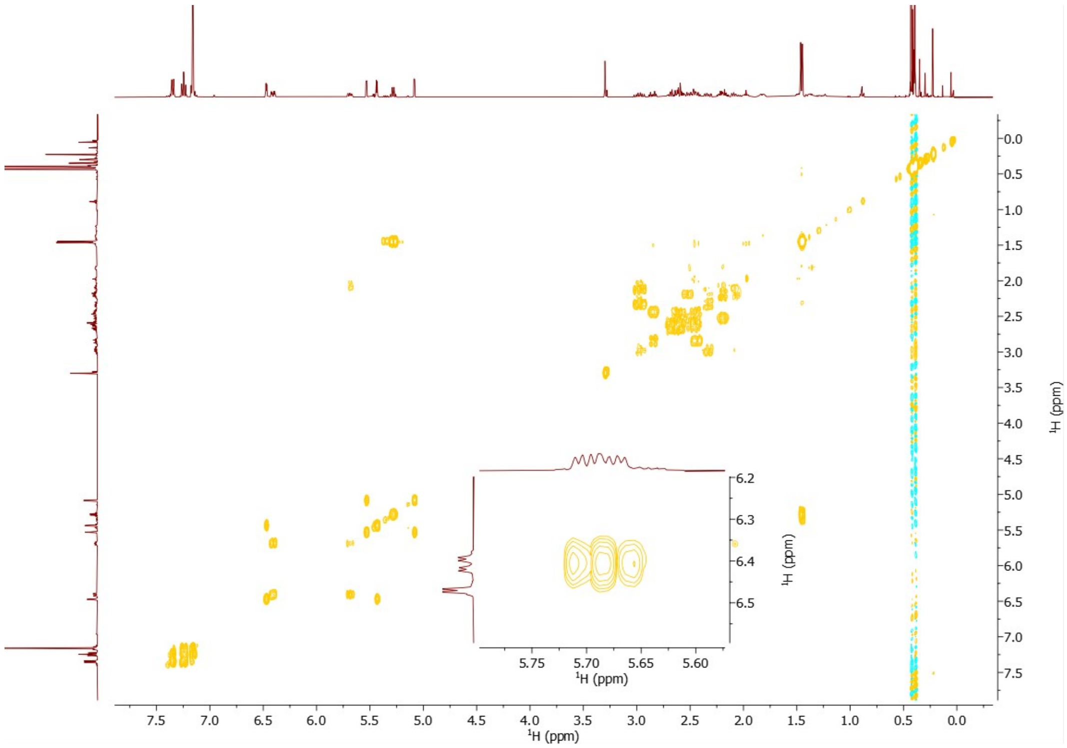

Figure S64. ^{1}H-^{1}H COSY NMR spectrum of **14** (25 °C, benzene-d_6, 400.1 MHz).

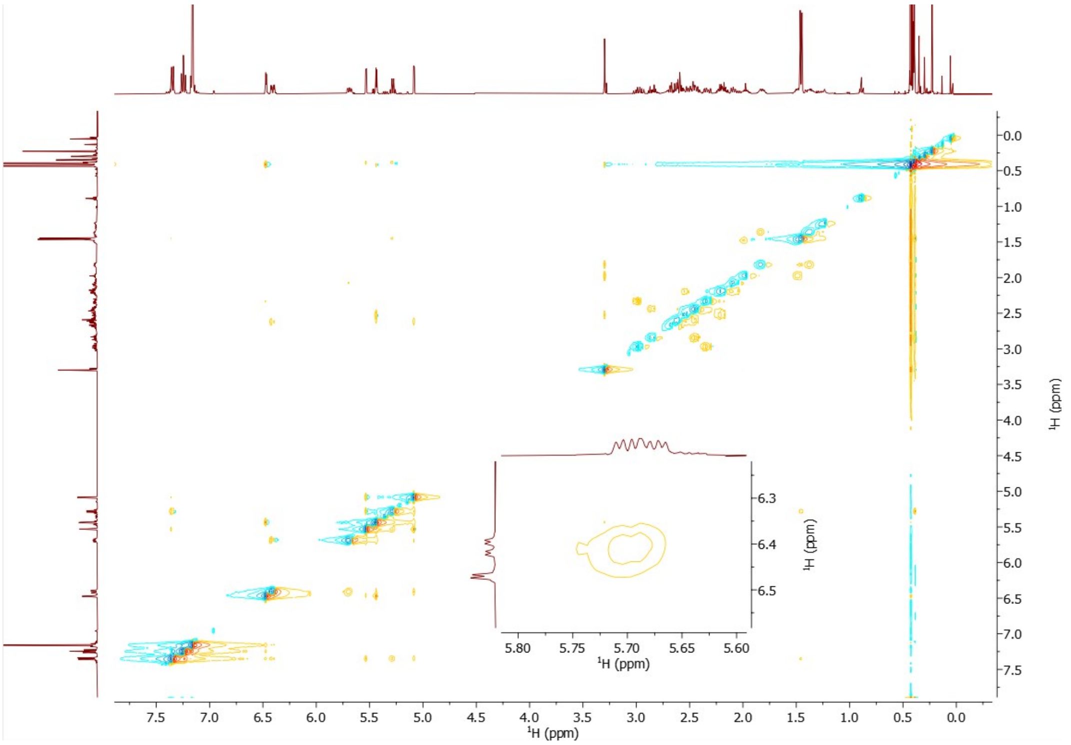

Figure S65. ^{1}H-^{1}H NOESY NMR spectrum of **14** (25 °C, benzene-d_6, 400.1 MHz).

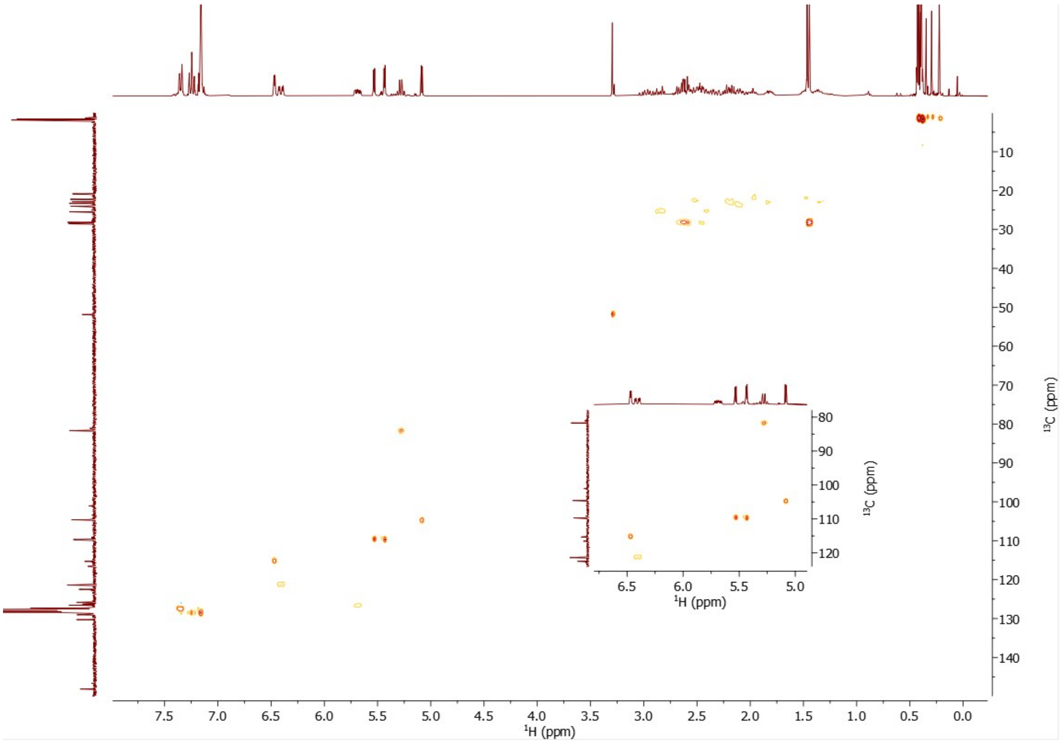

Figure S66. ^{1}H-^{13}C HSQC NMR spectrum of **14** (25 °C, benzene-d_6, 300.2 MHz).

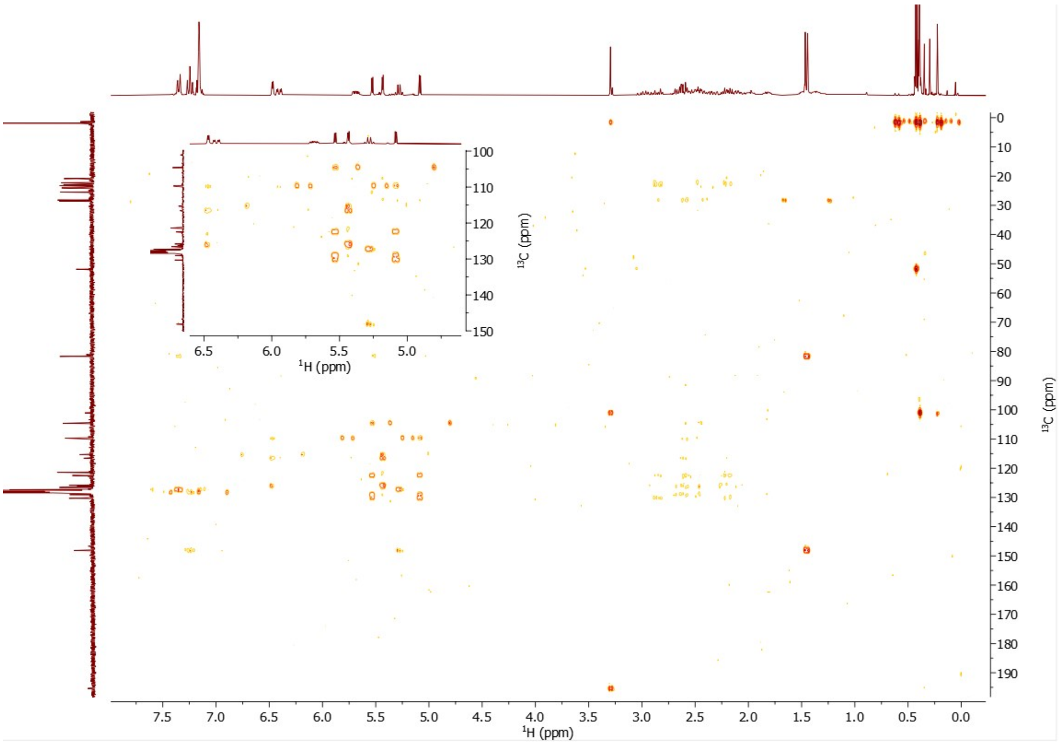

Figure S67. ^{1}H-^{13}C HMBC NMR spectrum of **14** (25 °C, benzene-d_6, 300.2 MHz).

6.10 NMR spectra of 15

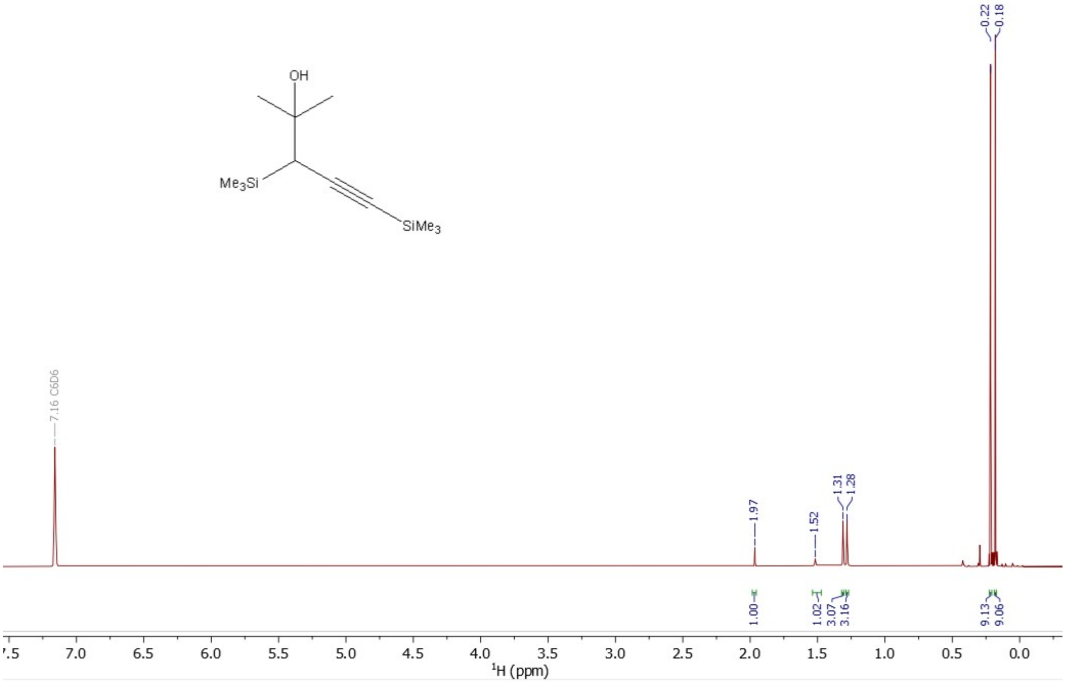

Figure S68. ^{1}H NMR spectrum of **15** (25 °C, benzene-d_6, 300.2 MHz).

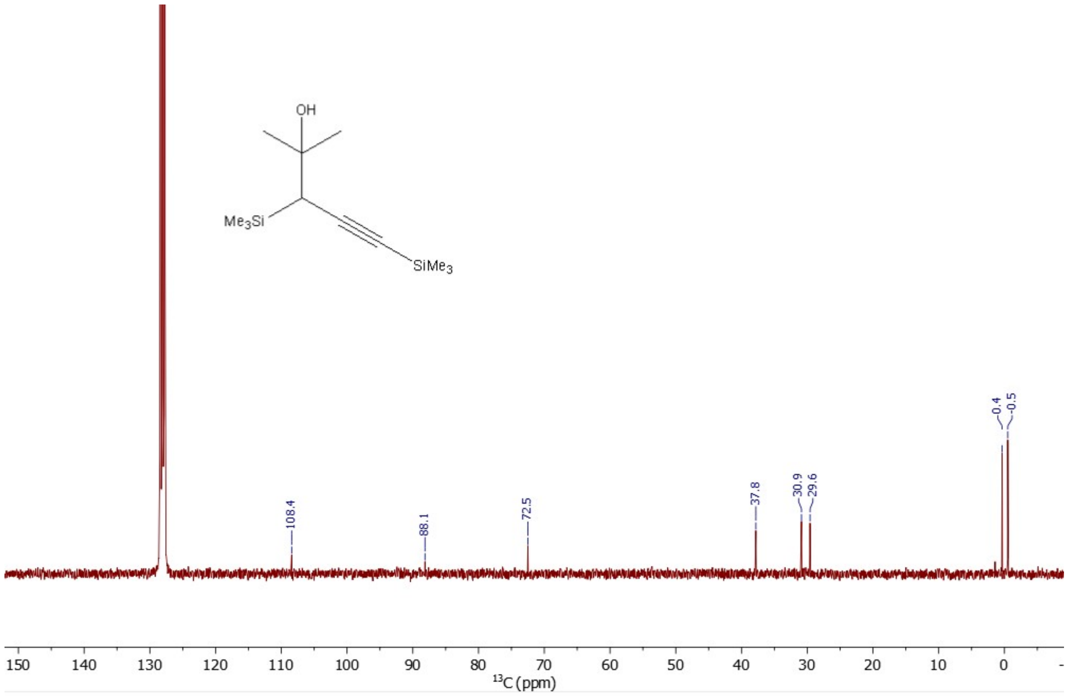

Figure S69. ^{13}C NMR spectrum of **15** (25 °C, benzene-d_6, 75.5 MHz).

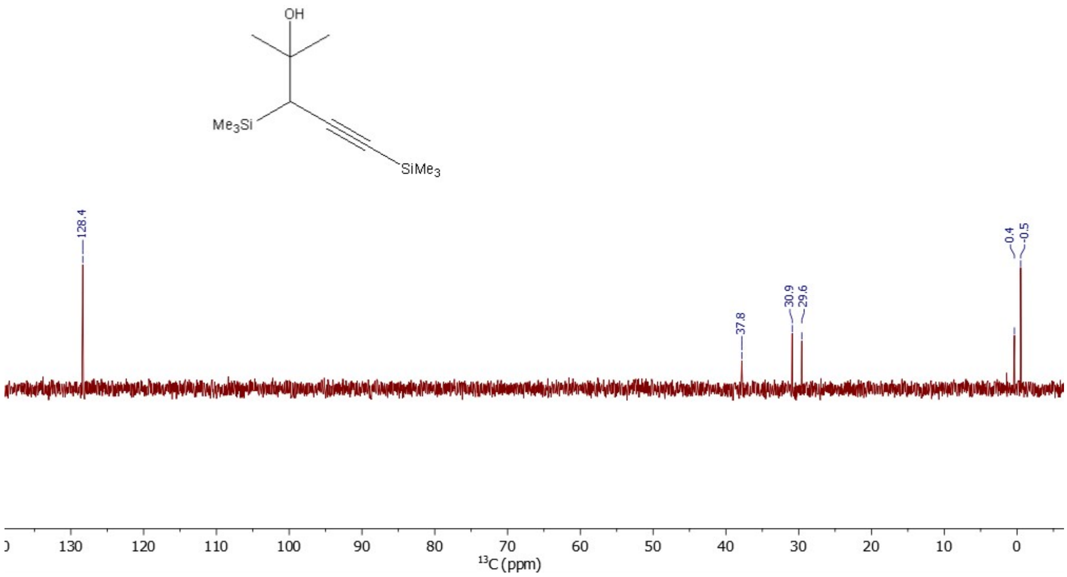

Figure S70. ^{13}C DEPT-135 NMR spectrum of **15** (25 °C, benzene-d_6, 75.5 MHz).

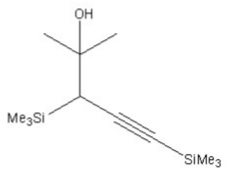

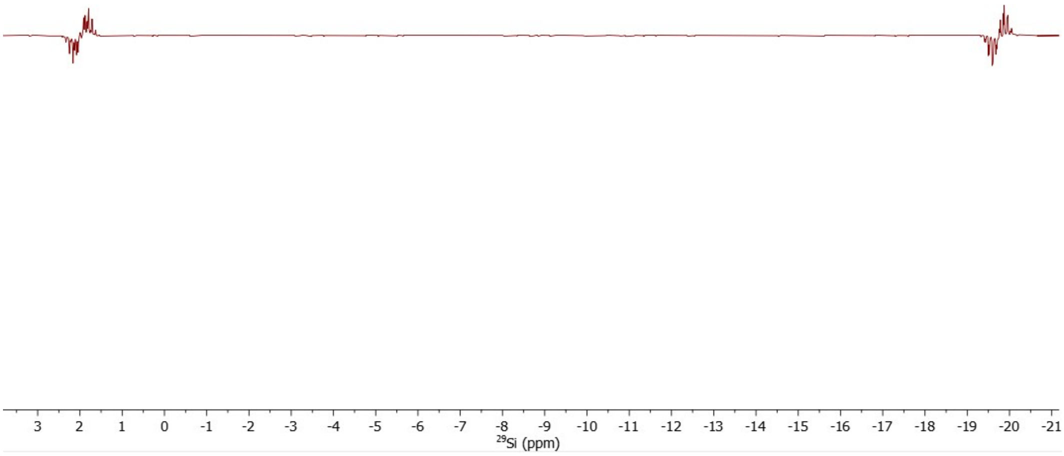

Figure S71. ^{29}Si INEPT NMR spectrum of **15** (25 °C, benzene-d_6, 79.5 MHz).

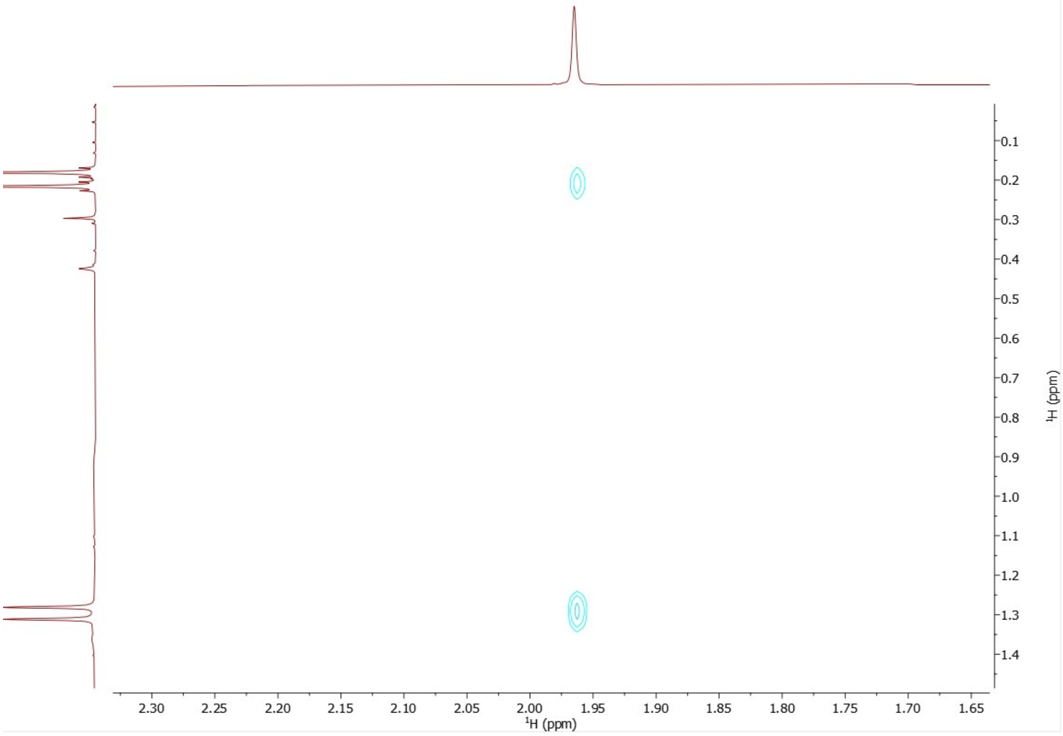

Figure S72. ^{1}H-^{1}H NOESY NMR spectrum of **15** (25 °C, benzene-d_6, 300.1 MHz, high-field region).

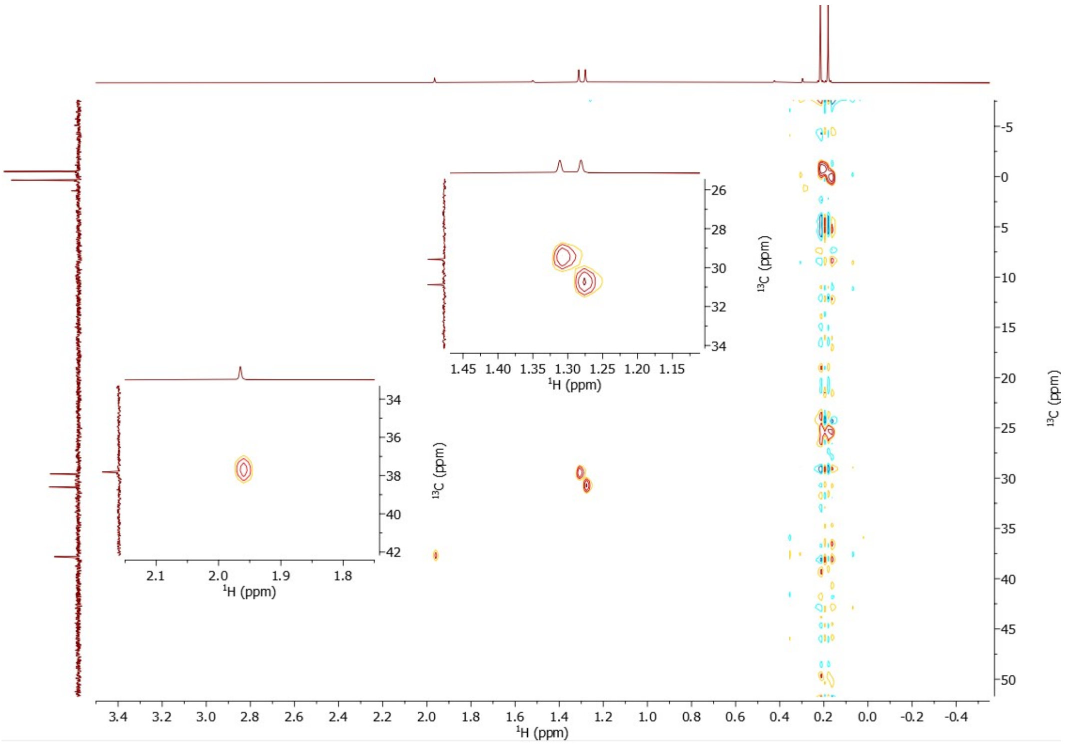

Figure S73. ^{1}H-^{13}C HSQC NMR spectrum of **15** (25 °C, benzene-d_6, 300.1 MHz).

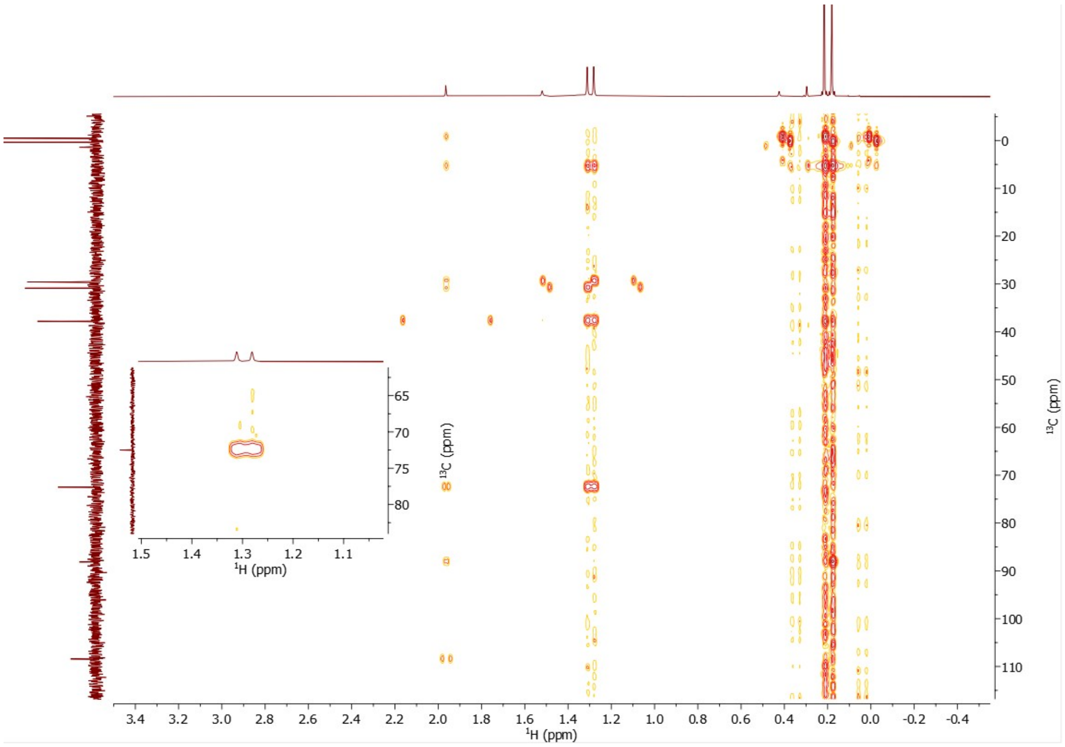

Figure S74. ^{1}H-^{13}C HMBC NMR spectrum of **15** (25 °C, benzene-d_6, 300.1 MHz).

6.11 NMR spectra of 16

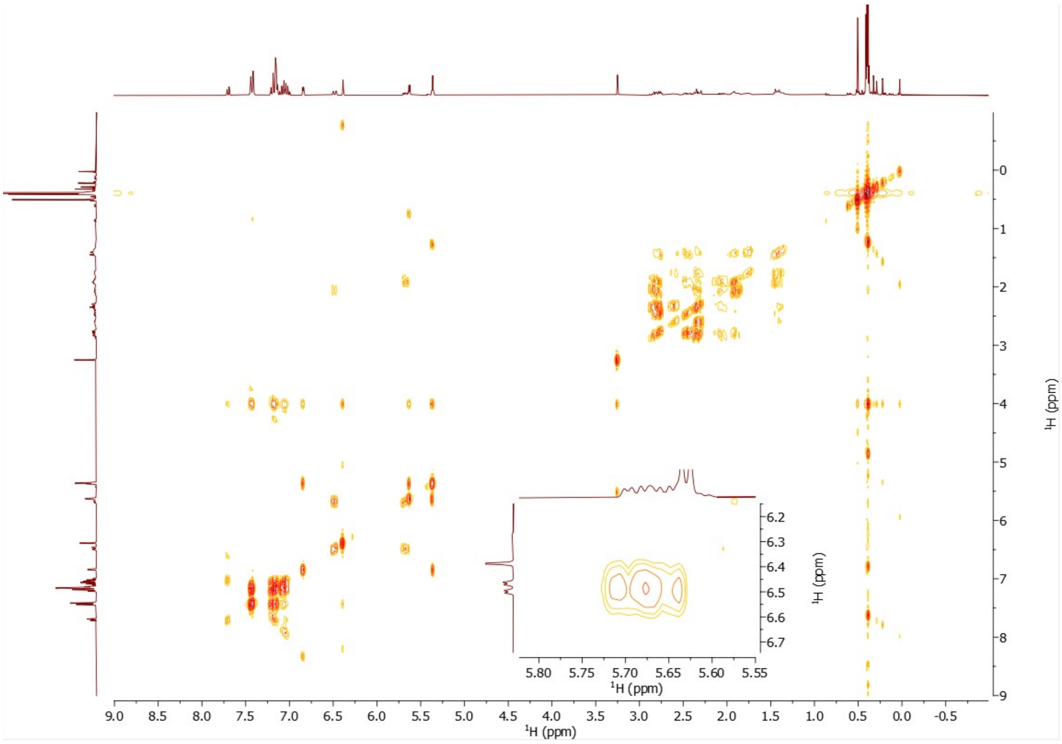

Figure S75. ^{1}H-^{1}H COSY NMR spectrum of **16** (25 °C, benzene-d_6, 300.1 MHz).

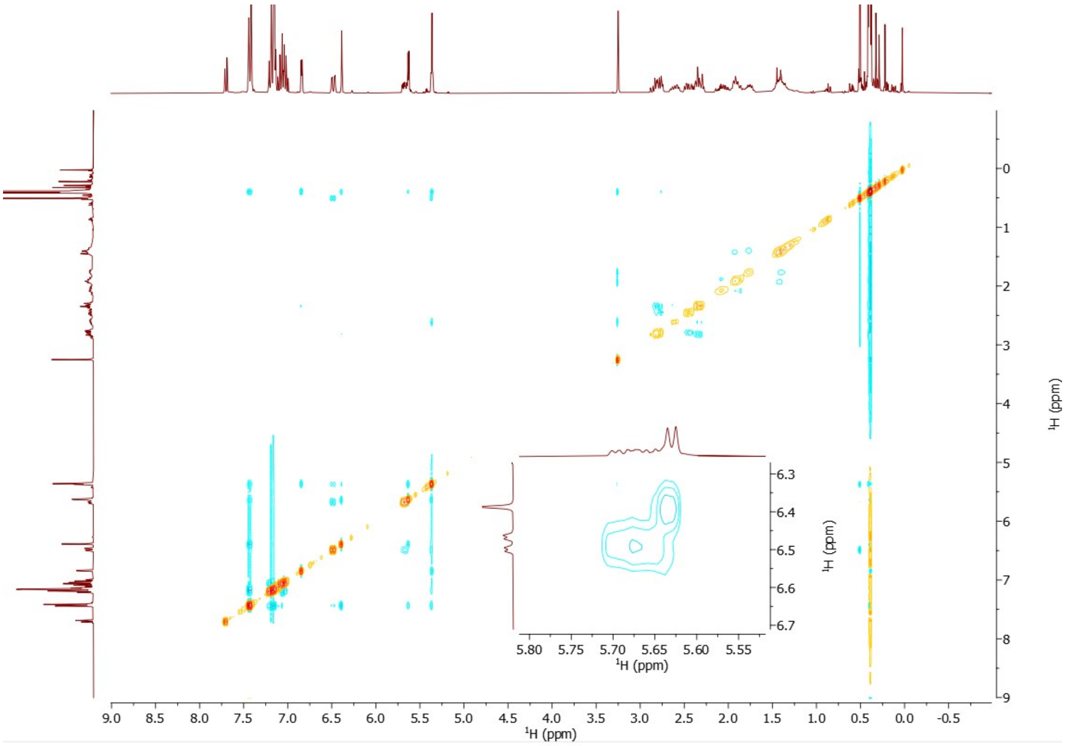

Figure S76. ^{1}H-^{1}H NOESY NMR spectrum of **16** (25 °C, benzene-d_6, 300.1 MHz).

6.12 NMR spectra of 17

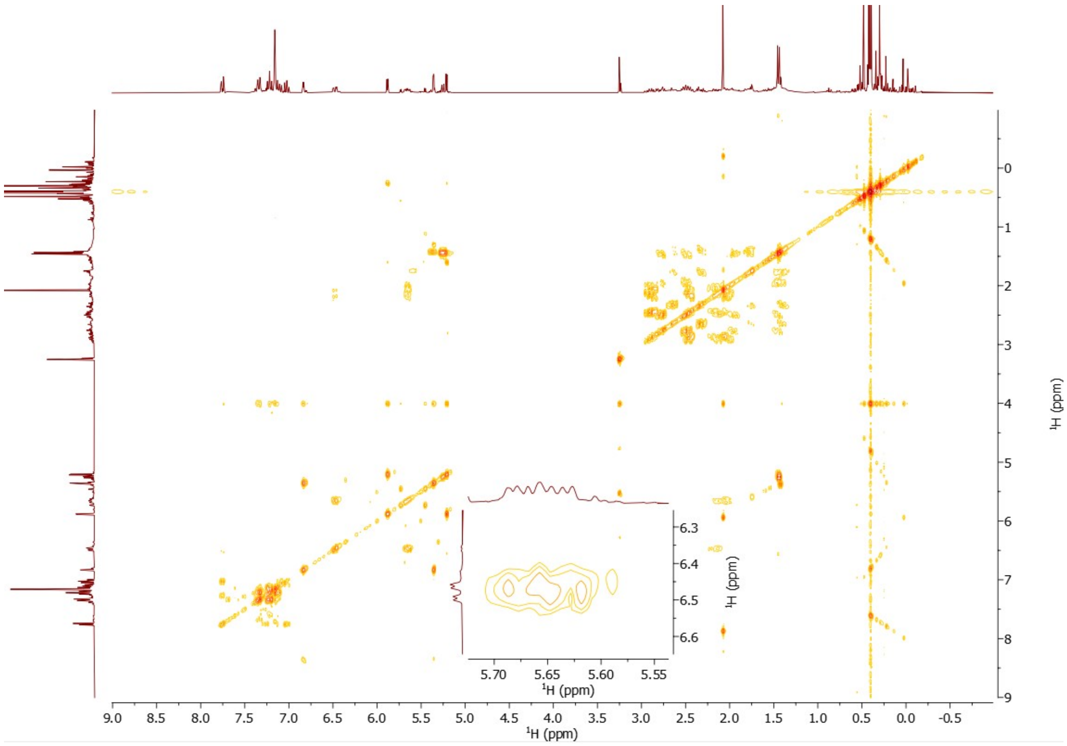

Figure S77. ^{1}H-^{1}H COSY NMR spectrum of **17** (25 °C, benzene-d_6, 300.1 MHz).

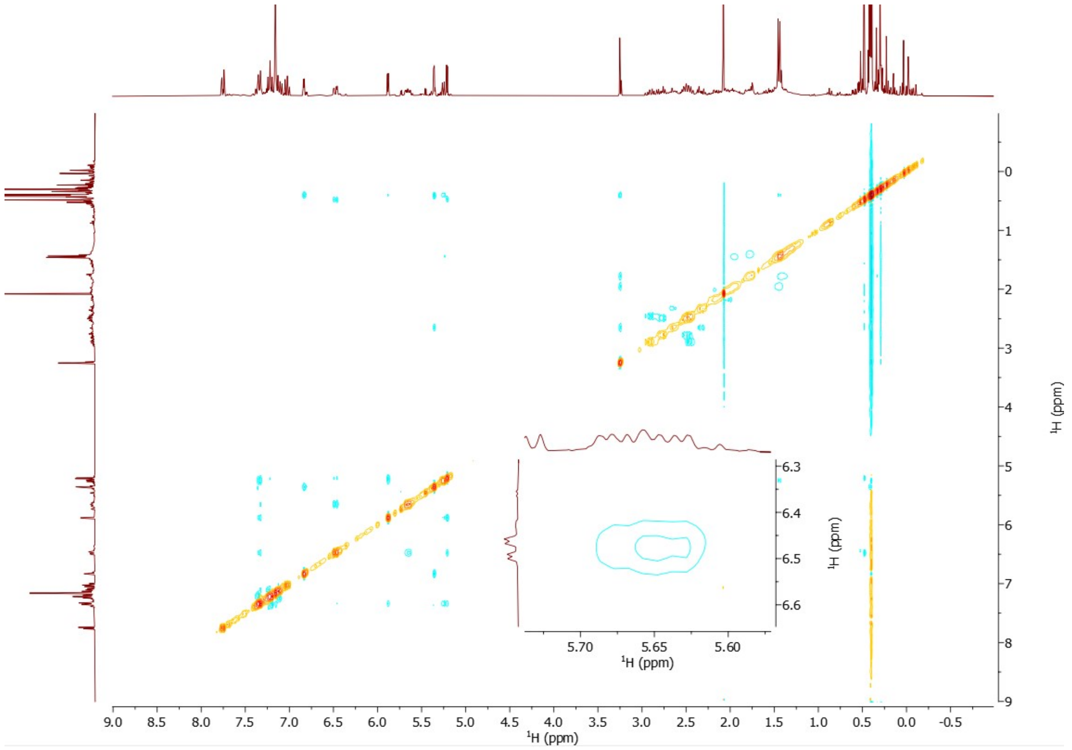

Figure S78. ^{1}H-^{1}H NOESY NMR spectrum of **17** (25 °C, benzene-d_6, 300.1 MHz).

6.13 NMR spectra of 18a

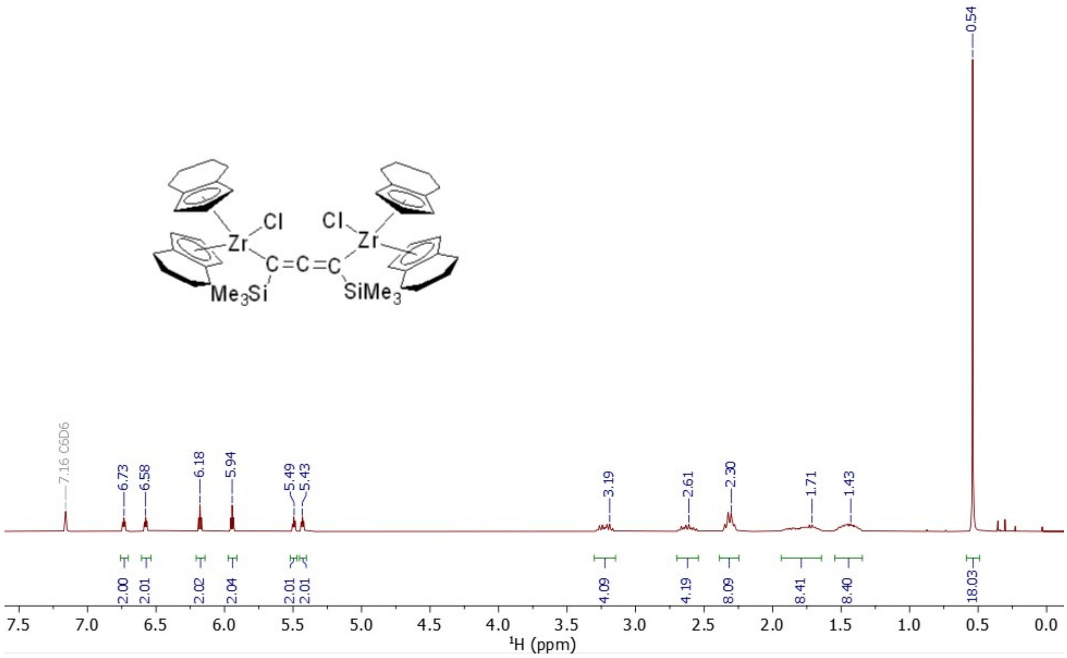

Figure S79. ^{1}H NMR spectrum of **18a** (25 °C, benzene-d_6, 300.2 MHz).

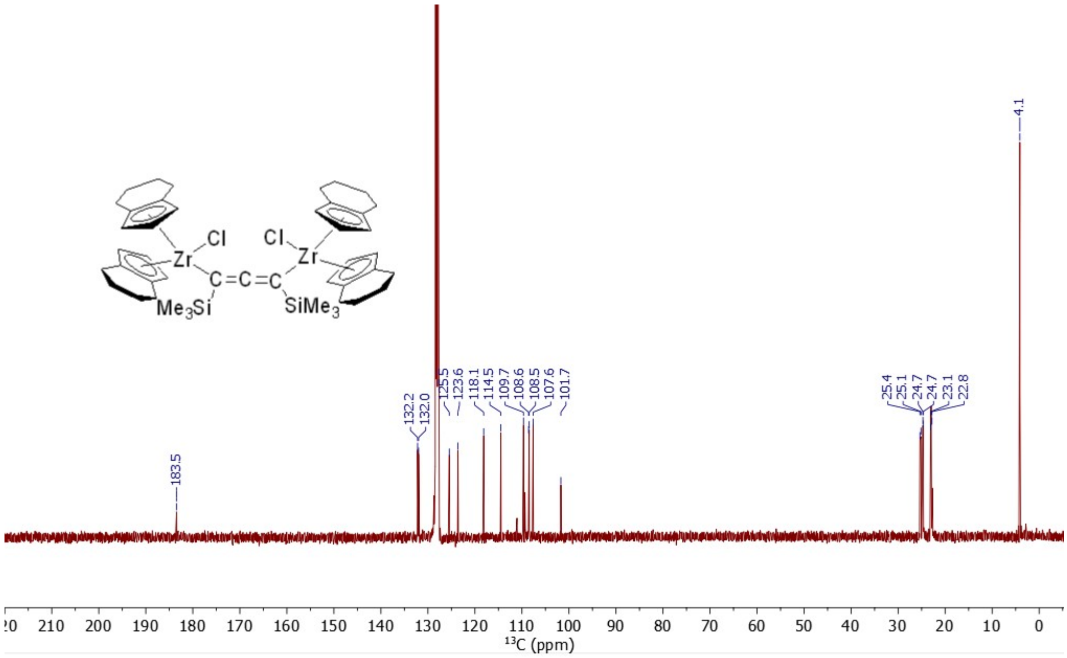

Figure S80. ^{13}C NMR spectrum of **18a** (25 °C, benzene-d_6, 100.6 MHz).

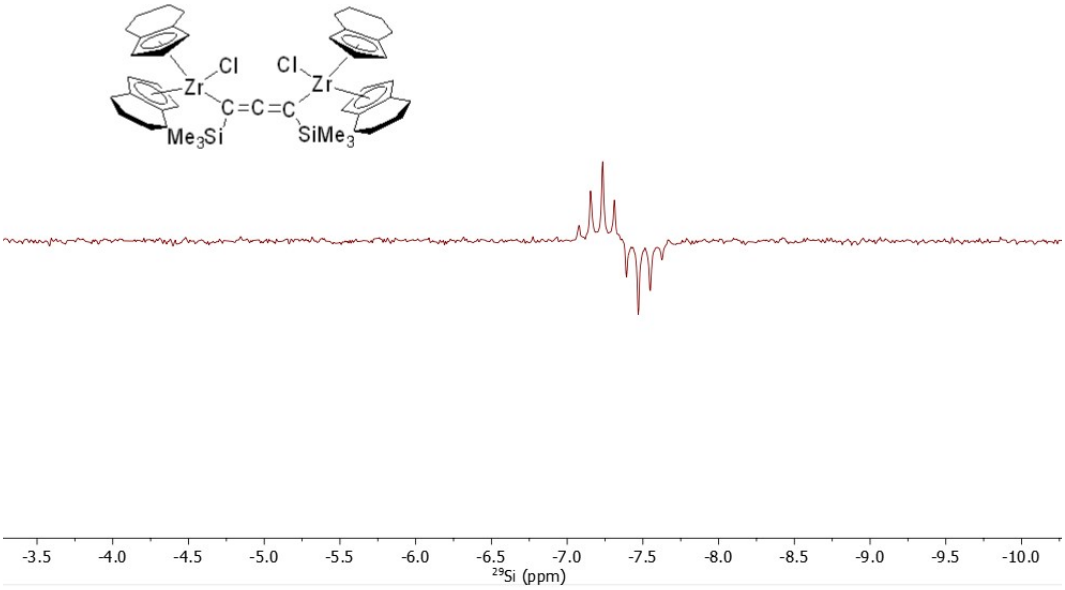

Figure S81. ^{29}Si INEPT NMR spectrum of **18a** (25 °C, benzene-d_6, 79.5 MHz).

6.14 NMR spectra of 18b

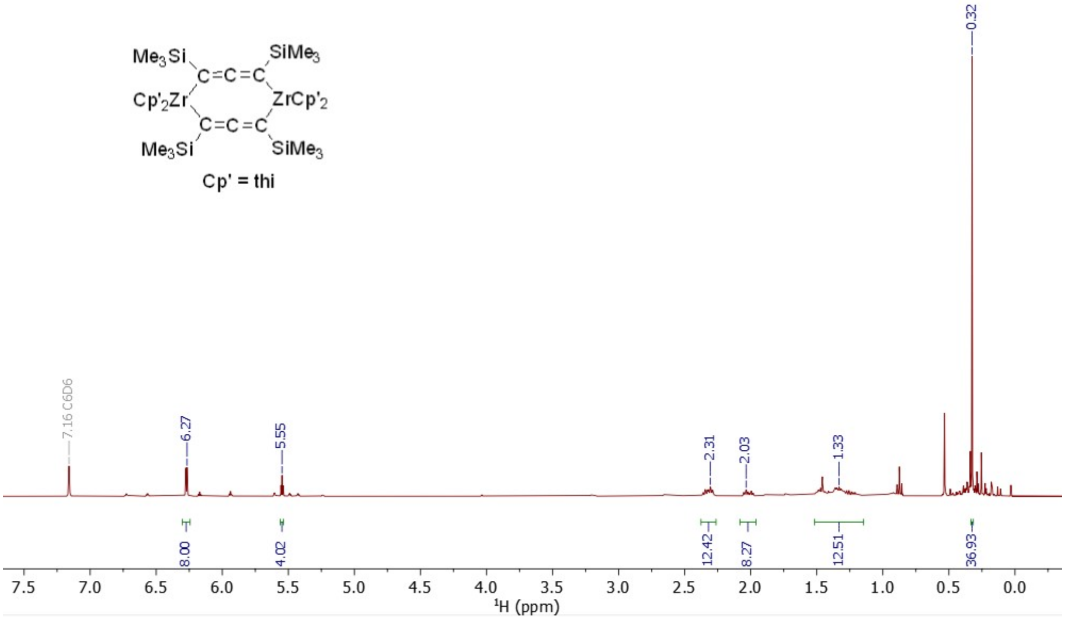

Figure S82. ^{1}H NMR spectrum of **18b** (containing **18a** as minor species; 25 °C, benzene-d_6, 400.1 MHz).

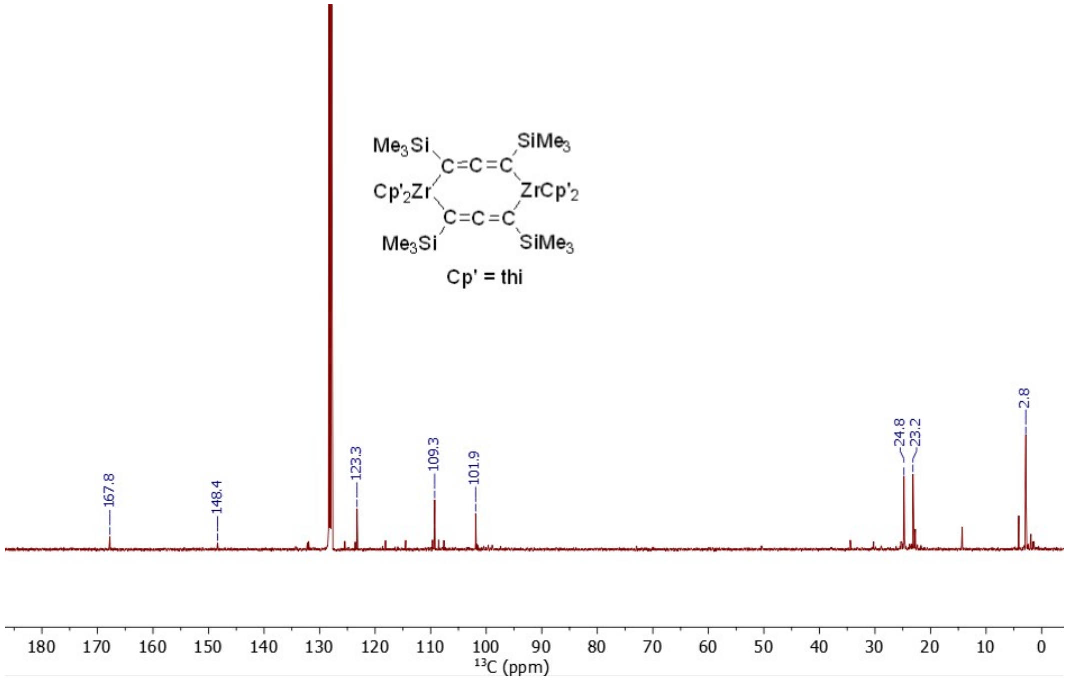

Figure S83. ^{13}C NMR spectrum of **18b** (containing **18a** as minor species; 25 °C, benzene-d_6, 100.6 MHz).

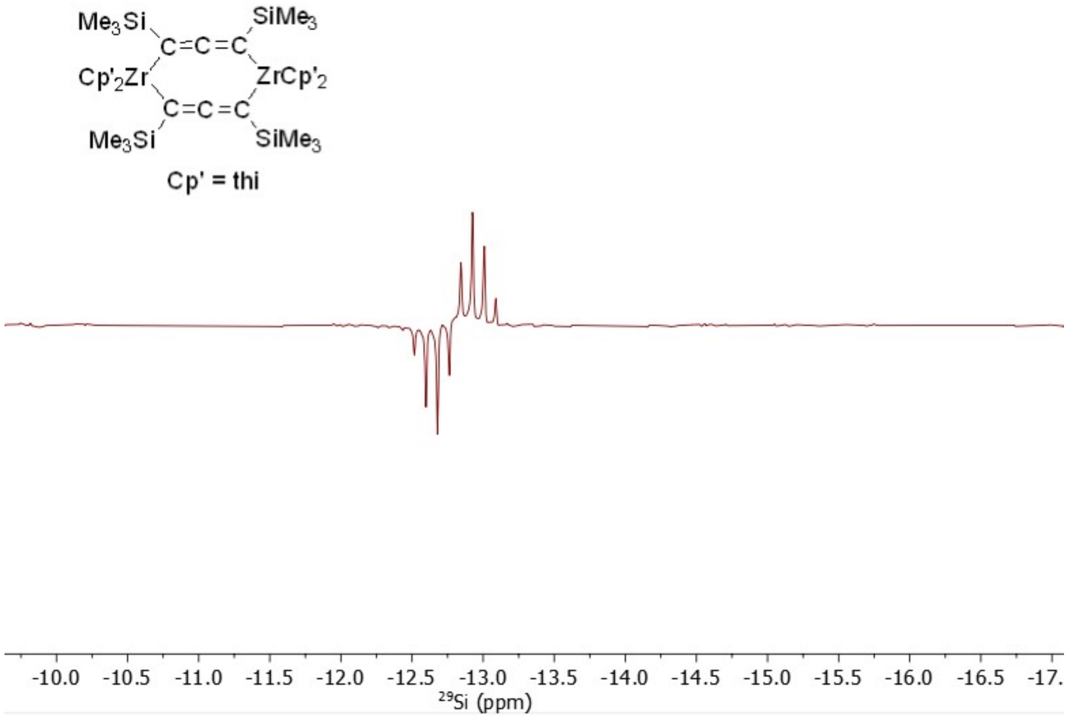

Figure S84. ^{29}Si INEPT NMR spectrum of **18b** (containing **18a** as minor species; 25 °C, benzene-d_6, 79.5 MHz).

6.15 NMR spectra of **19**

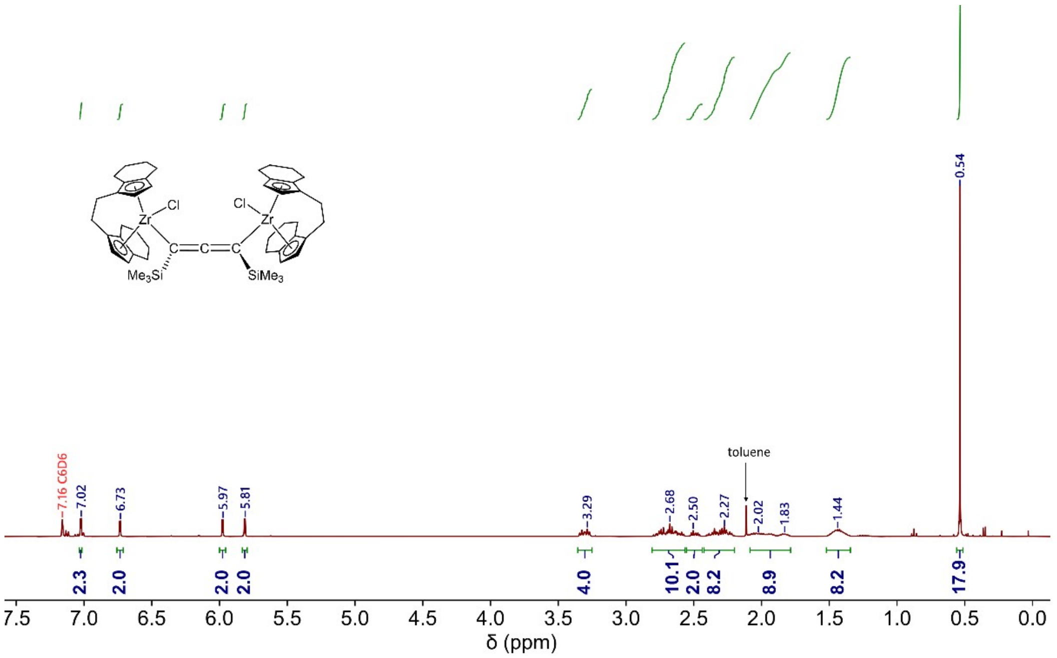

Figure S85. ^{1}H NMR spectrum of **19** (25 °C, benzene-d_6, 400.1 MHz).

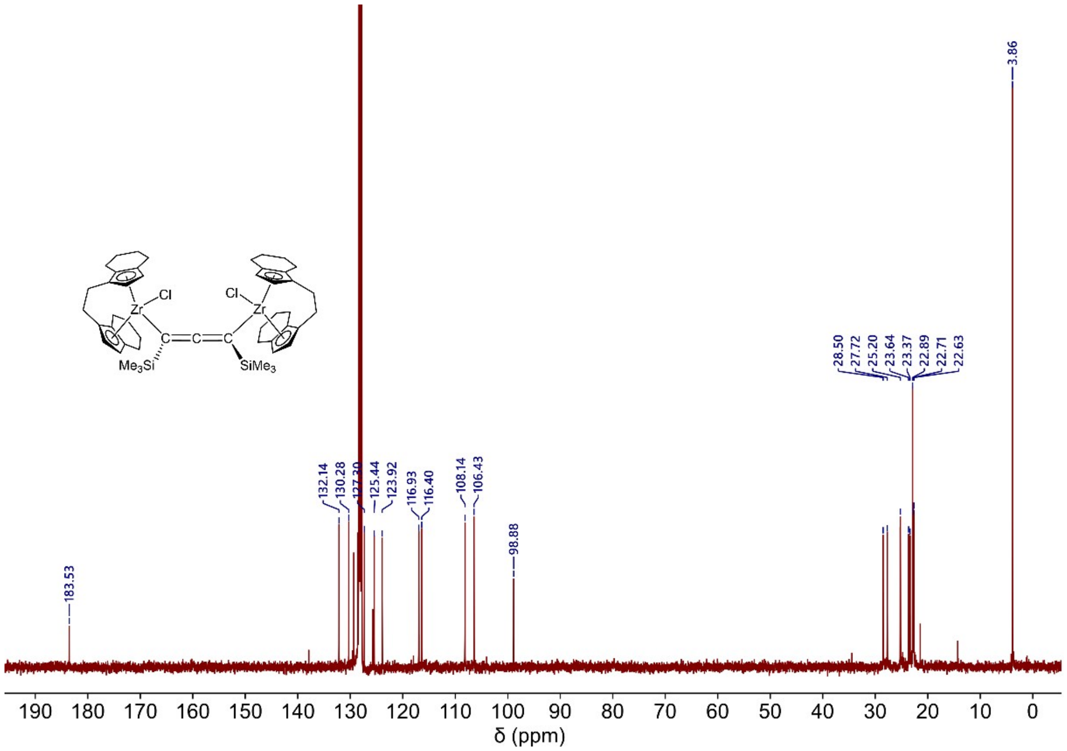

Figure S86. ^{13}C NMR spectrum of **19** (25 °C, benzene-d_6, 100.6 MHz).

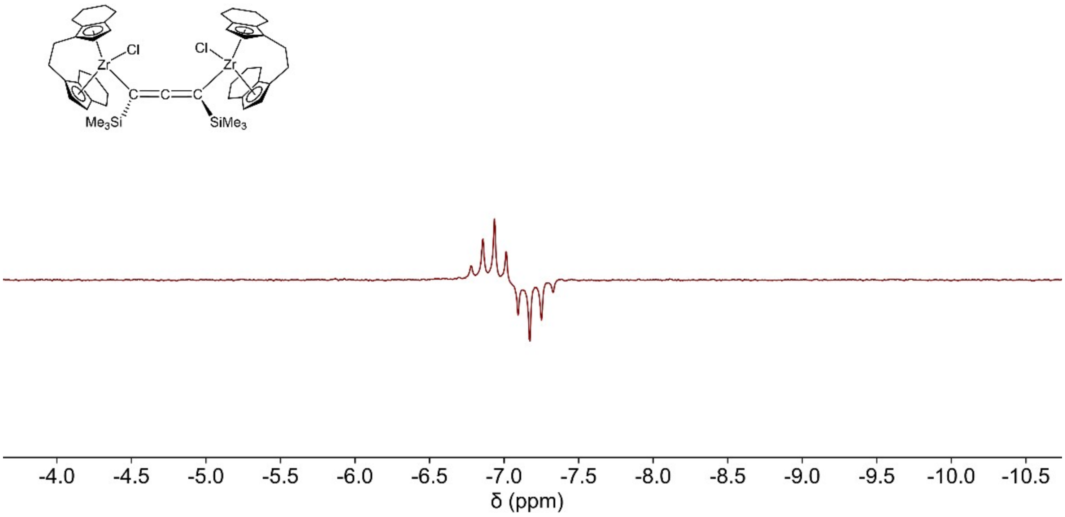

Figure S87. ^{29}Si INEPT NMR spectrum of **19** (25 °C, benzene-d_6, 79.5 MHz).

6.16 NMR spectra of 20

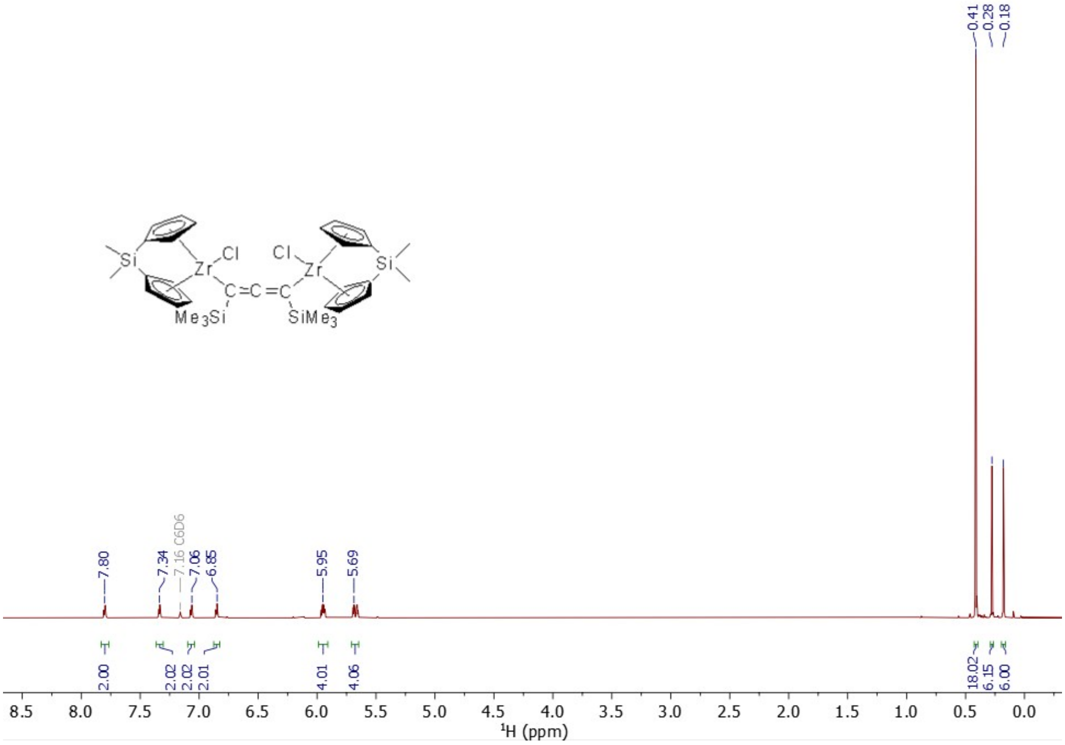

Figure S88. ^{1}H NMR spectrum of **20** (25 °C, benzene-d_6, 400.1 MHz).

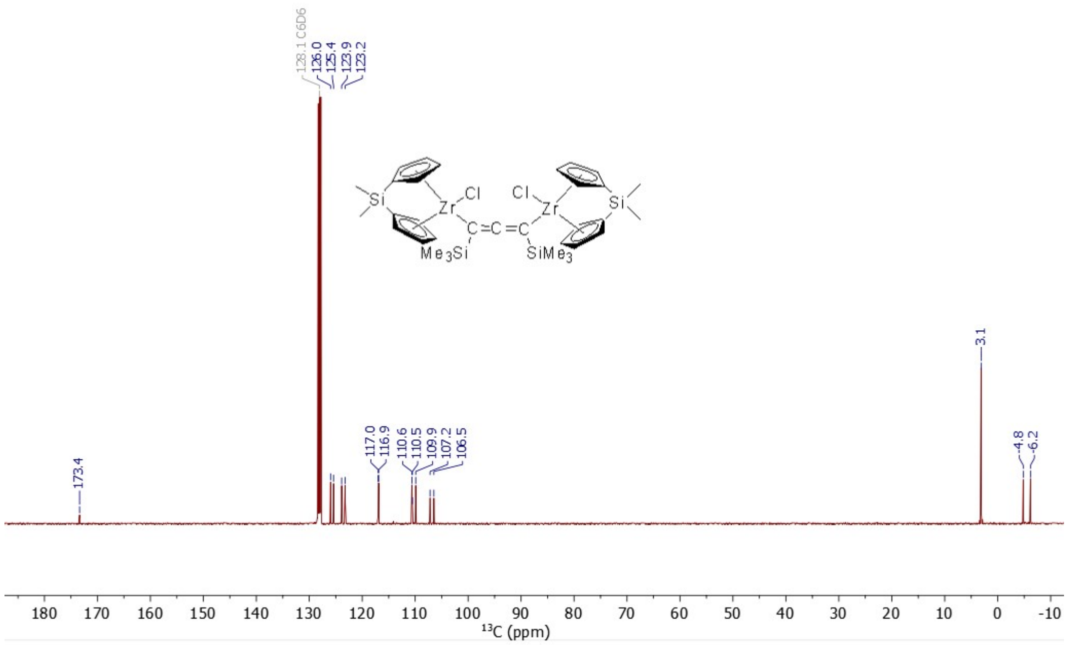

Figure S89. ^{13}C NMR spectrum of **20** (25 °C, benzene-d_6, 100.6 MHz).

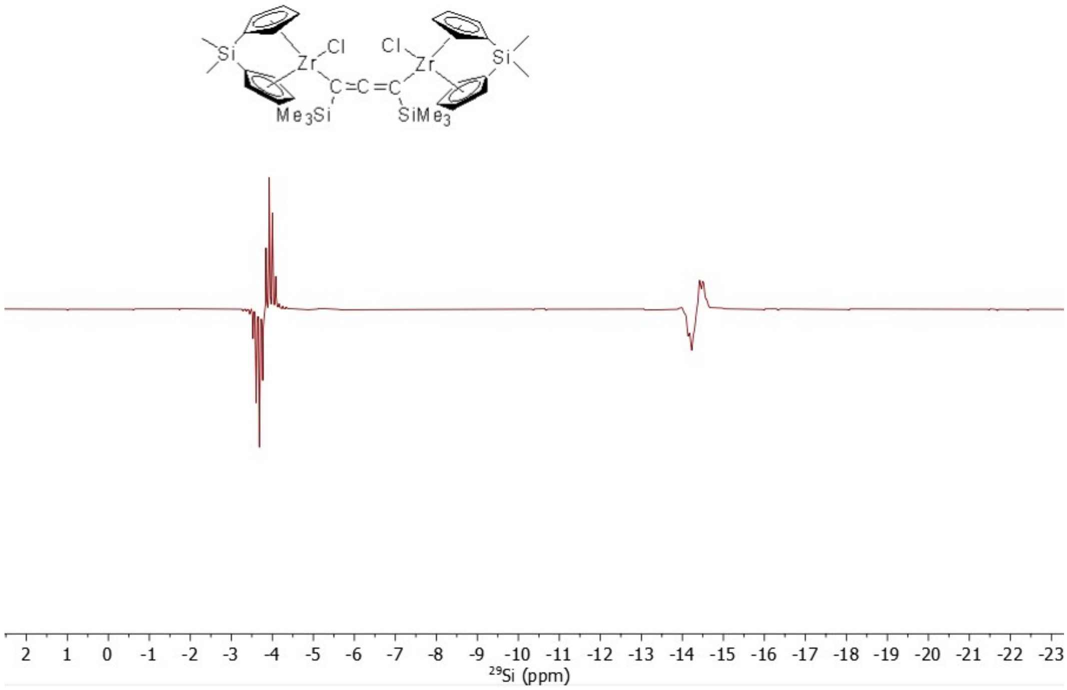

Figure S90. ^{29}Si INEPT NMR spectrum of **20** (25 °C, benzene-d_6, 79.5 MHz).

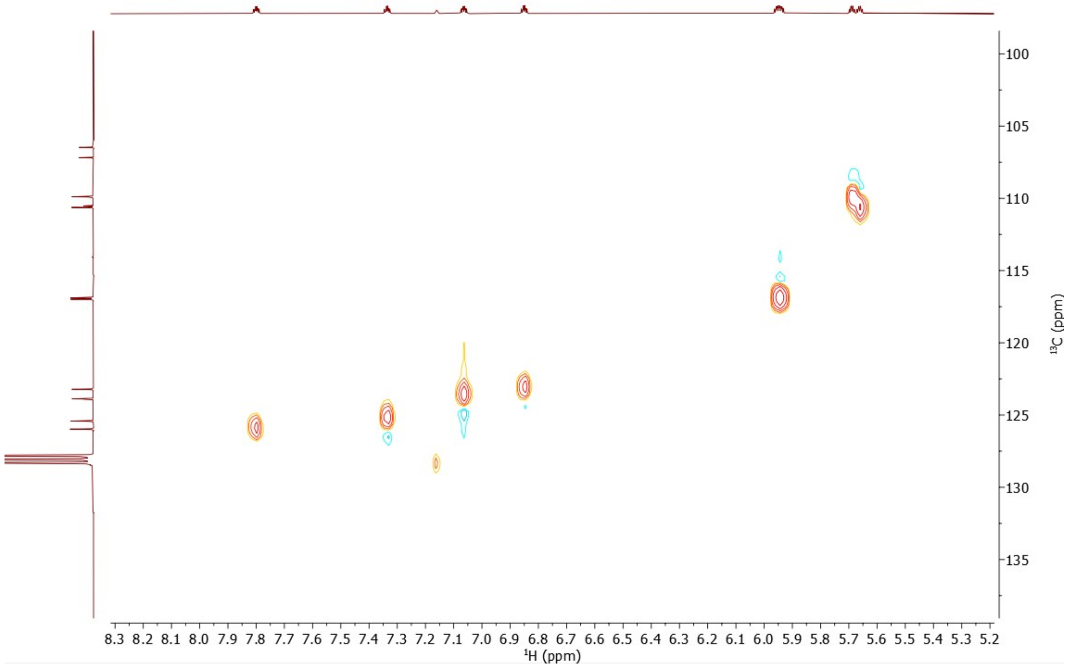

Figure S91. ^{1}H-^{13}C HSQC NMR spectrum of **20** (25 °C, benzene-d_6, 400.1 MHz, low-field region).

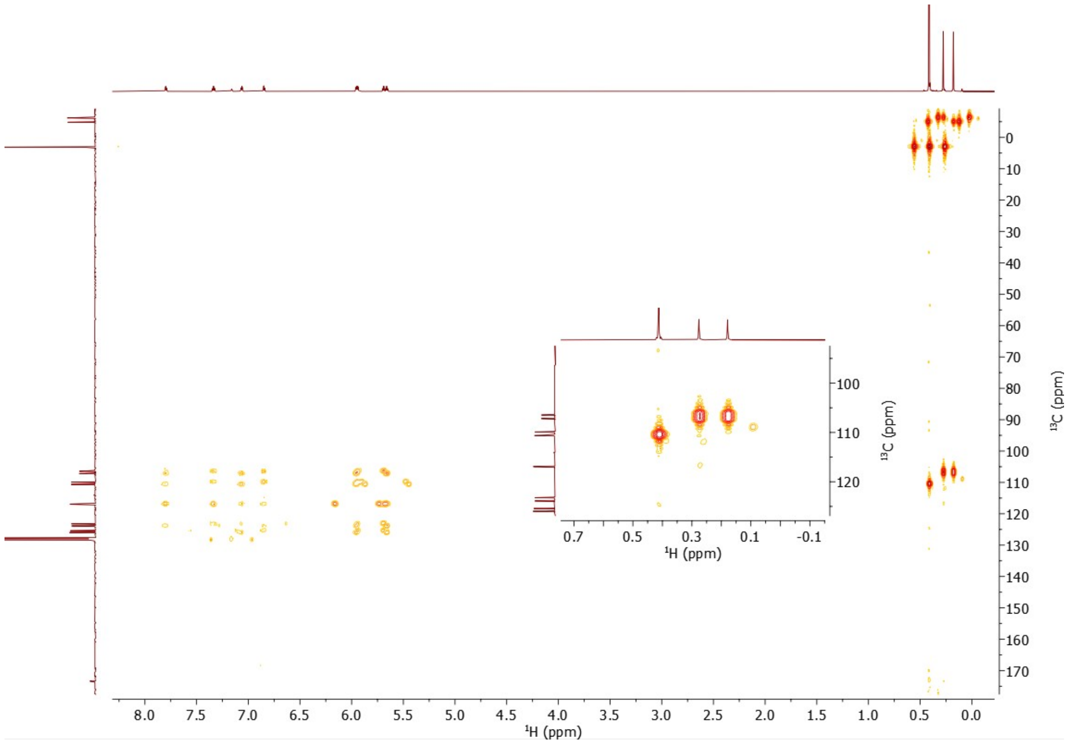

Figure S92. ^{1}H-^{13}C HMBC NMR spectrum of **20** (25 °C, benzene-d_6, 400.1 MHz).

7. Vibrational Spectra

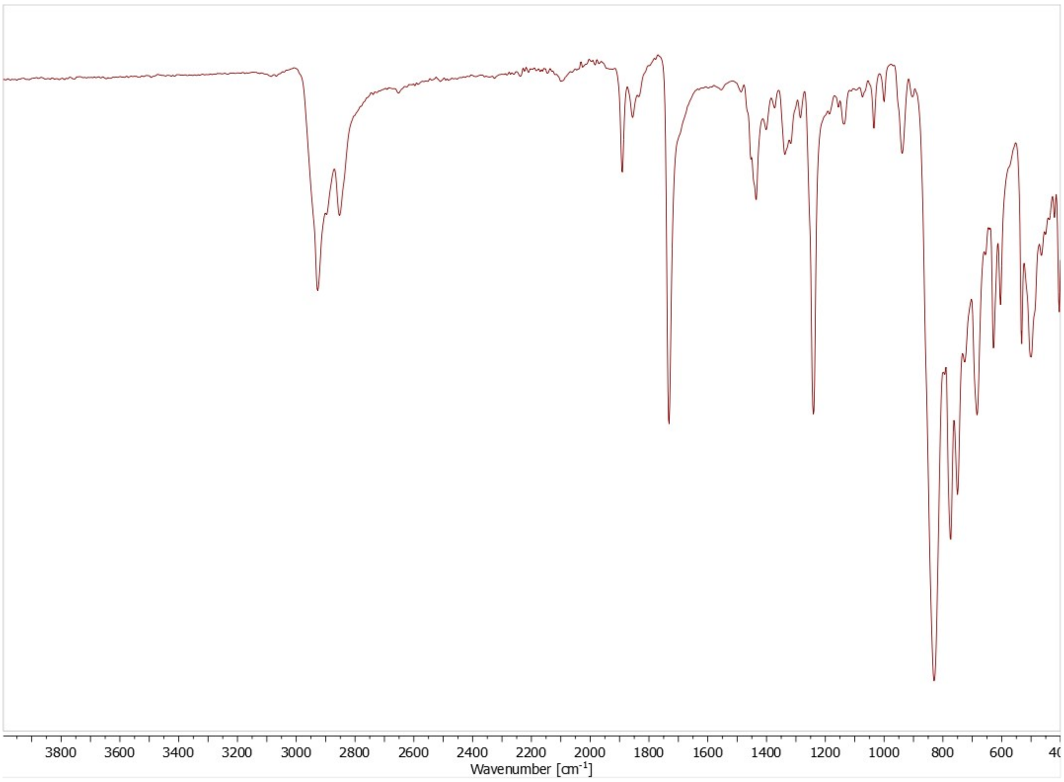

Figure S93. IR spectrum of **2a**.

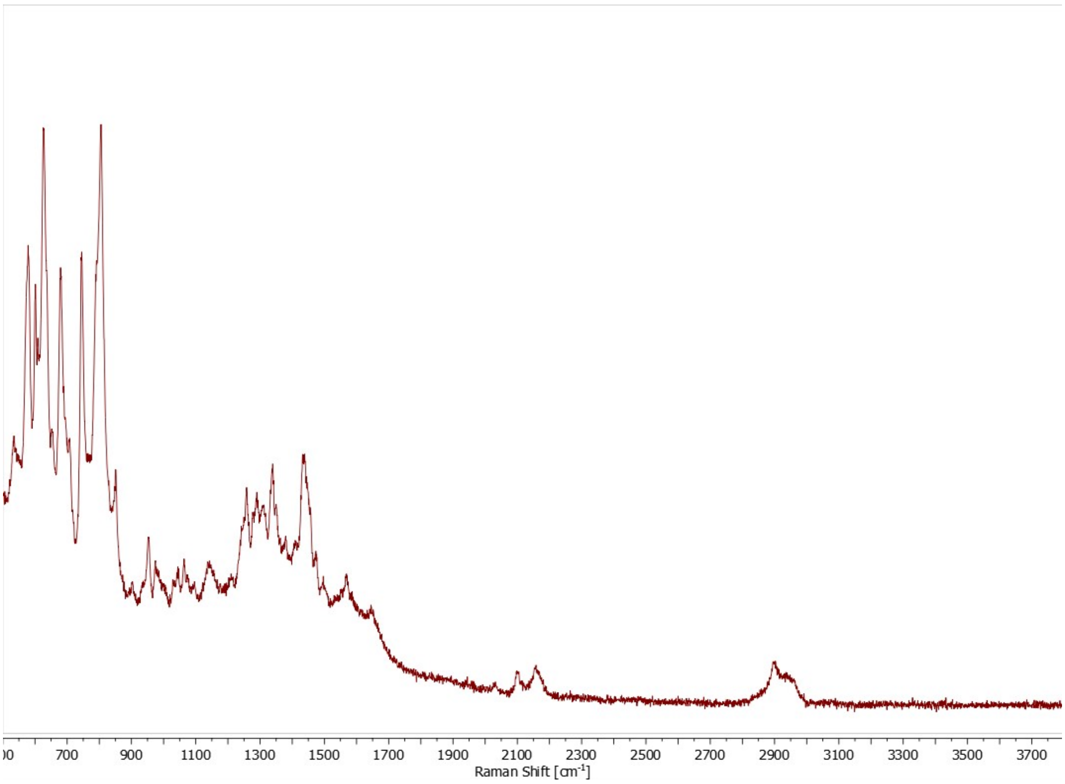

Figure S94. Raman spectrum of **2a**.

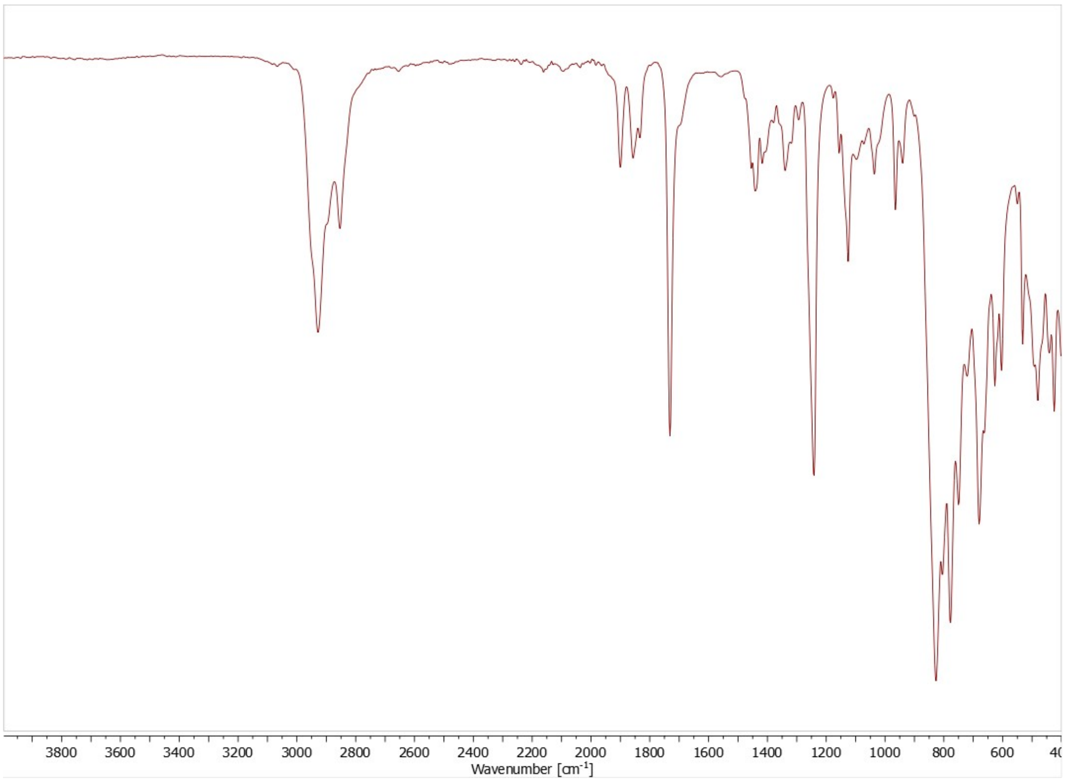

Figure S95. IR spectrum of **2b**.

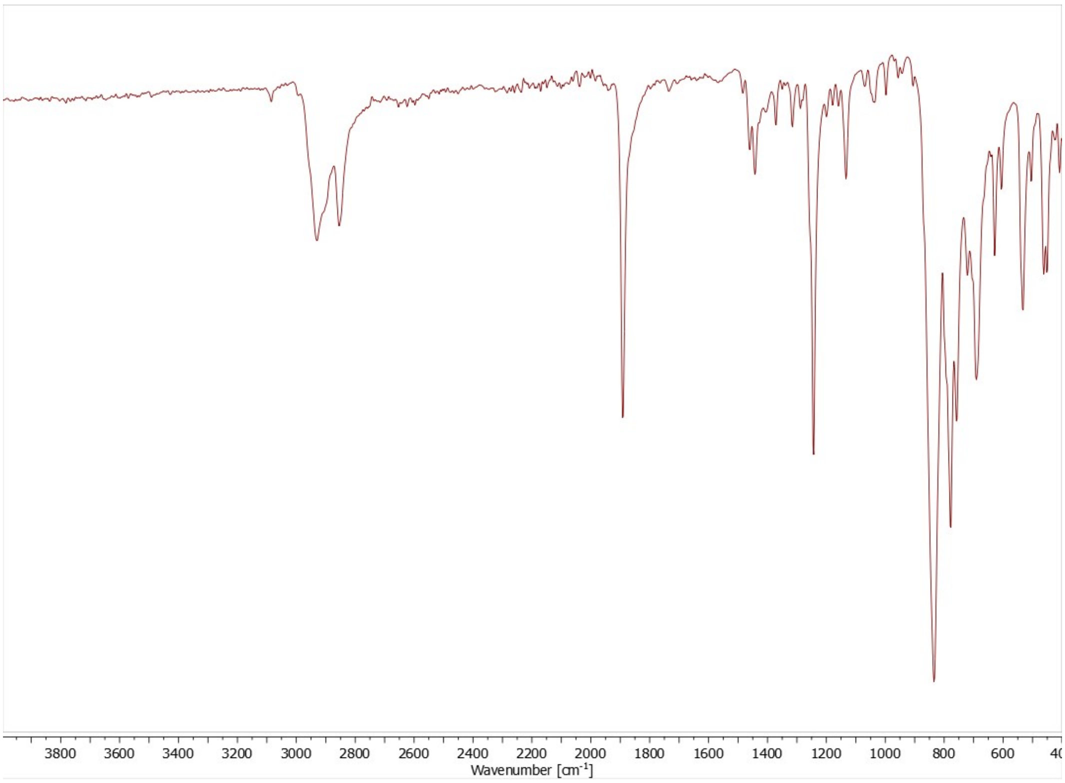

Figure S96. IR spectrum of **3a**.

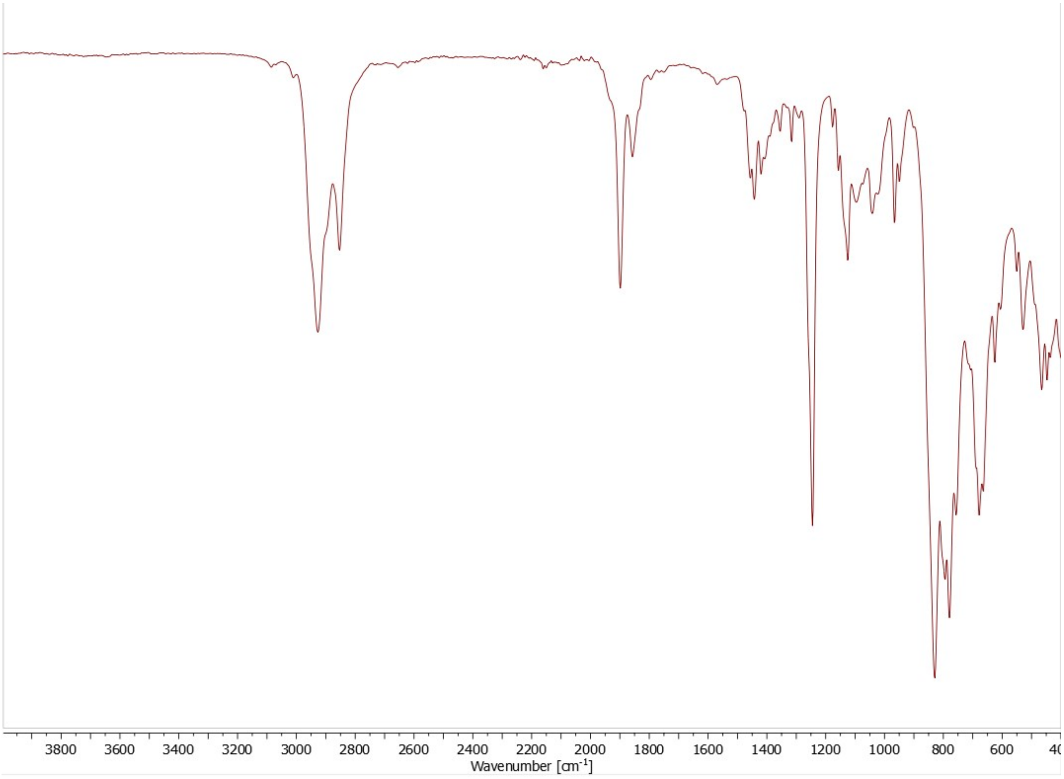

Figure S97. IR spectrum of **3b**.

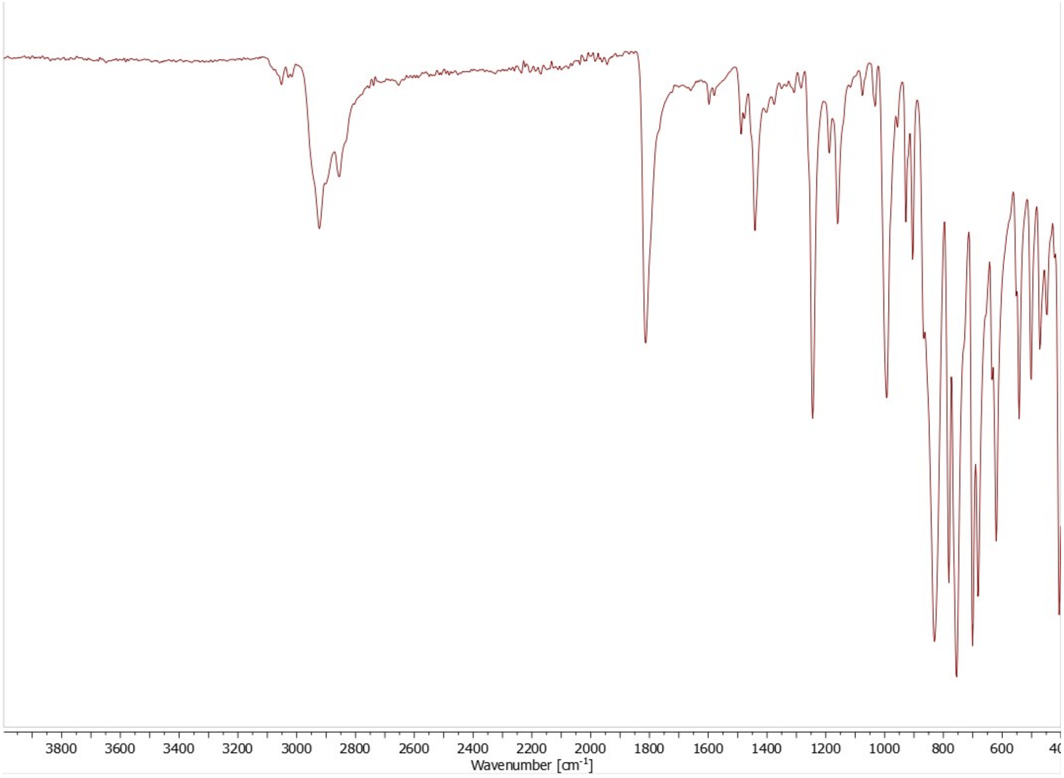

Figure S98. IR spectrum of **4**.

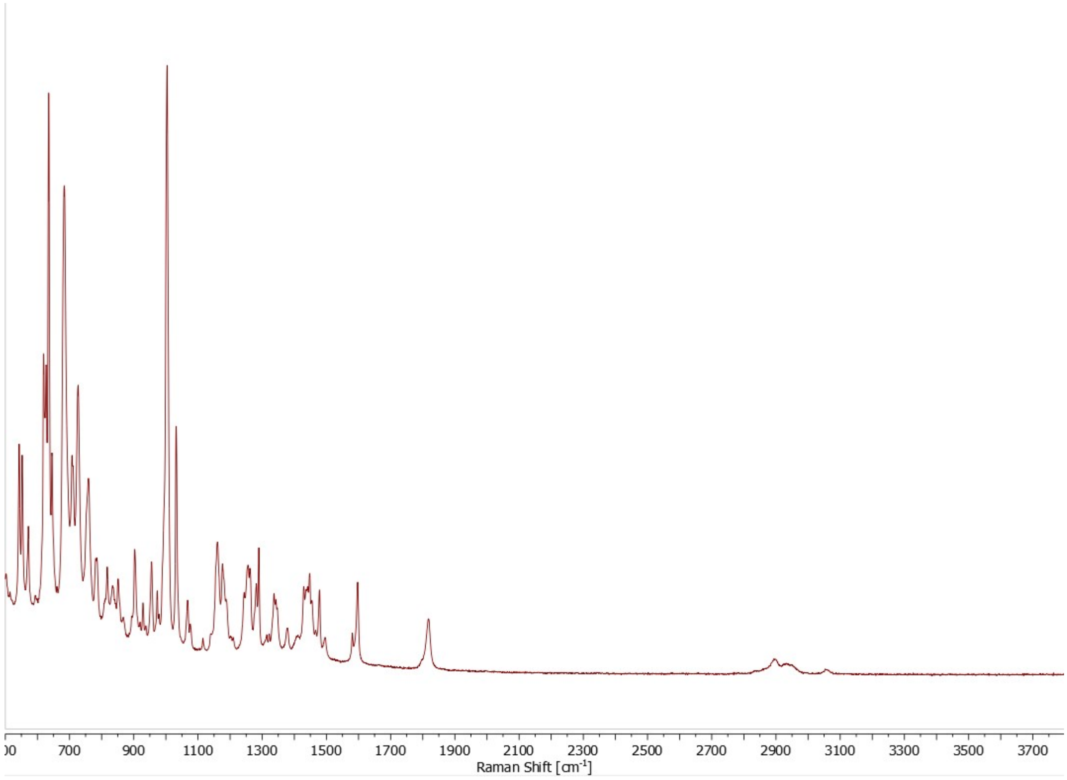

Figure S99. Raman spectrum of **4**.

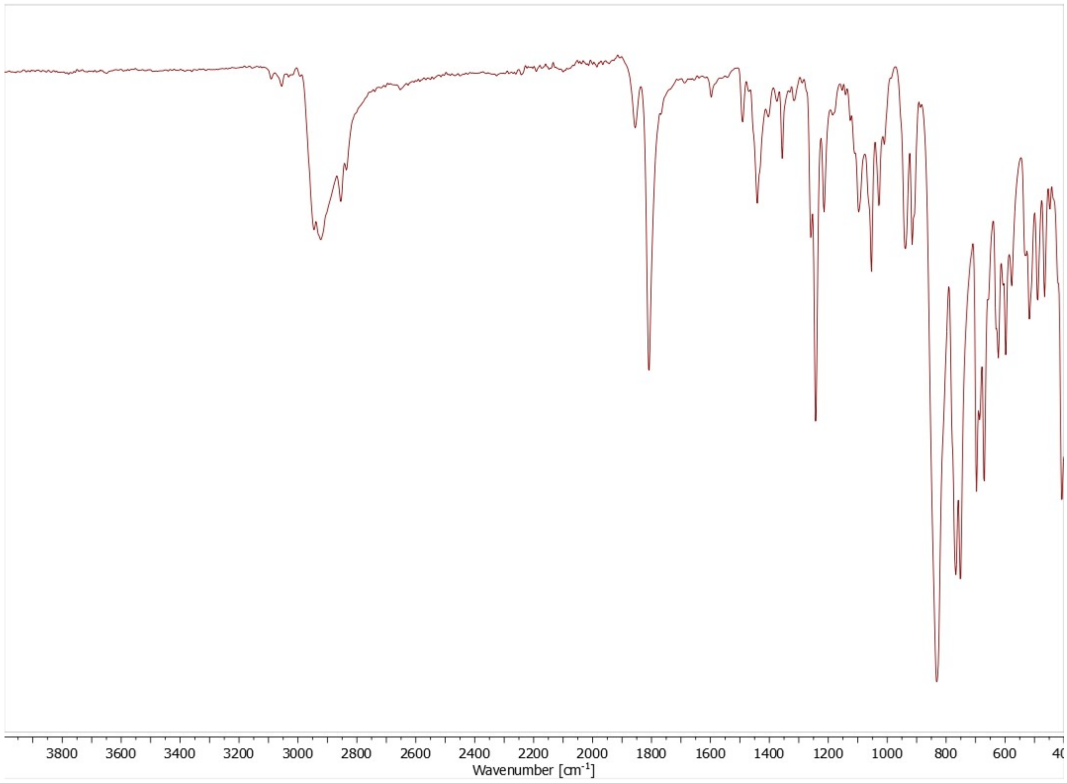

Figure S100. IR spectrum of **6**.

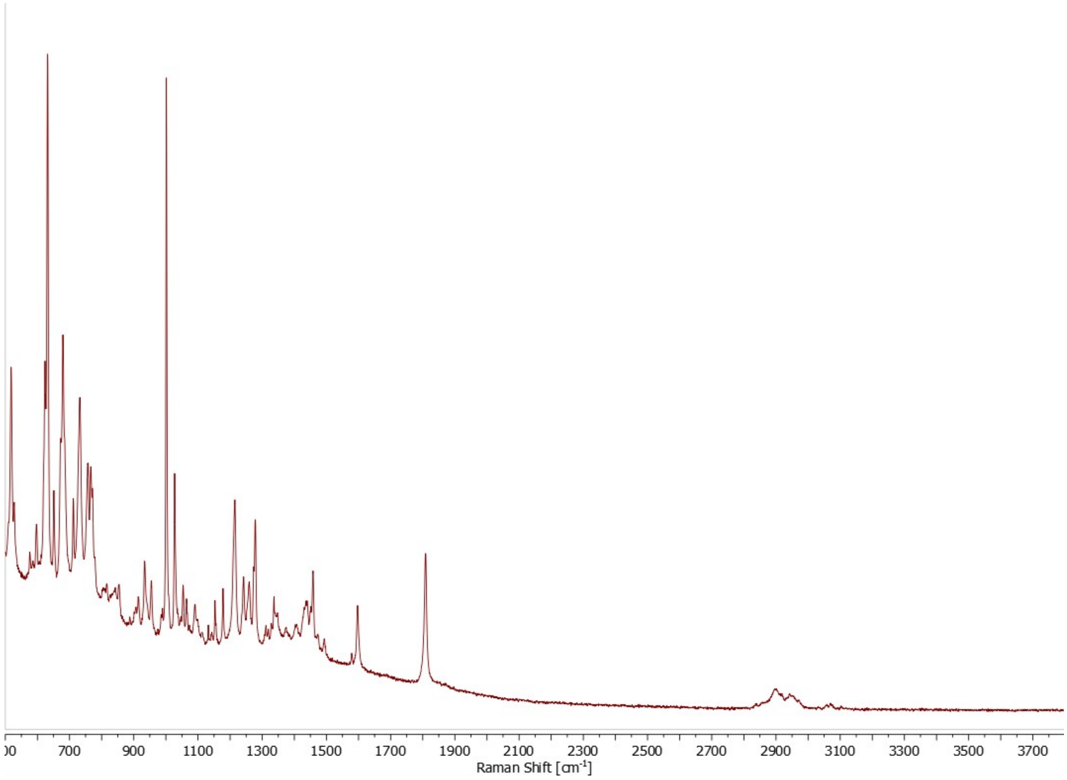

Figure S101. Raman spectrum of **6**.

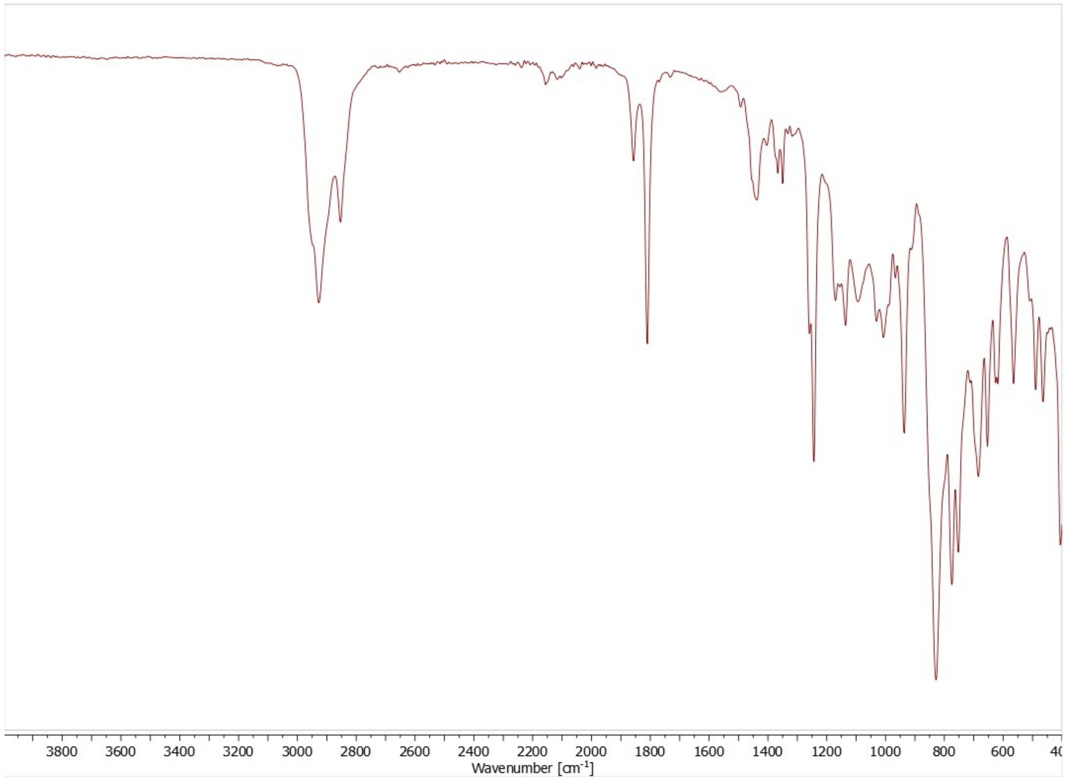

Figure S102. IR spectrum of **8**.

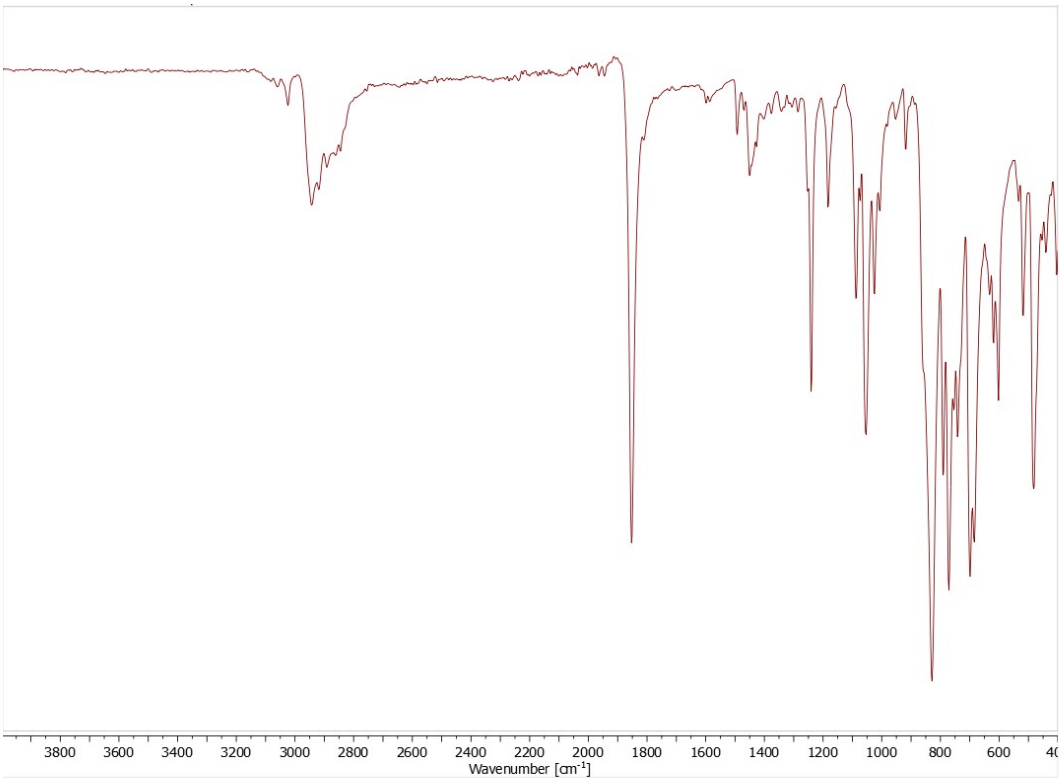

Figure S103. IR spectrum of **13**.

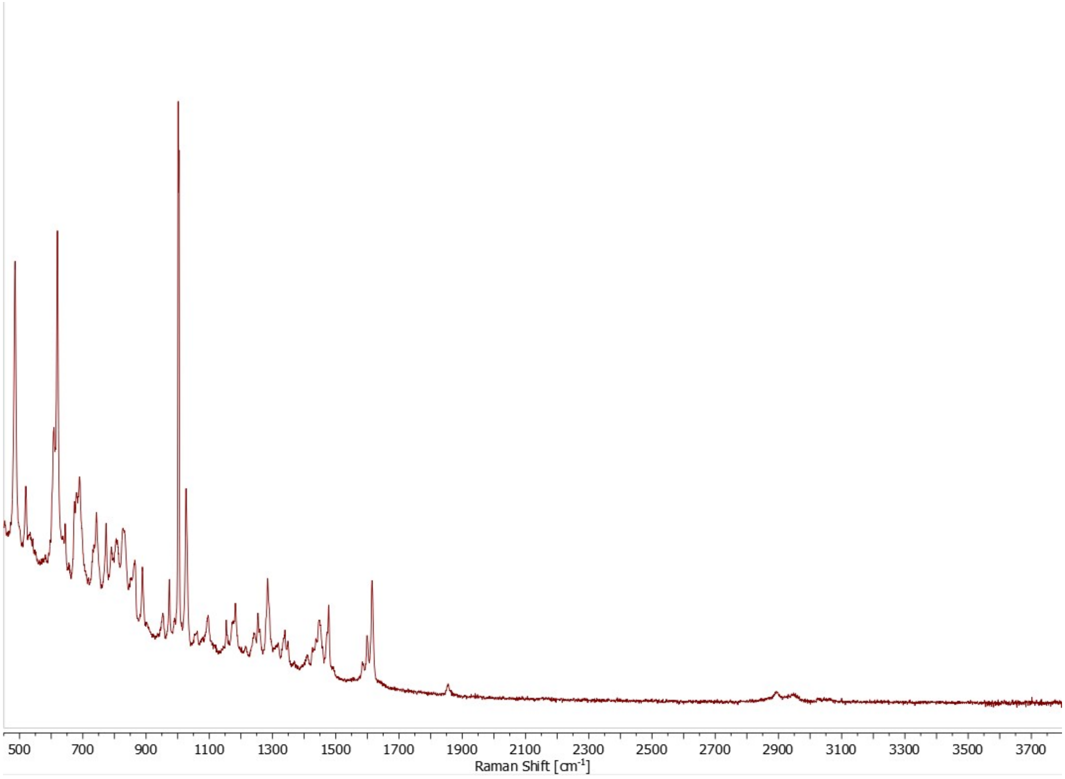

Figure S104. Raman spectrum of **13**.

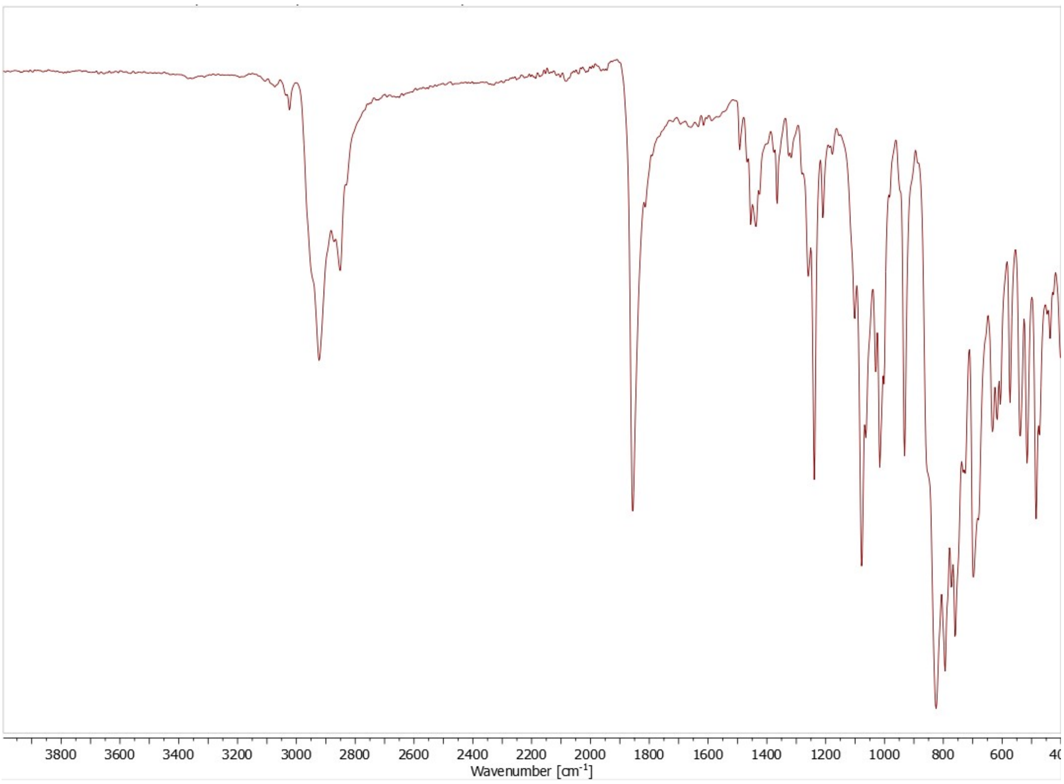

Figure S105. IR spectrum of **14**.

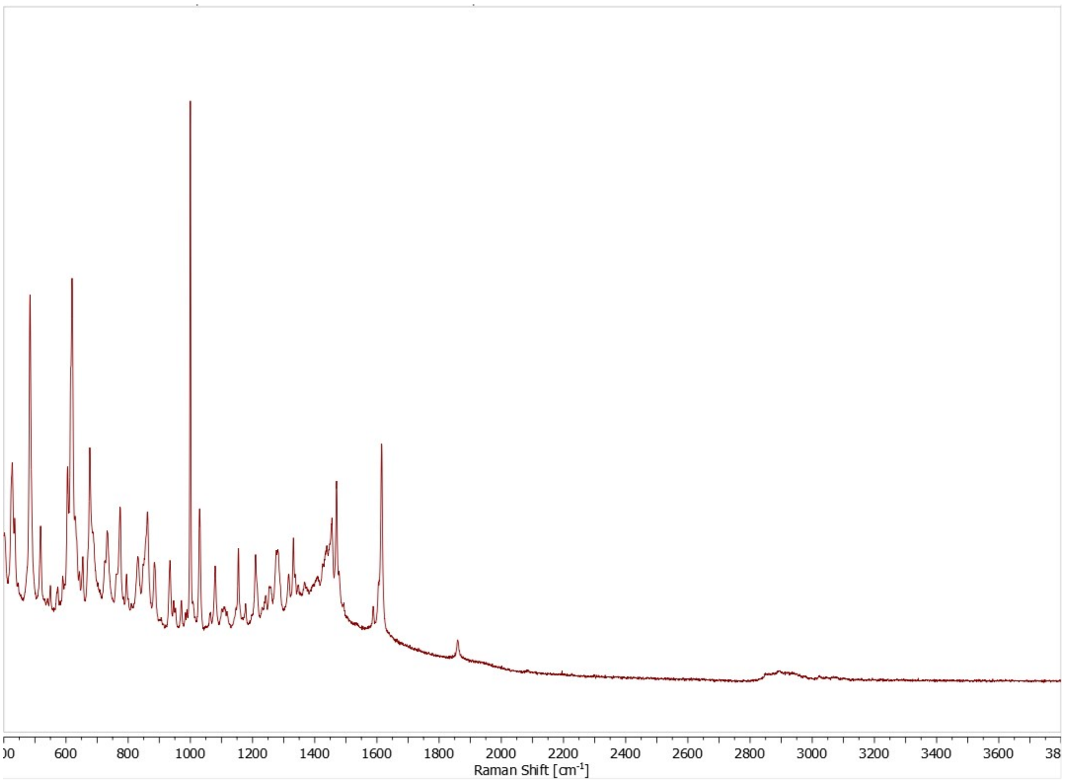

Figure S106. Raman spectrum of **14**.

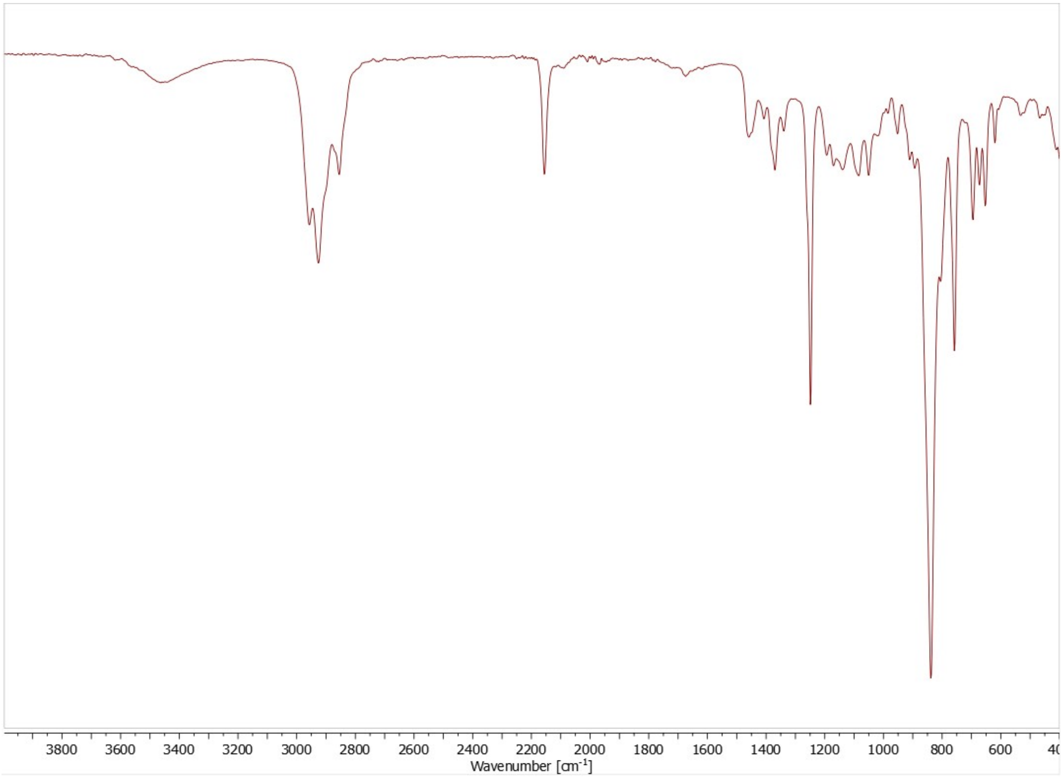

Figure S107. IR spectrum of **15**.

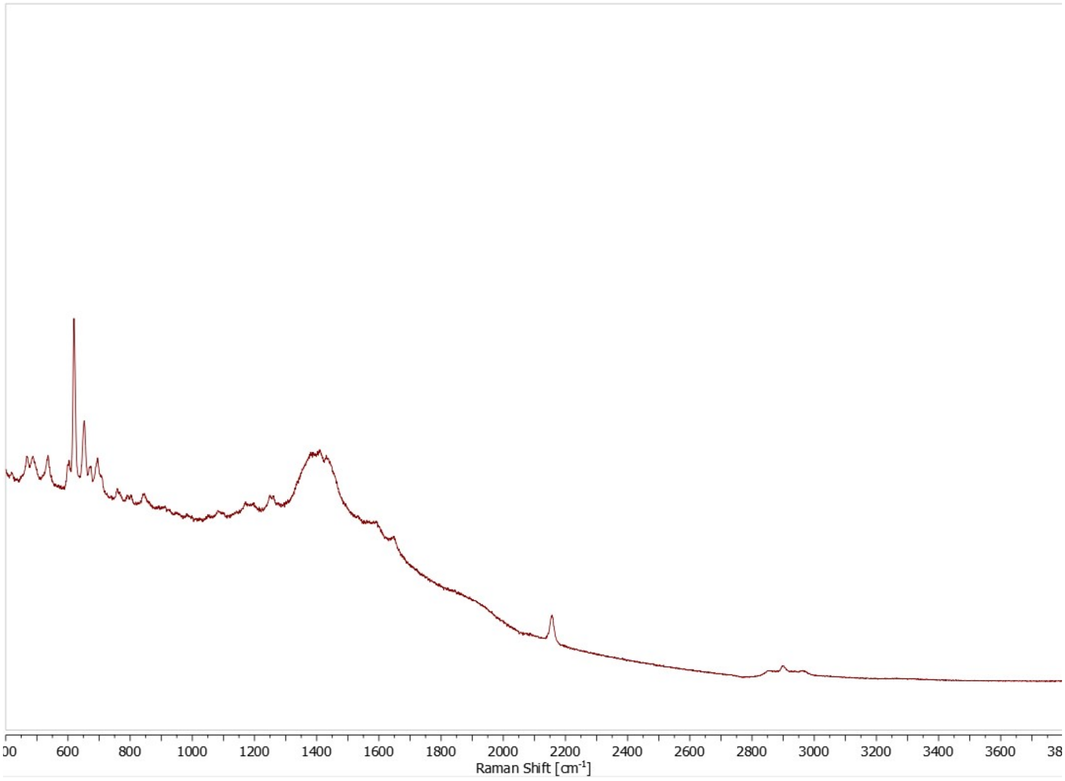

Figure S108. Raman spectrum of **15**.

8. Computational Details

8.1 General remarks

Computations were carried out using *Gaussian16*[8] and the standalone version of *NBO 6.0*.[9]

To understand the formation of complex *rac*-(ebthi)*Zr($Me_3SiCHCCSiMe_3$) (**3a**) we first evaluated the isodesmic exchange reaction to give the 1-zirconacyclobuta-2,3-diene (**2a**) developed in this context using quantum mechanical analysis. Next, we have analyzed different possible reaction pathways for the formation of **3a**. Therefore, we have optimized the real-size molecules using the hybrid density functional method B3LYP,[10,11] in combination with the basis set def2svpp,[12] and the empirical dispersion correction GD3BJ[13] (notation: B3LYP/GD3BJ/def2svpp). We were thus able to identify a probable path of C-H activation. We subsequently recalculated it using B3LYP/GD3BJ/def2tzvp. Finally, we performed all the bond analysis calculations at this higher level. Vibrational frequencies were also computed, to include zero-point vibrational energies in thermodynamic parameters and to characterize all structures as minima on the potential energy surface. To confirm the accuracy of the calculated transition states (TS), we performed intrinsic reaction coordinate (IRC) analysis on these structures. QT-AIM, ELF and Wiberg bond index calculations/visualization were performed using MultiWfn 3.5 employing *Gaussian16* formatted checkpoint files.[14] For the visualization of 3D-quantum chemical results we used GaussView 6.1.1.[15] In addition to the Supporting Information, we provide a multi-structure xyz file with all calculated molecules.

For a better understanding and a more intuitive view of the calculated 3D structures, we strongly recommend using this file e.g. with the free program MERCURY.[16]

Please note that all computations were carried out for single, isolated molecules in the gas phase (ideal gas approximation). There may well be significant differences between gas phase and condensed phase.

8.2 Thermochemistry

In this chapter we summarize the results of our thermodynamic calculations, which were performed on the B3LYP/GD3BJ/def2svpp level of theory as described above.

8.2.1 Formation of 1-zirconacyclobuta-2,3-dienes

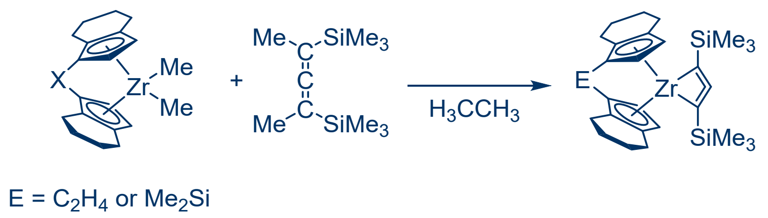

Scheme S1. Calculated isodesmic exchange reaction for the formation of 1-zirconacyclobuta-2,3-dienes.

Table S3. Calculated isodesmic exchange reaction for the formation of 1-zirconacyclobuta-2,3-dienes (B3LYP/GD3BJ/def2svpp).

Main Product	$\Delta_R H$ [kJ·mol^{-1}]	$\Delta_R G$ [kJ·mol^{-1}]	$\Delta_R H$ [kcal·mol^{-1}]	$\Delta_R G$ [kcal·mol^{-1}]
rac-(ebthi)Zr($Me_3SiCCCSiMe_3$)	-26.6	-53.5	-6.4	-12.8
rac-$Me_2Si(thi)_2Zr(Me_3SiCCCSiMe_3)$	-25.0	-49.6	-6.0	-11.9

8.2.2 C-H activation

In a first set of calculations, we investigated the thermodynamic data for the experimentally observed C-H activation of the thi* ligand system.

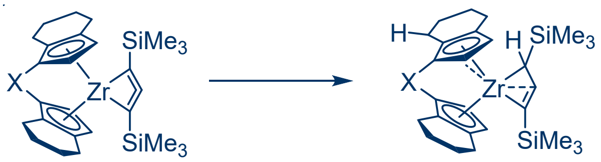

Scheme S2. Calculated reaction sequence of C-H activation.

Table S4. Calculated thermodynamic data for the formation of C-H activation products (B3LYP/GD3BJ/def2svpp).

Main Product	$\Delta_R H$ [kJ·mol^{-1}]	$\Delta_R G$ [kJ·mol^{-1}]	$\Delta_R H$ [kcal·mol^{-1}]	$\Delta_R G$ [kcal·mol^{-1}]
rac-(ebthi)*Zr($Me_3SiCHCCSiMe_3$)	-9.0	-0.2	-2.2	-0.1
rac-$Me_2Si(thi)_2$*Zr($Me_3SiCHCCSiMe_3$)	-17.3	-14.1	-4.1	-3.4

Further detailed mechanistic considerations were first made for *rac*-(ebthi)Zr($Me_3SiCCCSiMe_3$) (**2a**). Although only one C-H activated species was found in the final thermodynamically stable product, it could theoretically have formed from a different intermediate. To verify this, Gibbs free reaction enthalpies ($\Delta_R G$) were first calculated for all derivatives that also carried a CH_2 group and thus could formally undergo the same C_{CH2}-H activation. These hypothetical isomers could transform into the thermodynamic final product (iso1) via proton migration along the ligand scaffold. Scheme S3 shows the identifiers of the activated isomers.

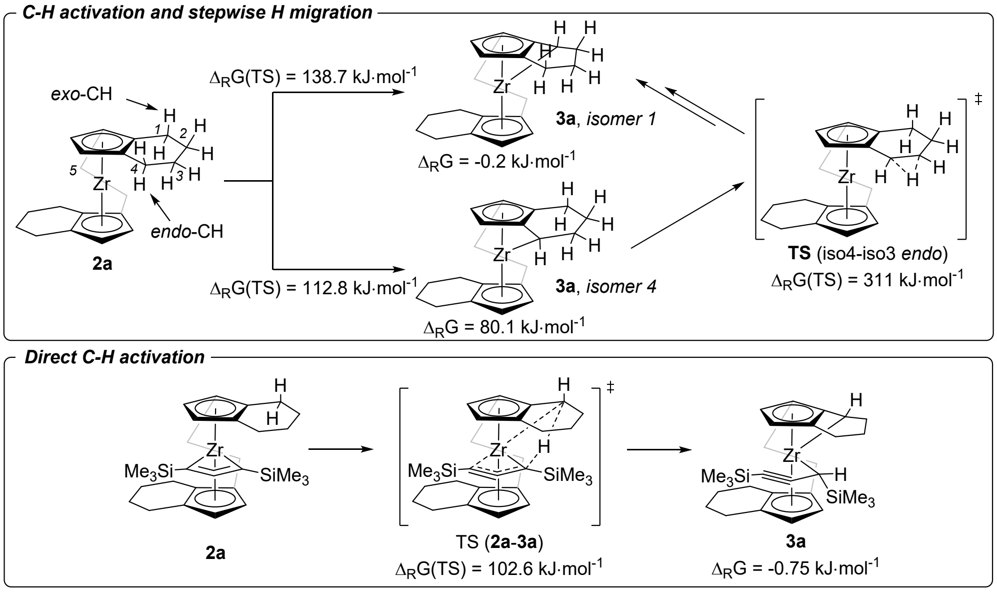

Scheme S3. Labelling schemes for the considered CH_2 activation isomers.

Table S5. Calculated thermodynamic data for the formation of formal CH activation products referred to complex **2a** (B3LYP/GD3BJ/def2svpp).

Main Product	Δ_RH [kJ·mol^{-1}]	Δ_RG [kJ·mol^{-1}]	Δ_RH [kcal·mol^{-1}]	Δ_RG [kcal·mol^{-1}]
rac-(ebthi)*Zr($Me_3SiCHCCSiMe_3$) iso1TS	131.9	138.7	31.5	33.2
rac-(ebthi)*Zr($Me_3SiCHCCSiMe_3$) iso1	-9.0	-0.2	-2.2	-0.1
rac-(ebthi)*Zr($Me_3SiCHCCSiMe_3$) iso2TS	141.2	151.8	33.7	36.3
rac-(ebthi)*Zr($Me_3SiCHCCSiMe_3$) iso2	32.1	38.0	7.7	9.1
rac-(ebthi)*Zr($Me_3SiCHCCSiMe_3$) iso3TS	142.4	153.7	34.0	36.7
rac-(ebthi)*Zr($Me_3SiCHCCSiMe_3$) iso3	34.7	39.5	8.3	9.4
rac-(ebthi)*Zr($Me_3SiCHCCSiMe_3$) iso4TS	107.4	112.8	25.7	27.0
rac-(ebthi)*Zr($Me_3SiCHCCSiMe_3$) iso4	81.5	80.1	19.5	19.1
rac-(ebthi)*Zr($Me_3SiCHCCSiMe_3$) iso5	127.9	129.9	30.6	31.0

These calculations show that only the experimentally found isomer 1 displays an exergonic Gibbs free reaction enthalpy. However, the activation energy for the transition state to isomer 4 turned out to be 6 kcal·mol^{-1} lower than the TS to isomer 1. For this reason, we have next looked at two possible paths of H migration from position 1 to position 4. Neither migration of the "outer/exo" protons nor that of the protons facing the metal “endo” show transition states which would support this concept.

Table S6. Calculated thermodynamic data for the two different considered H migration path along the ligand (B3LYP/GD3BJ/def2svpp).

Main Product	$\Delta_R H$ [kJ·mol^{-1}]	$\Delta_R G$ [kJ·mol^{-1}]	$\Delta_R H$ [kcal·mol^{-1}]	$\Delta_R G$ [kcal·mol^{-1}]
rac-(ebthi)*Zr($Me_3SiCHCCSiMe_3$) TSiso2to1 exo	273.3	281.2	65.3	67.2
rac-(ebthi)*Zr($Me_3SiCHCCSiMe_3$) TSiso3to2 exo	358.7	367.4	85.7	87.8
rac-(ebthi)*Zr($Me_3SiCHCCSiMe_3$) TSiso4to3 exo	303.8	302.6	72.6	72.3
rac-(ebthi)*Zr($Me_3SiCHCCSiMe_3$) TSiso2to1 endo	252.6	257.6	60.4	61.6
rac-(ebthi)*Zr($Me_3SiCHCCSiMe_3$) TSiso3to2 endo	Even after 5 different attempts, no TS was found.			
rac-(ebthi)*Zr($Me_3SiCHCCSiMe_3$) TSiso4to3 endo	311.6	310.6	74.5	74.2

As these calculations clearly exclude this mode of C-H activation, direct C-H activation was next calculated using the larger basis set def2tzvp.

Table S7. Calculated thermodynamic data for the formation of C-H activation products referred to complexes **2a**, **2b**, and Ti complex **A** (B3LYP/GD3BJ/def2tzvp).

Main Product	$\Delta_R H$ [kJ·mol^{-1}]	$\Delta_R G$ [kJ·mol^{-1}]	$\Delta_R H$ [kcal·mol^{-1}]	$\Delta_R G$ [kcal·mol^{-1}]
rac-(ebthi)Zr($Me_3SiCCCSiMe_3$)	-27.83	-50.60	-6.65	-12.09
rac-(ebthi)*Zr($Me_3SiCHCCSiMe_3$) iso1_TS	107.34	102.61	25.66	24.52
rac-(ebthi)*Zr($Me_3SiCHCCSiMe_3$) iso1	-7.32	-0.75	-1.75	-0.18
rac-(ebthi)*Zr($Me_3SiCHCCSiMe_3$) iso2TS	138.30	145.68	33.05	34.82
rac-(ebthi)*Zr($Me_3SiCHCCSiMe_3$) iso2	35.41	41.11	8.46	9.83
rac-(ebthi)*Zr($Me_3SiCHCCSiMe_3$) iso3TS	139.31	147.93	33.30	35.36
rac-(ebthi)*Zr($Me_3SiCHCCSiMe_3$) iso3	36.61	39.50	8.75	9.44
rac-(ebthi)*Zr($Me_3SiCHCCSiMe_3$) iso4TS	114.95	117.69	27.47	28.13
rac-(ebthi)*Zr($Me_3SiCHCCSiMe_3$) iso4	83.10	80.45	19.86	19.23
rac-$Me_2Si(thi)_2Zr(Me_3SiCCCSiMe_3)$	-26.24	-47.79	-6.27	-11.42
rac-$Me_2Si(thi)_2Zr(Me_3SiCHCCSiMe_3)$ iso1_TS	146.53	149.97	35.02	35.84
rac-$Me_2Si(thi)_2Zr(Me_3SiCHCCSiMe_3)$ iso1	-16.46	-10.93	-3.94	-2.61
rac-(ebi)Zr($Me_3SiCCCSiMe_3$)	-18.30	-46.50	-4.37	-11.11
rac-(ebthi)*Ti($Me_3SiCHCCSiMe_3$) iso1_TS	124.63	126.24	29.79	30.17
rac-(ebthi)*Ti($Me_3SiCHCCSiMe_3$) iso1	5.53	11.00	1.32	2.63

These calculations confirm isomer 1 as the thermodynamic product of the reaction. Interestingly, the transition state (iso1TS) is now also the one with the lowest activation barrier and even allows a reaction at room temperature. This is in line with the experimental findings of formation of complex **3a** from **2a** within days (Scheme S3). Furthermore, we calculated the same pathway for the *rac*-$Me_2Si(thi)_2$ substituted complex **2b**, at the same level of theory, and found a higher activation barrier for the C-H activation step but a higher exergonic Gibbs free reaction enthalpy for the formation of complex **3b**. These findings agree with the elevated reaction temperature of 60 °C and shorter reaction time in this case. Finally, we calculated the same sequence for Ti analog **A** and found that its activation barrier in the same range as for the Zr examples. However, it shows an endergonic Gibbs free enthalpy of reaction for the formation of the hypothetical complex in which the CH_2 group was activated. This is in line with the experimental observation.

8.2.3 Reaction of 2a/2b with carbonyl compounds

We were next interested in the thermodynamic values of the reactions of complexes **2a/2b** with ketones to produce six-membered oxa-zirconacycles and enynes. Therefore, we computed the isodesmic reaction, which we already proposed for the 1-titanacyclobuta-2,3-diene **A**, to the Zr analogues. Please note that the formation of the corresponding *rac*-(ebthi)ZrO or *rac*-$Me_2Si(thi)_2ZrO$ species could not be verified spectroscopically during the reaction, as such species are known to decompose in the absence of stabilizing ligands. However, the formation of these oxido species should be the first step after extrusion of the organic coupling products from the six-membered metallacyclic complexes (Scheme S4).

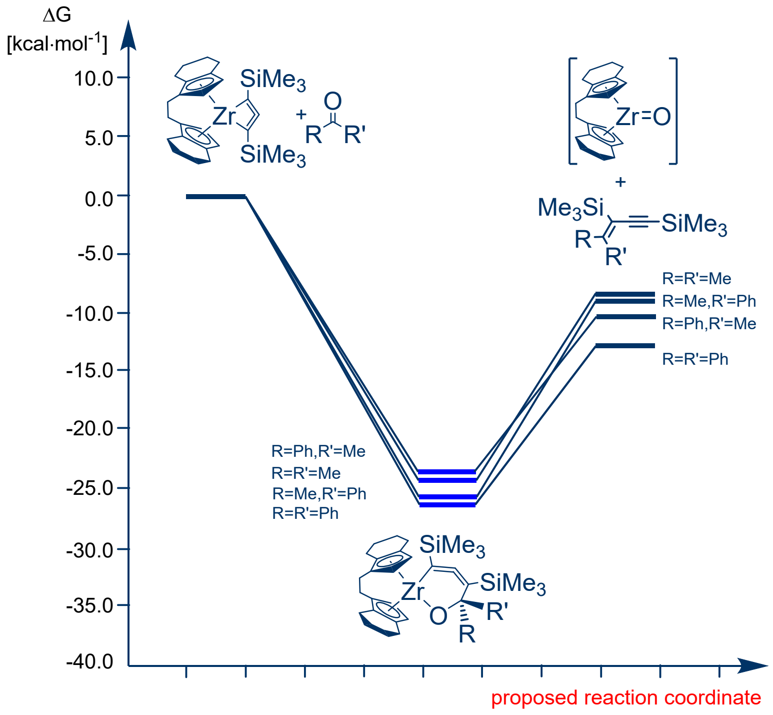

Scheme S4. Isodesmic reaction of **2a** and selected ketones.

Table S8. Calculated thermodynamic data for the reaction of **2a** and ketones (B3LYP/GD3BJ/def2tzvp).

Main Product	$\Delta_R H$ [kJ·mol^{-1}]	$\Delta_R G$ [kJ·mol^{-1}]	$\Delta_R H$ [kcal·mol^{-1}]	$\Delta_R G$ [kcal·mol^{-1}]
Acetone6Memb. complex	-175.90	-103.49	-42.04	-24.73
Acetophenone6Memb. complex iso1	-175.64	-107.82	-41.98	-25.77
Acetophenone6Memb. complex iso2	-168.68	-101.32	-40.32	-24.22
Benzophenone6Memb. complex	-181.66	-109.75	-43.42	-26.23
Acetone couplingprod.	154.61	65.96	36.95	15.76
EAcetophenone couplingprod. from iso1	157.97	70.22	37.76	16.78
EAcetophenone couplingprod. from iso2	151.02	63.72	36.09	15.23
ZAcetophenone couplingprod. from iso1	151.59	62.76	36.23	15.00
ZAcetophenone couplingprod. from iso2	144.64	56.26	34.57	13.45
Benzophenone couplingprod.	147.62	56.28	35.28	13.45
	Direct from **2a** to coupling products			
Acetone couplingprod.	-21.29	-37.53	-5.09	-8.97
EAcetophenone couplingprod.	-17.67	-37.60	-4.22	-8.99
ZAcetophenone couplingprod.	-24.05	-45.06	-5.75	-10.77
Benzophenone couplingprod.	-34.03	-53.47	-8.13	-12.78

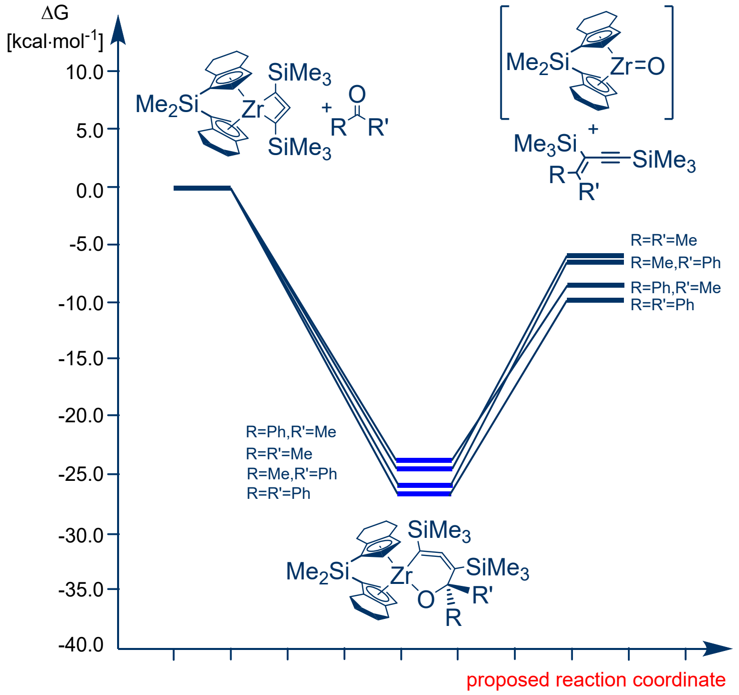

Scheme S5. Isodesmic reaction of **2b** and selected ketones.

Table S9. Calculated thermodynamic data for the reaction of **2b** and ketones (B3LYP/GD3BJ/def2tzvp).

Main Product	Δ_RH [kJ·mol^{-1}]	Δ_RG [kJ·mol^{-1}]	Δ_RH [kcal·mol^{-1}]	Δ_RG [kcal·mol^{-1}]
Acetone6Memb. complex	-180.51	-107.23	-43.14	-25.63
Acetophenone6Memb. complex iso1	-179.35	-111.16	-42.87	-26.57
Acetophenone6Memb. complex iso2	-173.91	-106.50	-41.56	-25.45
Benzophenone6Memb. complex	-185.78	-114.52	-44.40	-27.37
Acetone couplingprod.	164.63	78.45	39.35	18.75
EAcetophenone couplingprod. from iso1	167.10	82.31	39.94	19.67
EAcetophenone couplingprod. from iso2	161.66	77.66	38.64	18.56
ZAcetophenone couplingprod. from iso1	160.72	74.85	38.41	17.89
ZAcetophenone couplingprod. from iso2	155.28	70.20	37.11	16.78
Benzophenone couplingprod.	157.17	69.80	37.56	16.68
	Direct from **2b** to coupling products			
Acetone couplingprod.	-15.87	-28.78	-3.79	-6.88
EAcetophenone couplingprod.	-12.25	-28.85	-2.93	-6.89
ZAcetophenone couplingprod.	-18.63	-36.31	-4.45	-8.68
Benzophenone couplingprod.	-28.61	-44.72	-6.84	-10.69

8.2.4 Reaction of **3a/3b** with carbonyl compounds

We next computed the thermodynamic values of reactions of C-H activated complexes **3a/3b** with ketones.

Table S10. Calculated thermodynamic data for the reaction of **3a/3b** and ketones (B3LYP/GD3BJ/def2tzvp).

Main Product	Δ_RH [kJ·mol^{-1}]	Δ_RG [kJ·mol^{-1}]	Δ_RH [kcal·mol^{-1}]	Δ_RG [kcal·mol^{-1}]
3a+acetone Complex	-169.59	-115.00	-40.53	-27.49
3b+acetone Complex	-164.69	-110.01	-39.36	-26.29
3a+acetophenone Complex **14**	-169.92	-122.68	-40.61	-29.32
3b+acetophenone Complex **17**	-164.23	-113.60	-39.25	-27.15
3a+benzophneone Complex **13**	-171.72	-122.98	-41.04	-29.39
3b+benzophenone Complex **16**	-168.17	-117.00	-40.19	-27.96

8.2.5 Consideration of selectivity determining factors: Formation of 1-zirconacyclobuta-2,3-dienes vs. dinuclear complexes

To understand the differences in selectivity in reactions of *rac*-(ebthi)$ZrCl_2$ (giving metallacycle **2a**) and Cp_2ZrCl_2 (giving dinuclear complexes), we have calculated the Gibbs free energies for the aforementioned isodesmic exchange reaction for seven available zirconocenes.

We have considered a stepwise reaction where the first step is the formation of a singly methylated complex bearing a σ-bonded allene group (**Int1**, Scheme S6, Table S11). For all considered zirconocenes the formation of **Int1** is strongly exergonic with similar values ranging from -56.8 kJ·mol^{-1} to -65.3 kJ·mol^{-1}, with no significant trend or ligand influence.

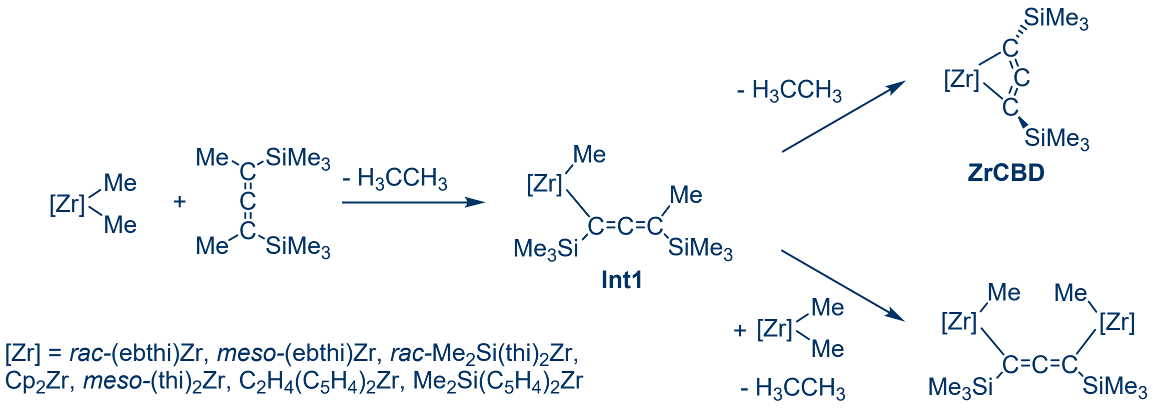

Scheme S6. Isodesmic reaction to **ZrCBD** or singly allene bridged dinuclear zirconocene complexes (B3LYP/GD3BJ/def2svpp).

In contrast, computation of the subsequent reaction of **Int1** shows that only the formation of the experimentally observed **ZrCBD** complexes **2a** and **2b** is nearly thermoneutral. For all other cyclopentadienyl ligands the Gibbs free energies are endergonic. Furthermore, these calculations show that the formation of the dinuclear complexes from **Int1** is also exergonic in all cases. Since a 2:1 stoichiometry is required in these cases, which is in contrast to the stoichiometric ratio provided experimentally, we further calculated the energies for the formation of **ZrCOT** complexes, which again require 1:1 stoichiometry. The results of this investigation are discussed in the manuscript. Furthermore, we expanded the Table 1, shown in the manuscript, by the calculated reaction enthalpies and report these here as Table S12.

Table S11. Calculated thermodynamic data for stepwise reaction to **ZrCBD** or singly allene bridged dinuclear zirconocene complexes (B3LYP/GD3BJ/def2svpp).

Main Product	$\Delta_R H$ [kJ·mol^{-1}]	$\Delta_R G$ [kJ·mol^{-1}]	$\Delta_R H$ [kcal·mol^{-1}]	$\Delta_R G$ [kcal·mol^{-1}]
rac-(ebthi)ZrMe_$Me_3SiCCCMeSiMe_3$	-82.20	-57.22	-19.65	-13.68
meso-(ebthi)ZrMe_$Me_3SiCCCMeSiMe_3$	-84.37	-65.30	-20.16	-15.61
rac-$Me_2Si(thi)_2ZrMe$_$Me_3SiCCCMeSiMe_3$	-82.77	-56.78	-19.78	-13.57
Cp_2ZrMe_$Me_3SiCCCMeSiMe_3$	-77.83	-58.34	-18.60	-13.94
meso-$(thi)_2ZrMe$_$Me_3SiCCCMeSiMe_3$	-76.25	-58.83	-18.22	-14.06
$C_2H_4(C_5H_4)_2ZrMe$_$Me_3SiCCCMeSiMe_3$	-86.04	-61.89	-20.56	-14.79
$Me_2Si(C_5H_4)_2ZrMe$_$Me_3SiCCCMeSiMe_3$	-84.67	-62.16	-20.24	-14.86
rac-(ebthi)Zr($Me_3SiCCCSiMe_3$)	55.59	3.74	13.29	0.89
meso-(ebthi)Zr($Me_3SiCCCSiMe_3$)	97.25	52.32	23.24	12.51
rac-$Me_2Si(thi)_2Zr$(Me3SiCCCSiMe3)	57.77	7.19	13.81	1.72
meso-$(thi)_2Zr(Me_3SiCCCSiMe_3)$	93.28	49.02	22.29	11.71
Cp_2Zr ($Me_3SiCCCSiMe_3$)	84.34	43.19	22.29	11.71
$C_2H_4(C_5H_4)_2Zr(Me_3SiCCCSiMe_3)$	85.91	37.41	20.53	8.94
$Me_2Si(C_5H_4)_2Zr(Me_3SiCCCSiMe_3)$	88.47	39.23	21.14	9.38
rac-(ebthi)ZrCCCZr(ebthi)-*rac*_inin	-57.39	-38.08	-13.72	-9.10
meso-(ebthi)ZrCCCZr(ebthi)-*meso* _inin	-93.53	-68.72	-22.35	-16.42
rac-$Me_2Si(thi)_2ZrCCCZrMe_2Si(thi)_2$-*rac* inin	-63.34	-45.40	-15.14	-10.85
meso-$(thi)_2ZrCCCZr(thi)_2$-*meso*_inin	-83.99	-55.57	-20.08	-13.28
$Cp_2ZrCCCCZrCp_2$ inin	-88.26	-62.83	-21.10	-15.02
$C_2H_4(C_5H_4)_2ZrCCCZr(C_5H_4)_2C_2H_4$ inin	-94.96	-74.23	-22.70	-17.74
$Me_2Si(C_5H_4)_2ZrCCCZr(C_5H_4)_2SiMe_2$inin	-89.34	-71.21	-21.35	-17.02

Table S12. Computed Gibbs free energies and reaction enthalpies (in kJ·mol^{-1}) for hypothetical 1-zirconacyclobuta-2,3-dienes (**ZrCBD**) and dinuclear complexes (**Zr_2COT**)[a]. B3LYP/GD3BJ/def2svpp.

Entry	[Zr]	2 x ΔH(ZrCBD) [kJ·mol^{-1}]	2 x ΔG(ZrCBD) [kJ·mol^{-1}]	ΔH(Zr_2COT) [kJ·mol^{-1}]	ΔG(Zr_2COT) [kJ·mol^{-1}]	ΔΔH [kJ·mol^{-1}]	ΔΔG [kJ·mol^{-1}]
1	*rac*-(ebthi)Zr	-53.22	-106.96	-137.42	-90.92	-84.2	16.04
2	*rac*-Me_2Si(thi)$_2$Zr	-50.01	-99.17	-158.48	-117.81	-108.47	-18.64
3	*meso*-(ebthi)Zr	25.77	-25.95	-148.08	-115.35	-173.85	-89.40
4	$C_2H_4(C_5H_4)_2$Zr	-0.27	-48.95	-295.43	-260.22	-295.16	-211.27
5	$Me_2Si(C_5H_4)_2$Zr	7.59	-45.86	-291.19	-253.85	-298.78	-208.00
6	Cp_2Zr	13.03	-30.30	-277.63	-239.16	-290.66	-208.86
7	*meso*-(thi)$_2$Zr	34.06	-19.62	-188.37	-149.15	-222.43	-129.52

[a] ΔΔG = ΔG(**Zr_2COT**)-(2x ΔG(**ZrCBD**)); ΔΔH = ΔH(**Zr_2COT**)-(2x ΔH(**ZrCBD**)).

8.2.6 Total enthalpies and energies for all calculated molecules

Table S13. Summary of thermodynamic data of all calculated compounds, * label for "activated" EBTHI ligands.

Compound	Nimag	HF	ZPE [kcal·mol^{-1}]	H_{tot} [a.u.]	G_{tot} [a.u.]	Method	Basis set
rac-(ebthi)Zr($Me_3SiCCCSiMe_3$)	0	-1756.500479	382.931250	-1755.850603	-1755.959783	BP86/Lanl2dz	TZVP
rac-(ebthi)* Zr($Me_3SiCHCCSiMe_3$) iso1	0	-1756.502635	383.055380	-1755.853185	-1755.958615	BP86/Lanl2dz	TZVP
Ethane	0	-79.7630454	46.566970	-79.684427	-79.710255	B3LYP/GD3BJ	def2svpp
R_$Me_3Si(Me)CCC(Me)SiMe_3$	0	-1012.202151	196.868340	-1011.865299	-1011.939121	B3LYP/GD3BJ	def2svpp
S_$Me_3Si(Me)CCC(Me)SiMe_3$	0	-1012.202151	196.866970	-1011.865299	-1011.939131	B3LYP/GD3BJ	def2svpp
rac-(ebthi)$ZrMe_2$	0	-902.9918843	289.407860	-902.507127	-902.579645	B3LYP/GD3BJ	def2svpp
meso-(ebthi)$ZrMe_2$	0	-902.9929113	289.494780	-902.508091	-902.579868	B3LYP/GD3BJ	def2svpp
rac-$Me_2Si(thi)_2ZrMe_2$	0	-1193.645287	300.186610	-1193.139522	-1193.219799	B3LYP/GD3BJ	def2svpp
Cp_2ZrMe_2	0	-513.695225	149.211930	-513.441838	-513.499035	B3LYP/GD3BJ	def2svpp
rac-$(thi)_2ZrMe_2$	0	-825.6080766	266.908480	-825.160419	-825.231174	B3LYP/GD3BJ	def2svpp
meso-$(thi)_2ZrMe_2$	0	-825.6086388	266.745950	-825.161157	-825.231923	B3LYP/GD3BJ	def2svpp
$C_2H_4(C_5H_4)_2ZrMe_2$	0	-591.0782536	171.860280	-590.787730	-590.846431	B3LYP/GD3BJ	def2svpp
$Me_2Si(C_5H_4)_2ZrMe_2$	0	-881.7319154	182.513590	-881.420521	-881.487521	B3LYP/GD3BJ	def2svpp
rac-(ebthi)ZrMe_ $Me_3SiCCCMeSiMe_3$	0	-1835.466305	442.449710	-1834.719307	-1834.830305	B3LYP/GD3BJ	def2svpp
meso-(ebthi)ZrMe_ $Me_3SiCCCMeSiMe_3$	0	-1835.467713	442.098450	-1834.721096	-1834.833604	B3LYP/GD3BJ	def2svpp
rac-$Me_2Si(thi)_2ZrMe$_ $Me_3SiCCCMeSiMe_3$	0	-2126.120023	453.413360	-2125.351919	-2125.470291	B3LYP/GD3BJ	def2svpp
Cp_2ZrMe_ $Me_3SiCCCMeSiMe_3$	0	-1446.167438	301.827120	-1445.652353	-1445.750120	B3LYP/GD3BJ	def2svpp
meso-$(thi)_2ZrMe$_ $Me_3SiCCCMeSiMe_3$	0	-1758.080029	419.038050	-1757.371070	-1757.483195	B3LYP/GD3BJ	def2svpp

$C_2H_4(C_5H_4)_2$ZrMe_ Me_3SiCCCMeSiMe_3	0	-1523.554062	325.027390	-1523.001373	-1523.098869	B3LYP/GD3BJ	def2svpp
$Me_2Si(C_5H_4)_2$ZrMe_ Me_3SiCCCMeSiMe_3	0	-1814.207424	335.883660	-1813.633642	-1813.740061	B3LYP/GD3BJ	def2svpp
rac-(ebthi)Zr(Me_3SiCCCSiMe_3)	0	-1755.681117	394.910900	-1755.013707	-1755.118625	B3LYP/GD3BJ	def2svpp
meso-(ebthi)Zr(Me_3SiCCCSiMe_3)	0	-1755.667153	395.060110	-1754.999628	-1755.103421	B3LYP/GD3BJ	def2svpp
rac-$(thi)_2$Zr(Me_3SiCCCSiMe_3)	0	-1678.297195	372.292940	-1677.667216	-1677.768000	B3LYP/GD3BJ	def2svpp
meso-$(thi)_2$Zr(Me_3SiCCCSiMe_3)	0	-1678.281111	372.124920	-1677.651116	-1677.754271	B3LYP/GD3BJ	def2svpp
Cp_2Zr(Me_3SiCCCSiMe_3)	0	-1366.371619	254.871540	-1365.935802	-1366.023416	B3LYP/GD3BJ	def2svpp
$C_2H_4(C_5H_4)_2$Zr(Me_3SiCCCSiMe_3)	0	-1443.757186	277.496250	-1443.284226	-1443.374364	B3LYP/GD3BJ	def2svpp
$Me_2Si(C_5H_4)_2$Zr(Me_3SiCCCSiMe_3)	0	-1734.409368	288.088830	-1733.915520	-1734.014865	B3LYP/GD3BJ	def2svpp
rac-(ebthi)*Zr(Me_3SiCHCCSiMe_3) iso1TS	1	-1755.623821	390.928480	-1754.963472	-1755.065796	B3LYP/GD3BJ	def2svpp
rac-(ebthi)*Zr(Me_3SiCHCCSiMe_3) iso1	0	-1755.68400	394.910260	-1755.017145	-1755.118716	B3LYP/GD3BJ	def2svpp
rac-(ebthi)*Zr(Me_3SiCHCCSiMe_3) iso2TS	1	-1755.6215413	391.928800	-1754.959935	-1755.060813	B3LYP/GD3BJ	def2svpp
rac-(ebthi)*Zr(Me_3SiCHCCSiMe_3) iso2	0	-1755.6678416	394.680670	-1755.001488	-1755.104139	B3LYP/GD3BJ	def2svpp
rac-(ebthi)*Zr(Me_3SiCHCCSiMe_3) iso3TS	1	-1755.6211941	392.064920	-1754.959459	-1755.060088	B3LYP/GD3BJ	def2svpp
rac-(ebthi)*Zr(Me_3SiCHCCSiMe_3) iso3	0	-1755.666968	394.783640	-1755.000495	-1755.103577	B3LYP/GD3BJ	def2svpp
rac-(ebthi)*Zr(Me_3SiCHCCSiMe_3) iso4TS	1	-1755.633178	390.818470	-1754.972792	-1755.075646	B3LYP/GD3BJ	def2svpp
rac-(ebthi)*Zr(Me_3SiCHCCSiMe_3) iso4	0	-1755.64750	393.102320	-1754.982650	-1755.088133	B3LYP/GD3BJ	def2svpp
rac-(ebthi)*Zr(Me_3SiCHCCSiMe_3) iso5	0	-1755.62980	393.169300	-1754.964982	-1755.069149	B3LYP/GD3BJ	def2svpp
rac-$Me_2Si(thi)_2$Zr(Me_3SiCCCSiMe_3)	0	-2046.333913	405.802780	-2045.645490	-2045.757296	B3LYP/GD3BJ	def2svpp
rac-$Me_2Si(thi)_2$*Zr(Me3SiCHCCSiMe3)	0	-2046.339976	405.625830	-2045.652071	-2045.762663	B3LYP/GD3BJ	def2svpp
rac-(ebthi)*Zr(Me_3SiCHCCSiMe_3) TSiso2to1 exo	1	-1755.570539	391.1965	-1754.90963	-1755.011537	B3LYP/GD3BJ	def2svpp
rac-(ebthi)*Zr(Me_3SiCHCCSiMe_3) TSiso3to2 exo	1	-1755.535617	389.62138	-1754.877088	-1754.97868	B3LYP/GD3BJ	def2svpp

rac-(ebthi)*Zr($Me_3SiCHCCSiMe_3$) TSiso4to3 exo	1	-1755.557502	389.71291	-1754.897994	-1755.003379	B3LYP/GD3BJ	def2svpp
rac-(ebthi)*Zr($Me_3SiCHCCSiMe_3$) TSiso2to1 endo	1	-1755.577553	390.39656	-1754.917508	-1755.020507	B3LYP/GD3BJ	def2svpp
rac-(ebthi)*Zr($Me_3SiCHCCSiMe_3$) TSiso3to2 endo	X	not found after 5 attempts				B3LYP/GD3BJ	def2svpp
rac-(ebthi)*Zr($Me_3SiCHCCSiMe_3$) TSiso4to3 endo	1	-1755.5543862	389.63333	-1754.895027	-1755.000339	B3LYP/GD3BJ	def2svpp
rac-(ebthi)ZrCCCZr(ebthi)-*rac*_inin	0	-2658.7216758	688.05561	-2657.563865	-2657.714197	B3LYP/GD3BJ	def2svpp
rac-(ebthi)ZrCCCZr(ebthi)-*rac*_inout	0	-2658.7165387	688.78039	-2657.558249	-2657.705669	B3LYP/GD3BJ	def2svpp
rac-(ebthi)ZrCCCZr(ebthi)-*rac* _outout	0	-2658.7048470	689.27807	-2657.546161	-2657.692920	B3LYP/GD3BJ	def2svpp
meso-(ebthi)ZrCCCZr(ebthi)-*meso*_inin	0	-2658.7372073	687.5184	-2657.580383	-2657.729391	B3LYP/GD3BJ	def2svpp
meso-(ebthi)ZrCCCZr(ebthi)-*meso* _inout	0	-2658.7340745	687.37299	-2657.577472	-2657.726827	B3LYP/GD3BJ	def2svpp
meso-(ebthi)ZrCCCZr(ebthi)-*meso* _outout	0	-2658.7296611	687.42807	-2657.572887	-2657.723579	B3LYP/GD3BJ	def2svpp
rac-$(thi)_2ZrCCCZr(thi)_2$-*rac*_inin	0	-2503.9527904	641.99549	-2502.870669	-2503.017136	B3LYP/GD3BJ	def2svpp
meso-$(thi)_2ZrCCCZr(thi)_2$-*meso* _inin	0	-2503.9570413	642.16552	-2502.87483	-2503.020856	B3LYP/GD3BJ	def2svpp
meso-$(thi)_2ZrCCCZr(thi)_2$-*meso* _inin	0	-2503.9619466	642.15406	-2502.879792	-2503.026028	B3LYP/GD3BJ	def2svpp
meso-$(thi)_2ZrCCCZr(thi)_2$-*meso* _inout	0	-2503.9598924	641.75104	-2502.878156	-2503.025634	B3LYP/GD3BJ	def2svpp
meso-$(thi)_2ZrCCCZr(thi)_2$-*meso* _outout iso1	0	-2503.9577526	641.88889	-2502.87589	-2503.023640	B3LYP/GD3BJ	def2svpp
meso-$(thi)_2ZrCCCZr(thi)_2$-*meso*_outout iso2	0	-2503.9577524	641.88899	-2502.87589	-2503.023626	B3LYP/GD3BJ	def2svpp
rac-$Me_2Si(thi)_2ZrCCCZr(thi)_2SiMe_2$ rac inin	0	-3240.0307055	709.45836	-3238.831139	-3238.997127	B3LYP/GD3BJ	def2svpp
$Cp_2ZrCCCCZrCp_2$ inin	0	-1880.1365541	406.57627	-1879.443382	-1879.562829	B3LYP/GD3BJ	def2svpp
$C_2H_4(C_5H_4)_2ZrCCCZr(C_5H_4)_2$ C_2H_4inin	0	-2034.9085265	452.14551	-2034.140844	-2034.263319	B3LYP/GD3BJ	def2svpp
$Me_2Si(C_5H_4)_2ZrCCCZr$ $(C_5H_4)_2SiMe_2$ inin	0	-2616.2133349	473.48278	-2615.403762	-2615.544449	B3LYP/GD3BJ	def2svpp
Cp_2ZrCOT	0	-2732.8588382	513.38268	-2731.982311	-2732.126383	B3LYP/GD3BJ	def2svpp
$C_2H_4(C_5H_4)_2ZrCOT$	0	-2887.6320904	559.00306	-2886.680874	-2886.829198	B3LYP/GD3BJ	def2svpp

$Me_2Si(C_5H_4)_2ZrCOT$	0	-3468.9380966	580.64667	-3467.944842	-3468.108952	B3LYP/GD3BJ	def2svpp
rac-(ebthi)ZrCOT	0	-3511.4028500	796.87616	-3510.059483	-3510.231141	B3LYP/GD3BJ	def2svpp
meso-(ebthi)ZrCOT	0	-3511.4072121	795.05334	-3510.065472	-3510.240893	B3LYP/GD3BJ	def2svpp
rac-$Me_2Si(thi)_2ZrCOT$	0	-4092.7150606	816.53992	-4091.332294	-4091.521692	B3LYP/GD3BJ	def2svpp
$(thi)_2ZrCOT$ iso 1	0	-3356.6502888	749.16922	-3355.383803	-3355.556954	B3LYP/GD3BJ	def2svpp
$(thi)_2ZrCOT$ iso 2	0	-3356.6537844	749.69963	-3355.38695	-3355.557875	B3LYP/GD3BJ	def2svpp
Ethane	0	-79.8694016	46.708110	-79.790541	-79.816384	B3LYP/GD3BJ	def2tzvp
Acetone	0	-193.2481561	52.24638	-193.15849	-193.194179	B3LYP/GD3BJ	def2tzvp
Acetophenone	0	-385.0743385	86.34292	-384.927953	-384.969597	B3LYP/GD3BJ	def2tzvp
Benzophenone	0	-576.8981672	119.97312	-576.695283	-576.744654	B3LYP/GD3BJ	def2tzvp
R_$Me_3SiCMeCCMeSiMe_3$	0	-1012.959244	197.445430	-1012.621394	-1012.696084	B3LYP/GD3BJ	def2tzvp
S_$Me_3SiCMeCCMeSiMe_3$	0	-1012.959244	197.445400	-1012.621394	-1012.696084	B3LYP/GD3BJ	def2tzvp
AcetoneCouplingprod. **9**	0	-1051.066112	201.09778	-1050.721684	-1050.799023	B3LYP/GD3BJ	def2tzvp
EAcetophenonecouplingprod. **7**	0	-1242.890539	234.58398	-1242.489766	-1242.574465	B3LYP/GD3BJ	def2tzvp
ZAcetophenonecouplingprod. **7**	0	-1242.89293	234.5819	-1242.492196	-1242.577306	B3LYP/GD3BJ	def2tzvp
Benzophenonecouplingprod. **5**	0	-1434.720405	268.04741	-1434.26333	-1434.355568	B3LYP/GD3BJ	def2tzvp
rac-$(ebthi)ZrMe_2$	0	-903.9987739	289.39662	-903.513891	-903.586976	B3LYP/GD3BJ	def2tzvp
rac-$Me_2Si(thi)_2ZrMe_2$	0	-1194.770949	300.29944	-1194.264846	-1194.345954	B3LYP/GD3BJ	def2tzvp
rac-$(ebi)_2ZrMe_2$		-899.1501255	230.88838	-898.760405	-898.829056	B3LYP/GD3BJ	def2tzvp
rac-(ebthi)ZrO sing.	0	-899.4229762	246.43767	-899.009718	-899.079015	B3LYP/GD3BJ	def2tzvp
rac-(ebthi)ZrO trip.	0	-899.3439768	245.66399	-898.931736	-899.001353	B3LYP/GD3BJ	def2tzvp
rac-$Me_2Si(thi)_2ZrO$ sing.	0	-1190.192674	257.57811	-1189.758004	-1189.833592	B3LYP/GD3BJ	def2tzvp

rac-(ebthi)Zr($Me_3SiCCCSiMe_3$) sing	0	-1757.232947	395.319800	-1756.564802	-1756.669563	B3LYP/GD3BJ	def2tzvp
rac-(ebthi)Zr($Me_3SiCCCSiMe_3$) sp trip	1	-1757.194949	394.55129	-1756.528695	-1756.632130	B3LYP/GD3BJ	def2tzvp
rac-(ebthi)*Zr($Me_3SiCHCCSiMe_3$) iso1	0	-1757.235022	395.17033	-1756.567589	-1756.669849	B3LYP/GD3BJ	def2tzvp
rac-(ebthi)*Zr($Me_3SiCHCCSiMe_3$) iso1_Int1	0	-1757.189005	393.19661	-1756.523918	-1756.630481	B3LYP/GD3BJ	def2tzvp
rac-(ebthi)*Zr($Me_3SiCHCCSiMe_3$) iso1_TS	1	-1757.172666	391.14279	-1756.511816	-1756.614778	B3LYP/GD3BJ	def2tzvp
rac-(ebthi)*Zr($Me_3SiCHCCSiMe_3$) iso2	0	-1757.218347	395.06525	-1756.551314	-1756.653905	B3LYP/GD3BJ	def2tzvp
rac-(ebthi)*Zr($Me_3SiCHCCSiMe_3$) iso2_TS	1	-1757.174272	392.13004	-1756.512127	-1756.614076	B3LYP/GD3BJ	def2tzvp
rac-(ebthi)*Zr($Me_3SiCHCCSiMe_3$) iso3	0	-1757.217832	394.98376	-1756.550857	-1756.654517	B3LYP/GD3BJ	def2tzvp
rac-(ebthi)*Zr($Me_3SiCHCCSiMe_3$) iso3_TS	1	-1757.173979	392.24865	-1756.511741	-1756.61322	B3LYP/GD3BJ	def2tzvp
rac-(ebthi)*Zr($Me_3SiCHCCSiMe_3$) iso4	0	-1757.19871	393.48507	-1756.533149	-1756.638921	B3LYP/GD3BJ	def2tzvp
rac-(ebthi)*Zr($Me_3SiCHCCSiMe_3$) iso4_TS	1	-1757.181901	391.02885	-1756.521021	-1756.624736	B3LYP/GD3BJ	def2tzvp
rac-$Me_2Si(thi)_2Zr(Me_3SiCCCSiMe_3)$ sing	0	-2048.004523	406.34419	-2047.315153	-2047.427473	B3LYP/GD3BJ	def2tzvp
rac-$Me_2Si(thi)_2$*Zr($Me_3SiCHCCSiMe_3$) iso1	0	-2048.010259	406.25846	-2047.321424	-2047.431635	B3LYP/GD3BJ	def2tzvp
rac-$Me_2Si(thi)_2$*Zr($Me_3SiCHCCSiMe_3$) iso1 TS	1	-2047.941399	402.1158	-2047.259342	-2047.370354	B3LYP/GD3BJ	def2tzvp
rac-(ebthi)Ti($Me_3SiCCCSiMe_3$)	0	-2559.611824	395.88384	-2558.943125	-2559.04643	B3LYP/GD3BJ	def2tzvp
rac-(ebthi)*Ti($Me_3SiCHCCSiMe_3$) iso1_TS	1	-2559.557099	391.57373	-2558.895655	-2558.998348	B3LYP/GD3BJ	def2tzvp
rac-(ebthi)*Ti($Me_3SiCHCCSiMe_3$) iso1	0	-2559.609332	395.89922	-2558.941019	-2559.042241	B3LYP/GD3BJ	def2tzvp
rac-$(ebi)_2Zr(Me_3SiCCCSiMe_3)$ sing		-1752.380268	336.44402	-1751.807689	-1751.910082	B3LYP/GD3BJ	def2tzvp
Acetone6Memb. *rac*-(ebthi) Complex **8**	0	-1950.551839	451.08043	-1949.79029	-1949.903159	B3LYP/GD3BJ	def2tzvp
Acetophenone6Memb. *rac*-(ebthi) Complex iso1 **6**	0	-2142.377706	484.54663	-2141.559652	-2141.680225	B3LYP/GD3BJ	def2tzvp
Acetophenone6Memb. *rac*-(ebthi) Complex iso2 **6**	0	-2142.375178	484.65178	-2141.557003	-2141.67775	B3LYP/GD3BJ	def2tzvp
Benzophenone6Memb. *rac*-(ebthi) Complex **4**	0	-2334.203854	518.11075	-2333.329274	-2333.45602	B3LYP/GD3BJ	def2tzvp

Acetone6Memb. *rac*-$Me_2Si(thi)_2$ Complex **12**	0	-2241.325108	462.04439	-2240.542394	-2240.662495	B3LYP/GD3BJ	def2tzvp
Acetophenone6Memb. *rac*-$Me_2Si(thi)_2$ Complex iso1 **11**	0	-2433.150549	495.40401	-2432.311416	-2432.439408	B3LYP/GD3BJ	def2tzvp
Acetophenone6Memb. *rac*-$Me_2Si(thi)_2$ Complex iso2 **11**	0	-2433.148659	495.56997	-2432.309343	-2432.437635	B3LYP/GD3BJ	def2tzvp
Benzophenone6Memb. *rac*-$Me_2Si(thi)_2$ Complex **10**	0	-2624.976797	528.92199	-2624.081195	-2624.215746	B3LYP/GD3BJ	def2tzvp
3a+acetone Complex	0	-1950.550334	449.06151	-1949.790674	-1949.907831	B3LYP/GD3BJ	def2tzvp
3b+acetone Complex	0	-2241.323663	460.18596	-2240.542642	-2240.667714	B3LYP/GD3BJ	def2tzvp
3a+acetophenone Complex **14**	0	-2142.376381	482.49654	-2141.560261	-2141.686172	B3LYP/GD3BJ	def2tzvp
3b+acetophenone Complex **17**	0	-2433.149465	493.69032	-2432.31193	-2432.444499	B3LYP/GD3BJ	def2tzvp
3a+benzophneone Complex **13**	0	-2334.200695	515.88407	-2333.328275	-2333.461344	B3LYP/GD3BJ	def2tzvp
3b+benzophenone Complex **16**	0	-2624.974496	526.9907	-2624.080758	-2624.220851	B3LYP/GD3BJ	def2tzvp

8.3 Bond analysis of selected complexes

8.3.1 Summary of NBO/NLMO Analysis of 2a, 2b and 3a

NBO6.0 analyses of the B3LYP/GD3BJ/def2tzvp optimized structures of **2a**/**2b** and **3a** were performed to analyze the natural localized molecular orbitals (NLMO). The NBO routine found two double bonds along the central CCC unit in the three investigated complexes (**2a**: Table S14; **3a**: Table S18; **2b**: Table S16). As expected, these bonds can be described as one σ-bond type with sp^2-sp hybrid orbitals, and one π-type bond formed from a pure p-type orbital at the central carbon atom (β-C) and one p-type orbital with minor s-character at the α-carbon atoms (or γ in **3a**). These findings are corroborated by the corresponding NLMO analysis of the central CCC unit in the investigated complexes (**2a**: Table S15; **3a**: Table S19; **2b**: Table S17). Furthermore, this analysis reveals for the CC π-type orbitals minor contributions (7.4 – 2.3%) of a d-orbital at the Zr center, which are absent for the corresponding CC σ-type orbitals.

While this NBO/NLMO routine works well for the bond description of the allene unit, it cannot be adapted for the Zr-C bond description. The NBO analysis of complex **2a** found two lone pairs (LPs) located at the α-carbon atoms with an occupancy of 1.3 electrons, the corresponding NLMO analysis of these LPs revealed that these show contributions (15%) of a d-type orbital from the Zr center and describe the metal-carbon bond. In contrast the NBO analysis of **2b** found a pair of a π-bonding and π*-antibonding orbital between the two α-carbon atoms, also with low occupancies (π: 1.1; π* 1.5 e^-) and again the NLMO analysis of these can be best interpreted as the Zr-carbon bond orbitals, with main contributions (π: 13; π* 18 %) of a d-type orbitals from Zr. Furthermore, the NBO analysis of **3a** found two Zr-C bonds with low occupancy, one with the carbon atom of the activated *rac*-(ebthi)* ligand (1.7 e^-) and one to the "unsaturated" carbon of the propargyl/allenyl unit (1.6 e^-).

8.3.2 NBO/NLMO analysis of 2a

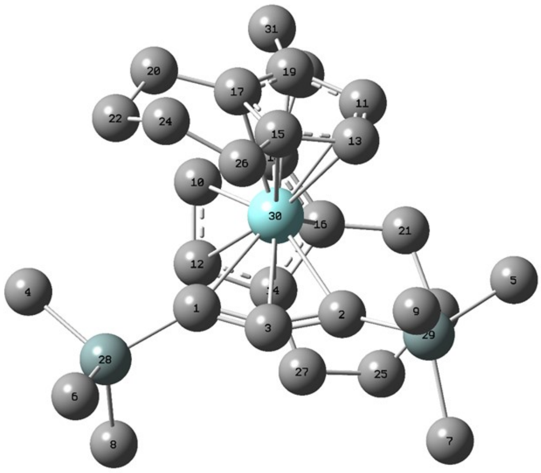

Figure S109. Labeling scheme of the NBO/NLMO analysis of complex **2a**.

Table S14. Summary of selected NBO analysis of **2a**.

44. (1.33758) LP (1) C 1<br><br>s(11.91%)p 7.39(87.93%)d 0.01(0.11%) f 0.00(0.05%)	45. (1.33758) LP (1) C 2<br><br>s(11.91%)p 7.39(87.93%)d 0.01(0.11%) f 0.00(0.05%)
48. (1.95562) BD (1) C 1- C 3<br><br>(47.68%) 0.6905* C 1 s(31.03%)p 2.21(68.67%)d 0.01(0.25%) f 0.00(0.05%)<br><br>(52.32%) 0.7233* C 3 s(49.71%)p 1.01(50.07%)d 0.00(0.11%)f 0.00(0.10%)	49. (1.78617) BD (2) C 1- C 3<br><br>(56.99%) 0.7549* C 1 s(9.19%)p 9.87(90.66%)d 0.01(0.12%) f 0.00(0.04%)<br><br>(43.01%) 0.6558* C 3 s(0.21%)p99.99(99.33%)d 1.82(0.37%) f 0.43(0.09%)
51. (1.95562) BD (1) C 2- C 3<br><br>(47.68%) 0.6905* C 2 s(31.03%)p 2.21(68.67%)d 0.01(0.25%) f 0.00(0.05%)<br><br>(52.32%) 0.7233* C 3 s(49.71%)p 1.01(50.07%)d 0.00(0.11%) f 0.00(0.10%)	52. (1.78617) BD (2) C 2- C 3<br><br>(56.99%) 0.7549* C 2 s(9.19%)p 9.87(90.66%)d 0.01(0.12%) f 0.00(0.04%)<br><br>(43.01%) 0.6558* C 3 s(0.21%)p99.99(99.33%)d 1.82(0.37%) f 0.43(0.09%)

Table S15. Summary of selected NLMOs of **2a**.

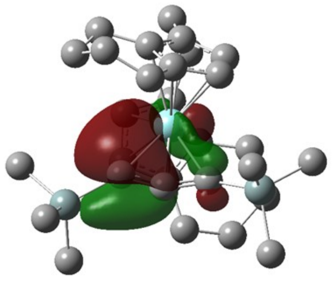	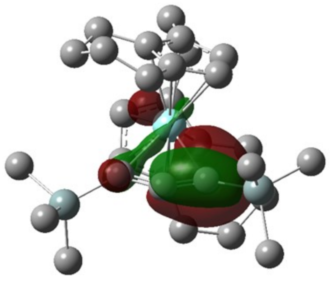
Shortened NLMO Analysis of C(1) "LP" (threshold > 1.3%)<br><br>→ C1-C3 π-bond with binding contribution of one d orbital at the Zr center<br><br>44. (2.00000) 63.2149% LP (1) C 1<br><br>65.957% C 1 s(13.93%)p 6.17(85.92%)d 0.01(0.12%) f 0.00(0.03%)<br><br>5.637% C 2 s(0.27%)p99.99(99.52%)d 0.29(0.08%) f 0.51(0.14%)<br><br>9.274% C 3 s(0.16%)p99.99(99.19%)d 3.58(0.56%) f 0.65(0.10%)<br><br>15.421% Zr 30 s(9.55%)p 0.01(0.07%)d 9.46(90.29%) f 0.01(0.09%)	Shortened NLMO Analysis of C(2) "LP" (threshold > 1.3%)<br><br>→ C2-C3 π-bond with binding contribution of one d orbital at the Zr center<br><br>45. (2.00000) 63.2149% LP (1) C 2<br><br>5.637% C 1 s(0.27%)p99.99(99.52%)d 0.29(0.08%) f 0.51(0.14%)<br><br>65.957% C 2 s(13.93%)p 6.17(85.92%)d 0.01(0.12%) f 0.00(0.03%)<br><br>9.274% C 3 s(0.16%)p99.99(99.19%)d 3.58(0.56%) f 0.65(0.10%)<br><br>15.421% Zr 30 s(9.55%)p 0.01(0.07%)d 9.46(90.29%) f 0.01(0.09%)
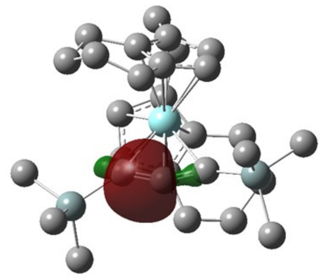	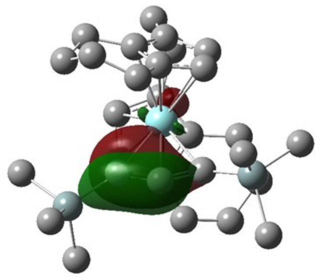
Shortened NLMO Analysis of of C(1)-C(3) σ-bond (threshold > 1.3%)<br><br>48. (2.00000) 97.7449% BD (1) C 1- C 3	Shortened NLMO Analysis of of C(1)-C(3) π-bond (threshold > 1.3%)<br><br>49. (2.00000) 88.7790% BD (2) C 1- C 3

46.748% C 1 s(29.17%)p 2.42(70.53%)d 0.01(0.26%) f 0.00(0.05%)<br><br>51.462% C 3 s(44.39%)p 1.25(55.39%)d 0.00(0.12%) f 0.00(0.11%)	50.756% C 1 s(7.27%)p12.73(92.58%)d 0.01(0.11%) f 0.01(0.04%)<br><br>1.691% C 2 s(1.87%)p52.15(97.58%)d 0.26(0.48%) f 0.04(0.07%)<br><br>39.161% C 3 s(0.15%)p99.99(99.41%)d 2.35(0.36%) f 0.55(0.08%)<br><br>6.516% Zr 30 s(1.18%)p 0.26(0.31%)d83.52(98.30%) f 0.19(0.22%)
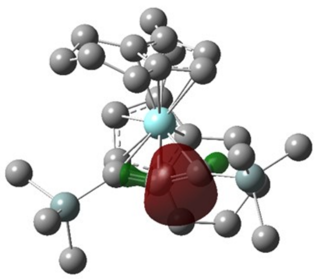	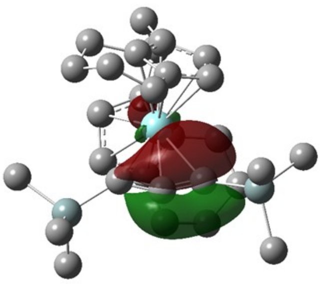
Shortened NLMO Analysis of of C(2)-C(3) σ-bond (threshold > 1.3%)<br><br>51. (2.00000) 97.7449% BD (1) C 2- C 3<br><br>46.748% C 2 s(29.17%)p 2.42(70.53%)d 0.01(0.26%) f 0.00(0.05%)<br><br>51.462% C 3 s(44.39%)p 1.25(55.39%)d 0.00(0.12%) f 0.00(0.11%)	Shortened NLMO Analysis of of C(2)-C(3) π-bond (threshold > 1.3%)<br><br>52. (2.00000) 88.7790% BD (2) C 2- C 3<br><br>1.691% C 1 s(1.87%)p52.15(97.58%)d 0.26(0.48%) f 0.04(0.07%)<br><br>50.756% C 2 s(7.27%)p12.73(92.58%)d 0.01(0.11%) f 0.01(0.04%)<br><br>39.161% C 3 s(0.15%)p99.99(99.41%)d 2.35(0.36%) f 0.55(0.08%)<br><br>6.516% Zr 30 s(1.18%)p 0.26(0.31%)d83.52(98.30%) f 0.19(0.22%)

8.3.3 NBO/NLMO analysis of 2b

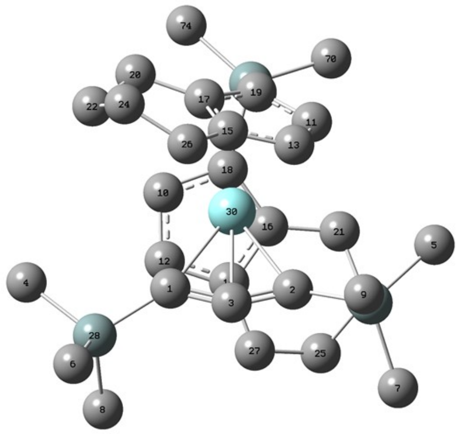

Figure S110. Labelling scheme of the NBO/NLMO analysis of complex **2b**.

Table S16. Summary of selected NBO analysis of **2b**.

51. (1.10988) BD (1) C 1- C 2<br><br>(53.15%) 0.7291* C 1 s(11.40%)p 7.76(88.44%)d 0.01(0.10%) f 0.00(0.05%)<br>(46.85%) 0.6845* C 2 s(11.41%)p 7.75(88.45%)d 0.01(0.10%) f 0.00(0.05%)	136. (1.53335) BD*(1) C 1- C 2<br><br>(46.85%) 0.6845* C 1 s(11.40%)p 7.76(88.44%)d 0.01(0.10%) f 0.00(0.05%)<br>(53.15%) -0.7291* C 2 s(11.41%)p 7.75(88.45%)d 0.01(0.10%) f 0.00(0.05%)
52. (1.95656) BD (1) C 1- C 3<br><br>(47.62%) 0.6901* C 1 s(30.42%)p 2.28(69.28%)d 0.01(0.26%)f 0.00(0.04%)<br>(52.38%) 0.7237* C 3 s(49.63%)p 1.01(50.18%)d 0.00(0.10%) f 0.00(0.09%)	53. (1.76471) BD (2) C 1- C 3<br><br>(58.15%) 0.7625* C 1 s(11.38%)p 7.77(88.45%)d 0.01(0.13%) f 0.00(0.03%)<br>(41.85%) 0.6469* C 3 s(0.21%)p99.99(99.33%)d 1.75(0.37%) f 0.41(0.09%)
55. (1.95748) BD (1) C 2- C 3<br><br>(47.68%) 0.6905* C 2 s(31.54%)p 2.16(68.18%)d 0.01(0.25%) f 0.00(0.04%)<br>(52.32%) 0.7233* C 3 s(49.78%)p 1.01(50.03%)d 0.00(0.10%) f 0.00(0.09%)	56. (1.79616) BD (2) C 2- C 3<br><br>(56.51%) 0.7517* C 2 s(7.95%)p11.56(91.90%)d 0.01(0.11%) f 0.01(0.04%)<br>(43.49%) 0.6595* C 3 s(0.22%)p99.99(99.30%)d 1.83(0.40%) f 0.40(0.09%)

Table S17. Summary of selected NLMOs of **2b**.

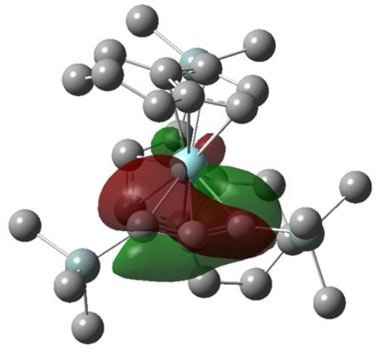	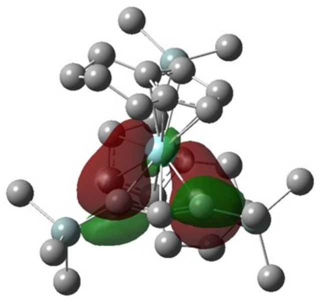
Shortened NLMO Analysis of C(1)-C(2) π-bond (threshold > 1.3%)<br><br>51. (2.00000) 51.4074% BD (1) C 1- C 2<br><br>34.121% C 1 s(15.20%)p 5.57(84.64%)d 0.01(0.14%)<br>f 0.00(0.02%)<br><br>31.087% C 2 s(13.41%)p 6.44(86.44%)d 0.01(0.12%) f 0.00(0.02%)<br><br>19.809% C 3 s(0.00%)p 1.00(99.58%)d 0.00(0.32%)<br>f 0.00(0.10%)<br><br>13.043% Zr 30 s(0.09%)p 0.39(0.04%)d99.99(99.78%)<br>f 1.02(0.09%)	Shortened NLMO Analysis of C(1)-C(2) π*-bond (threshold > 1.3%)<br><br>136. (2.00000) 75.0306% BD*(1) C 1- C 2<br><br>35.971% C 1 s(11.62%)p 7.59(88.22%)d 0.01(0.10%)<br>f 0.00(0.05%)<br><br>39.497% C 2 s(10.42%)p 8.58(89.44%)d 0.01(0.09%) f 0.00(0.05%)<br><br>18.306% Zr 30 s(16.99%)p 0.00(0.06%)d 4.88(82.86%)<br>f 0.01(0.09%)
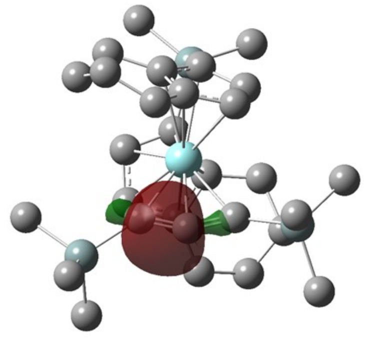	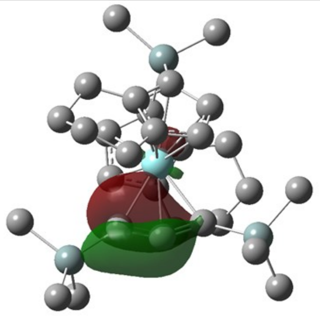
Shortened NLMO Analysis of of C(1)-C(3) σ -bond (threshold > 1.3%)<br><br>52. (2.00000) 97.8051% BD (1) C 1- C 3	Shortened NLMO Analysis of of C(1)-C(3) π -bond (threshold > 1.3%)<br><br>53. (2.00000) 87.3705% BD (2) C 1- C 3

46.746% C 1 s(28.39%)p 2.51(71.31%)d 0.01(0.26%)<br>f 0.00(0.04%)<br><br>51.565% C 3 s(44.30%)p 1.25(55.50%)d 0.00(0.11%)<br>f 0.00(0.09%)	52.191% C 1 s(8.58%)p10.63(91.26%)d 0.01(0.12%)<br>f 0.00(0.04%)<br><br>1.898% C 2 s(0.32%)p99.99(99.20%)d 1.28(0.41%)<br>f 0.21(0.07%)<br><br>36.703% C 3 s(0.10%)p99.99(99.46%)d 3.52(0.35%)<br>f 0.80(0.08%)<br><br>7.362% Zr 30 s(1.49%)p 0.20(0.29%)d65.80(98.03%)<br>f 0.13(0.19%)
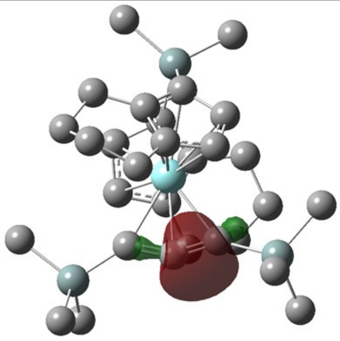	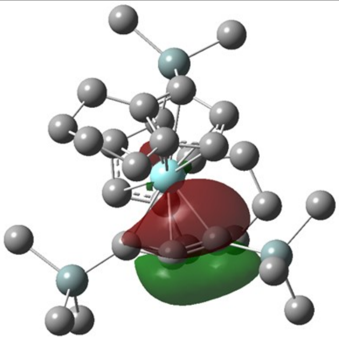
Shortened NLMO Analysis of of C(2)-C(3) σ -bond (threshold > 1.3%)<br><br>55. (2.00000) 97.8535% BD (1) C 2- C 3<br><br>46.797% C 2 s(29.25%)p 2.41(70.47%)d 0.01(0.25%)<br>f 0.00(0.04%)<br><br>51.503% C 3 s(44.49%)p 1.24(55.32%)d 0.00(0.10%) f 0.00(0.09%)	Shortened NLMO Analysis of of C(2)-C(3) π -bond (threshold > 1.3%)<br><br>56. (2.00000) 89.3922% BD (2) C 2- C 3<br><br>1.517% C 1 s(2.62%)p36.89(96.73%)d 0.22(0.58%)<br>f 0.03(0.07%)<br><br>50.646% C 2 s(7.05%)p13.17(92.81%)d 0.01(0.10%)<br>f 0.01(0.04%)<br><br>39.684% C 3 s(0.14%)p99.99(99.40%)d 2.65(0.38%)<br>f 0.57(0.08%)<br><br>6.253% Zr 30 s(1.06%)p 0.37(0.40%)d92.44(98.31%)<br>f 0.21(0.23%)

8.3.4 NBO/NLMO analysis of 3a

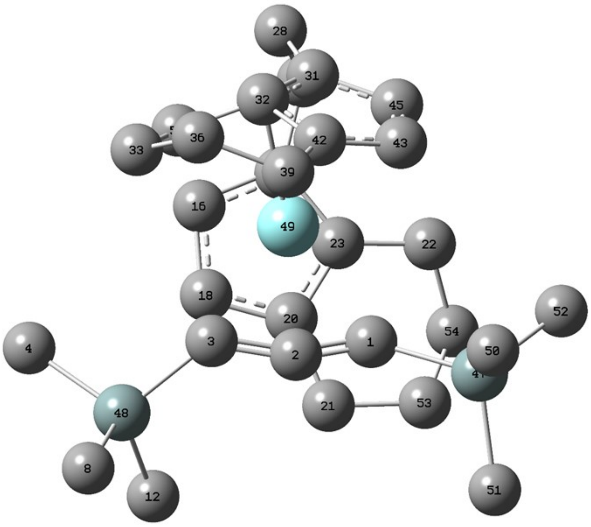

Figure S111. Labelling scheme of the NBO/NLMO analysis of complex **3a**.

Table S18. Summary of selected NBO analysis of **3a**.

46. (1.96057) BD (1) C 1- C 2<br><br>(47.95%) 0.6924* C 1 s(39.76%)p 1.51(59.98%)d 0.01(0.23%) f 0.00(0.03%)<br><br>(52.05%) 0.7215* C 2 s(52.13%)p 0.92(47.73%)d 0.00(0.05%) f 0.00(0.08%)	47. (1.90291) BD (2) C 1- C 2<br><br>(51.95%) 0.7208* C 1 s(0.03%)p99.99(99.86%)d 1.65(0.06%) f 1.60(0.05%)<br><br>(48.05%) 0.6932* C 2 s(0.11%)p99.99(99.58%)d 2.08(0.22%)f 0.87(0.09%)
49. (1.59096) BD (1) C 1-Zr 49<br><br>(82.49%) 0.9082* C 1 s(30.05%)p 2.32(69.76%)d 0.01(0.17%)f 0.00(0.01%)<br><br>(17.51%) 0.4185*Zr 49 s(3.64%)p 0.04(0.14%)d26.39(96.14%) f 0.02(0.08%)	50. (1.96701) BD (1) C 2- C 3<br><br>(51.00%) 0.7141* C 2 s(46.22%)p 1.16(53.61%)d 0.00(0.07%)f 0.00(0.09%)<br><br>(49.00%) 0.7000* C 3 s(29.70%)p 2.36(70.12%)d 0.01(0.16%)f 0.00(0.03%)
51. (1.82597) BD (2) C 2- C 3<br><br>(38.40%) 0.6197* C 2 s(1.49%)p65.71(98.14%)d 0.19(0.28%)f 0.05(0.08%)<br><br>(61.60%) 0.7849* C 3 s(8.31%)p11.03(91.59%)d 0.01(0.08%)f 0.00(0.02%)	113. (1.71387) BD (1)Zr 49- C 56<br><br>(27.22%) 0.5217*Zr 49 s(10.09%)p 0.01(0.06%)d 8.89(89.79%) f 0.01(0.06%)<br><br>(72.78%) 0.8531* C 56 s(14.08%)p 6.09(85.80%)d 0.01(0.11%) f 0.00(0.01%)

Table S19. Summary of selected NLMOs of **3a**.

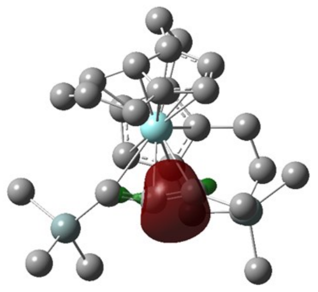	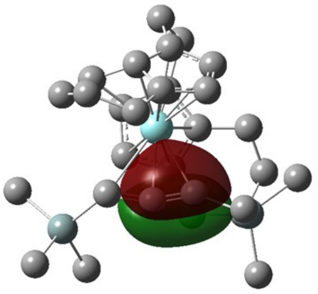
Shortened NLMO Analysis of C(1)-C(2) σ-bond (threshold > 1.3%)<br><br>46. (2.00000) 97.9970% BD (1) C 1- C 2<br><br>47.127% C 1 s(34.99%)p 1.85(64.75%)d 0.01(0.23%)<br>f 0.00(0.03%)<br><br>51.225% C 2 s(45.97%)p 1.17(53.88%)d 0.00(0.06%)<br>f 0.00(0.08%)	Shortened NLMO Analysis of of C(1)-C(2) π -bond (threshold > 1.3%)<br><br>47. (2.00000) 95.0346% BD (2) C 1- C 2<br><br>49.353% C 1 s(0.01%)p 1.00(99.88%)d 0.00(0.06%)f 0.00(0.05%)<br><br>45.692% C 2 s(0.08%)p99.99(99.60%)d 2.67(0.22%) f 1.11(0.09%)<br><br>2.306% Zr 49 s(0.02%)p21.61(0.44%)d99.99(98.83%)<br>f35.06(0.71%)
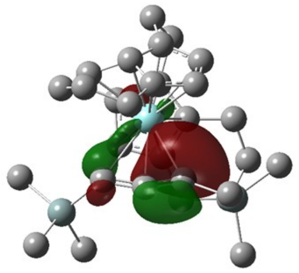	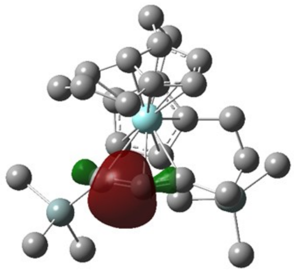
Shortened NLMO Analysis of of C(1)-Zr(49)-bond (threshold > 1.3%)<br><br>49. (2.00000) 78.1417% BD (1) C 1-Zr 49<br><br>64.871% C 1 s(24.52%)p 3.07(75.31%)d 0.01(0.16%) f 0.00(0.02%)<br><br>11.496% C 2 s(0.20%)p99.99(99.29%)d 1.97(0.40%) f 0.51(0.11%)	Shortened NLMO Analysis of of C(2)-C(3) σ -bond (threshold > 1.3%)<br><br>50. (2.00000) 98.3308% BD (1) C 2- C 3<br><br>50.268% C 2 s(41.57%)p 1.40(58.26%)d 0.00(0.07%) f 0.00(0.10%)<br><br>48.258% C 3 s(26.12%)p 2.82(73.69%)d 0.01(0.16%) f 0.00(0.03%)

6.858% C 3 s(1.89%)p51.76(97.99%)d 0.03(0.05%) f 0.03(0.07%)<br><br>13.662% Zr 49 s(3.18%)p 0.05(0.15%)d30.39(96.59%) f 0.03(0.09%)	
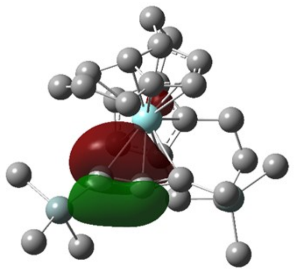	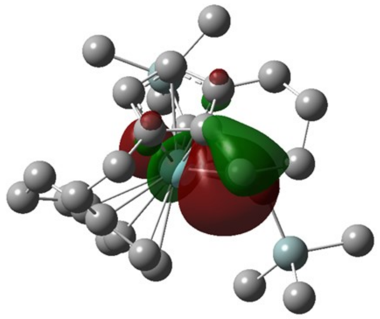
Shortened NLMO Analysis of of C(2)-C(3) π-bond (threshold > 1.3%)<br><br>51. (2.00000) 90.6303% BD (2) C 2- C 3<br><br>34.411% C 2 s(0.95%)p99.99(98.69%)d 0.30(0.29%) f 0.09(0.08%)<br><br>56.307% C 3 s(7.98%)p11.52(91.92%)d 0.01(0.08%)<br><br>6.678% Zr 49 s(8.95%)p 0.03(0.30%)d10.13(90.66%) f 0.01(0.10%)	Shortened NLMO Analysis of of Zr(49)-C(56) bond (threshold > 1.3%)<br><br>113. (2.00000) 85.2496% BD (1)Zr 49- C 56<br><br>2.342% C 31 s(1.63%)p60.01(97.53%)d 0.48(0.78%) f 0.04(0.07%)<br><br>3.449% C 32 s(1.62%)p59.99(97.15%)d 0.70(1.13%) f 0.06(0.10%)<br><br>1.962% C 42 s(2.84%)p33.90(96.18%)d 0.31(0.89%) f 0.03(0.10%)<br><br>24.586% Zr 49 s(11.81%)p 0.00(0.04%)d 7.46(88.10%) f 0.00(0.05%)<br><br>61.172% C 56 s(13.12%)p 6.61(86.75%)d 0.01(0.11%) f 0.00(0.01%)

8.3.5 Comparison of natural charges from NBO analysis in the formal allenediide unit in complexes 2a, 2b, and A.

To evaluate the differences in the bond situation of group 4 1-metalla-cyclobuta-2,3-dienes (M = Ti (**A**),[7] Zr (**2a,2b**)) we calculated the sum of the natural charges in the formal allenediide units in these complexes (

Table **S20**). The higher negative charge in the zirconocene complexes suggest a higher polarity of the M-C interaction in the metallacycles which is well in line with the greater biradicaloid character of the Ti complex **A** leading to a formal Ti(III) center which is antiferromagnetically coupled to the delocalized electron in the allene unit. Based on this calculation, the formal oxidation state of Zr is greater than Zr(III), so the interaction in **2a** and **2b** is best described as a Zr(IV) center coordinated with an allenediide ligand.

Table S20. Calculation of the sum of natural charges in the formal allenediide/allenide units from NBO analysis in complexes **2a**, **2b**, **A**, **TS 2a to 3a** and **3a** (B3LYP/GD3BJ/def2tzvp).

2a		2b		A		TS 2a to 3a		3a	
Atom	Nat Charge	Atom	Nat Charge	Atom	Nat Charge	Atom	Nat Charge	Atom	Nat Charge
C 1	-0.89502	C 1	-0.885	C 1	-0.76858	C 1	-0.85261	C 1	-0.74595
C 2	-0.89502	C 2	-0.88348	C 2	-0.76858	C 2	-0.99355	C 2	-0.01571
C 3	-0.06522	C 3	-0.06565	C 3	-0.08284	C 3	-0.00983	C 3	-0.92578
C 4	-1.09072	C 4	-1.09131	C 4	-1.09218	C 4	-1.08308	H 55	0.25337
C 5	-1.09072	C 5	-1.09217	C 5	-1.09218	C 5	-1.09683	C 4	-1.09307
C 6	-1.0835	C 6	-1.08324	C 6	-1.08335	C 6	-1.08336	H 5	0.23036
C 7	-1.0835	C 7	-1.08392	C 7	-1.08335	C 7	-1.09114	H 6	0.22775
C 8	-1.09285	C 8	-1.09248	C 8	-1.09268	C 8	-1.08825	H 7	0.2278
C 9	-1.09285	C 9	-1.09142	C 9	-1.09268	C 9	-1.10216	C 8	-1.09135
Si 28	1.67753	Si 28	1.67659	Si 28	1.67321	Si 28	1.6641	H 9	0.22796
Si 29	1.67753	Si 29	1.67925	Si 29	1.67321	Si 29	1.70837	H 10	0.22522
H 33	0.22759	H 31	0.2291	H 33	0.2288	H 33	0.22892	H 11	0.22578
H 34	0.22759	H 32	0.22787	H 34	0.2288	H 34	0.23286	C 12	-1.10066
H 35	0.22711	H 33	0.22619	H 35	0.22771	H 35	0.23264	H 13	0.22711
H 36	0.22711	H 34	0.22689	H 36	0.22771	H 36	0.21925	H 14	0.23133
H 37	0.22027	H 35	0.22253	H 37	0.22178	H 37	0.21714	H 15	0.23246
H 38	0.22027	H 36	0.22102	H 38	0.22178	H 38	0.22934	Si 47	1.67465
H 39	0.22559	H 37	0.22585	H 39	0.22603	H 39	0.23005	Si 48	1.68894
H 40	0.22559	H 38	0.22588	H 40	0.22603	H 40	0.22593	C 50	-1.08616
H 41	0.22035	H 39	0.22112	H 41	0.22189	H 41	0.22415	C 51	-1.0901
H 42	0.22035	H 40	0.22002	H 42	0.22189	H 42	0.22937	C 52	-1.0951
H 43	0.23032	H 41	0.23085	H 43	0.23047	H 43	0.22954	H 66	0.2303
H 44	0.23032	H 42	0.23012	H 44	0.23047	H 44	0.23193	H 67	0.22733
H 45	0.22277	H 43	0.22314	H 45	0.22347	H 45	0.22347	H 68	0.23073
H 46	0.22277	H 44	0.22292	H 46	0.22347	H 46	0.22438	H 69	0.22839
H 47	0.22849	H 45	0.229	H 47	0.22923	H 47	0.22779	H 70	0.23
H 48	0.22849	H 46	0.22854	H 48	0.22923	H 48	0.23123	H 71	0.22755
H 49	0.22684	H 47	0.22616	H 49	0.22631	H 49	0.22989	H 72	0.22618
H 50	0.22684	H 48	0.22715	H 50	0.22631	H 50	0.22384	H 73	0.23108
Sum	-0.97568	Sum	-0.94848	Sum	-0.73862	Sum	-0.93662	H 74	0.22695
						Shifted H 66	0.30138	Sum	-0.51264

8.3.6 QT-AIM analysis of complexes 2a, 2b, A, 3a and 3b

QT-AIM analysis[17] for group 4 1-metallacyclobuta-2,3-diene complexes **2a**, **2b** and **A** revealed two M–C bond paths in the metallacycles in between the metal center and the α-carbon atoms (Figure S112 - Figure S114). Furthermore, this analysis shows in all cases a ring critical point

between the metal center and the β-carbon atom. These findings point to the absence of a bond between the central carbon atom and the metal center, which was considered when drawing the Lewis representations of these complexes. In addition to the contour plot of the Laplacian of the electron density $\nabla^2 r$ of the investigated complexes we added the Wiberg bond indices (WBI) next to the bond critical points in italic numbers. The lower WBI between the central carbon atom and the metal center compared to the α-C-M bonds supports the result of the QT-AIM analysis. Furthermore, the WBI of 1.95 for the C-C bonds in the allene units in **2a** and **2b** clearly indicate double bonds based on this theory (*c.f.* 1.93 in **A**). The same analysis was performed for the η^3-propargyl/allenyl complexes **3a** and **3b** (Figure S115 - Figure S116). The QT-AIM analysis for these compounds found only one bond path between the $Me_3SiCCCHSiMe_3$ unit and the Zr atom. The WBI of these bonds are lower than 0.6 but these values are larger than WBI for the other M-C interactions, which supports the QT-AIM analysis. In line with the description as η^3-propargyl/allenyl complexes we found two different WBI along this unit (1.7 and 2.2 in both complexes).

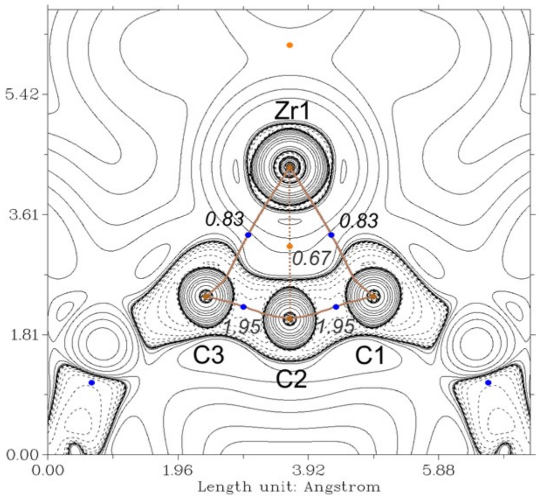

Figure S112. Contour plot of the Laplacian of the electron density $\nabla^2 r$ of complex **2a** in the C-Zr-C plane. Dashed lines indicate negative (local charge concentration), solid lines indicate positive values (local charge depletion). The Laplacian plot is overlaid with the molecular graph from QT-AIM analysis and Wiberg bond indices (italic small numbers). Brown lines indicate bond paths, brown dashed lines are hypothetical bonds, blue dots correspond to bond critical points, light brown dots indicate ring critical points. Density from B3LYP/GD3BJ/def2tzvp calculation.

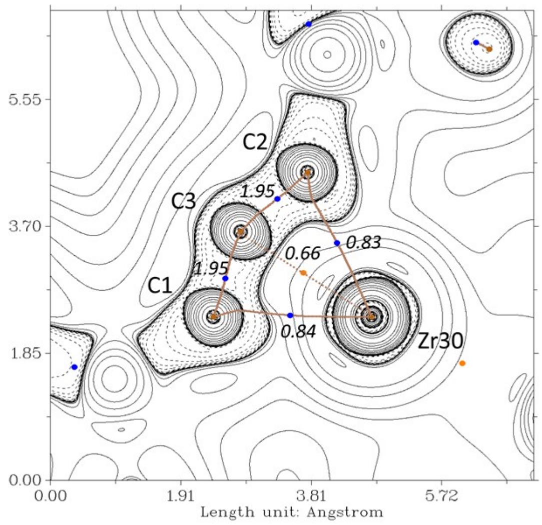

Figure S113. Contour plot of the Laplacian of the electron density $\nabla^2 r$ of complex **2b** in the C-Zr-C plane. Dashed lines indicate negative (local charge concentration), solid lines indicate positive values (local charge depletion). The Laplacian plot is overlaid with the molecular graph from QT-AIM analysis and Wiberg bond indices (italic small numbers). Brown lines indicate bond paths, brown dashed lines are hypothetical bonds, blue dots correspond to bond critical points, light brown dots indicate ring critical points. Density from B3LYP/GD3BJ/def2tzvp calculation.

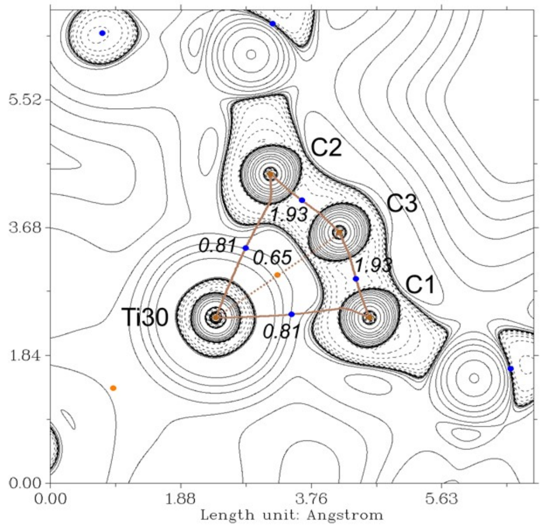

Figure S114. Contour plot of the Laplacian of the electron density $\nabla^2 r$ of complex **A** in the C-Ti-C plane. Dashed lines indicate negative (local charge concentration), solid lines indicate positive values (local charge depletion). The Laplacian plot is overlaid with the molecular graph from QT-AIM analysis and Wiberg bond indices (italic small numbers). Brown lines indicate bond paths, brown dashed lines are hypothetical bonds, blue dots correspond to bond critical points, light brown dots indicate ring critical points. Density from B3LYP/GD3BJ/def2tzvp calculation.

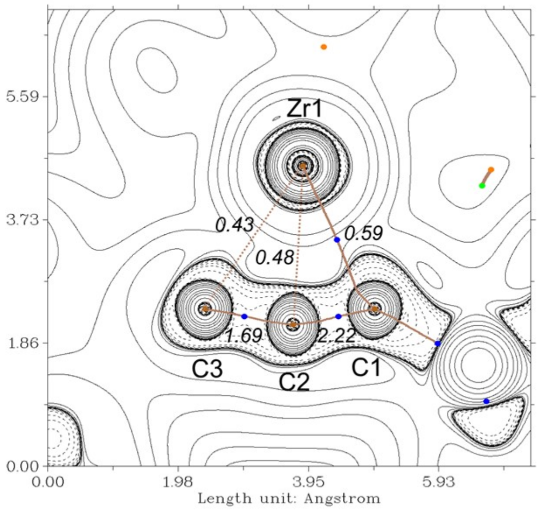

Figure S115. Contour plot of the Laplacian of the electron density ∇²*r* of complex **3a** in the C-Zr-C plane. Dashed lines indicate negative (local charge concentration), solid lines indicate positive values (local charge depletion). The Laplacian plot is overlaid with the molecular graph from QT-AIM analysis and Wiberg bond indices (italic small numbers). Brown lines indicate bond paths, brown dashed lines are hypothetical bonds, blue dots correspond to bond critical points, light brown dots indicate ring critical points. Density from B3LYP/GD3BJ/def2tzvp calculation.

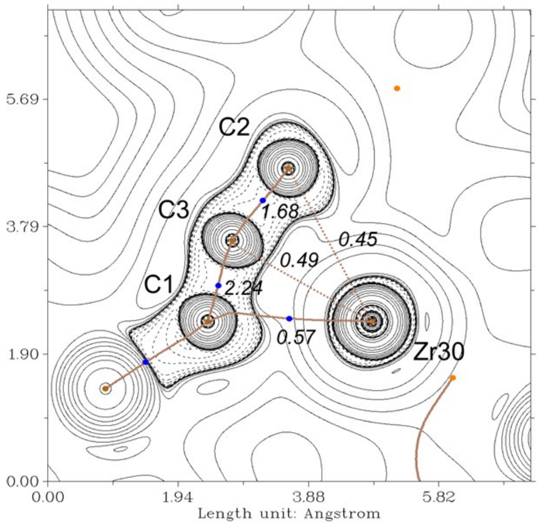

Figure S116. Contour plot of the Laplacian of the electron density ∇²*r* of complex **3b** in the C-Zr-C plane. Dashed lines indicate negative (local charge concentration), solid lines indicate positive values (local charge depletion). The Laplacian plot is overlaid with the molecular graph from QT-AIM analysis and Wiberg bond indices (italic small numbers). Brown lines indicate bond paths, brown dashed lines are hypothetical bonds, blue dots correspond to bond critical points, light brown dots indicate ring critical points. Density from B3LYP/GD3BJ/def2tzvp calculation.

8.3.7 Electron localization function of complexes 2a and 3a

To further classify the bond situation in **2a** and **3a** we analyzed the 3D plots of the electron localization functions (ELF). The main results from QT-AIM and NBO analysis are corroborated by ELF analysis (Figure S117 and Figure S119), just showing slight differences for the bond situation in the η³-propargyl/allenyl unit of complex **3a**. In both cases two orthogonal dumbbell-shaped ELF could be found along the C_3 unit which indicates two orthogonal C=C double bonds based on this theory.

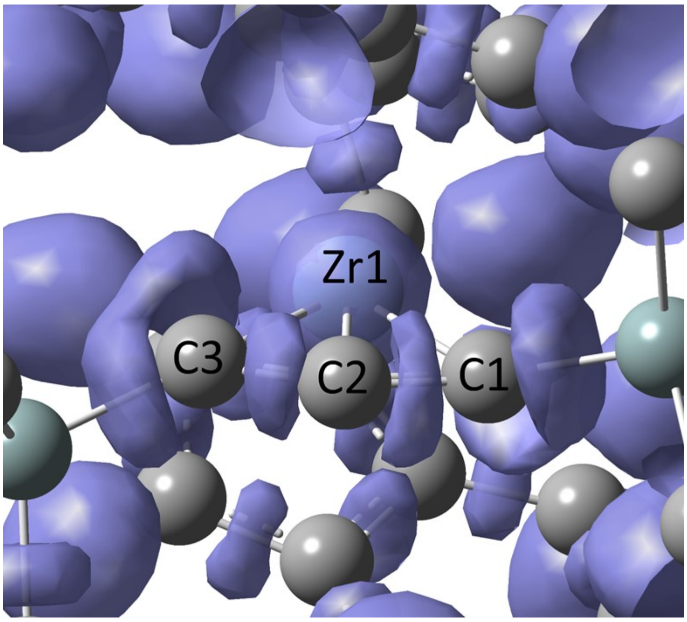

Figure S117. 3D-ELF plot of the central Zr-CCC unit in **2a**.

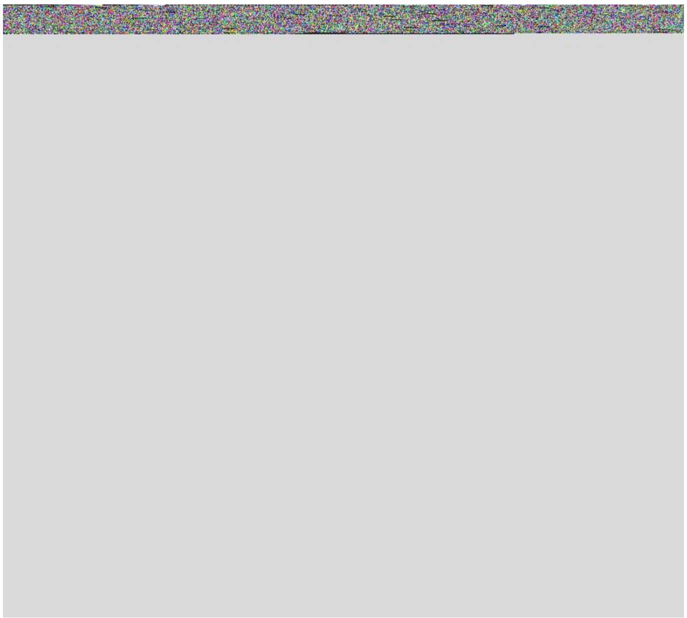

Figure S118: 2D-ELF plot of the central Zr-CCC unit in **2a**.

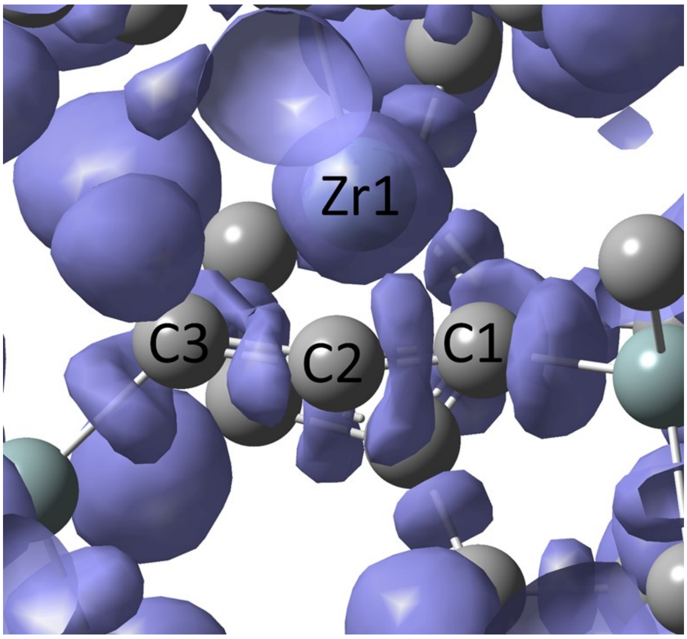

Figure S119. 3D-ELF plot of the central Zr-CCC unit in **3a**.

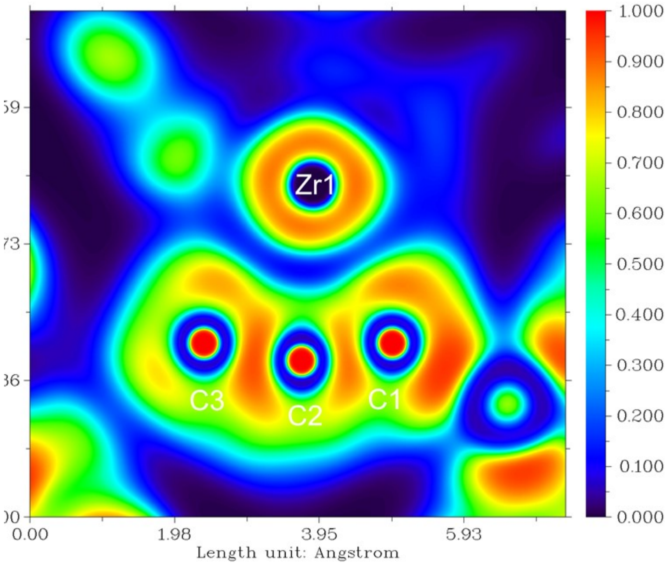

Figure S120. 2D-ELF plot of the central Zr-CCC unit in **3a**.

8.3.8 Biradical character of *rac*-(ebthi)Zr($Me_3SiCCCSiMe_3$) (**2a**)

Since the electronical situation in the 1-titanacylobuta-2,3-diene complex **A** is best described as a biradicaloid bearing a Ti(III) fragment which is antiferromagnetically coupled to a delocalized electron at the C_3 unit, we analyzed the stability of the Kohn-Sham wavefunction from B3LYP calculation. This was found to be stable, but the Hartree-Fock wavefunction shows an RHF/UHF instability for complex **2a**. To further evaluate the electronic situation of **2a** as open-shell singlet we next performed Complete Active Space (CAS) calculations.

The "broken-symmetry" solution is not a true eigenfunction of the S^2 operator. In fact, it may be considered as a 50:50 mixture of the singlet and triplet state, if the overlap between the singly occupied orbitals and spin polarization are small.[18] The actual singlet wave function can then be expressed in terms of a linear combination of *two* "broken-symmetry" wave functions

$$^1\Psi = \frac{1}{\sqrt{2}}\left(\left|\cdots\chi_+\bar{\chi}_-\right\rangle - \left|\cdots\bar{\chi}_+\chi_-\right\rangle\right)$$

where χ_+, χ_- are the singly occupied orbitals and the overline indicates β spin. Therefore, the open-shell singlet must be described by a multi-reference wave function.

In the "broken-symmetry" picture, the singly occupied orbitals χ_+ and χ_- are, in principle, localized orbitals formed by linear combinations of the (delocalized) canonical HOMO ϕ_H and LUMO ϕ_L:

$$\chi_\pm = \frac{1}{\sqrt{2}}(\phi_H \pm \phi_L)$$

Hence, the multi-reference wave function expressed in terms of the canonical MOs is given by

$$^1\Psi = c_1\left|\cdots\phi_H^2\right\rangle + c_2\left|\cdots\phi_L^2\right\rangle$$

where the expansion coefficients c_i are the square roots of the relative weight of each determinant. This type of multi-determinant open-shell singlet wave function can be obtained by the CAS SCF method and gives a qualitatively correct description of the electronic structure of a biradical. The biradical character can be evaluated as

$$\beta = \frac{2c_2^2}{c_1^2 + c_2^2}$$

where a value of β = 1 indicates a "perfect" biradical with two electrons in two degenerate orbitals.[19] Smaller values indicate an increasing energy gap between HOMO and LUMO, and $\beta \rightarrow 0$ indicates a closed-shell species.

Consequently, the smallest active space to properly describe a biradical is a CAS(2,2) calculation (*i.e.* two electrons in two orbitals). In case of compound **2a**, in analogy to the calculations on **A**, we chose to include eight electrons in nine orbitals in the active space (CAS(8,9), comprising the formal π orbitals at the ligand and d orbitals at Zr, *vide infra*), as these orbitals are energetically relatively closely spaced.

In complex **A** the largest contributions to the multi-determinant wave function are the two determinants placing two electrons either in the formal HOMO (ϕ_4) or LUMO (ϕ_5; β = 28 %), which show an occupancy of the formal LUMO with 0.34 electrons. For the Zr analog **2a** only a low coefficient for this determinant and a neglectable occupation of the formal LUMO ϕ_5 with only 0.08 electrons could be found. Based on this calculation compound **2a** show a negligible biradicaloid character and we abstain from further discussions. This is well in line with previous calculations of group 4 metallocene phosphinidenes, where the Zr derivative also showed significantly lower biradicaloid character compared to the Ti complex.[20]

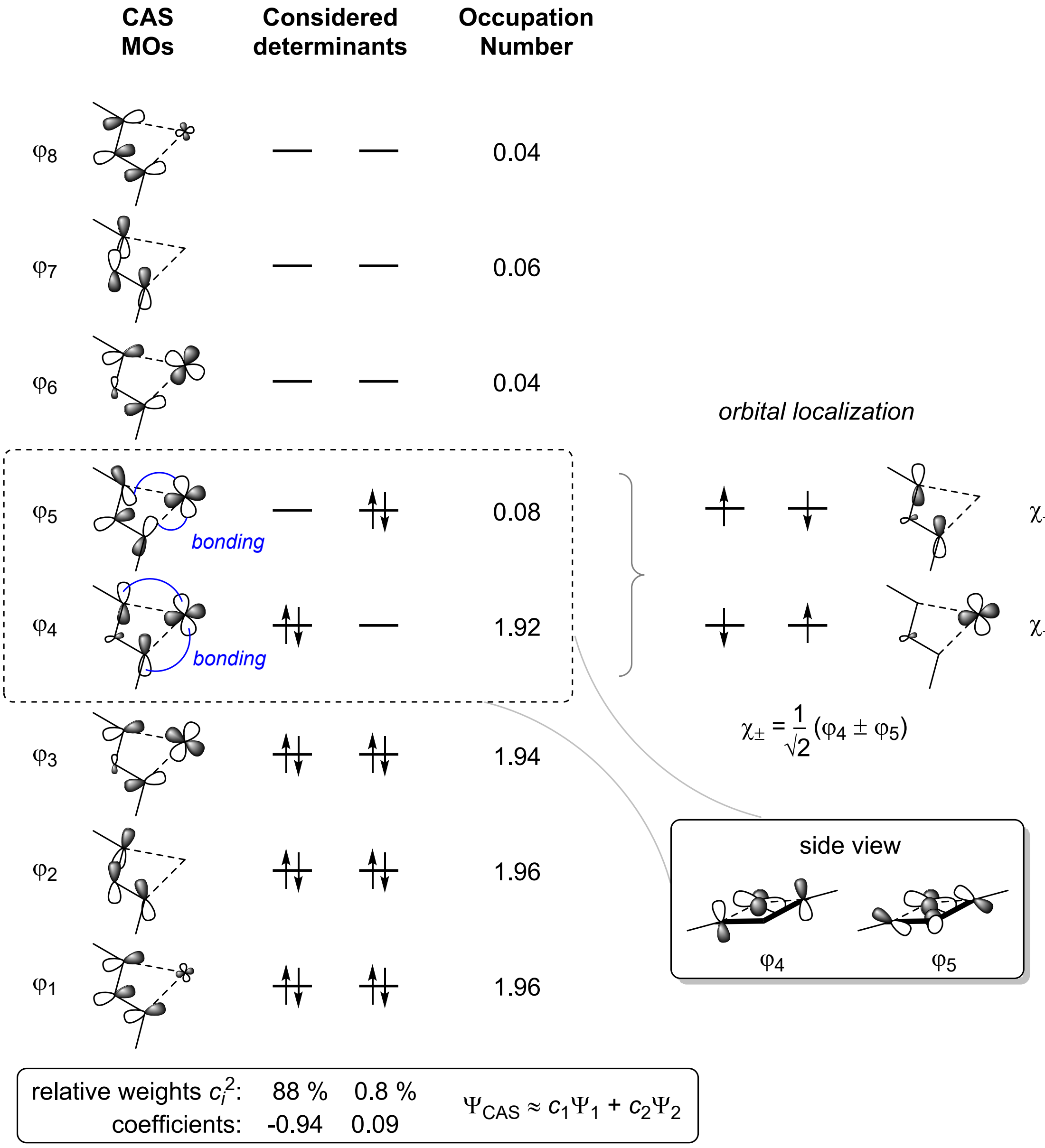

Figure S121. Schematic depiction of the active orbitals of a CAS(8,9) calculation of **2a**. The low occupation of ϕ_4 and the small coefficient c_2 reveal a negligible biradicaloid character in this molecule.

Table S21. Representation of the most important CAS(8,9) molecular orbitals of **2a**.

LUMO+3	LUMO+2
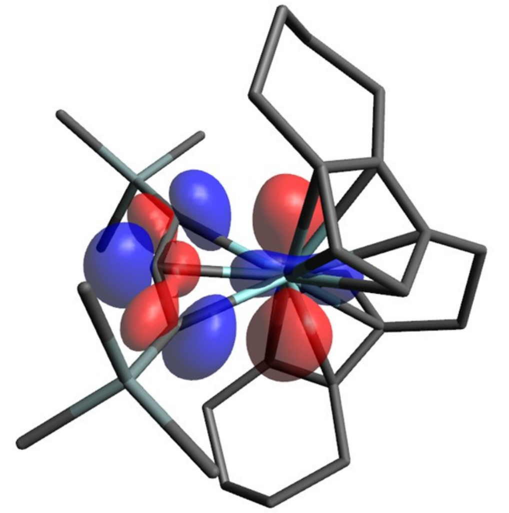	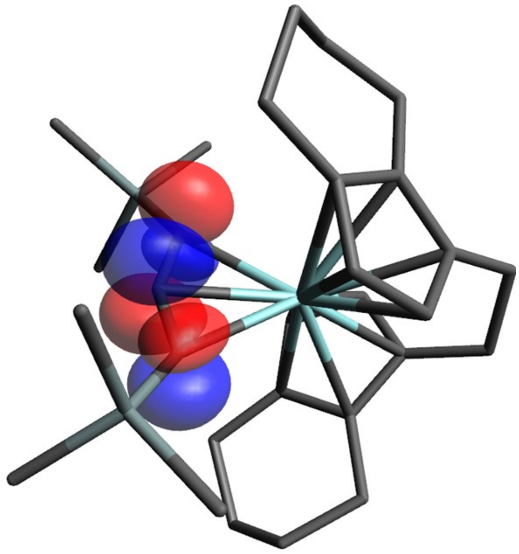
LUMO+1	LUMO
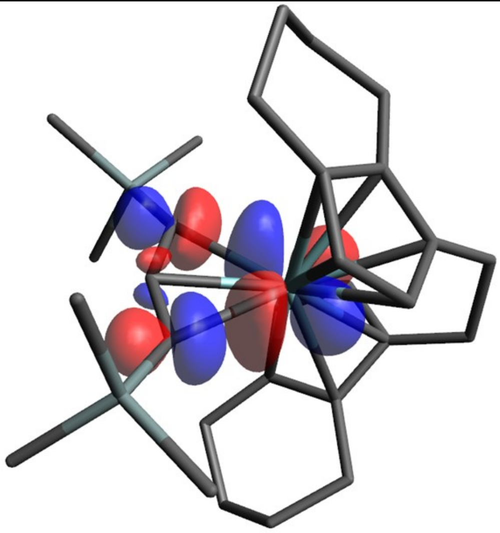	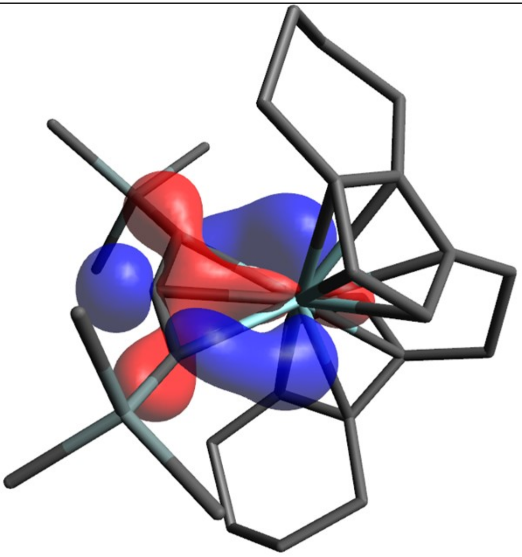
HOMO	HOMO-1

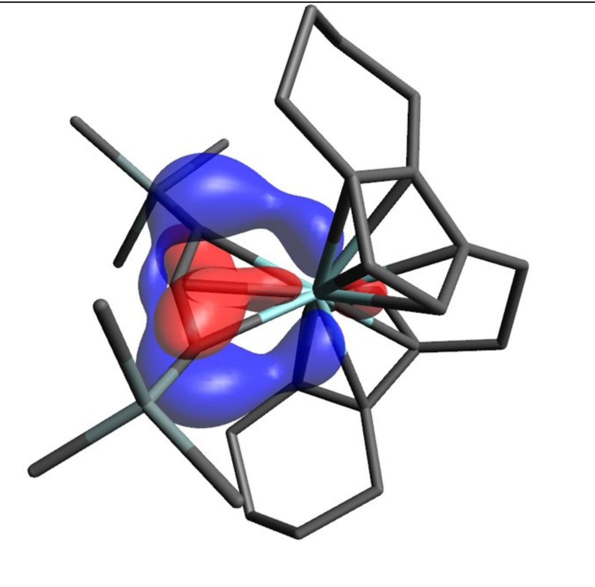	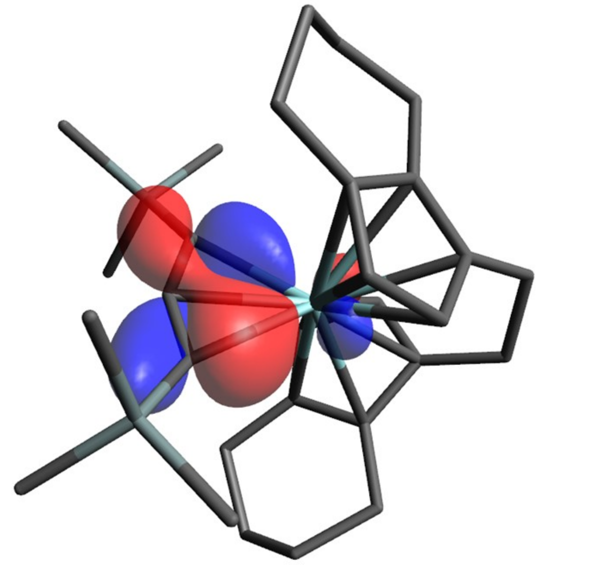
HOMO-2	HOMO-3
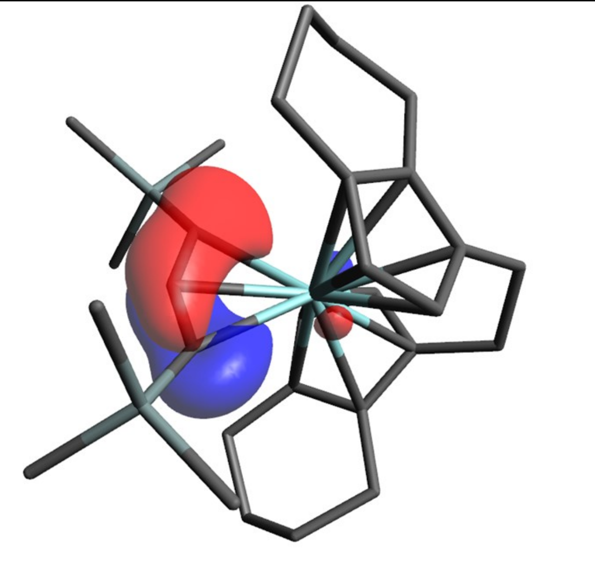	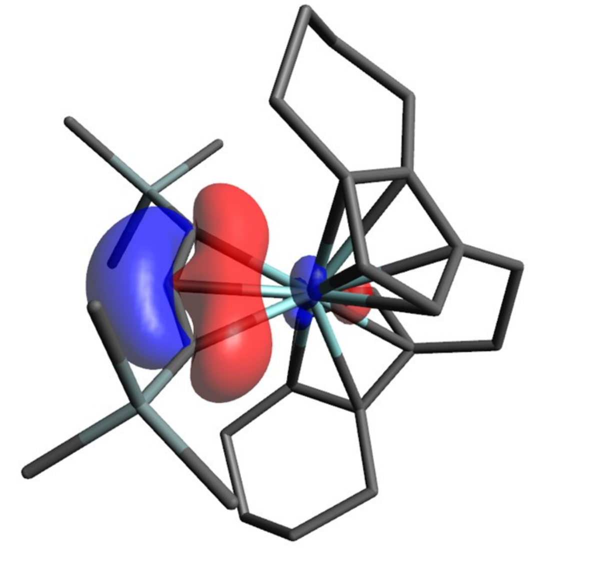

9. References

1 F. Reiß, M. Reiß, A. Spannenberg, H. Jiao, W. Baumann, P. Arndt, U. Rosenthal and T. Beweries, *Chem. Eur. J.*, 2018, **24**, 5667-5674.

2 D. Roberto, E. Cariati, R. Psaro and R. Ugo, *Organometallics*, 1994, **13**, 4227-4231.

3 G. R. Fulmer, A. J. Miller, N. H. Sherden, H. E. Gottlieb, A. Nudelman, B. M. Stoltz, J. E. Bercaw and K. I. Goldberg, *Organometallics*, 2010, **29**, 2176-2179.

4 G. M. Sheldrick, *Acta Cryst., Sect. A: Found. Crystallogr.*, 2008, **64**, 112-122.

5 G. M. Sheldrick, *Acta Cryst.*, *Sect. C: Struct. Chem.*, 2015, **71**, 3-8.

6 H. Putz and K. Brandenburg, *Diamond-Crystal and Molecular Structure Visualization*, Crystal Impact-GbR, Kreuzherrenstr. 102, 53227, Bonn, Germany, 2018.

7 F. Reiß, M. Reiß, J. Bresien, A. Spannenberg, H. Jiao, W. Baumann, P. Arndt and T. Beweries, *Chem. Sci.*, 2019, **10**, 5319-5325.

8 M. J. Frisch, G. W. Trucks, H. B. Schlegel, G. E. Scuseria, M. A. Robb, J. R. Cheeseman, G. Scalmani, V. Barone, G. A. Petersson, H. Nakatsuji, X. Li, M. Caricato, A. V. Marenich, J. Bloino, B. G. Janesko, R. Gomperts, B. Mennucci, H. P. Hratchian, J. V. Ortiz, A. F. Izmaylov, J. L. Sonnenberg, D. Williams-Young, F. Ding, F. Lipparini, F. Egidi, J. Goings, B. Peng, A. Petrone, T. Henderson, D. Ranasinghe, V. G. Zakrzewski, J. Gao, N. Rega, G. Zheng, W. Liang, M. Hada, M. Ehara, K. Toyota, R. Fukuda, J. Hasegawa, M. Ishida, T. Nakajima, Y. Honda, O. Kitao, H. Nakai, T. Vreven, K. Throssell, J. A., Jr. Montgomery, J. E. Peralta, F. Ogliaro, M. J. Bearpark, J. J. Heyd, E. N. Brothers, K. N. Kudin, V. N. Staroverov, T. A. Keith, R. Kobayashi, J. Normand, K. Raghavachari, A. P. Rendell, J. C. Burant, S. S. Iyengar, J. Tomasi, M. Cossi, J. M. Millam, M. Klene, C. Adamo, R. Cammi, J. W. Ochterski, R. L. Martin, K. Morokuma, O. Farkas, J. B. Foresman and D. J. Fox, *Gaussian 16, Rev. C.01*, Gaussian, Inc., Wallingford, CT, 2016.

9 (a) E. D. Glendening, J. K. Badenhoop, A. E. Reed, J. E. Carpenter, J. A. Bohmann, C. M. Morales, C. R. Landis and F. Weinhold, Theoretical Chemistry Institute, University of Wisconsin, Madison, 2013; (b) J. E. Carpenter and F. Weinhold, *J. Mol. Struct.: THEOCHEM*, 1988, **169**, 41-62; (c) F. Weinhold and J. E. Carpenter, in *The structure of small molecules and ions*, Springer, 1988, pp. 227-236; (d) F. Weinhold and C. R. Landis, *Valency and bonding: a natural bond orbital donor-acceptor perspective*, Cambridge University Press, 2005.

10 (a) A. D. Becke, *Phys. Rev. A*, 1988, **38**, 3098; (b) J. P. Perdew, *Phys. Rev. B*, 1986, **33**, 8822.

11 (a) S. H. Vosko, L. Wilk and M. Nusair, *Can. J. Phys.*, 1980, **58**, 1200-1211; (b) C. Lee, W. Yang and R. Parr, *Phys. Rev. B*, 1988, **37**, 785-789; (c) B. Miehlich, A. Savin, H. Stoll and H. Preuss, *Chem. Phys. Lett.*, 1989, **157**, 200-206; (d) A. D. Becke, *J. Chem. Phys*, 1993, **98**, 5648-5652.

12 F. Weigend and R. Ahlrichs, *Phys. Chem. Chem. Phys.*, 2005, **7**, 3297-3305.

13 (a) S. Grimme, J. Antony, S. Ehrlich and H. Krieg, *J. Chem. Phys.*, 2010, **132**, 154104; (b) S. Grimme, S. Ehrlich and L. Goerigk, *J. Comput. Chem.*, 2011, **32**, 1456-1465.

14 T. Lu and F. Chen, *J. Comput. Chem.*, 2012, **33**, 580-592.

15 R. Dennington, T. A. Keith and J. M. Millam, GaussView, version 6.0. 16. *Semichem Inc. Shawnee Mission KS*, 2016.

16 Mercury: http://www.ccdc.cam.ac.uk/mercury/

17 (a) R. F. Bader, *Acc. Chem. Res.*, 1985, **18**, 9-15; (b) R. F. Bader, *Chem. Rev.*, 1991, **91**, 893-928; (c) R. F. Bader, *Atoms in Molecules: A Quantum Theory*, Oxford University Press, 1994; (d) R. F. Bader, *Monatsh. Chem.*, 2005, **136**, 819-854.

18 (a) J. C. Christopher, Essentials of computational chemistry: theories and models. 2004; (b) L. Salem and C. Rowland, *Angew. Chem. Int. Ed. Engl.*, 1972, **11**, 92-111; (c) J.-P. Malrieu and G. Trinquier, *J. Phys. Chem. A*, 2012, **116**, 8226-8237.
19 E. Miliordos, K. Ruedenberg and S. S. Xantheas, *Angew. Chem. Int. Ed.*, 2013, **125**, 5848-5851.
20 M. Fischer, F. Reiß and C. Hering-Junghans, *Chem. Commun.*, 2021, **57**, 5626-5629.
